# New Uncharged 2-Thienostilbene Oximes as Reactivators of Organophosphate-Inhibited Cholinesterases

**DOI:** 10.3390/ph14111147

**Published:** 2021-11-11

**Authors:** Milena Mlakić, Tena Čadež, Danijela Barić, Ivana Puček, Ana Ratković, Željko Marinić, Kornelija Lasić, Zrinka Kovarik, Irena Škorić

**Affiliations:** 1 Department of Organic Chemistry, Faculty of Chemical Engineering and Technology, University of Zagreb, Marulićev trg 19, HR-10 000 Zagreb, Croatia; mdragojev@fkit.hr (M.M.); ivana.pucek812@gmail.com (I.P.); 2 Institute for Medical Research and Occupational Health, Ksaverska cesta 2, HR-10 000 Zagreb, Croatia; tcadez@imi.hr; 3 Group for Computational Life Sciences, Division of Physical Chemistry, Ruđer Bošković Institute, Bijenička cesta 54, HR-10 000 Zagreb, Croatia; dbaric@irb.hr; 4 Fidelta Ltd., Prilaz Baruna Filipovića 29, HR-10 000 Zagreb, Croatia; ana.ratkovic30@gmail.com; 5 NMR Center, Ruđer Bošković Institute, Bijenička cesta 54, HR-10 000 Zagreb, Croatia; zmarinic@irb.hr; 6 Pliva Tapi R&D, TEVA, Prilaz baruna Filipovića 25, HR-10 000 Zagreb, Croatia; Kornelija.Lasic@pliva.com

**Keywords:** AChE, BChE, reactivation, heterostilbenes, spectroscopy, docking

## Abstract

The inhibition of acetylcholinesterase (AChE) and butyrylcholinesterase (BChE) by organophosphates (OPs) as nerve agents and pesticides compromises normal cholinergic nerve signal transduction in the peripheral and central nervous systems (CNS) leading to cholinergic crisis. The treatment comprises an antimuscarinic drug and an oxime reactivator of the inhibited enzyme. Oximes in use have quaternary nitrogens, and therefore poorly cross the brain–blood barrier. In this work, we synthesized novel uncharged thienostilbene oximes by the Wittig reaction, converted to aldehydes by Vilsmeier formylation, and transformed to the corresponding uncharged oximes in very high yields. Eight *trans,anti*- and *trans,syn*-isomers of oximes were tested as reactivators of nerve-agent-inhibited AChE and BChE. Four derivatives reactivated cyclosarin-inhibited BChE up to 70% in two hours of reactivation, and docking studies confirmed their productive interactions with the active site of cyclosarin-inhibited BChE. Based on the moderate binding affinity of both AChE and BChE for all selected oximes, and in silico evaluated ADME properties regarding lipophilicity and CNS activity, these compounds present a new class of oximes with the potential for further development of CNS-active therapeutics in OP poisoning.

## 1. Introduction

Oximes, unlike the symptomatic drugs currently used as inhibitors of acetylcholinesterase (AChE) in the treatment of neurological disorders such as Alzheimer’s and Parkinson’s disease, have an important role for the regeneration of the cholinergic system in cases of poisoning with organophosphate compounds (OPs). OPs present a large group of chemicals used as pesticides, flame retardants, etc., and misused as nerve agents. Their non-selective species toxicity can cause severe biological effects to humans [1,2]. The related enzyme butyrylcholinesterase (BChE) is also involved in the regulation of the cholinergic system as a backup to AChE in hydrolyzing the neurotransmitter acetylcholine (ACh) in neurological disorders [3]. BChE’s role also seems to be the protection of synaptic AChE because it reacts with a variety of drugs, xenobiotics as well as with OPs, but its inhibition is safe for human health [4,5]. Emergency treatment for AChE inhibited with OPs is based on oxime reactivators that regenerate the enzyme through the nucleophilic displacement of the OP moiety conjugated at the catalytic center serine. Oxime therapy comprises anticonvulsants and antimuscarinics to manage symptoms prompted by poisoning manifested as salivation, tremors, respiratory paralysis, and in severe exposures, death [6,7]. Unfortunately, the current standard therapy in OP poisoning is still based on a small number of quaternary pyridinium compounds, 2-PAM, obidoxime, or HI-6, which are not universal reactivators due to the structural variability of OPs and their structural impotence in passing the brain–blood barrier (BBB) [7,8,9,10].

To improve the central nervous system (CNS) activity of oximes, our recent research was directed towards oximes with increased lipophilicity, uncharged oximes, and zwitterionic oximes with a tertiary nitrogen atom [9,10]. Even though uncharged oximes were usually proven to be much better in BBB penetration due to increased lipophilicity [11,12], their downfall is the low affinity as a consequence of the absence of a positive charge for productive interactions with the OP-inhibited enzyme [13,14]. This is where the concept of a peripheral site ligand (PSL) was introduced into the oxime structure as compensation for the positively charged nitrogen in order to preserve, if not increase, the affinity for the enzyme [9,10,15,16]. The introduction of the PSL strategy, which involves the coupling of uncharged pyridine oximes with different PSLs via alkyl chains, has opened up many options for new oxime structures.

In this paper, we describe the synthesis of a new uncharged thienostilbene oxime whose design was based on our long-term experience in the preparation of heterostilbenes with thiophene and furan nuclei [17]. Moreover, a study on (thiophen-2-yl)-aldoximes reported that the sulfur atom, due to its potential for polarization, helps to stabilize the negative charge of its anionic form, similarly to the pyridinium ring of the standard oxime 2-PAM [18]. Therefore, we present here the preparation of a five-membered heterocyclic thiophene nucleus connected by a double bond to the benzene ring with *para*-position substitution that could be accommodated at the peripheral site and improve binding affinity. It is worth mentioning that the double-bond introduction between two (hetero)aryl groups eliminates the possibility of a pronounced conformational change. Nevertheless, a new oxime group was obtained by an efficient and cost-effective reaction pathway involving the Wittig reaction, Vilsmeier formylation, and the formation of two configurationally stable oxime isomers. We evaluated the kinetic interaction of each isomer with both AChE and BChE, together with their reactivation potential in sarin, cyclosarin, tabun, and VX poisoning. An in silico analysis of basic physicochemical characteristics was performed to estimate the ability of novel oximes in BBB crossing, as well as to assess their potential for further biological testing.

## 2. Results and Discussion

### 2.1. Synthesis and Spectroscopic Characterization of New Thienostilbene Oximes ***13**–**18***

**Scheme 1:**
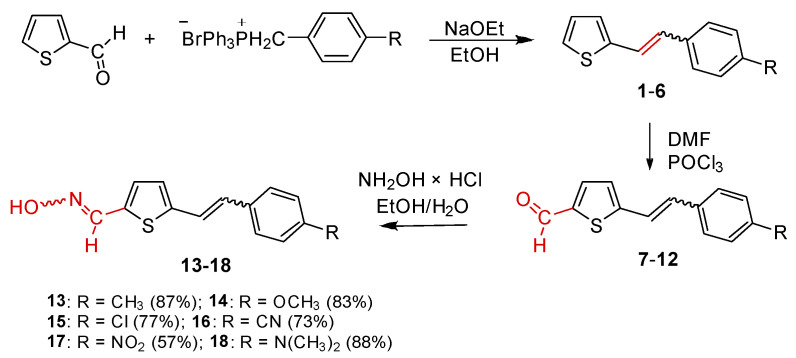
The three-step reaction pathway for obtaining heterostilbene oxime derivatives **13**–**18**. The yields are expressed for the third reaction step.

The preparation of the desired thienostilbene oximes **13**–**18** was achieved according to the three-step reaction path shown in Scheme 1. It began by forming a double bond using the Wittig reaction to yield the starting compounds **1**–**6** as mixtures of *cis*- and *trans*-isomers (48–61%). In the second step, Vilsmeier formylation provided the corresponding mixtures of aldehydes **7**–**12** (24–47%) and in the third step, formyl derivatives were transformed to oximes **13**–**18** (57–88%) as the targeted structures. Mixtures of the geometrical isomers of oximes **13**–**18** were separated to obtain pure compounds by repeated-column and thin-layer chromatography.

**Figure 1:**
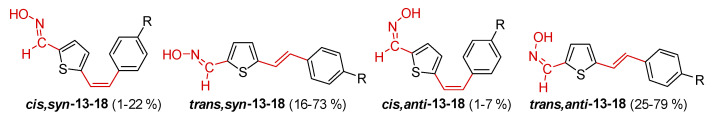
Ratio of geometrical isomers of the synthesized thienostilbene oximes **13**–**18**.

The Wittig reaction was performed using commercially available thiophene-2-carbaldehyde, and selected phosphonium salts prepared in our laboratory. The obtained thienostilbenes **1**–**6** represent the basic building blocks subjected to the Vilsmeier formylation reaction. In this reaction, POCl_3_ and DMF initially formed a chloriminium ion, a Vilsmeier reagent, which then reacted with the heterostilbene molecule, and an iminium ion was formed, which hydrolyzed to the final aldehydes **7**–**12**. We noticed that the *trans*-isomers of **1**–**6** reacted more successfully in the formylation carried out for the partially unreacted *cis*-isomers, and thus the proportion of *trans*-isomers of aldehydes **7**–**12** was significantly higher (18–30%) in comparison to those for the *cis*-isomers (1–19%). The reaction of converting the synthesized aldehydes **7**–**12** into the corresponding oximes **13**–**18** involved the use of NH_2_OH × HCl and a mixture of ethanol and water as solvents and was based on the reaction described in the literature on simpler heteroaromatic systems [18]. According to ^1^H NMR spectroscopic analyses, *trans*-isomers are more present in the reaction mixtures of oxime derivatives **13**–**18** (Figure 1). In the case of oximes **13**–**15** and **18** possessing CH_3_, OCH_3_, and Cl groups as substituents, respectively, *trans,anti*-isomers dominate. Oximes **16** and **17** with electron-withdrawing substituents CN and NO_2_, respectively, have the most *trans,syn*-isomers in the reaction mixture, while in the case of the N(CH_3_)_2_ substituent, the ratio of *syn*- and *anti*-isomers was 2:1, with the presence of *cis,syn*-**18** at the same abundance as that of *trans,syn*-**18** (See Experimental Section) [19].

**Figure 2:**
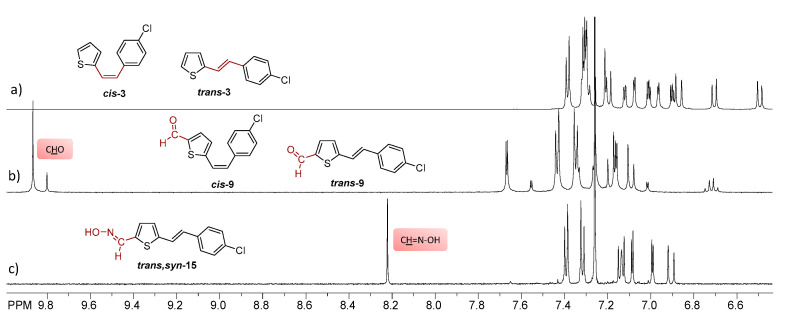
Downfield region of the ^1^H NMR spectra (CDCl_3_) of the mixture of *cis*-**3** and *trans*-**3** (**a**), mixture of *cis*-**9** and *trans*-**9** (**b**), and oxime *trans,syn*-**15** (**c**).

**Figure 3:**
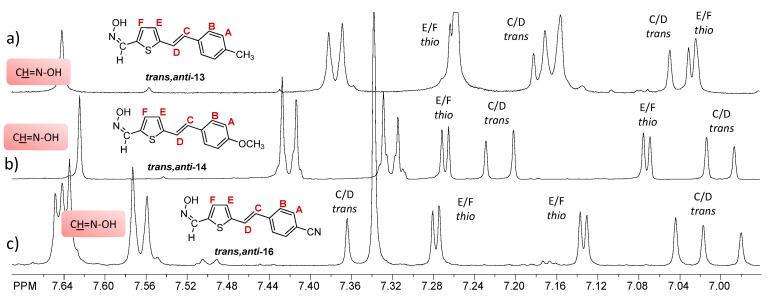
Downfield region of the ^1^H NMR spectra (CDCl_3_+CD_3_OD) of the oximes *trans,anti*-**13** (**a**), *trans,anti*-**14** (**b**), and *trans,anti*-**16** (**c**).

After successive column and thin-layer chromatography, the *trans,anti*- and *trans, syn*-isomers of the individual oxime derivatives **13**–**18** were isolated at a sufficient quantity to test them as potential cholinesterase reactivators (see Section 2.3). During the experiment, it was found that the *anti*-isomers of oximes are more polar and less soluble than those of *syn*-isomers, and due to the difference in their R*_f_* values, they are relatively easily isolated. The similarity of the R*_f_* values of the *cis*- and *trans*-isomers of the *syn*-oximes influenced the lower success of their separation. All of the isolated isomers of oxime **13**–**18** were fully spectroscopically characterized (See Figure 2 and Figure 3, Experimental Section and Supplementary Materials).

Additionally, from the reaction mixtures after the Wittig and Vilsmeier reaction, *cis*- and *trans*-isomers of thienostilbene **1**–**6** as well as the *cis*- and *trans*-isomers of the formylation products **7**–**12** were spectroscopically characterized and given for analysis by HRMS (See Experimental Section and Supplementary Materials).

### 2.2. In Silico Prediction of ADME Properties of Thienostilbene Oximes ***13**–**18***

The structure–property relationships [20,21] with predictive models for calculated compounds **13**–**18** were also analyzed by ACD/Percepta (ver. 14.2.0; Build 2977; ACD/Laboratories, Toronto, ON, Canada), which does not consider the geometrical isomerization. Physico-chemical properties were calculated, specifically the logP values, solubility, and permeability across biological membranes (Table 1). From a medicinal chemistry perspective, the design of drugs capable of penetrating the blood–brain barrier (BBB) and effecting the desired biological response is a formidable challenge. Moderately lipophilic drugs cross the BBB by passive diffusion, and the hydrogen bonding properties of drugs can significantly influence their CNS profiles. Polar molecules are generally poor CNS agents unless they undergo active transport across the CNS. Size, ionization properties, and molecular flexibility are other factors observed to influence the transport of an organic compound [22]. Lipophilicity is one of the most important physical properties that can affect the potency, distribution, and elimination of a drug in the body [23].

In general, the solubility of selected oximes **13**–**18** was low. Nitro derivative **17** and dimethylamino derivative **18** showed the highest solubility (0.01 mg/mL). All of the compounds showed an optimal lipophilicity value, meaning they should readily penetrate the BBB, since the majority of CNS drugs have logP within the range of 3.5 to 4.5 (ACD/Percepta (ver. 14.2.0; Build 2977; ACD/Laboratories, Toronto, ON, Canada). Moreover, estimations of the CNS factor displayed their high penetrant ability. Plasma protein binding (PPB) results showed high bioavailability and a long biological half-life because the bound portion may act as a reservoir from which the drug is slowly released in the unbound form to maintain equilibrium. Nevertheless, based on these in silico ADME analyses, chloro derivative **15** seems to be the one with the least-desired physico-chemical properties for a CNS active drug, yet the solubility of all thienostilbenes require improvement in the future.

### 2.3. In Vitro Evaluation of Thienostilbene Oximes ***13**–**18*** in Reactions with Cholinesterases

Eight thienostilbene oxime derivatives **13**–**18** were tested as reversible inhibitors of AChE and BChE, and dissociation constants are given in Table 2. Generally, all of the oximes were moderate reversible inhibitors of both enzymes with *K*_i_ constants in the micromolar range. All of the oximes, except *trans,anti*-**16,** were more potent inhibitors of AChE than BChE. Out of all of the tested oximes, BChE also exhibited higher potency for *cis,syn*-**18,** which has nitrogen at the substituent, as well as for *trans,anti*-**16.** In the case of AChE, it seems that the CN substituent affected the binding affinity, as *K*_i_ for *trans,anti*-**16** is up to 10-fold higher than for the most potent inhibitor of AChE *trans,syn*-**15**. In addition, inhibition of AChE was uncompetitive for all compounds, except for *trans,syn*-**15,** while all of the compounds exhibited competitive binding for BChE.

**Figure 4:**
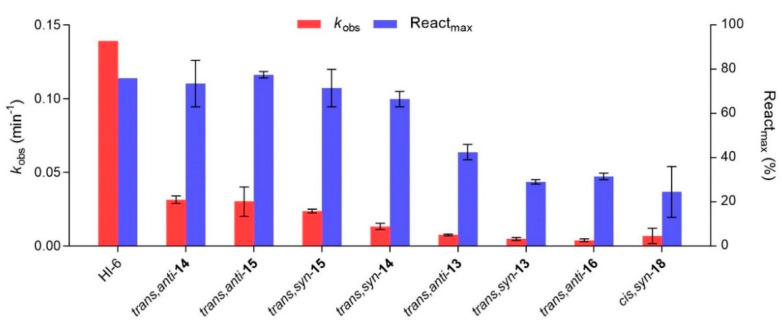
Reactivation of cyclosarin-inhibited human BChE with eight thienostilbene oxime derivatives and standard oxime HI-6 (0.1 mM) given as observed reactivation constant (*k*_obs_) and maximal reactivation in 22 h.

The reactivation of human AChE and BChE inhibited with nerve agents (sarin, cyclosarin, VX, and tabun) was screened with one concentration of thienostilbene oxime derivatives (0.1 mM). In the case of AChE inhibited with any of the four nerve agents, no reactivation higher than 20% was observed within 5 h, which does not present an improvement over TMB-4, 2-PAM, HI-6, and obidoxime in the case of AChE inhibited with tabun, VX, sarin, and cyclosarin, respectively. Unfortunately, reactivation could not be tested with a higher concentration due to poor solubility and solvent DMSO, which affects AChE activity. In the case of BChE, only the cyclosarin-inhibited BChE was prone to reactivation, and Figure 4 shows the percentage of maximal reactivation and the observed reactivation rate with 0.1 mM oximes. The fastest reactivation was observed with *trans,anti*-**15** and *trans,anti*-**14**, while with their *trans,syn*-counterparts follow. These four derivatives reactivated cyclosarin-inhibited BChE up to 70% in two hours of reactivation. Although the maximal percentage of reactivation was similar to the standard oxime HI-6, the observed reactivation rate was about four-fold slower than that obtained for HI-6. BChE inhibited by other nerve agents was resistant to reactivation with these compounds.

Nevertheless, oximes with 4-methoxyphenyl and 4-chlorophenyl substituents **14** and **15** seem to be successful in the reactivation of cyclosarin-inhibited human BChE. These findings are in accordance with our recent studies with quinuclidine-3 oximes from which the most potent reactivators of cyclosarin-inhibited BChE were bromo- or chlorobenzyl oximes [24].

### 2.4. Docking of Potential Reactivators

To gain insight into the structure of the enzyme–inhibitor complex after the oxime approaches the active site, we performed docking of thienostilbene oximes into inhibited cholinesterase. According to our experimental results, the significant reactivation ability of tested thienostilbene oximes is found only for cyclosarin-inhibited BChE; therefore, we conducted docking of the compounds of interest into the active site of BChE inhibited by cyclosarin. It should be mentioned that, unlike the cyclosarin-inhibited AChE, the crystal structure of cyclosarin-inhibited BChE is not available in the literature. To solve this obstacle, we tried to use the conformation of cyclosarin bound to AChE (from PDB structure 3ZLU [25]) and incorporate it within the active site of BChE. However, because of the differences between active sites of BChE and AChE, specifically, due to the positioning of His438 in BChE, this approach resulted in sterically unfavorable interactions (Figure S1). Cuya et al. performed a molecular dynamics study [26] to obtain the most realistic inhibitor conformation, but they also used cyclosarin-inhibited AChE. Therefore, in our work, the experimentally obtained crystal structure of BChE inhibited by tabun is utilized, replacing the dimethylamino and ethoxy groups of tabun with methyl and cyclohexyloxy groups of cyclosarin, respectively. The orientation of replaced substituents in the resulting cyclosarin conformation was as similar to the structure in AChE as possible, given the steric limitations in the active site of BChE (Figure S2).

**Figure 5:**
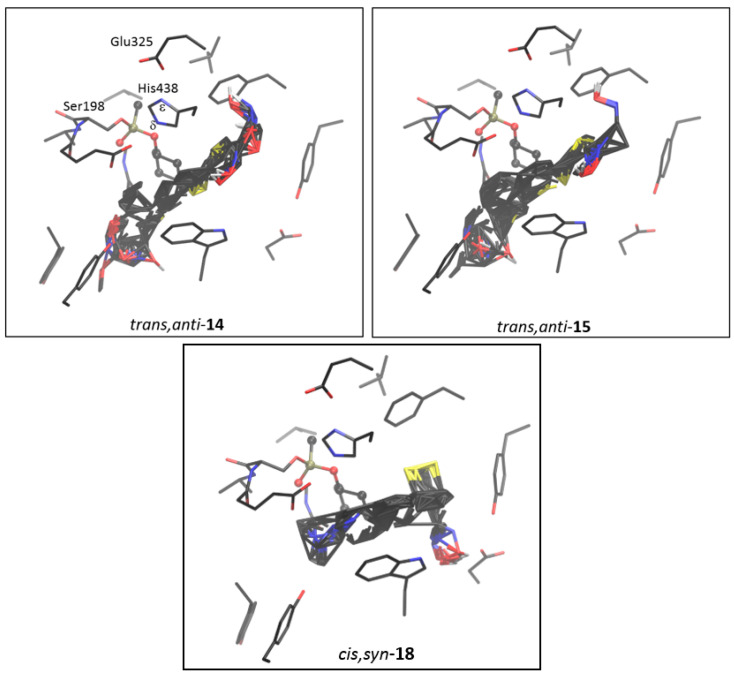
Structures of the active site of cyclosarin-inhibited BChE with docked thienostilbene oximes: *trans,anti*-**14**, *trans,anti*-**15**, and *cis,syn*-**18**. Cyclosarin (presented using the ball and stick model) is covalently bound to Ser198.

As the compounds with the best reactivating capability, *trans,anti*-**14** and *trans,anti*-**15** were chosen for docking into the active site of cyclosarin-inhibited BChE. For the sake of comparison, we also performed docking of the poor reactivator *cis,syn-***18**. The structures of the active site of cyclosarin-inhibited BChE with docked thienostilbene oximes are shown in Figure 5. For each of these three oximes, all conformations obtained by docking into the inhibited active site are superimposed, so the most abundant orientation of the reactivator is clearly depicted.

**Figure 6:**
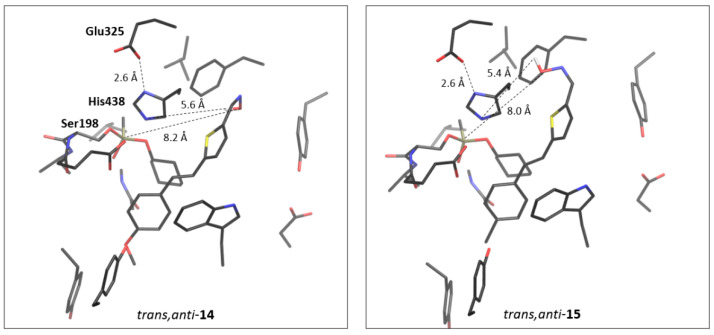
The most favorable conformation of *trans,anti*-**14** and *trans,anti*-**15** docked into the active site of cyclosarin-inhibited BChE.

Molecules **14** and **15** take various conformations, but it is visible from the presentations in Figure 5 that, in most cases, the oxime group is oriented toward His438. In contrast, for the molecule *cis,syn*-**18**, its oxime group is always oriented in the opposite direction. The orientation of the oxime group is relevant for the reactivation, as well as its proximity to His438 and the phosphorus atom of cyclosarin, because the first step of reactivation is a nucleophilic attack of the oxygen of the oxime group to phosphorus. Since the oximes in this study entered the active site in neutral form, the first step of efficient reactivation should be preceded by the total (or at least partial) deprotonation of the oxime group. This deprotonation is possible only if the proton of the oxime is in a sterically favorable position to the unprotonated nitrogen atom of His438. Although neutral histidine is a weak base and thus unable to deprotonate the oxime group, here it is placed close to Glu325, which accepts the proton of the epsilon nitrogen and thus facilitates proton transfer from the oxime to the delta nitrogen of histidine. Figure 5 shows that none of the conformations obtained by docking of *cis,syn*-**18** are not in a favorable position to proceed with reactivation. In contrast, for **14** and **15,** most conformers are placed so as to enable reactivation. In Figure 6, the most favorable conformations of docked molecules **14** and **15** are presented, along with some geometry parameters.

Figure 6 shows that the structures of the complexes formed between *trans,anti-***14** and *trans,anti*-**15** with the active site of cyclosarin-inhibited BChE are favorable for the reactivation of the enzyme. As already mentioned, the thienostilbene oximes studied here are not in ionic form; this is important because neutral oximes have an improved ability to cross the blood–brain barrier compared to charged ones. However, to be able to break the covalent P-O bond between the inhibitor and catalytic Ser198, the oxime should be totally or partially deprotonated by histidine, such as in the semi-empirical calculations of BChE reactivation reported recently [27]. Therefore, the proximity of the oxime to histidine along with the position of the oxime group, which provides the absence of steric hindrance for the oxygen attack of phosphorus, are crucial for efficient reactivation.

In the static structure obtained by docking, the distance between the covalently bound inhibitor and oxygen of the neutral oxime is too big to enable the reaction to start immediately (the nucleophilic attack of oxygen to phosphorus). However, the favorable orientation of oxime and its proximity to His438 make this process feasible. To additionally scrutinize this conclusion, we performed scanning of the Potential Energy Surface (PES), utilizing quantum chemistry calculations (see details in section Materials and Methods) on a small-model system consisting of His448, Glu325, and Ser198 with covalently bound cyclosarin and thienostilbene oxime *trans,anti*-**15**. Scanning the PES involved crude geometry optimization for the set of structures obtained by an incremental (0.1 Å) decrease in the distance between the oxygen of oxime and phosphorus, *d*(O_Oxime_-P). The starting value of the distance was 8 Å. The system’s total energy also decreased until the oxygen approached phosphorus at *d* = 4.3 Å (Figure S3 and Table S1). Further shortening the *d*(O_Oxime_-P) did not cause significant energy change until the oxygen was 3 Å from phosphorus. It may be concluded that the approximate equilibria distance *d*(O_Oxime_-P) before the start of reaction between oxime and cyclosarin is between 4 and 3 Å. It is important to emphasize that this structure may be achieved spontaneously (the process being exothermic, and more relevant, without energy barriers), starting from the first geometry obtained by docking.

The investigation performed in this section does not aim to answer how different substituents at the phenyl unit of thienostilbene oximes affect their reactivating ability. However, the presented results indicate that *trans,anti*-thienostilbene oximes have a conformational advantage compared to their *cis,syn*-counterparts, thus rationalizing the experimental findings shown in Figure 4.

## 3. Materials and Methods

### 3.1. General

Petroleum ether (PE), bp 40–60 °C, was used. Solvents were purified by distillation. Column chromatography was carried out on columns with silica gel (Fluka 0.063–0.2 nm and Fluka 60 Å, technical grade). TLC was carried out using plates coated with silica gel (0.2 mm, Kiselgel 60 F_254_). Organic layers were routinely dried with anhydrous MgSO_4_ and evaporated using a rotary evaporator. ^1^H and ^13^C NMR spectra were recorded on a spectrometer at 600 MHz. All NMR spectra were measured in CDCl_3_ using tetramethylsilane as a reference. The following abbreviations were used in the NMR spectra: s (singlet), d (doublet), t (triplet), q (quartet), dd (doublet of doublets), and m (multiplet). For the full characterization of targeted oximes, the additional techniques 2D-CH correlation (HSQC) and 2D-HH-COSY were used. Mass spectra were obtained on a UPLC-MS system. Melting points were obtained using a microscope-equipped apparatus and have not been corrected. HRMS analyses were carried out on a mass spectrometer (MALDI TOF/TOF analyzer), equipped with an Nd:YAG laser operating at 355 nm with a firing rate of 200 Hz in the positive (H+) or negative (-H) ion reflector mode. The initial phosphonium salts were prepared in the laboratory from the corresponding bromides, while the other starting compounds used were purchased chemicals.

### 3.2. Synthesis of Thienostilbenes ***1**–**6***

Thienostilbenes **1**–**6** were prepared by the Wittig reaction. The corresponding phosphonium salt (11 mmol) was dissolved in 130 mL of EtOH (dried on 4 Å sieves) in a three-necked flask. The appropriate aldehyde (10 mmol) was added. The content of the flask was mixed on a magnetic stirrer and purged with nitrogen. The reaction mixture was heated to 40 °C for 30 min. The NaOEt obtained from Na (11 mmol) reacted with dry EtOH (10 mL) and was added dropwise to the flask. Upon completion of the dropwise addition, the system was closed, and the reaction mixture was stirred on a magnetic stirrer overnight at room temperature. EtOH was applied to a rotary evaporator under reduced pressure. Extraction was carried out in a system of toluene and water, after which the organic layer was dried over MgSO_4_, filtered, and the toluene was evaporated. The dry reaction mixture was purified by column chromatography on silica gel using a PE/E or PE/DCM variable polarity eluent. The column chromatography yielded mixtures of *cis*- and *trans*-isomers of the desired products **1**–**6**, which were used as reactants in further Vilsmeier formylation reactions. The spectroscopic data for the geometrical isomers of compounds **1**–**6** were obtained from the ^1^H NMR spectra of the mixtures.

 
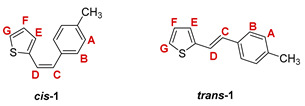


***cis*-2-(4-methylstyryl)thiophene (*cis*-1)** [28]: 404 mg (18.4%); *R_f_* (petroleum ether) = 0.71; ^1^H NMR (CDCl_3_, 600 MHz) *δ*/ppm: 7.14–7.21 (m, 4H, H_A_, H_B_), 7.09 (d, 1H, *J* = 4.7 Hz, H_G_), 6.98 (d, 1H, *J* = 3.6 Hz, H_E_), 6.89 (dd, 1H, *J* = 4.7; 3.6 Hz, H_F_), 6.66 (d, 1H, *J* = 12.1 Hz, H_C/D_), 6.54 (d, 1H, *J* = 12.1 Hz, H_C/D_), 2.37 (s, 3H, -CH_3_);

***trans*-2-(4-methylstyryl)thiophene (*trans*-1)** [28,29]: 810 mg (36.8%); *R_f_* (petroleum ether) = 0.57; ^1^H NMR (CDCl_3_, 600 MHz) *δ*/ppm: 7.37 (d, 2H, *J* = 7.8 Hz, H_A/B_), 7.26 (d, 1H, *J* = 5.2 Hz, H_G_), 7.21-7.14 (m, 3H, H_C/D_, H_A/B_), 7.05 (d, 1H, *J* = 3.6 Hz, H_E_), 7.00 (dd, 1H, *J* = 5.2; 3.6 Hz, H_F_), 6.91 (d, 1H, *J* = 16.0 Hz, H_C/D_), 2.36 (s, 3H, -CH_3_);

MS (ESI) *m/z* (%, fragment): 201 (100, M+H^+^); HRMS (*m/z*) for C_13_H_12_S (obtained for the pure mixture of geometrical isomers): [M+H]^+^_calcd_ = 201.0659, [M+H]^+^_measured_ = 201.0660.

 
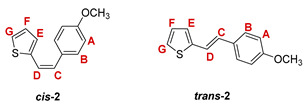


***cis*-2-(4-methoxystyryl)thiophene (*cis*-2)** [30]: 403 mg (17.0%); *R_f_* (petroleum ether/diethyl ether (1%)) = 0.30; ^1^H NMR (CDCl_3_, 600 MHz) *δ*/ppm: 7.29 (d, 2H, *J* = 8.7 Hz, H_A/B_), 7.09 (d, 1H, *J* = 4.9 Hz, H_G_), 6.98 (dd, 1H, *J* = 4.9; 3.6 Hz, H_F_), 6.98 (d, 1H, *J* = 3.6 Hz, H_E_), 6.87 (d, 2H, *J* = 8.7 Hz, H_A/B_), 6.63 (d, 1H, *J* = 11.8 Hz, H_C/D_), 6.51 (d, 1H, *J* = 11.8 Hz, H_C/D_), 3.83 (s, 3H, -OCH_3_);

***trans*-2-(4-methoxystyryl)thiophene (*trans*-2)** [29]: 803 mg (33.8%); *R_f_* (petroleum ether/diethyl ether (1%)) = 0.23; ^1^H NMR (CDCl_3_, 600 MHz) *δ*/ppm: 7.40 (d, 2H, *J* = 8.4 Hz, H_A/B_), 7.15 (d, 1H, *J* = 4.9 Hz, H_G_), 7.10 (d, 1H, *J* = 15.9 Hz, H_C/D_), 7.02 (d, 1H, *J* = 3.6 Hz, H_E_), 7.01 (d, 1H, *J* = 15.9 Hz, H_C/D_), 6.89 (d, 2H, *J* = 8.4 Hz, H_A/B_), 6.88 (dd, 1H, *J* = 4.9; 3.6 Hz, H_F_), 3.83 (s, 3H, -OCH_3_);

MS (ESI) *m/z* (%, fragment): 217 (100, M+H^+^); HRMS (*m/z*) for C_13_H_12_OS (obtained for the pure mixture of geometrical isomers): [M+H]^+^_calcd_ = 217.0609, [M+H]^+^_measured_ = 217.0605.

 
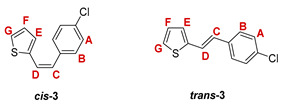


***cis*-2-(4-chlorostyryl)thiophene (*cis*-3)**: 591 mg (24.4%); *R_f_* (petroleum ether) = 0.75; ^1^H NMR (CDCl_3_, 600 MHz) *δ*/ppm: 7.33-7.28 (m, 4H, H_A/B_), 7.12 (d, 1H, *J* = 5.0 Hz, H_G_), 6.97 (d, 1H, *J* = 3.6 Hz, H_E_), 6.90 (dd, 1H, *J* = 5.0; 3.6 Hz, H_F_), 6.71 (d, 1H, *J* = 11.8 Hz, H_C/D_), 6.50 (d, 1H, *J* = 11.8 Hz, H_C/D_);

***trans*-2-(4-chlorostyryl)thiophene (*trans*-3)** [29]: 629 mg (26.0%); *R_f_* (petroleum ether) = 0.65; ^1^H NMR (CDCl_3_, 600 MHz) *δ*/ppm: 7.39 (d, 2H, *J* = 8.5 Hz, H_A/B_), 7.33-7.28 (m, 2H, H_A/B_), 7.21 (d, 1H, *J* = 5.0 Hz, H_G_), 7.20 (d, 1H, *J* = 16.2 Hz, H_C/D_), 7.08 (d, 1H, *J* = 3.4 Hz, H_E_), 7.01 (dd, 1H, *J* = 5.0; 3.4 Hz, H_F_), 6.87 (d, 1H, *J* = 16.2 Hz, H_C/D_);

MS (ESI) *m/z* (%, fragment): 221/223 (80, M+H^+^), 186 (100); HRMS (*m/z*) for C_12_H_9_ClS (obtained for the pure mixture of geometrical isomers): [M+H]^+^_calcd_ = 221.0112, [M+H]^+^_measured_ = 221.0114.

 
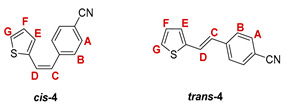


***cis*-4-(2-(thiophen-2-yl)vinyl)benzonitrile (*cis*-4)**: 831 mg (35.8%); *R_f_* (petroleum ether/diethyl ether (30%)) = 0.52; ^1^H NMR (CDCl_3_, 600 MHz) *δ*/ppm: 7.63 (d, 2H, *J* = 8.0 Hz, H_A/B_), 7.47 (d, 2H, *J* = 8.0 Hz, H_A/B_), 7.14 (d, 1H, *J* = 4.9 Hz, H_G_), 6.98 (d, 1H, *J* = 3.6 Hz, H_E_), 6.92 (dd, 1H, *J* = 4.9; 3.6 Hz, H_F_), 6.79 (d, 1H, *J* = 12.0 Hz, H_C/D_), 6.52 (d, 1H, *J* = 12.0 Hz, H_C/D_);

***trans*-4-(2-(thiophen-2-yl)vinyl)benzonitrile (*trans*-4)** [29]: 585 mg (25.2%); *R_f_* (petroleum ether/diethyl ether (30%)) = 0.43; ^1^H NMR (CDCl_3_, 600 MHz) *δ*/ppm: 7.62 (d, 2H, *J* = 8.3 Hz, H_A/B_), 7.53 (d, 2H, *J* = 8.3 Hz, H_A/B_), 7.34 (d, 1H, *J* = 16.2 Hz, H_C/D_), 7.28 (d, 1H, *J* = 5.1 Hz, H_G_), 7.15 (d, 1H, *J* = 3.5 Hz, H_E_), 7.04 (dd, 1H, *J* = 5.1; 3.5 Hz, H_F_), 6.90 (d, 1H, *J* = 16.2 Hz, H_C/D_);

MS (ESI) *m/z* (%, fragment): 212 (100, M+H^+^); HRMS (*m/z*) for C_13_H_9_NS (obtained for the pure mixture of geometrical isomers): [M+H]^+^_calcd_ = 212.0458, [M+H]^+^_measured_ = 212.0456.

 
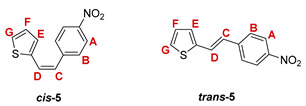


***cis*-2-(4-nitrostyryl)thiophene (*cis*-5)**: 582 mg (22.9%); *R_f_* (petroleum ether/diethyl ether (15%)) = 0.63; ^1^H NMR (CDCl_3_, 600 MHz) *δ*/ppm: 8.19 (d, 2H, *J* = 8.4 Hz, H_A/B_), 7.53 (d, 2H, *J* = 8.4 Hz, H_A/B_), 7.17 (d, 1H, *J* = 5.2 Hz, H_G_), 6.99 (d, 1H, *J* = 3.5 Hz, H_E_), 6.99 (dd, 1H, *J* = 5.2; 3.5 Hz, H_F_), 6.83 (d, 1H, *J* = 12.0 Hz, H_C/D_), 6.55 (d, 1H, *J* = 12.0 Hz, H_C/D_);

***trans*-2-(4-nitrostyryl)thiophene (*trans*-5)** [29]: 630 mg (24.8%); *R_f_* (petroleum ether/diethyl ether (15%)) = 0.51; ^1^H NMR (CDCl_3_, 600 MHz) *δ*/ppm: 8.21 (d, 2H, *J* = 8.6 Hz, H_A/B_), 7.58 (d, 2H, *J* = 8.6 Hz, H_A/B_), 7.39 (d, 1H, *J* = 16.1 Hz, H_C/D_), 7.30 (d, 1H, *J* = 5.2 Hz, H_G_), 7.18 (d, 1H, *J* = 3.7 Hz, H_E_), 7.05 (dd, 1H, *J* = 5.2; 3.7 Hz, H_F_), 6.95 (d, 1H, *J* = 16.1 Hz, H_C/D_);

MS (ESI) *m/z* (%, fragment): 232 (100, M+H^+^); HRMS (*m/z*) for C_12_H_9_NO_2_S (obtained for the pure mixture of geometrical isomers): [M+H]^+^_calcd_ = 232.0356, [M+H]^+^_measured_ = 232.0354.

 
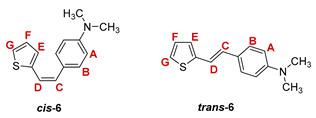


***cis*-*N*,*N*-dimethyl-4-(2-(thiophen-2-yl)vinyl)aniline (*cis*-6)** [31]: 524 mg (20.8%); *R_f_* (petroleum ether/diethyl ether (30%)) = 0.55; ^1^H NMR (CDCl_3_, 600 MHz) *δ*/ppm: 7.28 (d, 2H, *J* = 8.7 Hz, H_A/B_), 7.10 (d, 1H, *J* = 5.2 Hz, H_G_), 7.02 (d, 1H, *J* = 3.6 Hz, H_E_), 6.91 (dd, 1H, *J* = 5.2; 3.6 Hz, H_F_), 6.69 (d, 2H, *J* = 8.7 Hz, H_A/B_), 6.54 (d, 1H, *J* = 11.9 Hz, H_C/D_), 6.49 (d, 1H, *J* = 11.9 Hz, H_C/D_), 2.98 (s, 6H, -N(CH_3_)_2_);

***trans*-*N*,*N*-dimethyl-4-(2-(thiophen-2-yl)vinyl)aniline (*trans*-6)** [31]: 630 mg (25.0%); *R_f_* (petroleum ether/diethyl ether (30%)) = 0.47; ^1^H NMR (CDCl_3_, 600 MHz) *δ*/ppm: 7.37 (d, 2H, *J* = 8.7 Hz, H_A/B_), 7.12 (d, 1H, *J* = 4.7 Hz, H_G_), 7.05 (d, 1H, *J* = 16.0 Hz, H_C/D_), 6.99 (d, 1H, *J* = 3.6 Hz, H_E_), 6.98 (dd, 1H, *J* = 4.7; 3.6 Hz, H_F_), 6.88 (d, 1H, *J* = 16.0 Hz, H_C/D_), 6.71 (d, 2H, *J* = 8.7 Hz, H_A/B_), 2.99 (s, 6H, -N(CH_3_)_2_);

MS (ESI) *m/z* (%, fragment): 230 (100, M+H^+^); HRMS (*m/z*) for C_14_H_15_NS (obtained for the pure mixture of geometrical isomers): [M+H]^+^_calcd_ = 230.0929, [M+H]^+^_measured_ = 230.0925.

### 3.3. Synthesis of Aldehydes ***7**–**12***

The obtained thienostilbenes, products of Wittig reactions **1**–**6**, were subjected to Vilsmeier formylation. The selected thienostilbenes, as a mixture of isomers, were dissolved in 2 mL of DMF and stirred for 10 min at 10 °C. This temperature was achieved using a water bath with a few ice cubes and monitored with a thermometer. The weighed amount of POCl_3_ was slowly added dropwise. After 30 min, the water bath was removed, and the reaction mixture was allowed to stir at room temperature. In the reactions to prepare compounds **7**–**9**, the reaction mixture was stirred at room temperature for 48 h. The reaction mixture for preparing compounds **10** and **11** was heated to 80 °C, and the reaction mixture for preparing compound **12** to 60 °C. Heating was continued for 3 h, after which time the reaction mixture was left to stir at room temperature for 48 h. Upon completion of the reaction, the reaction mixture was neutralized using 10% NaOH solution. When neutralization was achieved, extraction was carried using diethyl ether and water. The combined organic layer was washed with water and dried over MgSO_4_, filtered, and the diethyl ether was evaporated on a rotary evaporator. The dry reaction mixture was purified by column chromatography on silica gel using a PE/E or PE/DCM variable polarity eluent. In the first fractions, the unreacted substrates **1**–**6** were isolated (mostly as *cis*-isomers), while in the last fractions, the desired formyl derivatives **7**–**12** were obtained (mostly as *trans*-isomers). They were further used in the preparation reactions for oximes **13**–**18**. The spectroscopic data for the geometrical isomers were obtained from the ^1^H NMR spectra of the mixtures of compounds **7**–**12**.

 
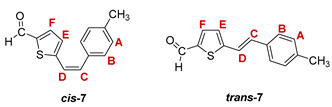


***cis*-5-(4-methylstyryl)thiophene-2-carbaldehyde (*cis*-7)**: 167 mg (12.1%); *R_f_* (petroleum ether/diethyl ether (10%)) = 0.41; ^1^H NMR (CDCl_3_, 600 MHz) *δ*/ppm: 9.79 (s, 1H, -CHO), 7.36 (d, 2H, *J* = 8.1 Hz, H_A/B_), 7.18 (d, 2H, *J* = 8.1 Hz, H_A/B_), 7.18-7.12 (m, 2H, H_E_, H_F_), 6.65 (d, 1H, *J* = 11.7 Hz, H_C/D_), 6.54 (d, 1H, *J* = 11.7 Hz, H_C/D_), 2.36 (s, 3H, -CH_3_);

***trans*-5-(4-methylstyryl)thiophene-2-carbaldehyde (*trans*-7)**: 335 mg (24.2%); *R_f_* (petroleum ether/diethyl ether (10%)) = 0.27; ^1^H NMR (CDCl_3_, 600 MHz) *δ*/ppm: ^1^H NMR (CDCl_3_, 600 MHz) *δ*/ppm: 9.85 (s, 1H, -CHO), 7.66 (d, 1H, *J* = 3.8 Hz, H_E/F_), 7.40 (d, 2H, *J* = 8.9 Hz, H_A/B_), 7.14 (d, 1H, *J* = 16.3 Hz, H_C/D_), 7.15 (d, 2H, *J* = 8.9 Hz, H_A/B_), 7.13 (d, 1H, *J* = 3.8 Hz, H_E/F_), 6.90 (d, 1H, *J* = 16.3 Hz, H_C/D_), 2.37 (s, 3H, -CH_3_);

MS (ESI) *m/z* (%, fragment): 229 (100, M+H^+^); HRMS (*m/z*) for C_14_H_12_OS (obtained for the pure mixture of geometrical isomers): [M+H]^+^_calcd_ = 229.0609, [M+H]^+^_measured_ = 229.0608.

 
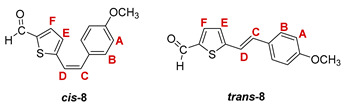


***cis*-5-(4-methoxystyryl)thiophene-2-carbaldehyde (*cis*-8)**: 66 mg (4.8%); *R_f_* (petroleum ether/diethyl ether (10%)) = 0.20; ^1^H NMR (CDCl_3_, 600 MHz) *δ*/ppm: 9.79 (s, 1H, -CHO), 7.55 (d, 1H, *J* = 3.9 Hz, H_E/F_), 7.45 (d, 2H, *J* = 8.8 Hz, H_A/B_), 7.04 (d, 1H, *J* = 3.9 Hz, H_E/F_), 6.87 (d, 2H, *J* = 8.8 Hz, H_A/B_), 6.75 (d, 1H, *J* = 11.9 Hz, H_C/D_), 6.61 (d, 1H, *J* = 11.9 Hz, H_C/D_), 3.82 (s, 3H, -OCH_3_);

***trans*-5-(4-methoxystyryl)thiophene-2-carbaldehyde (*trans*-8) [32]**: 261 mg (19.1%); *R_f_* (petroleum ether/diethyl ether (10%)) = 0.15; ^1^H NMR (CDCl_3_, 600 MHz) *δ*/ppm: 9.83 (s, 1H, -CHO), 7.65 (d, 1H, *J* = 3.9 Hz, H_E/F_), 7.45 (d, 2H, *J* = 8.8 Hz, H_A/B_), 7.11 (d, 1H, *J* = 16.1 Hz, H_C/D_), 7.10 (d, 1H, *J* = 3.9 Hz, H_E/F_), 7.07 (d, 1H, *J* = 16.1 Hz, H_C/D_), 6.91 (d, 2H, *J* = 8.8 Hz, H_A/B_), 3.84 (s, 3H, -OCH_3_);

MS (ESI) *m/z* (%, fragment): 245 (100, M+H^+^), 121 (40); HRMS (*m/z*) for C_14_H_12_O_2_S (obtained for the pure mixture of geometrical isomers): [M+H]^+^_calcd_ = 245.0458, [M+H]^+^_measured_ = 245.0456.

 
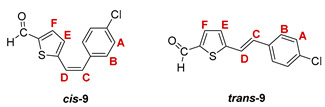


***cis*-5-(4-chlorostyryl)thiophene-2-carbaldehyde (*cis*-9)**: 74 mg (5.4%); *R_f_* (petroleum ether/diethyl ether (30%)) = 0.56; ^1^H NMR (CDCl_3_, 600 MHz) *δ*/ppm: 9.80 (s, 1H, -CHO), 7.56 (d, 1H, *J* = 3.9 Hz, H_E/F_), 7.34 (d, 2H, *J* = 8.2 Hz, H_A/B_), 7.26 (d, 2H, *J* = 8.2 Hz, H_A/B_), 7.02 (d, 1H, *J* = 3.9 Hz, H_E/F_), 6.74 (d, 1H, *J* = 12.1 Hz, H_C/D_), 6.70 (d, 1H, *J* = 12.1 Hz, H_C/D_);

***trans*-5-(4-chlorostyryl)thiophene-2-carbaldehyde (*trans*-9) [32]**: 370 mg (26.9%); *R_f_* (petroleum ether/diethyl ether (30%)) = 0.44; ^1^H NMR (CDCl_3_, 600 MHz) *δ*/ppm: 9.87 (s, 1H, -CHO), 7.67 (d, 1H, *J* = 3.9 Hz, H_E/F_), 7.43 (d, 2H, *J* = 8.4 Hz, H_A/B_), 7.35 (d, 2H, *J* = 8.4 Hz, H_A/B_), 7.19 (d, 1H, *J* = 16.1 Hz, H_C/D_), 7.16 (d, 1H, *J* = 3.9 Hz, H_E/F_), 7.09 (d, 1H, *J* = 16.1 Hz, H_C/D_);

MS (ESI) *m/z* (%, fragment): 249/251 (100, M+H^+^), 121 (30); HRMS (*m/z*) for C_13_H_9_ClOS (obtained for the pure mixture of geometrical isomers): [M+H]^+^_calcd_ = 249.0061, [M+H]^+^_measured_ = 249.0063.

 
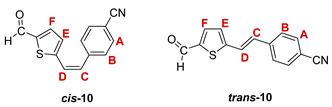


***cis*-4-(2-(5-formylthiophen-2-yl)vinyl)benzonitrile (*cis*-10)**: obtained only in traces; *R_f_* (petroleum ether/DCM (50%)) = 0,17;

***trans*-4-(2-(5-formylthiophen-2-yl)vinyl)benzonitrile (*trans*-10)**: 472 mg (29.4%); *R_f_* (petroleum ether/DCM (50%)) = 0.16; ^1^H NMR (CDCl_3_, 600 MHz) *δ*/ppm: 9.90 (s, 1H, -CHO), 7.70 (d, 1H, *J* = 3.9 Hz, H_E/F_), 7.67 (d, 2H, *J* = 8.3 Hz, H_A/B_), 7.59 (d, 2H, *J* = 8.3 Hz, H_A/B_), 7.32 (d, 1H, *J* = 16.1 Hz, H_C/D_), 7.23 (d, 1H, *J* = 3.9 Hz, H_E/F_), 7.12 (d, 1H, *J* = 16,1 Hz, H_C/D_);

MS (ESI) *m/z* (%, fragment): 240 (100, M+H^+^); HRMS (*m/z*) for C_14_H_9_NOS (obtained for the pure mixture of geometrical isomers): [M+H]^+^_calcd_ = 240.1331, [M+H]^+^_measured_ = 240.1335.

 
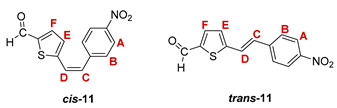


***cis*-5-(4-nitrostyryl)thiophene-2-carbaldehyde (*cis*-11)**: 122 mg (9.0%); *R_f_* (petroleum ether/diethyl ether (50%)) = 0.25; ^1^H NMR (CDCl_3_, 600 MHz) *δ*/ppm: 9.82 (s, 1H, -CHO), 8.23 (d, 2H, *J* = 8.7 Hz, H_A/B_), 7.60 (d, 2H, *J* = 8.7 Hz, H_A/B_), 7.57 (d, 1H, *J* = 3.9 Hz, H_E/F_), 7.03 (d, 1H, *J* = 3.9 Hz, H_E/F_), 6.85 (d, 1H, *J* = 12.2 Hz, H_C/D_), 6.80 (d, 1H, *J* = 12.2 Hz, H_C/D_);

***trans*-5-(4-nitrostyryl)thiophene-2-carbaldehyde (*trans*-11) [33]**: 244 mg (18.0%); *R_f_* (petroleter/diethyl ether (50%)) = 0.20; ^1^H NMR (CDCl_3_, 600 MHz) *δ*/ppm: 9.91 (s, 1H, -CHO), 8.25 (d, 2H, *J* = 8.6 Hz, H_A/B_), 7.71 (d, 1H, *J* = 4.0 Hz, H_E/F_), 7.65 (d, 2H, *J* = 8.6 Hz, H_A/B_), 7.37 (d, 1H, *J* = 16.2 Hz, H_C/D_), 7.26 (d, 1H, *J* = 4.0 Hz, H_E/F_), 7.18 (d, 1H, *J* = 16.2 Hz, H_C/D_);

MS (ESI) *m/z* (%, fragment): 260 (100, M+H^+^); HRMS (*m/z*) for C_13_H_9_NO_3_S (obtained for the pure mixture of geometrical isomers): [M+H]^+^_calcd_ = 260.0303, [M+H]^+^_measured_ = 260.0306.

 
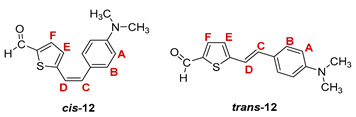


***cis*-5-(4-(dimethylamino)styryl)thiophene-2-carbaldehyde (*cis*-12)**: 247 mg (19.1%); *R_f_* (DCM) = 0.51; ^1^H NMR (CDCl_3_, 600 MHz) *δ*/ppm: 9.63 (s, 1H, -CHO), 7.02 (d, 1H, *J* = 3.4 Hz, H_E/F_), 6.70 (d, 1H, *J* = 3.4 Hz, H_E/F_), 6.68 (d, 1H, *J* = 11.8 Hz, H_C/D_), 6.66 (d, 2H, *J* = 8.7 Hz, H_A/B_), 6.55 (d, 2H, *J* = 8.7 Hz, H_A/B_), 6.49 (d, 1H, *J* = 11.8 Hz, H_C/D_), 2.98 (s, 6H, -N(CH_3_)_2_);

***trans*-5-(4-(dimethylamino)styryl)thiophene-2-carbaldehyde (*trans*-12)** [34]: 227 mg (27.5%); *R_f_* (DCM) = 0.44; ^1^H NMR (CDCl_3_, 600 MHz) *δ*/ppm: 9.80 (s, 1H, -CHO), 7.56 (d, 1H, *J* = 4.0 Hz, H_E/F_), 7.25 (d, 2H, *J* = 8.7 Hz, H_A/B_), 7.23 (d, 2H, *J* = 8.7 Hz, H_A/B_), 7.10 (d, 1H, *J* = 16.5 Hz, H_C/D_), 7.09 (d, 1H, *J* = 4.0 Hz, H_E/F_), 7.00 (d, 1H, *J* = 16.5 Hz, H_C/D_), 2.99 (s, 6H, -N(CH_3_)_2_);

MS (ESI) *m/z* (%, fragment): 258 (100, M+H^+^); HRMS (*m/z*) for C_15_H_15_NOS (obtained for the pure mixture of geometrical isomers): [M+H]^+^_calcd_ = 258.0874, [M+H]^+^_measured_ = 258.0879.

### 3.4. Synthesis of Oximes ***13**–**18***

The obtained mixture of *cis*- and *trans*-isomers of aldehydes, products of Vilsmeier formylations **7**–**12**, were converted into corresponding oximes according to the literature [25]. Crystals of NH_2_OH × HCl were dissolved in a prepared mixture of 10 mL of EtOH and 3 mL of distilled water. After a homogeneous solution was obtained, the corresponding prepared heterostilbene aldehyde derivative **7**–**12** was added. The reaction mixture was stirred at room temperature for 24 h. When the reaction was completed, the solvent was evaporated on a rotavapor under reduced pressure. The reaction mixture was purified by repeated column chromatography on silica gel using PE/DCM and DCM/methanol variable polarity eluents. The desired oxime derivatives **13**–**18** as pure geometrical isomers were thus isolated. The spectroscopic data and yields of pure isolated isomers of compounds **13**–**18** are given below.

**5-(4-methylstyryl)thiophene-2-carbaldehyde oxime.** Yield 465 mg (87%); according to ^1^H NMR spectroscopy the ratio of isomers in the mixture is *cis,syn-*: *trans,syn-*: *trans,anti-* = 1:3:15;

 
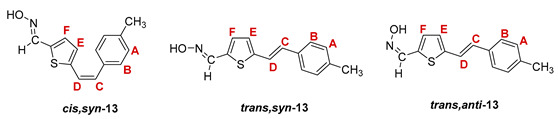


***cis,syn*-5-(4-methylstyryl)thiophene-2-carbaldehyde oxime (*cis,syn*-13)**: 5 mg; *R_f_* (petroleum ether/diethyl ether (30%)) = 0.33; ^1^H NMR (CDCl_3_, 600 MHz) *δ*/ppm: 8.14 (s, 1H, -CH = NOH), 7.25 (d, 2H, *J* = 7.6 Hz, H_A/B_), 7.15 (d, 2H, *J* = 7.6 Hz, H_A/B_), 6.98 (d, 1H, *J* = 3.6 Hz, H_E/F_), 6.89 (d, 1H, *J* = 3.6 Hz, H_E/F_), 6.61 (d, 1H, *J* = 12.3 Hz, H_C/D_), 6.58 (d, 1H, *J* = 12.3 Hz, H_C/D_), 2.17 (s, 3H, -CH_3_);

***trans,syn*-5-(4-methylstyryl)thiophene-2-carbaldehyde oxime (*trans,syn*-13)**: 55 mg; *R_f_* (petroleum ether/diethyl ether (30%)) = 0.34; ^1^H NMR (CDCl_3_, 600 MHz) *δ*/ppm: 8.22 (s, 1H, -CH = NOH), 7.47 (bs, 1H, = N-OH), 7.36 (d, 2H, *J* = 7.9 Hz, H_A/B_), 7.16 (d, 2H, *J* = 7.9 Hz, H_A/B_), 7.12 (d, 1H, *J* = 15.9 Hz, H_C/D_), 7.08 (d, 1H, *J* = 3.5 Hz, H_E/F_), 6.96 (d, 1H, *J* = 3.5 Hz, H_E/F_), 6.94 (d, 1H, *J* = 15.9 Hz, H_C/D_), 2.36 (s, 3H, -CH_3_); ^13^C NMR (CDCl_3_, 150 MHz) *δ*/ppm: 145.2 (d, -CH = NOH), 138.1 (s), 133.8 (s), 133.6 (s), 130.5 (d, C_E/F_), 130.2 (d, C_C/D_), 129.5 (2d, C_A/B_), 126.5 (s), 126.4 (2d, C_A/B_), 125.9 (d, C_E/F_), 120.4 (d, C_C/D_), 21.4 (q, -CH_3_);

***trans,anti*-5-(4-methylstyryl)thiophene-2-carbaldehyde oxime (*trans,anti*-13)**: 67 mg; *R_f_* (petroleum ether/diethyl ether (30%)) = 0.20; ^1^H NMR (CDCl_3_, 600 MHz) *δ*/ppm: 7.64 (s, 1H, -CH = NOH), 7.38 (d, 2H, *J* = 8.1 Hz, H_A/B_), 7.17 (d, 1H, *J* = 16.0 Hz, H_C/D_), 7.17 (d, 2H, *J* = 8.1 Hz, H_A/B_), 7.16 (d, 1H, *J* = 3.5 Hz, H_E/F_), 7.04 (d, 1H, *J* = 16.0 Hz, H_C/D_), 7.03 (d, 1H, *J* = 3.5 Hz, H_E/F_), 2.36 (s, 3H, -CH_3_); ^13^C NMR (CDCl_3_, 150 MHz) *δ*/ppm: 141.8 (d, -CH = NOH), 138.1 (s), 133.8 (s), 132.6 (d), 130.2 (d, C_C/D_), 129.5 (2d, C_A/B_), 128.9 (s), 126.5 (2d, C_A/B_), 126.5 (s), 124.8 (s), 120.4 (d, C_C/D_), 21.4 (q, -CH_3_);

MS (ESI) *m/z* (%, fragment): 244 (100, M+H^+^); HRMS (*m/z*) for C_14_H_13_NOS (obtained for the pure mixture of geometrical isomers): [M+H]^+^_calcd_ = 244.0718, [M+H]^+^_measured_ = 244.0716.

**5-(4-methoxystyryl)thiophene-2-carbaldehyde oxime.** Yield 288 mg (83%); according to ^1^H NMR spectroscopy the ratio of isomers in the mixture is *cis,syn-*:*trans,syn-*:*trans,anti-* = 1:6:20;

 
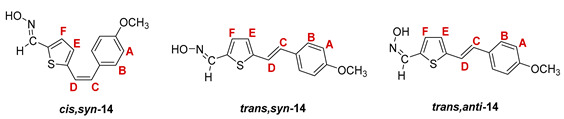


***cis,syn*-5-(4-methoxystyryl)thiophene-2-carbaldehyde oxime (*cis,syn*-14)**: 5 mg; *R_f_* (DCM) = 0.38; ^1^H NMR (CDCl_3_, 600 MHz) *δ*/ppm: 8.15 (s, 1H, -CH = NOH), 7.29 (d, 2H, *J* = 8.6 Hz, H_A/B_), 7.10 (bs, 1H, -CH = N-OH), 6.98 (d, 1H, *J* = 3,3 Hz, H_E/F_), 6.90 (d, 1H, *J* = 3,3 Hz, H_E/F_), 6.88 (d, 2H, *J* = 8.6 Hz, H_A/B_), 6.58 (d, 1H, *J* = 12.0 Hz, H_C/D_), 6.55 (d, 1H, *J* = 12.0 Hz, H_C/D_), 3.83 (s, 3H, -OCH_3_); ^13^C NMR (CDCl_3_, 150 MHz) *δ*/ppm: 144.7 (d, -CH = NOH), 134.0 (s), 132.7 (s), 129.5 (2d), 128.8 (d), 128.7 (s), 128.5 (s), 127.3 (2d), 124.9 (d), 118.8 (d), 113.7 (d), 54.7 (q);

***trans,syn*-5-(4-methoxystyryl)thiophene-2-carbaldehyde oxime (*trans,syn*-14)**: 68 mg; *R_f_* (DCM) = 0.38; ^1^H NMR (CDCl_3_, 600 MHz) *δ*/ppm: 8.22 (s, 1H, -CH = NOH), 7.54 (bs, 1H, = N-OH), 7.41 (d, 2H, *J* = 8.6 Hz, H_A/B_), 7.07 (d, 1H, *J* = 3.8 Hz, H_E/F_), 7.04 (d, 1H, *J* = 16.1 Hz, H_C/D_), 6.94 (d, 1H, *J* = 3.8 Hz, H_E/F_), 6.93 (d, 1H, *J* = 16.1 Hz, H_C/D_), 6.89 (d, 2H, *J* = 8.6 Hz, H_A/B_), 3.83 (s, 3H, -OCH_3_); ^13^C NMR (CDCl_3_, 150 MHz) *δ*/ppm: 145.2 (d, -CH = NOH), 133.3 (s), 130.7 (s), 130.4 (d, C_E/F_), 129.9 (d, C_C/D_), 129.4 (s), 127.8 (2d, C_A/B_), 125.4 (d, C_E/F_), 122.0 (s), 119.3 (d, C_C/D_), 114.3 (2d, C_A/B_), 55.2 (q, -OCH_3_);

***trans,anti*-5-(4-methoxystyryl)thiophene-2-carbaldehyde oxime (*trans,anti*-14)**: 93 mg; *R_f_* (DCM) = 0.19; ^1^H NMR (CDCl_3_, 600 MHz) *δ*/ppm: 7.64 (s, 1H, -CH = NOH), 7.42 (d, 2H, *J* = 8.6 Hz, H_A/B_), 7.26 (d, 1H, *J* = 4.0 Hz, H_E/F_), 7.09 (d, 1H, *J* = 16.2 Hz, H_C/D_), 7.02 (d, 1H, *J* = 16.0 Hz, H_C/D_), 7.01 (d, 1H, *J* = 4.0 Hz, H_E/F_), 6.90 (d, 2H, *J* = 8.6 Hz, H_A/B_), 4.62 (bs, 1H, -CH = NOH), 3.83 (s, 3H, -OCH_3_); ^13^C NMR (CDCl_3_, 150 MHz) *δ*/ppm: 141.2 (d, -CH = NOH), 133.3 (s), 132.6 (s), 132.1 (d, C_E/F_), 129.4 (d, C_C/D_), 128.9 (s), 128.7 (s), 127.4 (2d, C_A/B_), 124.0 (d, C_E/F_), 118.8 (d, C_C/D_), 113.8 (2d, C_A/B_), 54.9 (q, -OCH_3_);

MS (ESI) *m/z* (%, fragment): 260 (100, M+H^+^); HRMS (*m/z*) for C_14_H_13_NO_2_S (obtained for the pure mixture of geometrical isomers): [M+H]^+^_calcd_ = 260.0667, [M+H]^+^_measured_ = 260.0666.

**5-(4-chlorostyryl)thiophene-2-carbaldehyde oxime**. Yield 363 mg (77%); according to ^1^H NMR spectroscopy the ratio of isomers in the mixture is *cis,syn-*:*trans,syn-*:*trans,anti-* = 1:5:13;

 
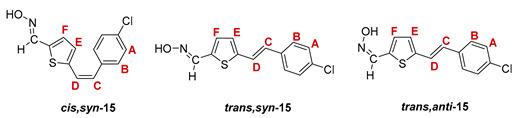


***cis,syn*-5-(4-chlorostyryl)thiophene-2-carbaldehyde oxime (*cis,syn*-15)**: 9 mg; *R_f_* (DCM/methanol (1%)) = 0.58; ^1^H NMR (CDCl_3_, 600 MHz) *δ*/ppm (data from the mixture with *trans,syn*-**15**): 8.15 (s, 1H, -CH = NOH), 7.32 (d, 2H, *J* = 8.5 Hz, H_A/B_), 7.29 (d, 2H, *J* = 8.5 Hz, H_A/B_), 7.14 (d, 1H, *J* = 3.4 Hz, H_E/F_), 6.89 (d, 1H, *J* = 3.4 Hz, H_E/F_), 6.64 (d, 1H, *J* = 12.1 Hz, H_C/D_), 6.56 (d, 1H, *J* = 12.1 Hz, H_C/D_);

***trans,syn*-5-(4-chlorostyryl)thiophene-2-carbaldehyde oxime (*trans,syn*-15)**: 56 mg; *R_f_* (DCM/methanol (1%)) = 0.50; ^1^H NMR (CDCl_3_, 600 MHz) *δ*/ppm: 8.22 (s, 1H, -CH = NOH), 7.39 (d, 2H, *J* = 8.5 Hz, H_A/B_), 7.31 (d, 2H, *J* = 8.5 Hz, H_A/B_), 7.14 (bs, 1H, = N-OH), 7.14 (d, 1H, *J* = 16.2 Hz, H_C/D_), 7.09 (d, 1H, *J* = 3.7 Hz, H_E/F_), 6.99 (d, 1H, *J* = 3.7 Hz, H_E/F_), 6.91 (d, 1H, *J* = 16.2 Hz, H_C/D_); ^13^C NMR (CDCl_3_, 150 MHz) *δ*/ppm: 145.2 (d, -CH = NOH), 144.4 (s), 135.1 (s), 134.2 (s), 133.6 (s), 130.4 (d, C_E/F_), 129.0 (2d, C_A/B_), 128.7 (d, C_C/D_), 127.6 (2d, C_A/B_), 126.5 (d, C_E/F_), 121.9 (d, C_C/D_);

***trans,anti*-5-(4-chlorostyryl)thiophene-2-carbaldehyde oxime (*trans,anti*-15)**: 94 mg; *R_f_* (DCM/methanol (1%)) = 0.20; ^1^H NMR (CDCl_3_, 600 MHz) *δ*/ppm: 7.66 (s, 1H, -CH = NOH), 7.40 (d, 2H, *J* = 8.5 Hz, H_A/B_), 7.32 (d, 2H, *J* = 8.5 Hz, H_A/B_), 7.27 (d, 1H, *J* = 4.0 Hz, H_E/F_), 7.19 (d, 1H, *J* = 16.1 Hz, H_C/D_), 7.06 (d, 1H, *J* = 4.0 Hz, H_E/F_), 7.00 (d, 1H, *J* = 16.1 Hz, H_C/D_); ^13^C NMR (CDCl_3_, 150 MHz) *δ*/ppm: 141.6 (d, -CH = NOH), 135.1 (s), 133.7 (s), 132.5 (d, C_E/F_), 130.1 (s), 129.0 (2d, C_A/B_), 128.8 (s), 128.7 (d, C_C/D_), 127.7 (2d, C_A/B_), 125.5 (d, C_E/F_), 122.0 (d, C_C/D_);

MS (ESI) *m/z* (%, fragment): 264/266 (100, M+H^+^), 121 (35); HRMS (*m/z*) for C_13_H_10_ClNOS (obtained for the pure mixture of geometrical isomers): [M+H]^+^_calcd_ = 264.0172, [M+H]^+^_measured_ = 264.0171.

**4-(2-(5-((hydroxyimino)methyl)thiophen-2-yl)vinyl)benzonitrile.** Yield 366 mg (73%); according to ^1^H NMR spectroscopy the ratio of isomers in the mixture is *cis,syn-*:*trans,syn-*:*trans,anti-* = 1:30:10;

 
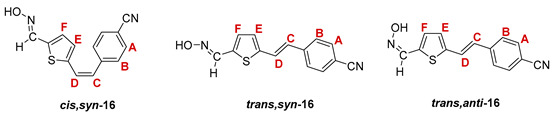


***cis,syn*-4-(2-(5-((hydroxyimino)methyl)thiophen-2-yl)vinyl)benzonitrile (*cis,syn*-16)**: 3 mg; *R_f_* (DCM/methanol (2.5%)) = 0.55; ^1^H NMR (CDCl_3_, 600 MHz) *δ*/ppm (data from the mixture with *trans,syn*-**16**): 8.14 (s, 1H, -CH = NOH), 7.64 (d, 2H, *J* = 8.1 Hz, H_A/B_), 7.47 (d, 2H, *J* = 8.1 Hz, H_A/B_), 7.35 (s, 1H, -CH = NOH), 6.99 (d, 1H, *J* = 3.7 Hz, H_E/F_), 6.89 (d, 1H, *J* = 3.7 Hz, H_E/F_), 6.73 (d, 1H, *J* = 12.1 Hz, H_C/D_), 6.58 (d, 1H, *J* = 12.1 Hz, H_C/D_);

***trans,syn*-4-(2-(5-((hydroxyimino)methyl)thiophen-2-yl)vinyl)benzonitrile (*trans,syn*-16)**: 83 mg; *R_f_* (DCM/methanol (2.5%)) = 0.55; ^1^H NMR (CDCl_3_, 600 MHz) *δ*/ppm: 8.23 (s, 1H, -CH = NOH), 7.62 (d, 2H, *J* = 8.4 Hz, H_A/B_), 7.53 (d, 2H, *J* = 8.4 Hz, H_A/B_), 7.43 (s, 1H, -CH = NOH), 7.27 (d, 1H, *J* = 16.1 Hz, H_C/D_), 7.11 (d, 1H, *J* = 3.6 Hz, H_E/F_), 7.06 (d, 1H, *J* = 3.6 Hz, H_E/F_), 6.93 (d, 1H, *J* = 16.1 Hz, H_C/D_); ^13^C NMR (CDCl_3_, 150 MHz) *δ*/ppm: 144.9 (d, -CH = NOH), 143.5 (s), 141.1 (s), 135.4 (s), 132.6 (2d, C_A/B_), 132.5 (s), 130.4 (d, C_E/F_), 127.9 (d, C_C/D_), 127.8 (d, C_E/F_), 126.8 (2d, C_A/B_), 124.8 (d, C_C/D_), 118.9 (s, -CN);

***trans,anti*-4-(2-(5-((hydroxyimino)methyl)thiophen-2-yl)vinyl)benzonitrile (*trans,anti*-16)**: 28 mg; *R_f_* (DCM/methanol (2.5%)) = 0.27; ^1^H NMR (CDCl_3_, 600 MHz) *δ*/ppm: 7.68 (s, 1H, -CH = NOH), 7.63 (d, 2H, *J* = 8.2 Hz, H_A/B_), 7.55 (d, 2H, *J* = 8.2 Hz, H_A/B_), 7.33 (d, 1H, *J* = 16.2 Hz, H_C/D_), 7.29 (d, 1H, *J* = 4.0 Hz, H_E/F_), 7.13 (d, 1H, *J* = 4.0 Hz, H_E/F_), 7.03 (d, 1H, *J* = 16.2 Hz, H_C/D_); ^13^C NMR (CDCl_3_, 150 MHz) *δ*/ppm: 141.4 (d, -CH = NOH), 141.1 (s), 132.7 (s), 132.6 (2d, C_A/B_), 132.5 (d, C_E/F_), 129.6 (s), 127.8 (d, C_C/D_), 127.2 (s), 126.8 (2d, C_A/B_), 126.7 (d, C_E/F_), 124.9 (d, C_C/D_), 118.9 (s, -CN);

MS (ESI) *m/z* (%, fragment): 255 (100, M+H^+^); HRMS (*m/z*) for C_14_H_10_N_2_OS (obtained for the pure mixture of geometrical isomers): [M+H]^+^_calcd_ = 255.0514, [M+H]^+^_measured_ = 255.0512.

**5-(4-nitrostyryl)thiophene-2-carbaldehyde oxime.** Yield 220 mg (57%); according to ^1^H NMR spectroscopy the ratio of isomers in the mixture is *trans,syn-*:*cis,anti-:trans,anti-* = 8:1:5;

 
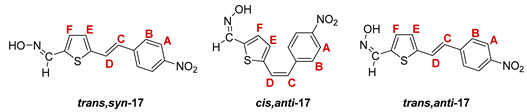


***trans,syn*-5-(4-nitrostyryl)thiophene-2-carbaldehyde oxime (*trans,syn*-17)**: 37 mg; *R_f_* (DCM/methanol (1%)) = 0.27; ^1^H NMR (CDCl_3_, 600 MHz) *δ*/ppm: 8.24 (s, 1H, -CH = NOH), 8.21 (d, 2H, *J* = 8.8 Hz, H_A/B_), 7.59 (d, 2H, *J* = 8.8 Hz, H_A/B_), 7.33 (d, 1H, *J* = 16.2 Hz, H_C/D_), 7.32 (bs, 1H, -CH = NOH), 7.13 (d, 1H, *J* = 3.7 Hz, H_E/F_), 7.10 (d, 1H, *J* = 3.7 Hz, H_E/F_), 6.98 (d, 1H, *J* = 16.2 Hz, H_C/D_); ^13^C NMR (CDCl_3_, 150 MHz) *δ*/ppm: 143.4 (s), 141.5 (s), 133.7 (s), 132.5 (d), 128.2 (d), 127.3 (d), 126.9 (2d), 126.8 (s), 125.8 (d), 124.2 (2d), (1 signal is missing);

***cis,anti*-5-(4-nitrostyryl)thiophene-2-carbaldehyde oxime (*cis,anti*-17)**: 6 mg; *R_f_* (DCM/methanol (1%)) = 0,45; ^1^H NMR (CDCl_3_, 600 MHz) *δ*/ppm: 8.20 (d, 2H, *J* = 8.5 Hz, H_A/B_), 7.58 (s, 1H, -CH = NOH), 7.54 (d, 2H, *J* = 8.5 Hz, H_A/B_), 7.19 (d, 1H, *J* = 4.0 Hz, H_E/F_), 6.94 (d, 1H, *J* = 4.0 Hz, H_E/F_), 6.81 (d, 1H, *J* = 12.1 Hz, H_C/D_), 6.65 (d, 1H, *J* = 12.1 Hz, H_C/D_);

***trans,anti*-5-(4-nitrostyryl)thiophene-2-carbaldehyde oxime (*trans,anti*-17)**: 16 mg; *R_f_* (DCM/methanol (1%)) = 0.43; ^1^H NMR (CDCl_3_, 600 MHz) *δ*/ppm: 8.22 (d, 2H, *J* = 8.6 Hz, H_A/B_), 7.68 (s, 1H, -CH = NOH), 7.60 (d, 2H, *J* = 8.6 Hz, H_A/B_), 7.38 (d, 1H, *J* = 16.2 Hz, H_C/D_), 7.30 (d, 1H, *J* = 3.9 Hz, H_E/F_), 7.16 (d, 1H, *J* = 3.9 Hz, H_E/F_), 7.08 (d, 1H, *J* = 16.2 Hz, H_C/D_); MS (ESI) *m/z* (%, fragment): 275 (100, M+H^+^); HRMS (*m/z*) for C_13_H_10_N_2_O_3_S (obtained for the pure mixture of geometrical isomers): [M+H]^+^_calcd_ = 275.0412, [M+H]^+^_measured_ = 275.0413.

**5-(4-dimethylaminostyryl)thiophene-2-carbaldehyde oxime.** Yield 436 mg (88%); according to ^1^H NMR spectroscopy the ratio of isomers in the mixture is *cis,syn-*:*trans,syn-*:*trans,anti-* = 1:1:1;

 
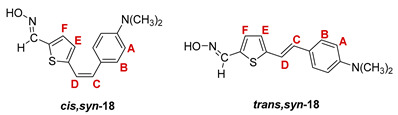


***cys,syn*-5-(4-dimethylaminostyryl)thiophene-2-carbaldehyde oxime (*cys,syn*-18)**: 75 mg; *R_f_* (DCM/methanol (1%)) = 0.21; ^1^H NMR (CDCl_3_, 600 MHz) *δ*/ppm: 8.42 (s, 1H, -CH = NOH), 7.34 (d, 2H, *J* = 8.8 Hz, H_A/B_), 7.31 (broad s, 1H, -CH = NOH), 7.10 (d, 1H, *J* = 3.9 Hz, H_E/F_), 6.99 (d, 2H, *J* = 8.8 Hz, H_A/B_), 6.90 (dd, 1H, *J* = 3.9; 1.9 Hz, H_E/F_), 6.65 (d, 1H, *J* = 11.9 Hz, H_C/D_), 6.48 (d, 1H, *J* = 11.9 Hz, H_C/D_), 2.77 (s, 6H); ^13^C NMR (CDCl_3_, 150 MHz) *δ*/ppm: 149.7 (s), 148.7 (d), 132.9 (s), 130.4 (d), 127.6 (2d), 127.6 (2d), 125.9 (d), 122.4 (d), 117.8 (d), 116.9 (s), 108.8 (s), 44.6 (2q);

***trans,syn*-5-(4-dimethylaminostyryl)thiophene-2-carbaldehyde oxime (*trans,syn*-18)** (it was not possible to isolate this isomer without the traces of *cis,syn*-**18):** *R_f_* (DCM/methanol (1%)) = 0.22; ^1^H NMR (CDCl_3_, 600 MHz) *δ*/ppm: 8.43 (s, 1H, -CH = NOH), 7.79 (broad s, 1H, -CH = NOH), 7.42 (dd, 1H, *J* = 8.5; 2.0 Hz, H_A/B_), 7.17 (d, 1H, *J* = 4.1 Hz, H_E/F_), 7.15 (d, 1H, *J* = 15.6 Hz, H_C/D_), 7.07-6.97 (m, 4H), 6.88 (d, 1H, *J* = 15.6 Hz, H_C/D_), 2.78 (s, 6H);

MS (ESI) *m/z* (%, fragment): 273 (100, M+H^+^); HRMS (*m/z*) for C_15_H_16_N_2_OS (obtained for the pure mixture of geometrical isomers): [M+H]^+^_calcd_ = 273.0553, [M+H]^+^_measured_ = 273.0557.

### 3.5. Chemicals for Enzyme Assays

Recombinant human AChE was kindly donated by Dr Zoran Radić (Skaggs School of Pharmacy and Pharmaceutical Sciences, University of California at San Diego, La Jolla, CA, USA), while purified BChE isolated from human plasma was prepared and generously gifted by Dr Florain Nachon (Institut de Recherche Biomédicale des Armées, Bretigny-sur-Orge, France). Enzymes were diluted in 1% of BSA buffer and stored at 4 °C as work solutions. OP compounds sarin, cyclosarin, VX, and tabun were purchased from the NC Laboratory (Spiez, Switzerland). OP stock solutions (5000 μg/mL) were made in isopropyl alcohol, and further dilutions were made in water just before use. The newly synthesized oximes were prepared in a DMSO solvent at a concentration of 100 mM and stored at 4 °C. Other reagents and solvents used in cholinesterase activity measurement were purchased from Sigma-Aldrich (St. Louis, MO, USA).

### 3.6. Cholinesterase Activity Measurements

Reversible inhibition of AChE and BChE with oximes (50–200 µM) was evaluated in the presence of the substrate concentration range ATCh (0.1–0.7 mM) in 0.1 mM phosphate buffer. Enzyme activity was measured by the Ellman method [35] at 25 °C and 412 nm on a Tecan Infinite M200PRO plate reader (Tecan Austria, GmbH, Salzburg, Austria). Due to the low solubility, a 100 mM stock solution of the tested compounds with the following aliquots was prepared in DMSO, so the same solvent was used in controls as well. The inhibition constants were evaluated as previously described [36].

For the reactivation of OP-inhibited cholinesterases, the enzyme was incubated with 10-fold excess sarin, cyclosarin, VX, or tabun concentrations for about 60 min to achieve a 95−100% inhibition rate. The inhibition mixture was fractionated on a Sephadex G-50 spin column (Roche Diagnostic GmbH, Mannheim, Germany) to remove the unconjugated nerve agent. The reactivation mixture, containing oxime (0.1 mM) in a 0.1 M sodium phosphate buffer pH 7.4, was combined with an inhibited enzyme for the initiation of the reactivation reaction. At specified time intervals, an aliquot was 100-fold diluted in a 0.1 M sodium phosphate buffer pH 7.4 and upon the addition of the substrate ATCh (1 mM) and the reagent DTNB (0.3 mM), residual enzyme activity was measured by the Ellman method [35] at 25 °C and 412 nm on a spectrophotometer (CARY 300, Varian Inc., Mulgrave,Australia). Activity was monitored up to 24 h with consistent correction for the inhibitory effect of oximes and the oxime-induced hydrolysis of ATCh. The first-order reactivation rate constants *k*_obs_ at given oxime concentrations were determined by non-linear regression as described earlier [37].

### 3.7. Docking of Thienostilbene Oximes

The model system for the docking study was prepared using the geometry of the active site of butyrylcholinesterase, taken from the PDB structure 3DJY [38], where the inhibitor tabun was replaced by cyclosarin, as described above. The docking of the oximes into the active site of butyrylcholinesterase (15 residues) was performed using the Autodock program suite [39]. The presented results were obtained using the Lamarckian Genetic Algorithm, with a maximum of 2.5 million energy evaluations for docking calculations. The number of requested genetic algorithm dockings was 10, and 10 binding poses for each ligand were obtained, resulting in different structures of the complex between the cyclosarin-inhibited active site and the potential reactivator. Scanning of PES was carried out using the Gaussian09 program package [40], at the B3LYP/6-31G(d) level of theory.

### 3.8. In Silico Prediction of ADME Properties

*In silico* predictions of ADME properties were performed with ACD/Percepta (ver. 14.2.0; Build 2977; ACD/Laboratories, Toronto, ON, Canada).

## 4. Conclusions

This paper presents the design and synthesis of new, uncharged 2-thienostilbene oximes by an economical three-step synthesis using the Wittig reaction, Vilsmeier formylation, and the transformation of aldehydes into oximes in high yields. The expected targeted products were pure *cis*- and *trans*-isomers of *syn*- and *anti*-oximes containing different substituents bound in the *para*-position of the benzene ring. Finally, in each oxime formation, although four isomers were expected, *cis,syn*, *trans,syn*-, *cis,anti*-, and *trans,anti*-, considering the configuration of the carbon–carbon and carbon–nitrogen double bonds, after successive column and thin layer chromatography of the reaction mixtures, only some of isomers **13**–**18** were successfully isolated. Out of the eight oximes selected for the reactivation of nerve-agent inhibited cholinesterases, *trans,anti*-**15** and *trans,anti*-**14** and their *trans,syn*-counterparts reactivated cyclosarin-inhibited BChE up to 70%. This is the first study to show the potential of thienostilbene oximes as therapeutics in OP poisoning, and it seems that further design of the compounds e.g., with amide, OH, mono- and dimethylamino groups, or a triazole ring, could provide a new platform for further antidote and scavenger development for exposure to organophosphates.

## Figures and Tables

**Table 1:** ADME properties of the oxime derivatives **13**–**18**.

Compound	logP ^a^	Solubility ^b^ [mg/mL]	Permeability ^c^ [10^−6^ cm/s]	PPB ^d^	CNS ^e^
**13**	3.95	0.004	23.6	83%	−1.86
**14**	3.70	0.009	23.7	77%	−1.77
**15**	4.27	0.002	23.3	90%	−2.05
**16**	3.32	0.009	23.4	77%	−1.82
**17**	3.58	0.01	23.6	81%	−1.92
**18**	3.94	0.01	23.6	83%	−1.86

^a^ lipophilicity logP (<−2.0 very hydrophilic, −2.0–(−1.0) hydrophilic, −1.0–4.2 optimal, 4.2–5.0 lipophilic); ^b^ solubility (<0.01 highly insoluble, 0.01–0.10 insoluble, >0.10 soluble); ^c^ Caco permeability (<1.0 × 10^−6^ poorly permeable, 1.0–7.0 × 10^−6^ moderately permeable, >7.0 × 10^−6^ highly permeable); ^d^ PPB—plasma-protein binding (10–40% weakly bound, 40–80% moderately, 80–90% strongly bound, >90% extensively bound); ^e^ CNS < −3.5 non-penetrant, −3.50–(−3.0) weak penetrant, >−3.0 penetrant); (ACD/Percepta (ver. 14.2.0; Build 2977; ACD/Laboratories, Toronto, ON, Canada).

**Table 2:** Dissociation constants, *K*_i_ (μM) ± SE, for reversible inhibition of human acetylcholinesterase (AChE) and butyrylcholinesterase (BChE) with new oximes at 25 °C.

Thienostilbene Oximes	AChE	BChE
*trans,syn*-**13**	46 ± 7	355 ± 26
*trans,anti*-**13**	41 ± 4	322 ± 38
*trans,syn*-**14**	67 ± 12	271 ± 24
*trans,anti*-**14**	54 ± 7	172 ± 14
*trans,syn*-**15**	52 ± 12	573 ± 35
*trans,anti*-**15**	76 ± 21	482 ± 45
*trans,anti*-**16**	158 ± 40	44 ± 9
*cis,syn*-**18**	39 ± 11	89 ± 16

## Data Availability

Data is contained within the article and Supplementary Material.

## Supplementary Materials

The following are available online at https://www.mdpi.com/article/10.3390/ph14111147/s1: Spectra contributions: ^1^H and ^13^C NMR spectra of the new investigated compounds, along with the 2D NMR spectra and mass spectra of all new isolated oxime derivatives **13**–**18**; Figure S1: Superposition of the cyclosarin-bound AChE (3ZLU) and BChE (3DJY) with cyclosarin in the same conformation at the active serine; Figure S2: Superposition of cyclosarin-bound AChE (3ZLU) and BChE (3DJY) with cyclosarin bound at the active serine obtained by replacing the dimethylamino and ethoxy groups of tabun with methyl and cyclohexyloxy groups of cyclosarin, respectively; Figure S3: Energy profile for the incremental decrease of the distance between the oxygen of oxime and phosphorus; Table S1: Data obtained by scanning of PES, presented in Figure S3.
