# Supplementary material for: New Uncharged 2-Thienostilbene Oximes as Reactivators of Organophosphate-Inhibited Cholinesterases
Source: Pharmaceuticals (Basel). 2021 Nov 11;14(11):1147. doi: 10.3390/ph14111147 (PMC8621217; doi:10.3390/ph14111147)
Supplement: Supplementary file 1 [file pharmaceuticals-14-01147-s001.zip › pharmaceuticals-1450270-supplementary.pdf]

pharmaceuticals

MDPI

Supplemental material

New Uncharged 2-Thienostilbene Oximes as Reactivators of Organophosphate-Inhibited Cholinesterases

Milena Mlakić [1,†], Tena Čadež [2,†], Danijela Barić [3], Ivana Puček [1], Ana Ratković [4], Željko Marinić [5], Kornelija Lasić [6], Zrinka Kovarik [2,*] and Irena Škorić [1,*]

1 Department of Organic Chemistry, Faculty of Chemical Engineering and Technology, University of Zagreb, Marulićev Trg 19, HR-10 000 Zagreb, Croatia; mdragojev@fkit.hr (M.M.); ivana.pucek812@gmail.com (I.P.)
2 Institute for Medical Research and Occupational Health, Ksaverska Cesta 2, HR-10 000 Zagreb, Croatia; tcadez@imi.hr
3 Group for Computational Life Sciences, Division of Physical Chemistry, Ruđer Bošković Institute, Bijenička Cesta 54, HR-10 000 Zagreb, Croatia; dbaric@irb.hr
4 Fidelta Ltd., Prilaz Baruna Filipovića 29, HR-10 000 Zagreb, Croatia; ana.ratkovic30@gmail.com
5 NMR Center, Ruđer Bošković Institute, Bijenička Cesta 54, HR-10 000 Zagreb, Croatia; zmarinic@irb.hr
6 Pliva Tapi R&D, TEVA, Prilaz Baruna Filipovića 25, HR-10 000 Zagreb, Croatia; Kornelija.Lasic@pliva.com
* Correspondence: zkovarik@imi.hr (Z.K.); iskoric@fkit.hr (I.Š.)
† These authors contributed equally.

Spectra contributions:

^{1}H NMR spectra (600 MHz, $CDCl_3$) of the mixture of isomers of 2-(4-methylstyryl)thiophene (*cis*-**1** and *trans*-**1**)

A part of the ^{1}H NMR spectra (600 MHz, $CDCl_3$) of the mixture of isomers of 2-(4-methylstyryl)thiophene (*cis*-**1** and *trans*-**1**)

Mass spectra and HRMS analysis of the mixture of isomers of 2-(4-methylstyryl)thiophene (*cis*-**1** and *trans*-**1**)

^{1}H NMR spectra (600 MHz, $CDCl_3$) of the mixture of isomers of 2-(4-methoxystyryl)thiophene (*cis*-**2** and *trans*-**2**)

A part of the ^{1}H NMR spectra (600 MHz, $CDCl_3$) of the mixture of isomers of 2-(4-methoxystyryl)thiophene (*cis*-**2** and *trans*-**2**)

Mass spectra and HRMS analysis of the mixture of isomers of 2-(4-methoxystyryl)thiophene (*cis*-**2** and *trans*-**2**)

^{1}H NMR spectra (600 MHz, $CDCl_3$) of the mixture of isomers of 2-(4-chlorostyryl)thiophene (*cis*-**3** and *trans*-**3**)

A part of the ^{1}H NMR spectra (600 MHz, $CDCl_3$) of the mixture of isomers of 2-(4-chlorostyryl)thiophene (*cis*-**3** and *trans*-**3**)

Mass spectra and HRMS analysis of the mixture of isomers of 2-(4-chlorostyryl)thiophene (*cis*-**3** and *trans*-**3**)

^{1}H NMR spectra (600 MHz, $CDCl_3$) of the mixture of isomers of 2-(4-(2-(thiophen-2-yl)vinyl)benzonitrile (*cis*-**4** and *trans*-**4**)

A part of the ^{1}H NMR spectra (600 MHz, $CDCl_3$) of the mixture of isomers of 2-(4-(2-(thiophen-2-yl)vinyl)benzonitrile (*cis*-**4** and *trans*-**4**)

Mass spectra and HRMS analysis of the mixture of isomers of 2-(4-(2-(thiophen-2-yl)vinyl)benzonitrile (*cis*-**4** and *trans*-**4**)

^{1}H NMR spectra (600 MHz, $CDCl_3$) of the mixture of isomers of 2-(4-nitrostyryl)thiophene (*cis*-**5** and *trans*-**5**)

A part of the ^{1}H NMR spectra (600 MHz, $CDCl_3$) of the mixture of isomers of 2-(4-nitrostyryl)thiophene (*cis*-**5** and *trans*-**5**)

Mass spectra and HRMS analysis of the mixture of isomers of 2-(4-nitrostyryl)thiophene (*cis*-**5** and *trans*-**5**)

^{1}H NMR spectra (600 MHz, $CDCl_3$) of the mixture of isomers of *N*,*N*-dimethyl-4-(2-(thiophen-2-yl)vinyl)aniline (*cis*-**6** and *trans*-**6**)

A part of the ^{1}H NMR spectra (600 MHz, $CDCl_3$) of the mixture of isomers of *N*,*N*-dimethyl-4-(2-(thiophen-2-yl)vinyl)aniline (*cis*-**6** and *trans*-**6**)

Mass spectra and HRMS analysis of the mixture of isomers of *N*,*N*-dimethyl-4-(2-(thiophen-2-yl)vinyl)aniline (*cis*-**6** and *trans*-**6**)

^{1}H NMR spectra (600 MHz, $CDCl_3$) of the mixture of isomers of 5-(4-methylstyryl)thiophene-2-carbaldehyde (*cis*-**7** and *trans*-**7**)

A part of the ^{1}H NMR spectra (600 MHz, $CDCl_3$) of the mixture of isomers of 5-(4-methylstyryl)thiophene-2-carbaldehyde (*cis*-**7** and *trans*-**7**)

Mass spectra and HRMS analysis of the mixture of isomers of 5-(4-methylstyryl)thiophene-2-carbaldehyde (*cis*-**7** and *trans*-**7**)

^{1}H NMR spectra (600 MHz, $CDCl_3$) of the mixture of isomers of 5-(4-methoxystyryl)thiophene-2-carbaldehyde (*cis*-**8** and *trans*-**8**)

A part of the ^{1}H NMR spectra (600 MHz, $CDCl_3$) of the mixture of isomers of 5-(4-methoxystyryl)thiophene-2-carbaldehyde (*cis*-**8** and *trans*-**8**)

Mass spectra and HRMS analysis of the mixture of isomers of 5-(4-methoxystyryl)thiophene-2-carbaldehyde (*cis*-**8** and *trans*-**8**)

^{1}H NMR spectra (600 MHz, $CDCl_3$) of the mixture of isomers of 5-(4-chlorostyryl)thiophene-2-carbaldehyde (*cis*-**9** and *trans*-**9**)

A part of the ^{1}H NMR spectra (600 MHz, $CDCl_3$) of the mixture of isomers of 5-(4-chlorostyryl)thiophene-2-carbaldehyde (*cis*-**9** and *trans*-**9**)

Mass spectra and HRMS analysis of the mixture of isomers of 5-(4-chlorostyryl)thiophene-2-carbaldehyde (*cis*-**9** and *trans*-**9**)

^{1}H NMR spectra (600 MHz, $CDCl_3$) of the mixture of isomers of 4-(2-(5-formylthiophen-2-yl)vinyl)benzonitrile (*cis*-**10** and *trans*-**10**)

A part of the ^{1}H NMR spectra (600 MHz, $CDCl_3$) of the mixture of isomers of 4-(2-(5-formylthiophen-2-yl)vinyl)benzonitrile (*cis*-**10** and *trans*-**10**)

Mass spectra and HRMS analysis of the mixture of isomers of 4-(2-(5-formylthiophen-2-yl)vinyl)benzonitrile (*cis*-**10** and *trans*-**10**)

^{1}H NMR spectra (600 MHz, $CDCl_3$) of the mixture of isomers of 5-(4-nitrostyryl)thiophene-2-carbaldehyde (*cis*-**11** and *trans*-**11**)

A part of the ^{1}H NMR spectra (600 MHz, $CDCl_3$) of the mixture of isomers of 5-(4-nitrostyryl)thiophene-2-carbaldehyde (*cis*-**11** and *trans*-**11**)

Mass spectra and HRMS analysis of the mixture of isomers of 5-(4-nitrostyryl)thiophene-2-carbaldehyde (*cis*-**11** and *trans*-**11**)

^{1}H NMR spectra (600 MHz, $CDCl_3$) of the mixture of isomers of 5-(4-(dimethylamino)styryl)thiophene-2-carbaldehyde (*cis*-**12** and *trans*-**12**)

A part of the ^{1}H NMR spectra (600 MHz, $CDCl_3$) of the mixture of isomers of 5-(4-(dimethylamino)styryl)thiophene-2-carbaldehyde (*cis*-**12** and *trans*-**12**)

Mass spectra and HRMS analysis of the mixture of isomers of 5-(4-(dimethylamino)styryl)thiophene-2-carbaldehyde (*cis*-**12** and *trans*-**12**)

^{1}H NMR spectra (600 MHz, $CDCl_3$) of *trans,anti*-5-(4-methylstyryl)thiophene-2-carbaldehyde oxime (*trans,anti*-**13**)

A part of the ^{1}H NMR spectra (600 MHz, $CDCl_3$) of *trans,anti*-5-(4-methylstyryl)thiophene-2-carbaldehyde oxime (*trans,anti*-**13**)

^{13}C NMR spectrum (150 MHz, $CDCl_3$) of *trans,anti*-5-(4-methylstyryl)thiophene-2-carbaldehyde oxime (*trans,anti*-**13**)

COSY spectrum of *trans,anti*-5-(4-methylstyryl)thiophene-2-carbaldehyde oxime (*trans,anti*-**13**)

HSQC spectrum of *trans,anti*-5-(4-methylstyryl)thiophene-2-carbaldehyde oxime (*trans,anti*-**13**)

^{1}H NMR spectra (600 MHz, $CDCl_3$) of *trans,syn*-5-(4-methylstyryl)thiophene-2-carbaldehyde oxime (*trans,syn*-**13**)

A part of the ^{1}H NMR spectra (600 MHz, $CDCl_3$) of *trans,syn*-5-(4-methylstyryl)thiophene-2-carbaldehyde oxime (*trans,syn*-**13**)

^{13}C NMR spectrum (150 MHz, $CDCl_3$) of *trans,syn*-5-(4-methylstyryl)thiophene-2-carbaldehyde oxime (*trans,syn*-**13**)

COSY spectrum of *trans,syn*-5-(4-methylstyryl)thiophene-2-carbaldehyde oxime (*trans,syn*-**13**)

HSQC spectrum of *trans,syn*-5-(4-methylstyryl)thiophene-2-carbaldehyde oxime (*trans,syn*-**13**)

^{1}H NMR spectra (600 MHz, $CDCl_3$) of *cis,syn*-5-(4-methylstyryl)thiophene-2-carbaldehyde oxime (*cis,syn*-**13**)

A part of the ^{1}H NMR spectra (600 MHz, $CDCl_3$) of *cis,syn*-5-(4-methylstyryl)thiophene-2-carbaldehyde oxime (*cis,syn*-**13**)

Mass spectra and HRMS analysis of the mixture of isomers of 5-(4-methylstyryl)thiophene-2-carbaldehyde oxime (**13**)

^{1}H NMR spectra (600 MHz, $CDCl_3$ + CD_3OD) of *trans,anti*-5-(4-methoxystyryl)thiophene-2-carbaldehyde oxime (*trans,anti*-**14**)

A part of the ^{1}H NMR spectra (600 MHz, $CDCl_3$ + CD_3OD) of *trans,anti*-5-(4-methoxystyryl)thiophene-2-carbaldehyde oxime (*trans,anti*-**14**)

^{13}C NMR spectrum (150 MHz, $CDCl_3$+ CD_3OD) of *trans,anti*-5-(4-methoxystyryl)thiophene-2-carbaldehyde oxime (*trans,anti*-**14**)

COSY spectrum of *trans,anti*-5-(4-methoxystyryl)thiophene-2-carbaldehyde oxime (*trans,anti*-**14**)

HSQC spectrum of *trans,anti*-5-(4-methoxystyryl)thiophene-2-carbaldehyde oxime (*trans,anti*-**14**)

^{1}H NMR spectra (600 MHz, $CDCl_3$) of *cis,syn*-5-(4-methoxystyryl)thiophene-2-carbaldehyde oxime (*cis,syn*-**14**)

A part of the ^{1}H NMR spectra (600 MHz, $CDCl_3$) of *cis,syn*-5-(4-methoxystyryl)thiophene-2-carbaldehyde oxime (*cis,syn*-**14**)

^{13}C NMR spectrum (150 MHz, $CDCl_3$) of *cis,syn*-5-(4-methoxystyryl)thiophene-2-carbaldehyde oxime (*cis,syn*-**14**)

COSY spectrum of *cis,syn*-5-(4-methoxystyryl)thiophene-2-carbaldehyde oxime (*cis,syn*-**14**)

HSQC spectrum of *cis,syn*-5-(4-methoxystyryl)thiophene-2-carbaldehyde oxime (*cis,syn*-**14**)

^{1}H NMR spectra (600 MHz, $CDCl_3$) of *trans,syn*-5-(4-methoxystyryl)thiophene-2-carbaldehyde oxime (*trans,syn*-**14**)

A part of the ^{1}H NMR spectra (600 MHz, $CDCl_3$) of *trans,syn*-5-(4-methoxystyryl)thiophene-2-carbaldehyde oxime (*trans,syn*-**14**)

^{13}C NMR spectrum (150 MHz, $CDCl_3$) of *trans,syn*-5-(4-methoxystyryl)thiophene-2-carbaldehyde oxime (*trans,syn*-**14**)

COSY spectrum of *trans,syn*-5-(4-methoxystyryl)thiophene-2-carbaldehyde oxime (*trans,syn*-**14**)

HSQC spectrum of *trans,syn*-5-(4-methoxystyryl)thiophene-2-carbaldehyde oxime (*trans,syn*-**14**)

Mass spectra and HRMS analysis of the mixture of isomers of 5-(4-methoxystyryl)thiophene-2-carbaldehyde oxime (**14**)

^{1}H NMR spectra (600 MHz, $CDCl_3$) of *trans,syn*-5-(4-chlorostyryl)thiophene-2-carbaldehyde oxime (*trans,syn*-**15**)

A part of the ^{1}H NMR spectra (600 MHz, $CDCl_3$) of *trans,syn*-5-(4-chlorostyryl)thiophene-2-carbaldehyde oxime (*trans,syn*-**15**)

^{13}C NMR spectrum (150 MHz, $CDCl_3$) of *trans,syn*-5-(4-chlorostyryl)thiophene-2-carbaldehyde oxime (*trans,syn*-**15**)

COSY spectrum of *trans,syn*-5-(4-chlorostyryl)thiophene-2-carbaldehyde oxime (*trans,syn*-**15**)

HSQC spectrum of *trans,syn*-5-(4-chlorostyryl)thiophene-2-carbaldehyde oxime (*trans,syn*-**15**)

^{1}H NMR spectra (600 MHz, $CDCl_3$) of *trans,anti*-5-(4-chlorostyryl)thiophene-2-carbaldehyde oxime (*trans,anti*-**15**)

^{13}C NMR spectrum (150 MHz, $CDCl_3$) of *trans,anti*-5-(4-chlorostyryl)thiophene-2-carbaldehyde oxime (*trans,anti*-**15**)

Mass spectra and HRMS analysis of the mixture of isomers of 5-(4-chlorostyryl)thiophene-2-carbaldehyde oxime (**15**)

^{1}H NMR spectra (600 MHz, $CDCl_3$) of *cis,syn*-4-(2-(5-((hydroxyimino)methyl)thiophen-2-yl)vinyl)benzonitrile (*cis,syn*-**16**) in the mixture with *trans,syn*-**16**

A part of the ^{1}H NMR spectra (600 MHz, $CDCl_3$) of *cis,syn*-4-(2-(5-((hydroxyimino)methyl)thiophen-2-yl)vinyl)benzonitrile (*cis,syn*-**16**) in the mixture with *trans,syn*-**16**

^{1}H NMR spectrum (600 MHz, $CDCl_3$) of *trans,syn*-4-(2-(5-((hydroxyimino)methyl)thiophen-2-yl)vinyl)benzonitrile (*trans,syn*-**16**)

A part of the ^{1}H NMR spectrum (600 MHz, $CDCl_3$) of *trans,syn*-4-(2-(5-((hydroxyimino)methyl)thiophen-2-yl)vinyl)benzonitrile (*trans,syn*-**16**)

^{13}C NMR spectrum (150 MHz, $CDCl_3$) of *trans,syn*-4-(2-(5-((hydroxyimino)methyl)thiophen-2-yl)vinyl)benzonitrile (*trans,syn*-**16**)

^{1}H NMR spectrum (600 MHz, $CDCl_3$) of *cis,anti*-4-(2-(5-((hydroxyimino)methyl)thiophen-2-yl)vinyl)benzonitrile (*cis,anti*-**16**) with traces of other isomers

A part of the ^{1}H NMR spectrum (600 MHz, $CDCl_3$) of *cis,anti*-4-(2-(5-((hydroxyimino)methyl)thiophen-2-yl)vinyl)benzonitrile (*cis,anti*-**16**) with traces of other isomers

^{1}H NMR spectrum (600 MHz, $CDCl_3$ + CD_3OD) of *trans,anti*-4-(2-(5-((hydroxyimino)methyl)thiophen-2-yl)vinyl)benzonitrile (*trans,anti*-**16**)

A part of the ^{1}H NMR spectrum (600 MHz, $CDCl_3$ + CD_3OD) of *trans,anti*-4-(2-(5-((hydroxyimino)methyl)thiophen-2-yl)vinyl)benzonitrile (*trans,anti*-**16**)

^{13}C NMR spectrum (150 MHz, $CDCl_3$+ CD_3OD) of *trans,anti*-4-(2-(5-((hydroxyimino)methyl)thiophen-2-yl)vinyl)benzonitrile (*trans,anti*-**16**)

COSY spectrum of *trans,anti*-4-(2-(5-((hydroxyimino)methyl)thiophen-2-yl)vinyl)benzonitrile (*trans,anti*-**16**)

HSQC spectrum of *trans,anti*-4-(2-(5-((hydroxyimino)methyl)thiophen-2-yl)vinyl)benzonitrile (*trans,anti*-**16**)

Mass spectra and HRMS analysis of the mixture of isomers of 4-(2-(5-((hydroxyimino)methyl)thiophen-2-yl)vinyl)benzonitrile (**16**)

^{1}H NMR spectrum (600 MHz, $CDCl_3$) of *trans,syn*-5-(4-nitrostyryl)thiophene-2-carbaldehyde oxime (*trans,syn*-**17**)

A part of the ^{1}H NMR spectrum (600 MHz, $CDCl_3$) of *trans,syn*-5-(4-nitrostyryl)thiophene-2-carbaldehyde oxime (*trans,syn*-**17**)

^{13}C NMR spectrum (150 MHz, $CDCl_3$) of *trans,syn*-5-(4-nitrostyryl)thiophene-2-carbaldehyde oxime (*trans,syn*-**17**)

COSY spectrum of *trans,syn*-5-(4-nitrostyryl)thiophene-2-carbaldehyde oxime (*trans,syn*-**17**)

^{1}H NMR spectrum (600 MHz, $CDCl_3$) of *trans,anti*-5-(4-nitrostyryl)thiophene-2-carbaldehyde oxime (*trans,anti*-**17**) with traces of *cis,anti*-**17**

A part of the ^{1}H NMR spectrum (600 MHz, $CDCl_3$) of *trans,anti*-5-(4-nitrostyryl)thiophene-2-carbaldehyde oxime (*trans,anti*-**17**) with traces of *cis,anti*-**17**

Mass spectra and HRMS analysis of the mixture of isomers of 5-(4-nitrostyryl)thiophene-2-carbaldehyde oxime (**17**)

^{1}H NMR spectrum (600 MHz, $CDCl_3$) of *cis,syn*-5-(4-dimethylaminostyryl)thiophene-2-carbaldehyde oxime (*cis,syn*-**18**)

A part of the ^{1}H NMR spectra (600 MHz, $CDCl_3$) of *cis,syn*-5-(4-dimethylaminostyryl)thiophene-2-carbaldehyde oxime (*cis,syn*-**18**)

^{13}C NMR spectrum (150 MHz, $CDCl_3$) of *cis,syn*-5-(4-dimethylaminostyryl)thiophene-2-carbaldehyde oxime (*cis,syn*-**18**)

COSY spectrum of *cis,syn*-5-(4-dimethylaminostyryl)thiophene-2-carbaldehyde oxime (*cis,syn*-**18**)

HSQC spectrum of *cis,syn*-5-(4-dimethylaminostyryl)thiophene-2-carbaldehyde oxime (*cis,syn*-**18**)

^{1}H NMR spectrum (600 MHz, $CDCl_3$) of *trans,syn*-5-(4-dimethylaminostyryl)thiophene-2-carbaldehyde oxime (*trans,syn*-**18**) with traces of *cis,syn*-**18**

A part of the ^{1}H NMR spectrum (600 MHz, $CDCl_3$) of *trans,syn*-5-(4-dimethylaminostyryl)thiophene-2-carbaldehyde oxime (*trans,syn*-**18**) with traces of *cis,syn*-**18**

Mass spectra and HRMS analysis of the mixture of isomers of 5-(4-dimethylaminostyryl)thiophene-2-carbaldehyde oxime (**18**)

Figure S1: Superposition of the cyclosarin-bound AChE (3ZLU) and BChE (3DJY) with cyclosarin in the same conformation at the active serine

Figure S2: Superposition of cyclosarin-bound AChE (3ZLU) and BChE (3DJY) with cyclosarin bound at the active serine obtained by replacing the dimethylamino and ethoxy groups of tabun with methyl and cyclohexyloxy groups of cyclosarin, respectively

Figure S3: Energy profile for the incremental decrease of the distance between the oxygen of oxime and phosphorus

Table S1: Data obtained by scanning of PES, presented in Figure S3.

^{1}H NMR spectrum (600 MHz, $CDCl_3$) of the mixture of geometrical isomers of 2-(4-methylstyryl)thiophene (*cis*-1 and *trans*-1)

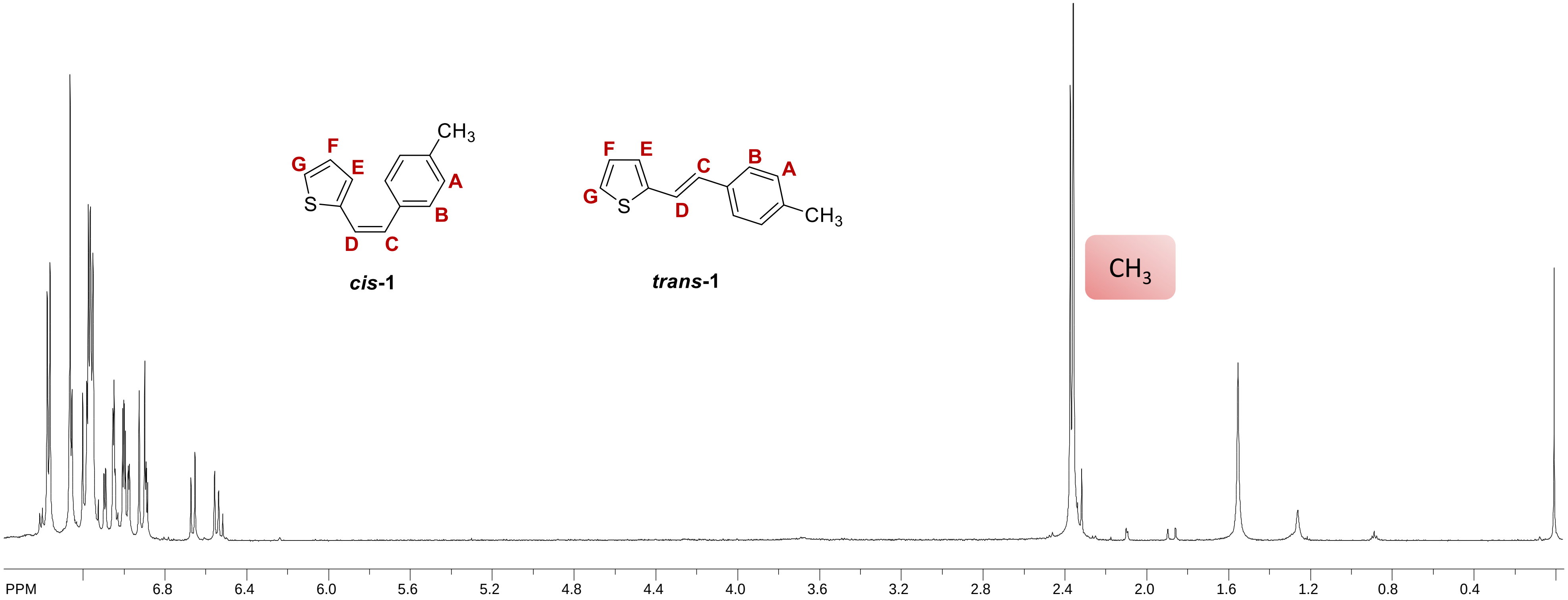

file: D:\PODACI\00 IVANA PUCEK\IvanaPucek diplomski rad\NMR\IP-45f\fid expt: <zg30>
transmitter freq.: 600.135401 MHz
time domain size: 32768 points
width: 12019.23 Hz = 20.027532 ppm = 0.366798 Hz/pt
number of scans: 21

freq. of 0 ppm: 600.130006 MHz
processed size: 32768 complex points
LB: 0.000 GB: 0.0000

A part of the ^{1}H NMR spectrum (600 MHz, $CDCl_3$) of the mixture of geometrical isomers of 2-(4-methylstyryl)thiophene (*cis*-1 and *trans*-1)

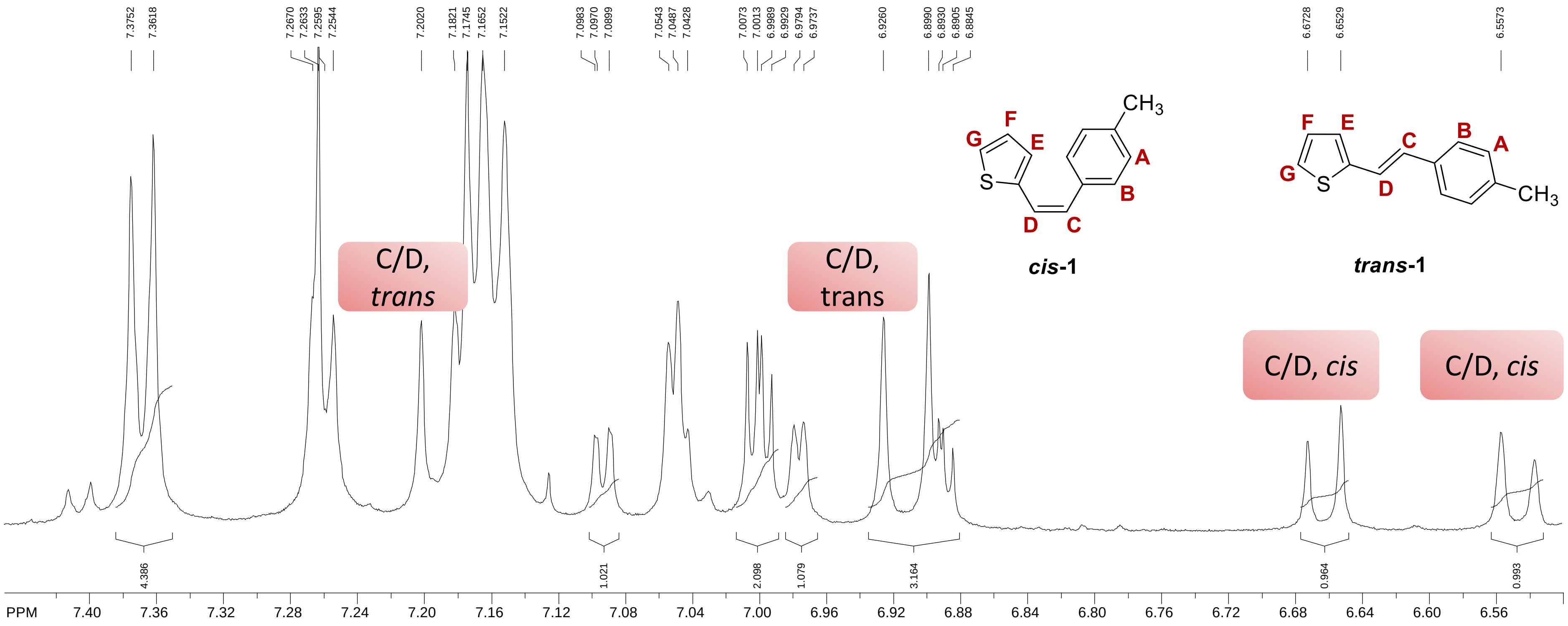

file: D:\PODACI\00 IVANA PUCEK\IvanaPucek diplomski rad\NMR\IP-45f\fid expt: <zg30>
transmitter freq.: 600.135401 MHz
time domain size: 32768 points
width: 12019.23 Hz = 20.027532 ppm = 0.366798 Hz/pt
number of scans: 21

freq. of 0 ppm: 600.130006 MHz
processed size: 32768 complex points
LB: 0.000 GB: 0.0000

Mass spectra and HRMS analysis of the mixture of geometrical isomers of 2-(4-methylstyryl)thiophene (*cis*-1 and *trans*-1)

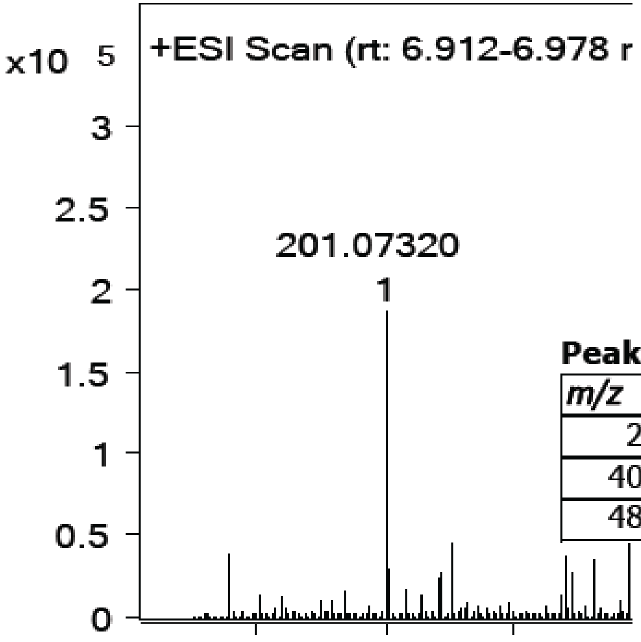

Peak List

m/z	z	Abund	Formula	Ion
201.0732	1	186840.83	C13 H12 S	(M+H)+
408.36757	1	93526.87		
488.32468	1	165932.14		

Formula Calculator Results

Formula	Best	Mass	Tgt Mass	Diff (ppm)	Ion Species	Score
C13 H12 S	True	200.06596	200.06597	0.06	C13 H13 S	99.68

^{1}H NMR spectrum (600 MHz, $CDCl_3$) of the mixture of geometrical isomers of 2-(4-methoxystyryl)thiophene (*cis*-2 and *trans*-2)

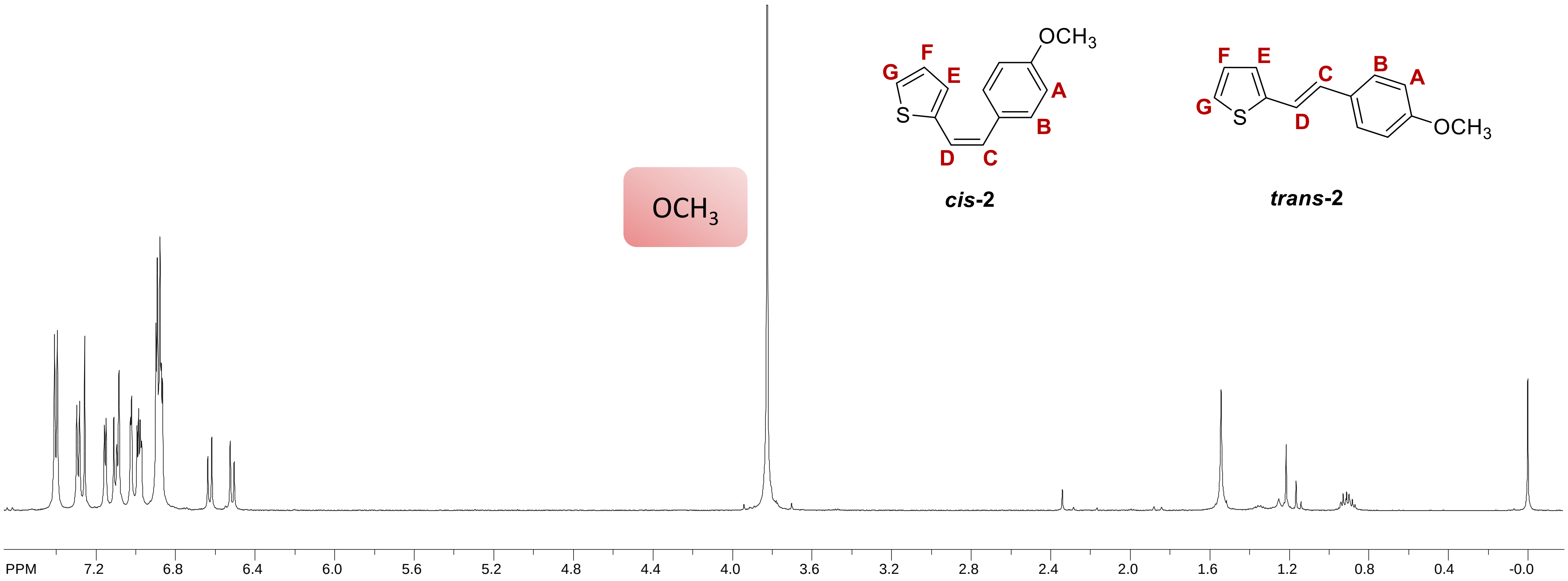

file: D:\PODACI\00 IVANA PUCEK\IvanaPucek diplomski rad\NMR\IP-46F\fid expt: <zg30>
transmitter freq.: 600.135401 MHz
time domain size: 32768 points
width: 12019.23 Hz = 20.027532 ppm = 0.366798 Hz/pt
number of scans: 31

freq. of 0 ppm: 600.130011 MHz
processed size: 32768 complex points
LB: 0.000 GB: 0.0000

A part of the ^{1}H NMR spectrum (600 MHz, $CDCl_3$) of the mixture of geometrical isomers of 2-(4-methoxystyryl)thiophene (*cis*-2 and *trans*-2)

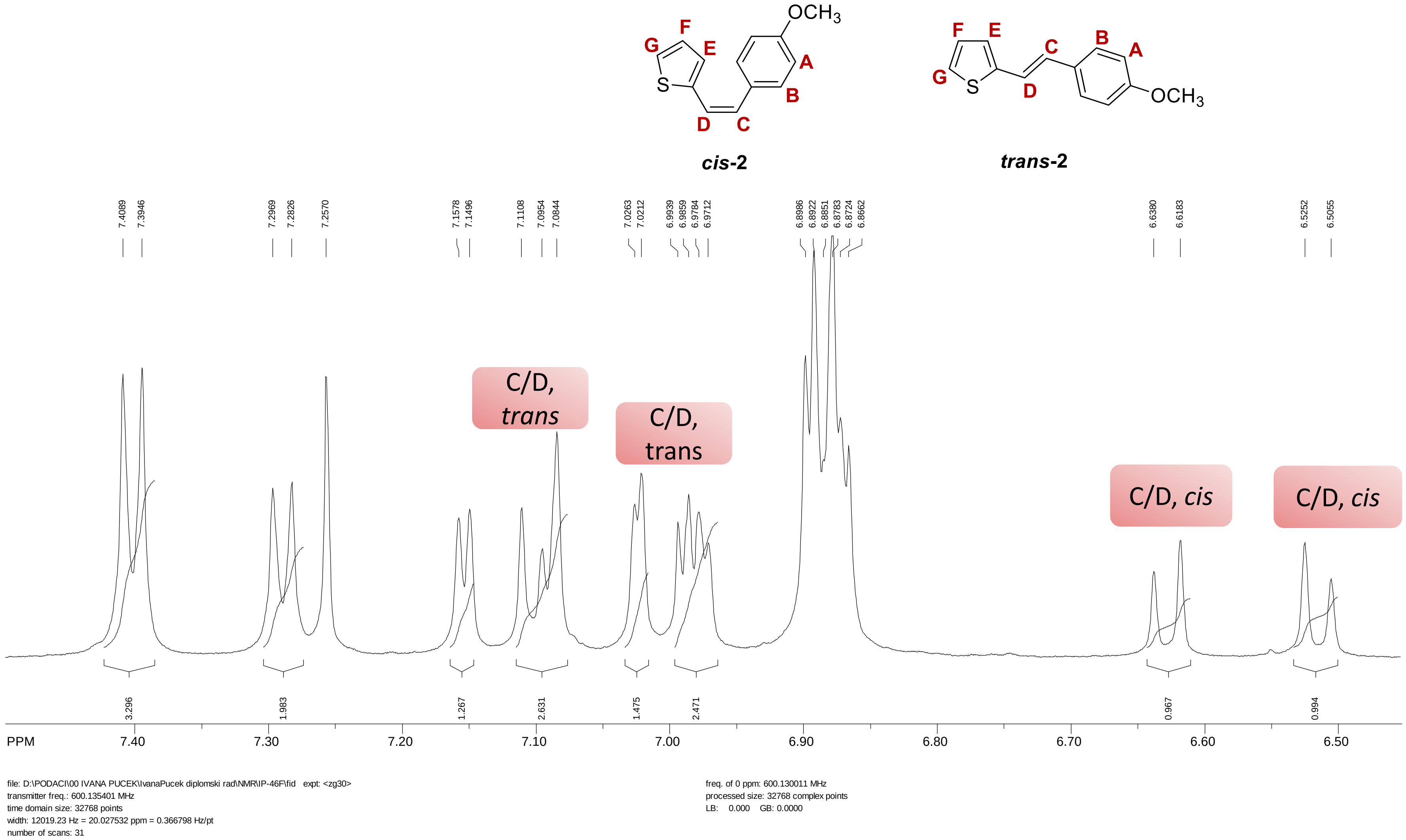

Mass spectra and HRMS analysis of the mixture of geometrical isomers of 2-(4-methoxystyryl)thiophene (*cis*-2 and *trans*-2)

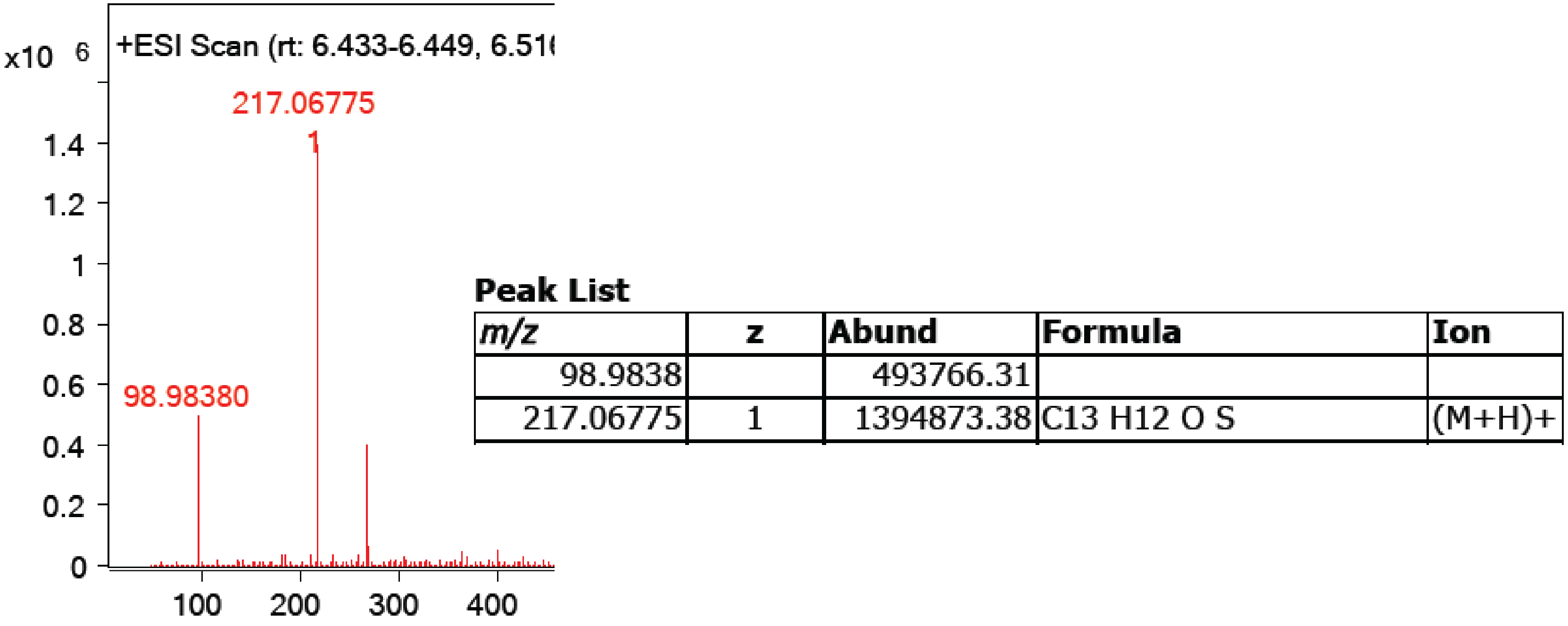

Peak List

m/z	z	Abund	Formula	Ion
98.9838		493766.31		
217.06775	1	1394873.38	C13 H12 O S	(M+H)+

Formula Calculator Results

Formula	Best	Mass	Tgt Mass	Diff (ppm)	Ion Species	Score
C13 H12 O S	True	216.06052	216.06089	1.7	C13 H13 O S	97.61

^{1}H NMR spectrum (600 MHz, $CDCl_3$) of the mixture of geometrical isomers of 2-(4-chlorostyryl)thiophene (*cis*-3 and *trans*-3)

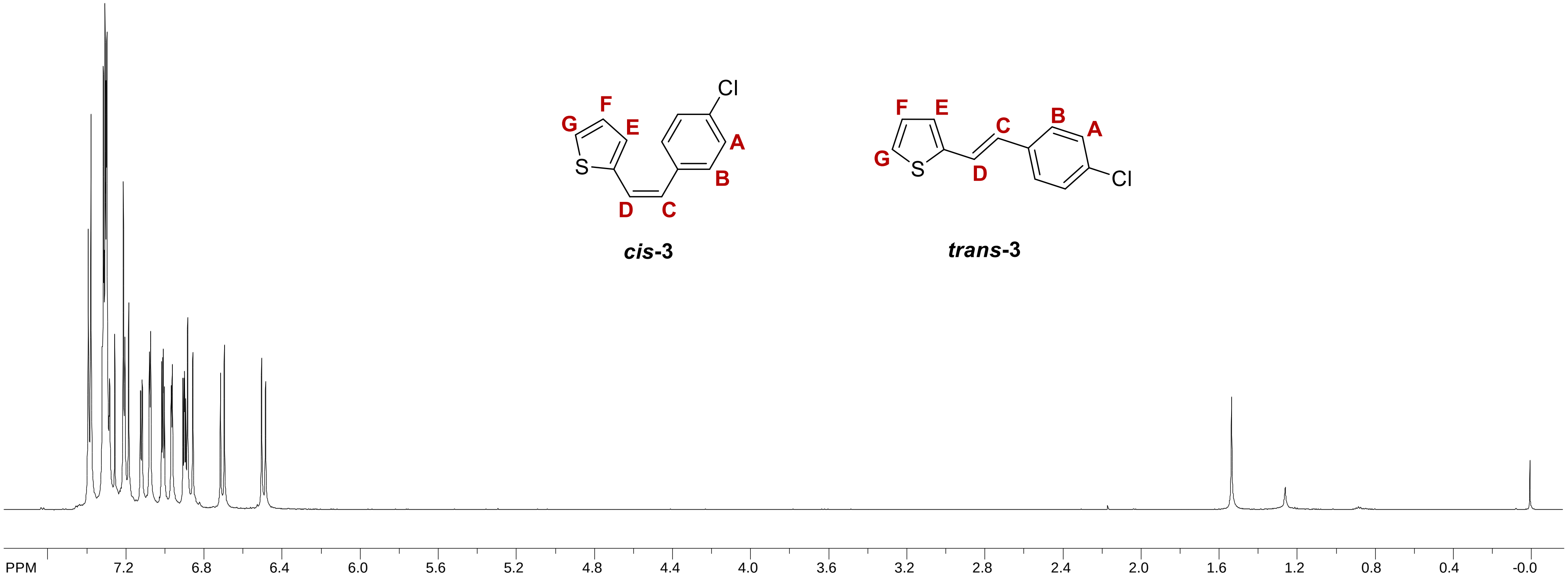

file: D:\PODACI\00 IVANA PUCEK\IvanaPucek diplomski rad\NMR\IP-49F\fid expt: <zg30>
transmitter freq.: 600.135401 MHz
time domain size: 32768 points
width: 12019.23 Hz = 20.027532 ppm = 0.366798 Hz/pt
number of scans: 17

freq. of 0 ppm: 600.130011 MHz
processed size: 32768 complex points
LB: 0.000 GB: 0.0000

A part of the ^{1}H NMR spectrum (600 MHz, $CDCl_3$) of the mixture of geometrical isomers of 2-(4-chlorostyryl)thiophene (*cis*-3 and *trans*-3)

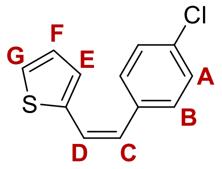

***cis*-3**

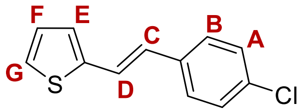

***trans*-3**

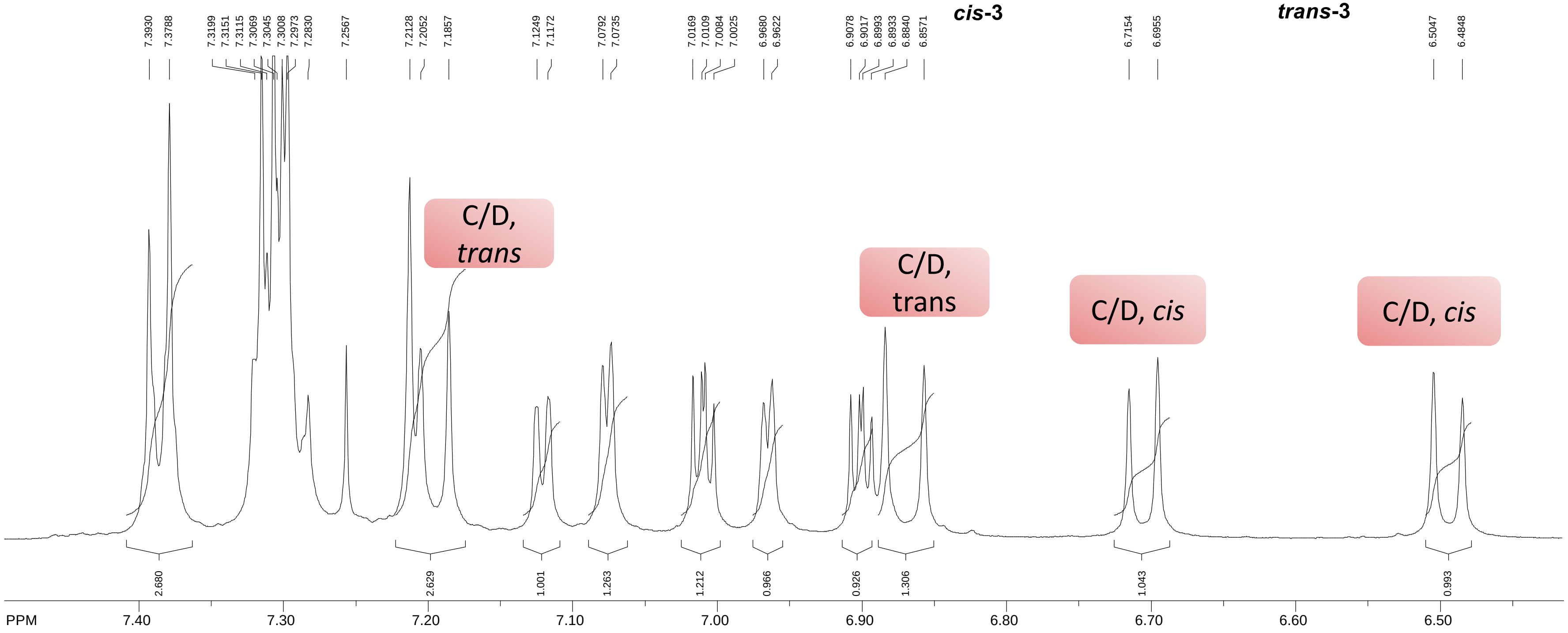

file: D:\PODACI\00 IVANA PUCEK\IvanaPucek diplomski rad\NMR\IP-49F\fid expt: <zg30>
transmitter freq.: 600.135401 MHz
time domain size: 32768 points
width: 12019.23 Hz = 20.027532 ppm = 0.366798 Hz/pt
number of scans: 17

freq. of 0 ppm: 600.130011 MHz
processed size: 32768 complex points
LB: 0.000 GB: 0.0000

Mass spectra and HRMS analysis of the mixture of geometrical isomers of 2-(4-chlorostyryl)thiophene (*cis*-3 and *trans*-3)

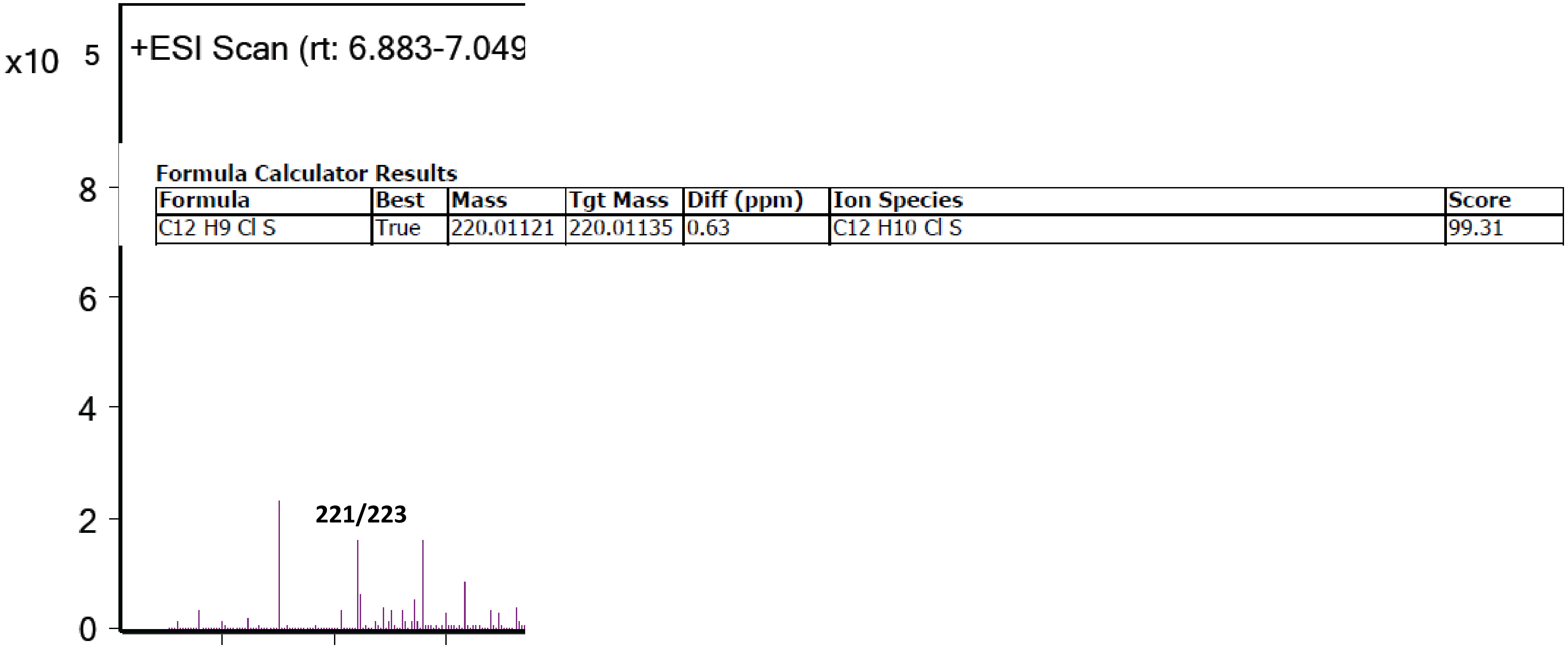

Formula Calculator Results

Formula	Best	Mass	Tgt Mass	Diff (ppm)	Ion Species	Score
C12 H9 Cl S	True	220.01121	220.01135	0.63	C12 H10 Cl S	99.31

^{1}H NMR spectrum (600 MHz, $CDCl_3$) of the mixture of geometrical isomers of 2-(4-(2-(thiophen-2-yl)vinyl)benzonitrile (*cis*-4 and *trans*-4)

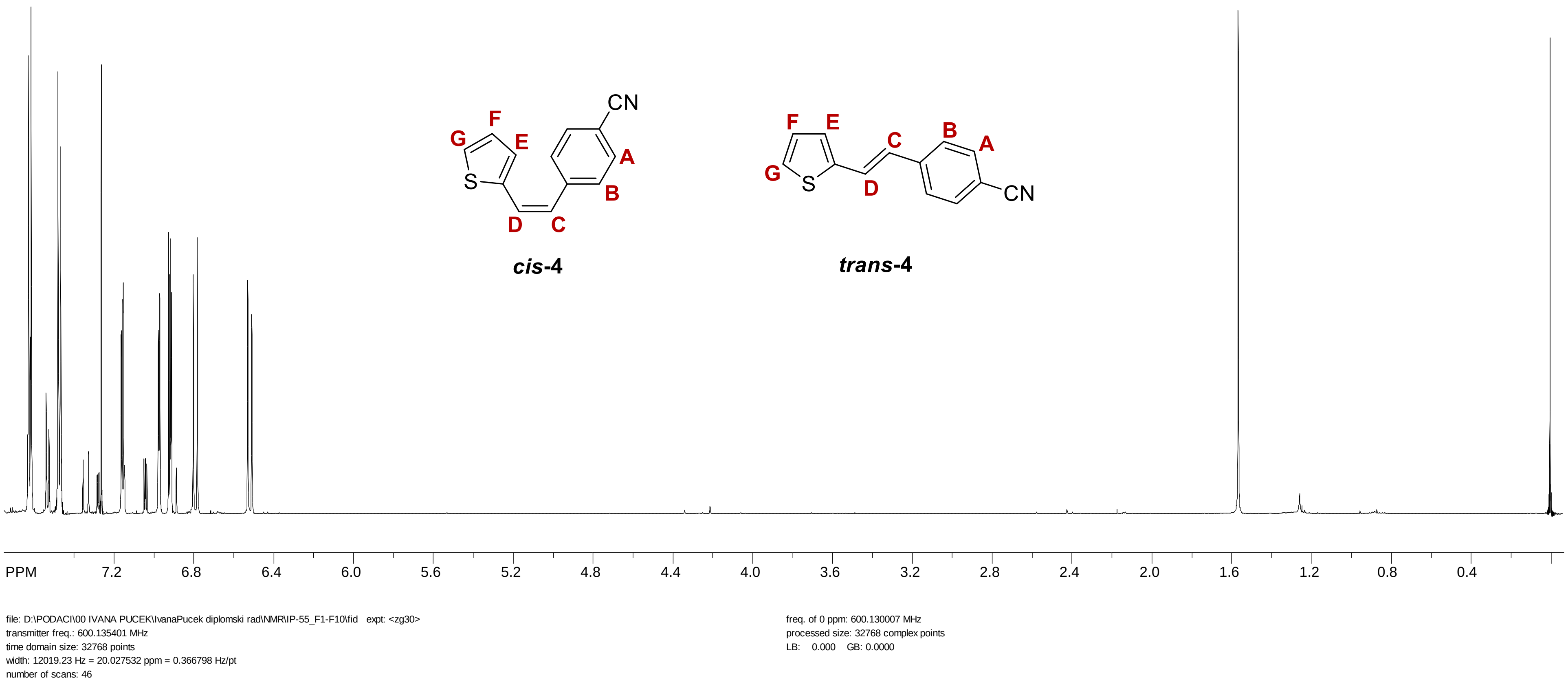

A part of the ^{1}H NMR spectrum (600 MHz, $CDCl_3$) of the mixture of geometrical isomers of 2-(4-(2-(thiophen-2-yl)vinyl)benzonitrile (*cis*-4 and *trans*-4)

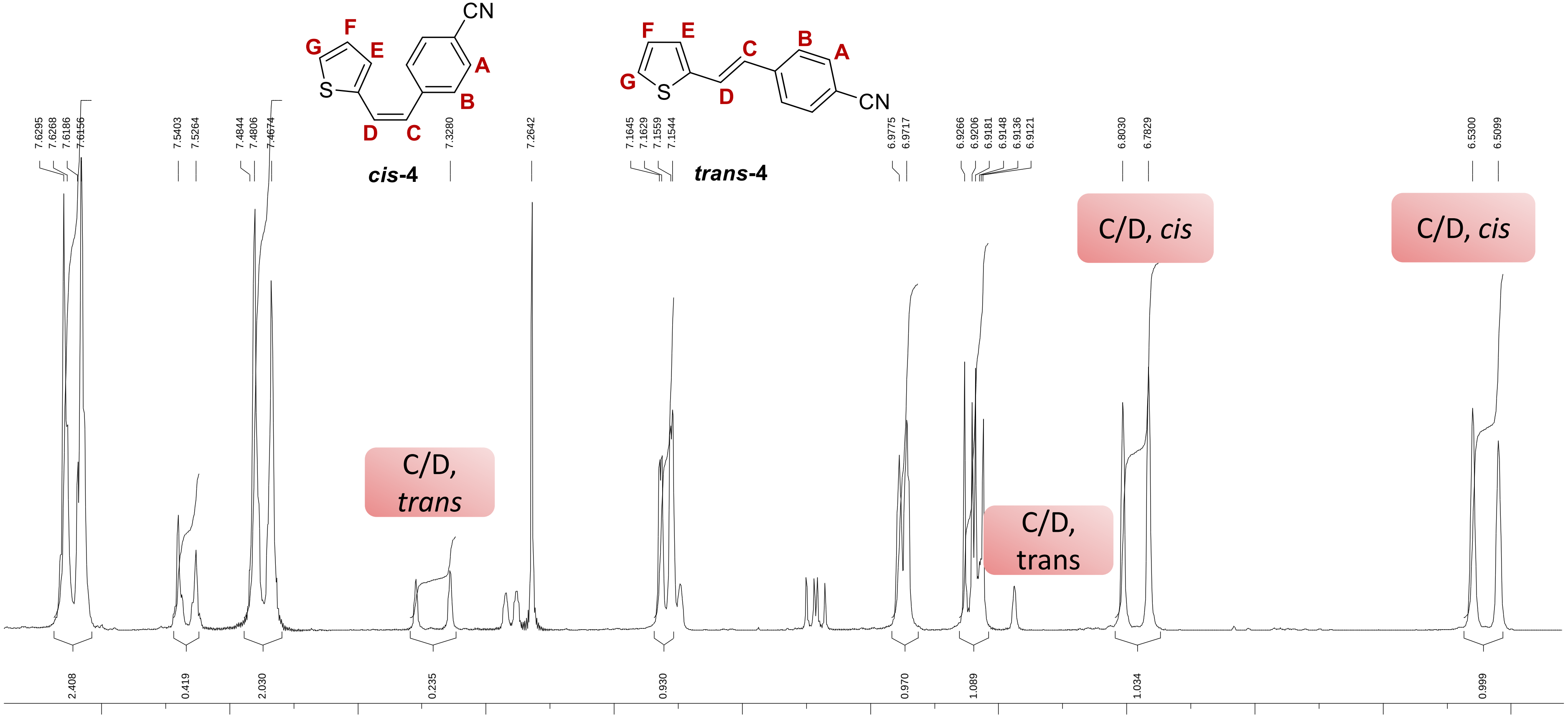

file: D:\PODACI\00 IVANA PUCEK\IvanaPucek diplomski rad\NMR\IP-55_F1-F10\fid expt: <zg30>
transmitter freq.: 600.135401 MHz
time domain size: 32768 points
width: 12019.23 Hz = 20.027532 ppm = 0.366798 Hz/pt
number of scans: 46

freq. of 0 ppm: 600.130007 MHz
processed size: 32768 complex points
LB: 0.000 GB: 0.0000

Mass spectra and HRMS analysis of the mixture of geometrical isomers of 2-(4-(2-(thiophen-2-yl)vinyl)benzonitrile (*cis*-4 and *trans*-4)

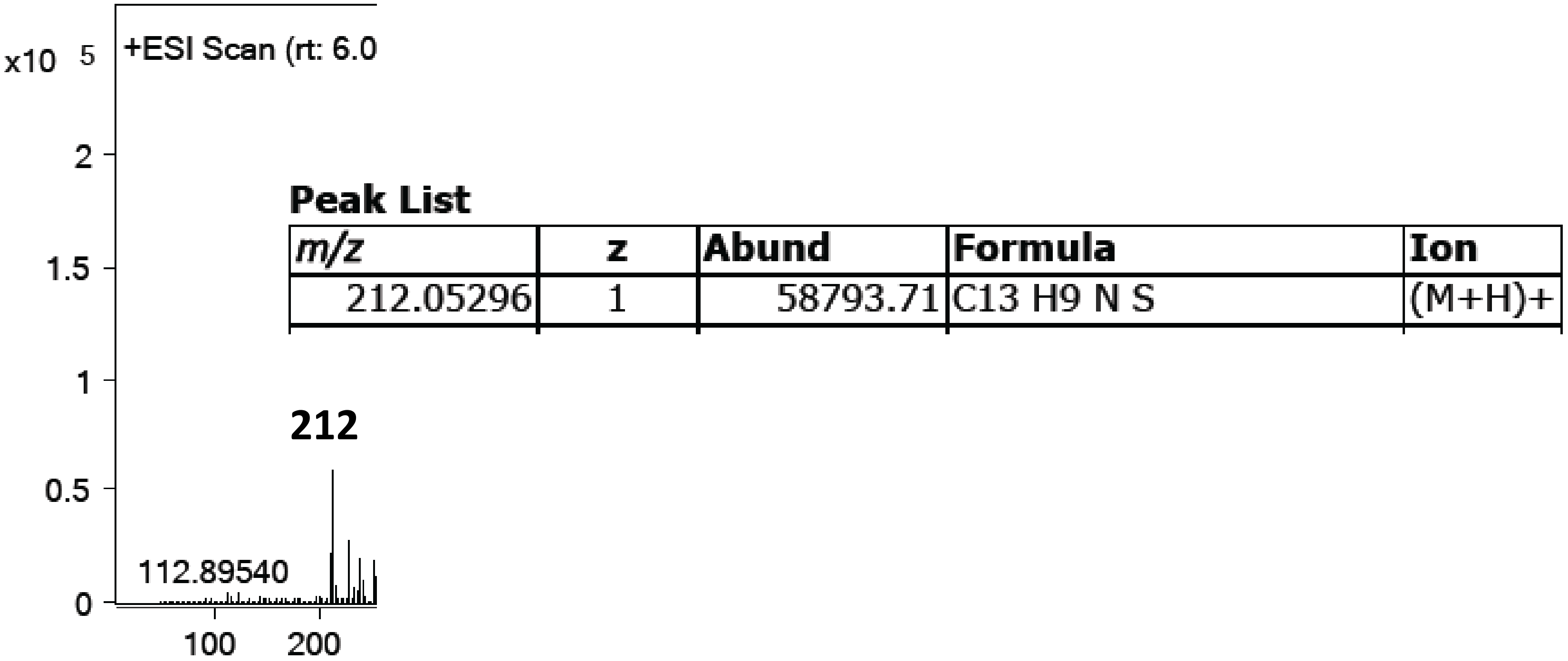

Peak List

m/z	z	Abund	Formula	Ion
212.05296	1	58793.71	C13 H9 N S	(M+H)+

^{1}H NMR spectrum (600 MHz, $CDCl_3$) of the mixture of geometrical isomers of 2-(4-nitrostyryl)thiophene (*cis*-5 and *trans*-5) with a certain amount of *p*-nitrotoluene (separated by coloumn chromatography after formylation)

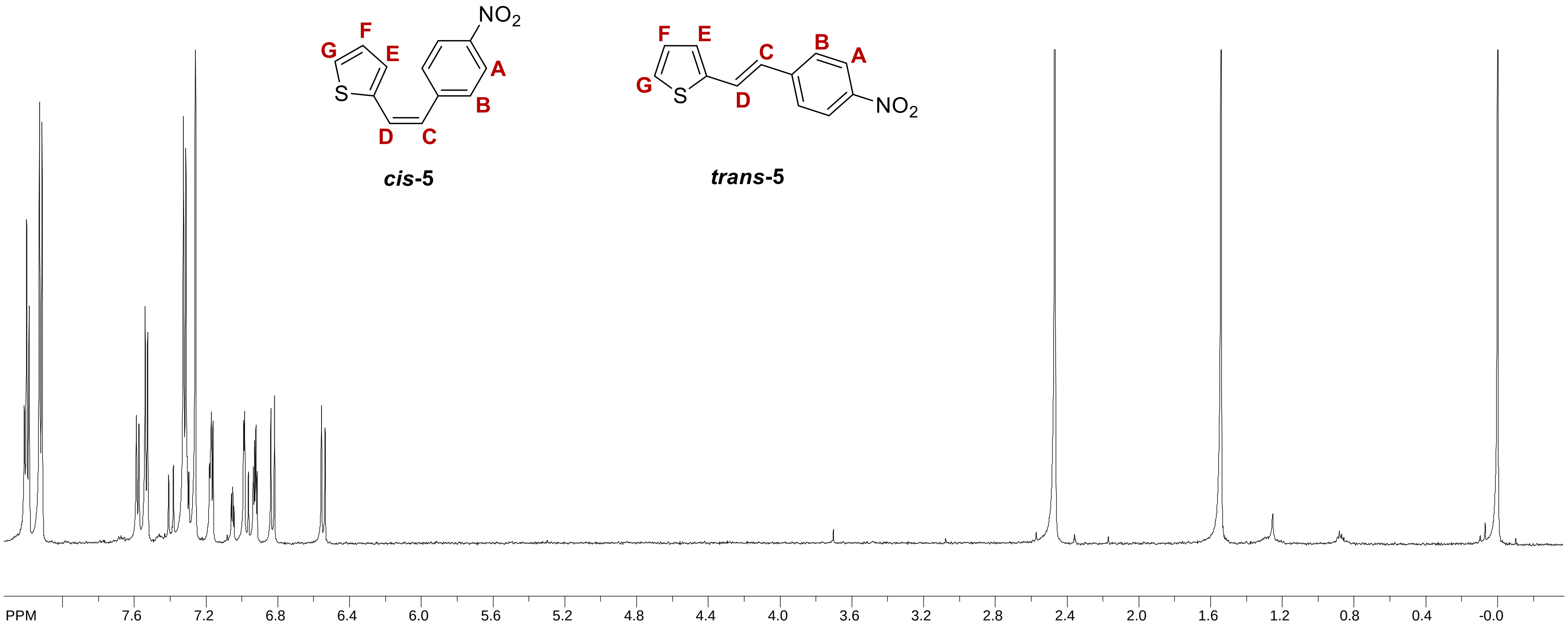

file: D:\PODACI\00 IVANA PUCEK\IvanaPucek diplomski rad\NMR\IP-50_F1\fid expt: <zg30>
transmitter freq.: 600.135401 MHz
time domain size: 32768 points
width: 12019.23 Hz = 20.027532 ppm = 0.366798 Hz/pt
number of scans: 55

freq. of 0 ppm: 600.130010 MHz
processed size: 32768 complex points
LB: 0.000 GB: 0.0000

A part of the ^{1}H NMR spectrum (600 MHz, $CDCl_3$) of the mixture of geometrical isomers of 2-(4-nitrostyryl)thiophene (*cis*-5 and *trans*-5) with a certain amount of p-nitrotoluene (separated by coloumn chromatography after formylation)

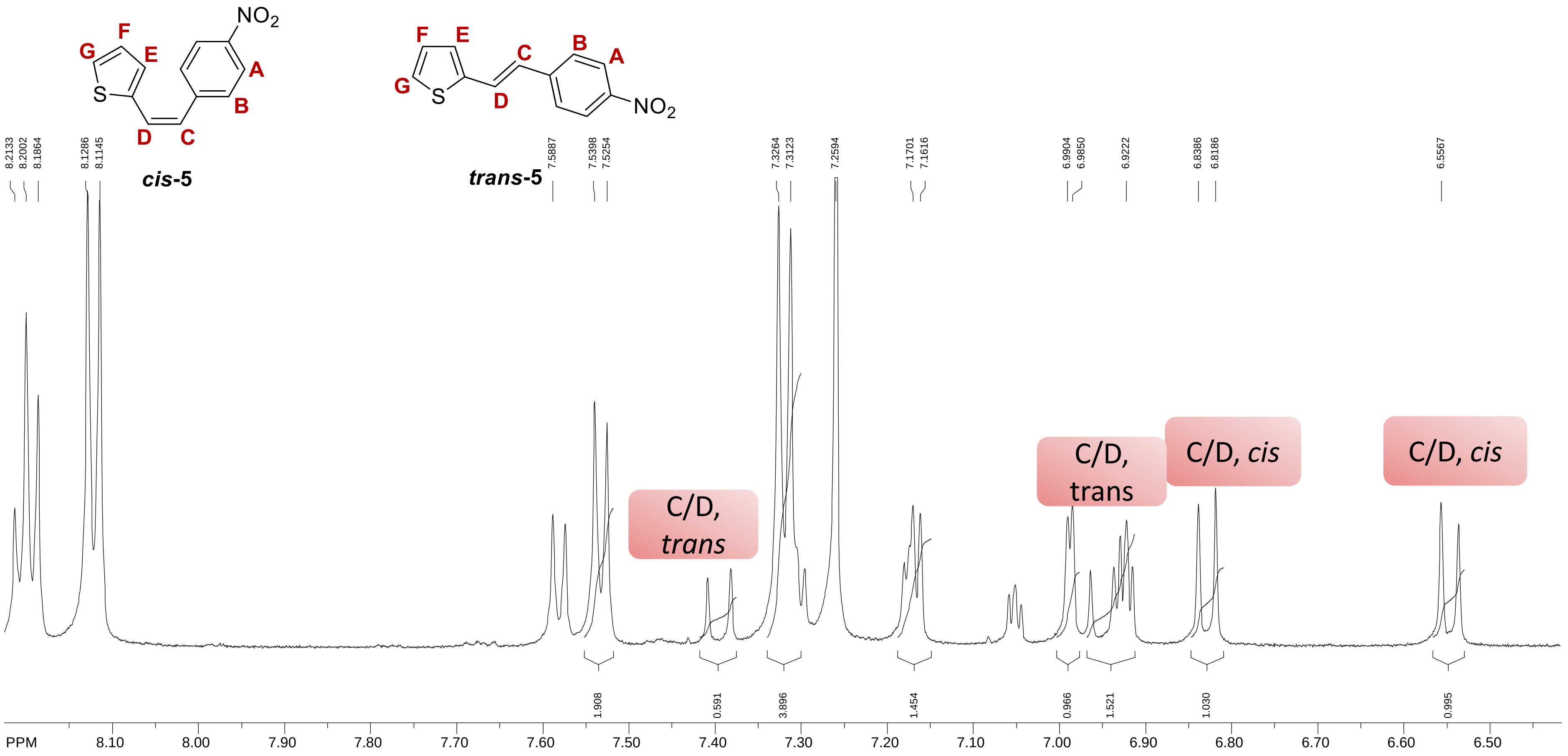

file: D:\PODACI\00 IVANA PUCEK\IvanaPucek diplomski rad\NMR\IP-50_F1\fid expt: <zg30>
transmitter freq.: 600.135401 MHz
time domain size: 32768 points
width: 12019.23 Hz = 20.027532 ppm = 0.366798 Hz/pt
number of scans: 55

freq. of 0 ppm: 600.130010 MHz
processed size: 32768 complex points
LB: 0.000 GB: 0.0000

Mass spectra and HRMS analysis of the mixture of geometrical isomers of 2-(4-nitrostyryl)thiophene (*cis*-5 and *trans*-5)

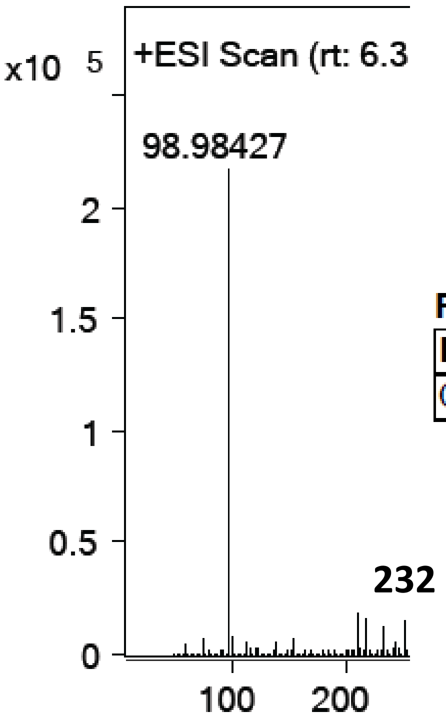

Formula Calculator Results

Formula	Best	Mass	Tgt Mass
C12 H9 N O2 S	True	231.03565	231.0354

^{1}H NMR spectrum (600 MHz, $CDCl_3$) of the mixture of geometrical isomers of *N,N*-dimethyl-4-(2-(thiophen-2-yl)vinyl)aniline (*cis*-6 and *trans*-6)

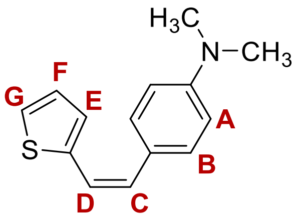

***cis*-6**

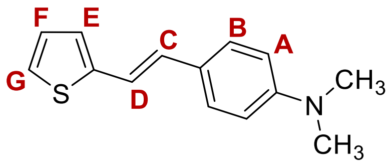

***trans*-6**

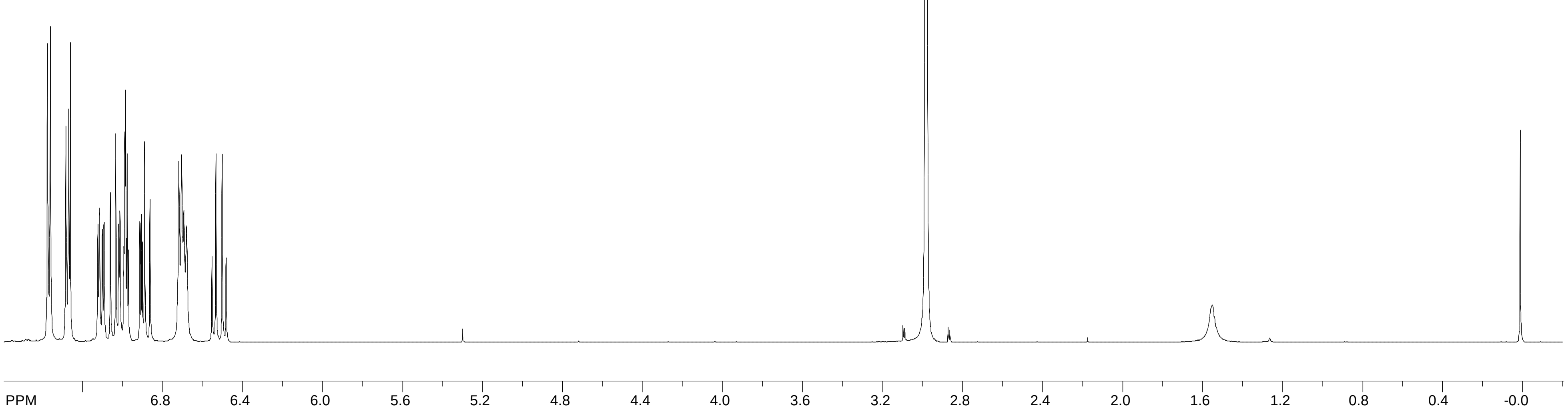

file: D:\PODACI\00 IVANA PUCEK\IvanaPucek diplomski rad\NMR\IP-60_F3-F6\fid expt: <zg30>
transmitter freq.: 600.135401 MHz
time domain size: 32768 points
width: 12019.23 Hz = 20.027532 ppm = 0.366798 Hz/pt
number of scans: 49

freq. of 0 ppm: 600.130009 MHz
processed size: 32768 complex points
LB: 0.000 GB: 0.0000

A part of the ^{1}H NMR spectrum (600 MHz, $CDCl_3$) of the mixture of geometrical isomers of *N,N*-dimethyl-4-(2-(thiophen-2-yl)vinyl)aniline (*cis*-6 and *trans*-6)

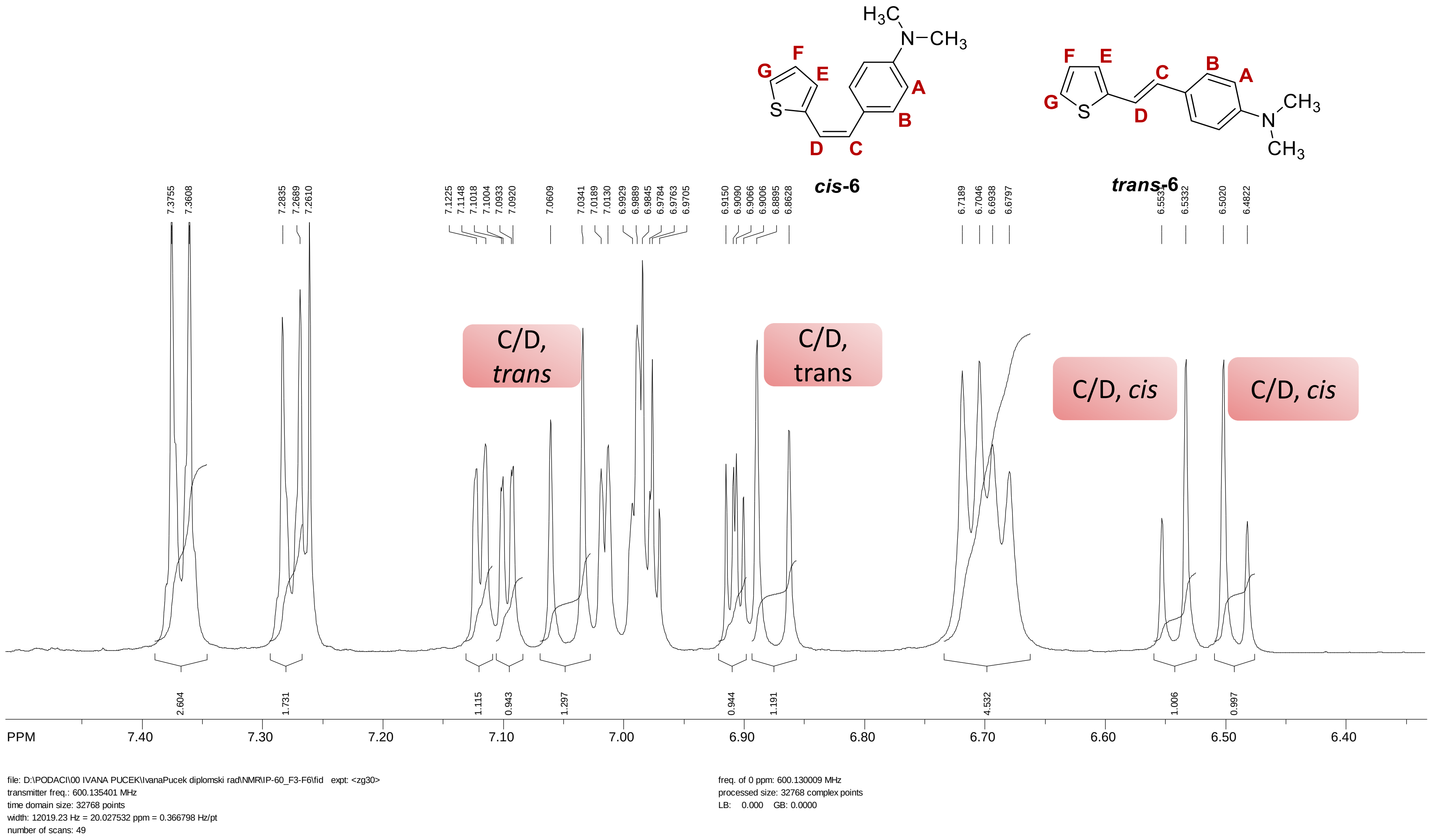

Mass spectra and HRMS analysis of the mixture of geometrical isomers of *N*,*N*-dimethyl-4-(2-(thiophen-2-yl)vinyl)aniline (*cis*-6 and *trans*-6)

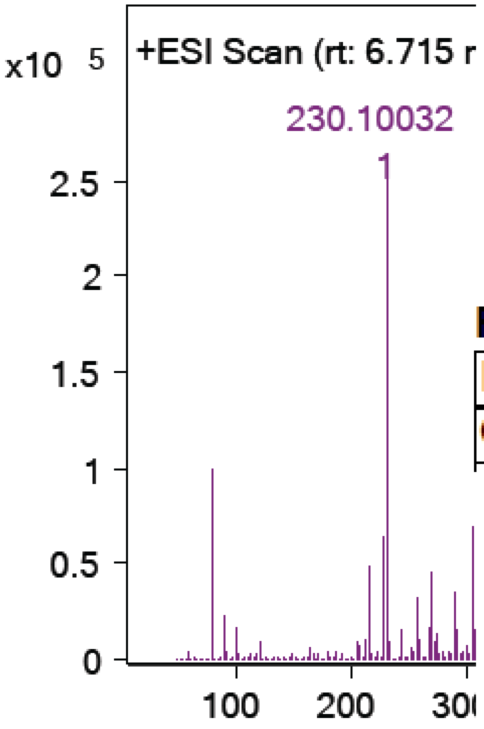

Formula Calculator Results

Formula	Best	Mass	Tgt Mass
C14 H15 N S	True	229.09286	229.09252

^{1}H NMR spectrum (600 MHz, $CDCl_3$) of the mixture of geometrical isomers of 5-(4-methylstyryl)thiophene-2-carbaldehyde (*cis*-7 and *trans*-7)

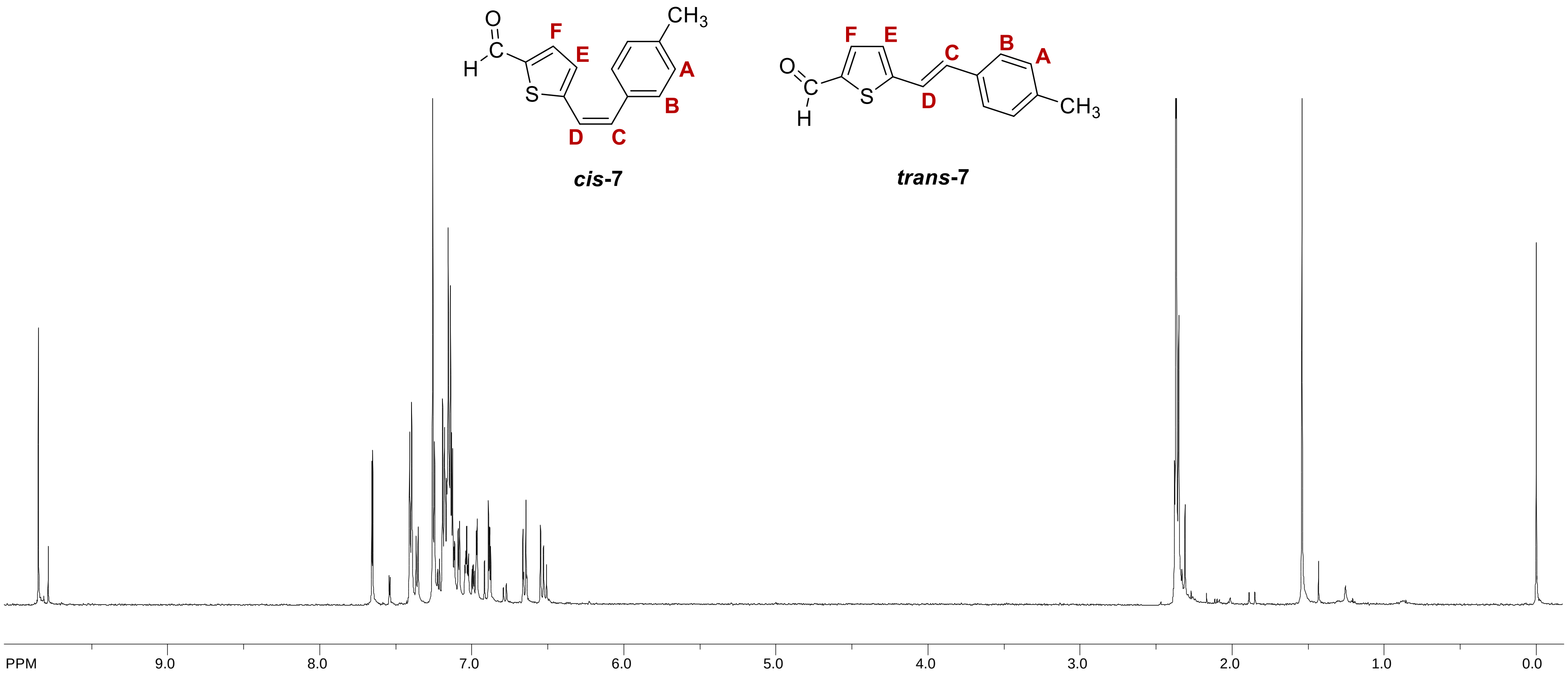

file: D:\PODACI\00 IVANA PUCEK\IvanaPucek diplomski rad\NMR\IP-47\fid expt: <zg30>
transmitter freq.: 600.135401 MHz
time domain size: 32768 points
width: 12019.23 Hz = 20.027532 ppm = 0.366798 Hz/pt
number of scans: 20

freq. of 0 ppm: 600.130011 MHz
processed size: 32768 complex points
LB: 0.000 GB: 0.0000

A part of the ^1H NMR spectrum (600 MHz, $CDCl_3$) of the mixture of geometrical isomers of 5-(4-methylstyryl)thiophene-2-carbaldehyde (*cis*-7 and *trans*-7)

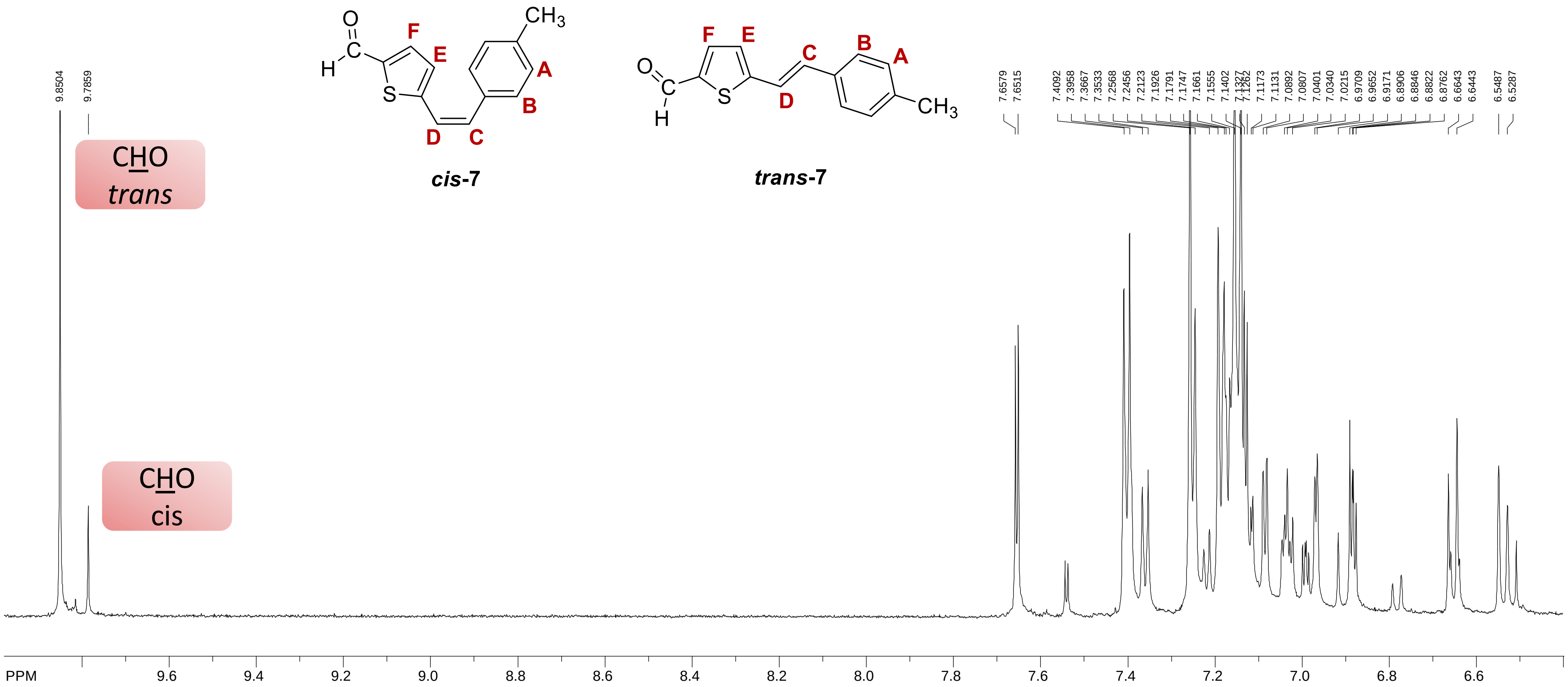

file: D:\PODACI\00 IVANA PUCEK\IvanaPucek diplomski rad\NMR\IP-47\fid expt: <zg30>
transmitter freq.: 600.135401 MHz
time domain size: 32768 points
width: 12019.23 Hz = 20.027532 ppm = 0.366798 Hz/pt
number of scans: 20

freq. of 0 ppm: 600.130011 MHz
processed size: 32768 complex points
LB: 0.000 GB: 0.0000

Mass spectra and HRMS analysis of the mixture of geometrical isomers of 5-(4-methylstyryl)thiophene-2-carbaldehyde (*cis*-7 and *trans*-7)

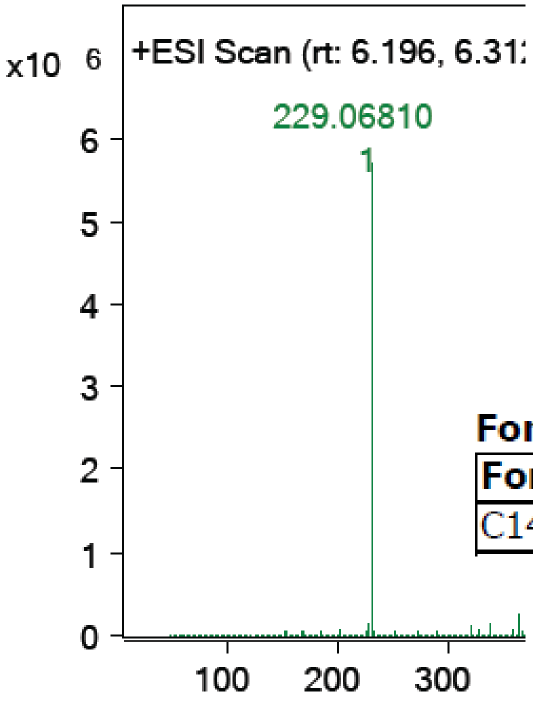

Formula Calculator Results

Formula	Best	Mass	Tgt Mass	Diff (ppm)
C14 H12 O S	True	228.06088	228.06089	0.03

^{1}H NMR spectrum (600 MHz, $CDCl_3$) of the mixture of geometrical isomers of 5-(4-methoxystyryl)thiophene-2-carbaldehyde (*cis*-8 and *trans*-8)

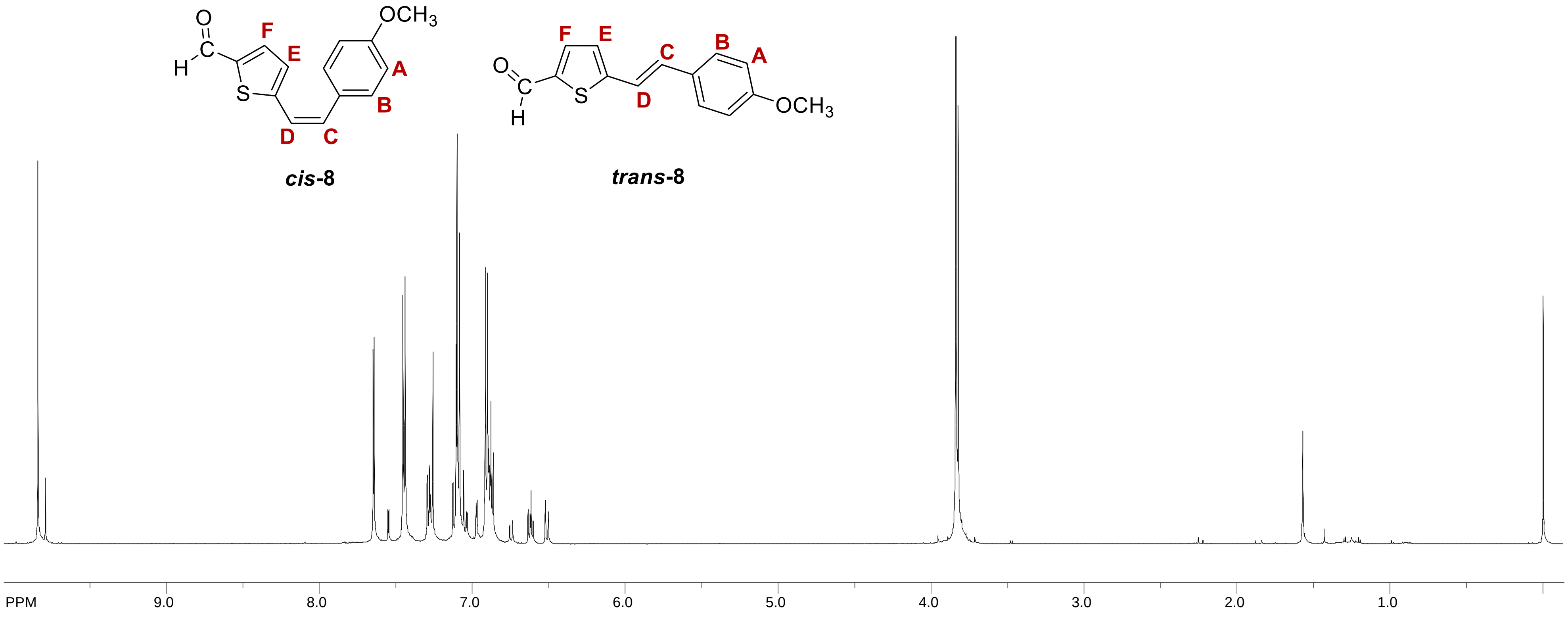

file: D:\PODACI\00 IVANA PUCEK\IvanaPucek diplomski rad\NMR\IP-48\fid expt: <zg30>
transmitter freq.: 600.135401 MHz
time domain size: 32768 points
width: 12019.23 Hz = 20.027532 ppm = 0.366798 Hz/pt
number of scans: 16

freq. of 0 ppm: 600.130011 MHz
processed size: 32768 complex points
LB: 0.000 GB: 0.0000

A part of the ^{1}H NMR spectrum (600 MHz, $CDCl_3$) of the mixture of geometrical isomers of 5-(4-methoxystyryl)thiophene-2-carbaldehyde (*cis*-8 and *trans*-8)

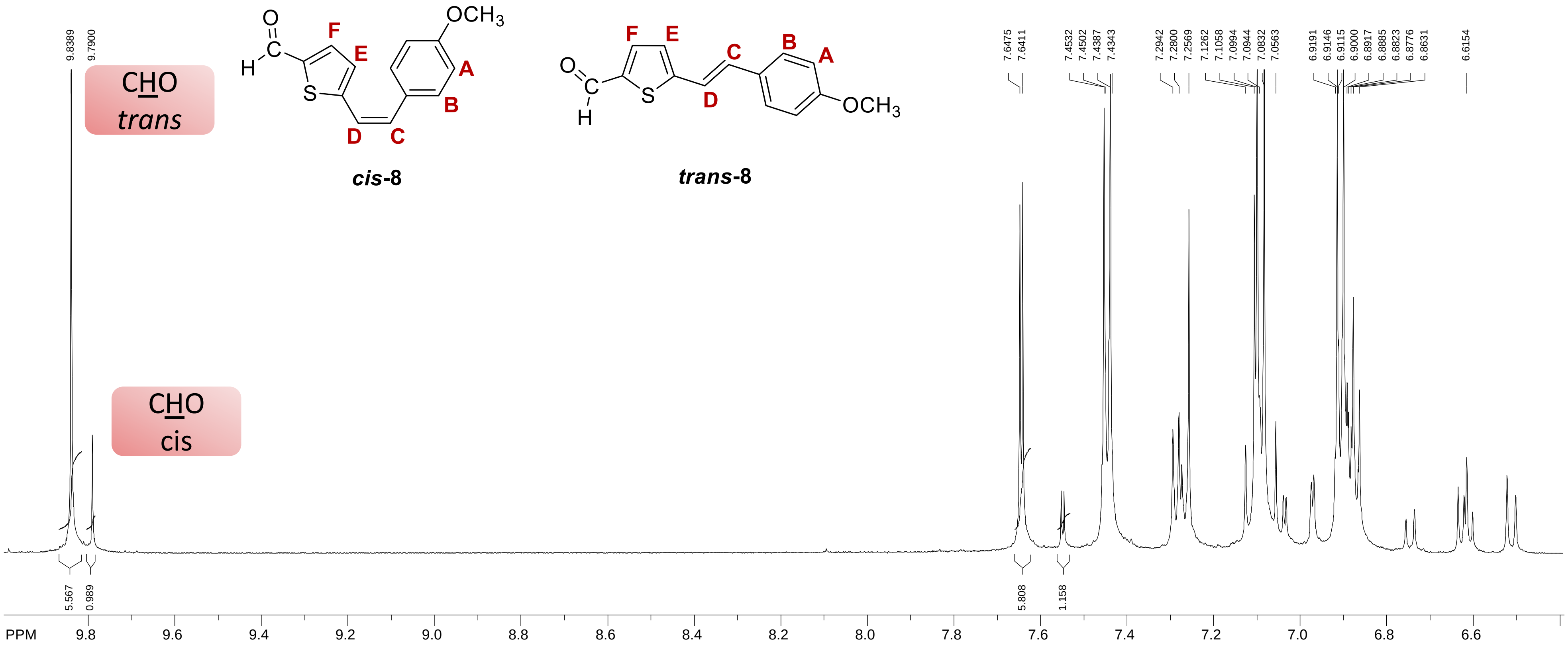

file: D:\PODACI\00 IVANA PUCEK\IvanaPucek diplomski rad\NMR\IP-48\fid expt: <zg30>
transmitter freq.: 600.135401 MHz
time domain size: 32768 points
width: 12019.23 Hz = 20.027532 ppm = 0.366798 Hz/pt
number of scans: 16

freq. of 0 ppm: 600.130011 MHz
processed size: 32768 complex points
LB: 0.000 GB: 0.0000

Mass spectra and HRMS analysis of the mixture of geometrical isomers of 5-(4-methoxystyryl)thiophene-2-carbaldehyde (*cis*-8 and *trans*-8)

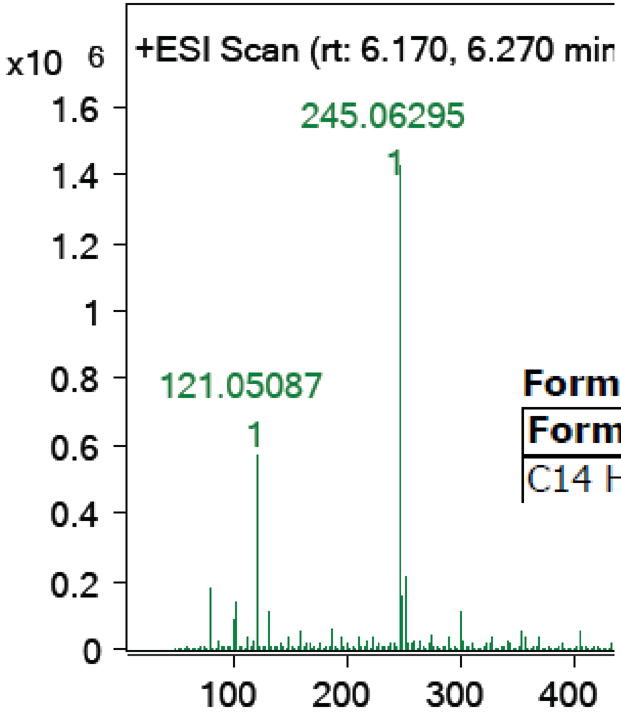

Formula Calculator Results

Formula	Best	Mass	Tgt Mass	Diff (ppm)
C14 H12 O2 S	True	244.05573	244.0558	0.31

^{1}H NMR spectrum (600 MHz, $CDCl_3$) of the mixture of geometrical isomers of 5-(4-chlorostyryl)thiophene-2-carbaldehyde (*cis*-9 and *trans*-9)

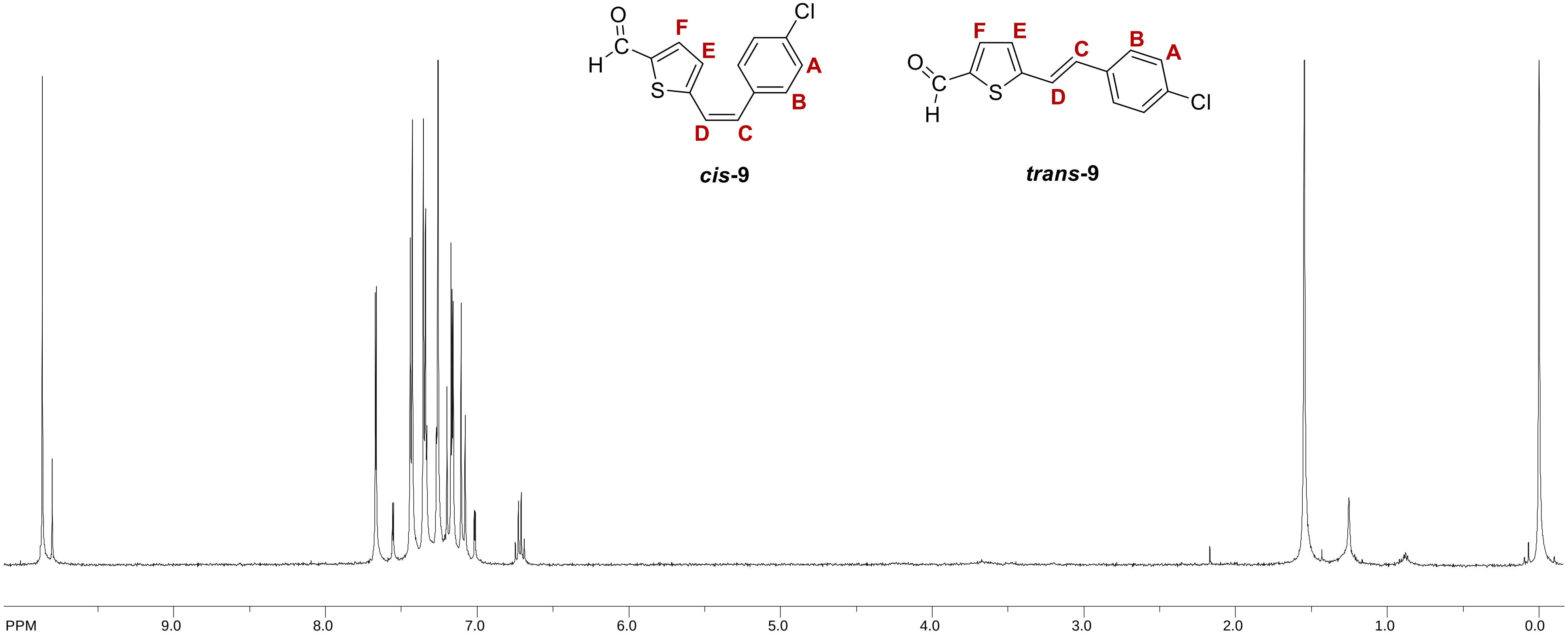

file: D:\PODACI\00 IVANA PUCEK\IvanaPucek diplomski rad\NMR\IP-53_F2\fid expt: <zg30>
transmitter freq.: 600.135401 MHz
time domain size: 32768 points
width: 12019.23 Hz = 20.027532 ppm = 0.366798 Hz/pt
number of scans: 51

freq. of 0 ppm: 600.130012 MHz
processed size: 32768 complex points
LB: 0.000 GB: 0.0000

A part of the ^{1}H NMR spectrum (600 MHz, $CDCl_3$) of the mixture of geometrical isomers of 5-(4-chlorostyryl)thiophene-2-carbaldehyde (*cis*-9 and *trans*-9)

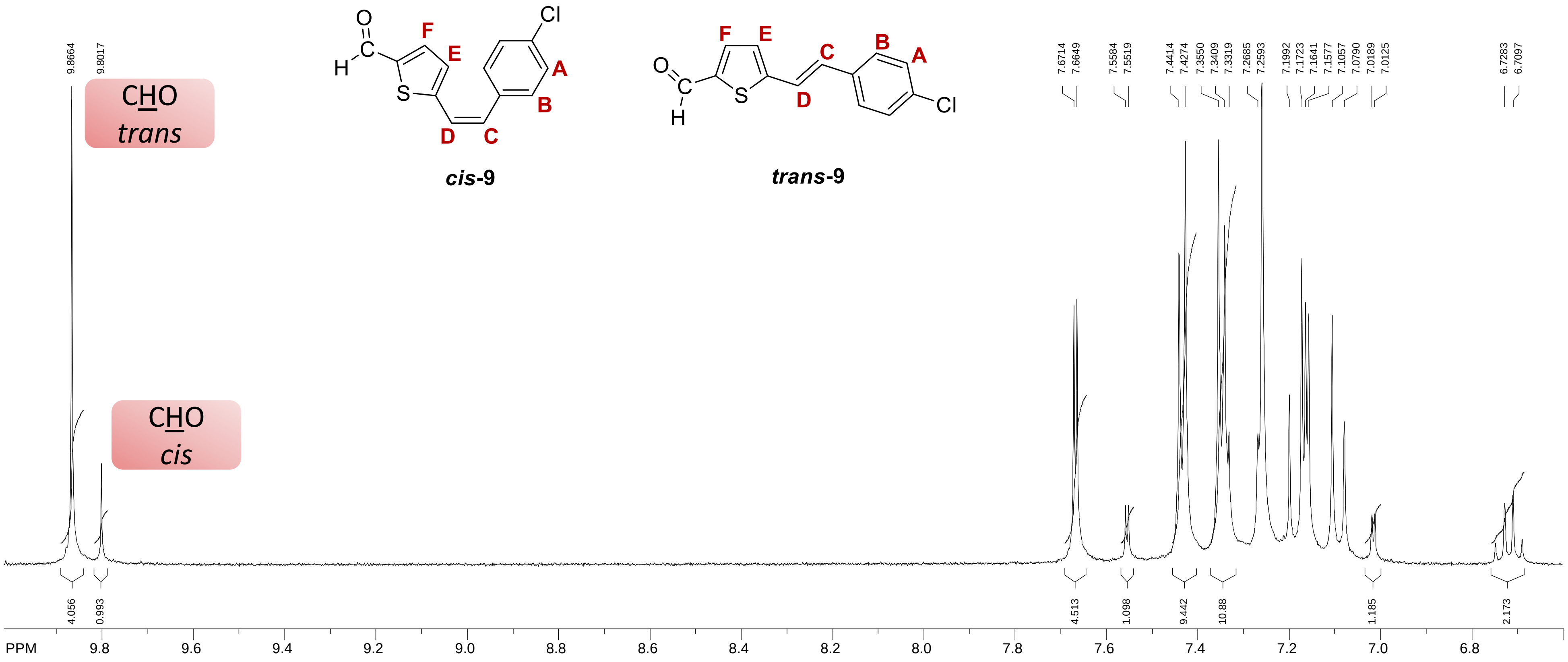

file: D:\PODACI\00 IVANA PUCEK\IvanaPucek diplomski rad\NMR\IP-53_F2\fid expt: <zg30>
transmitter freq.: 600.135401 MHz
time domain size: 32768 points
width: 12019.23 Hz = 20.027532 ppm = 0.366798 Hz/pt
number of scans: 51

freq. of 0 ppm: 600.130012 MHz
processed size: 32768 complex points
LB: 0.000 GB: 0.0000

Mass spectra and HRMS analysis of the mixture of geometrical isomers of 5-(4-chlorostyryl)thiophene-2-carbaldehyde (*cis*-9 and *trans*-9)

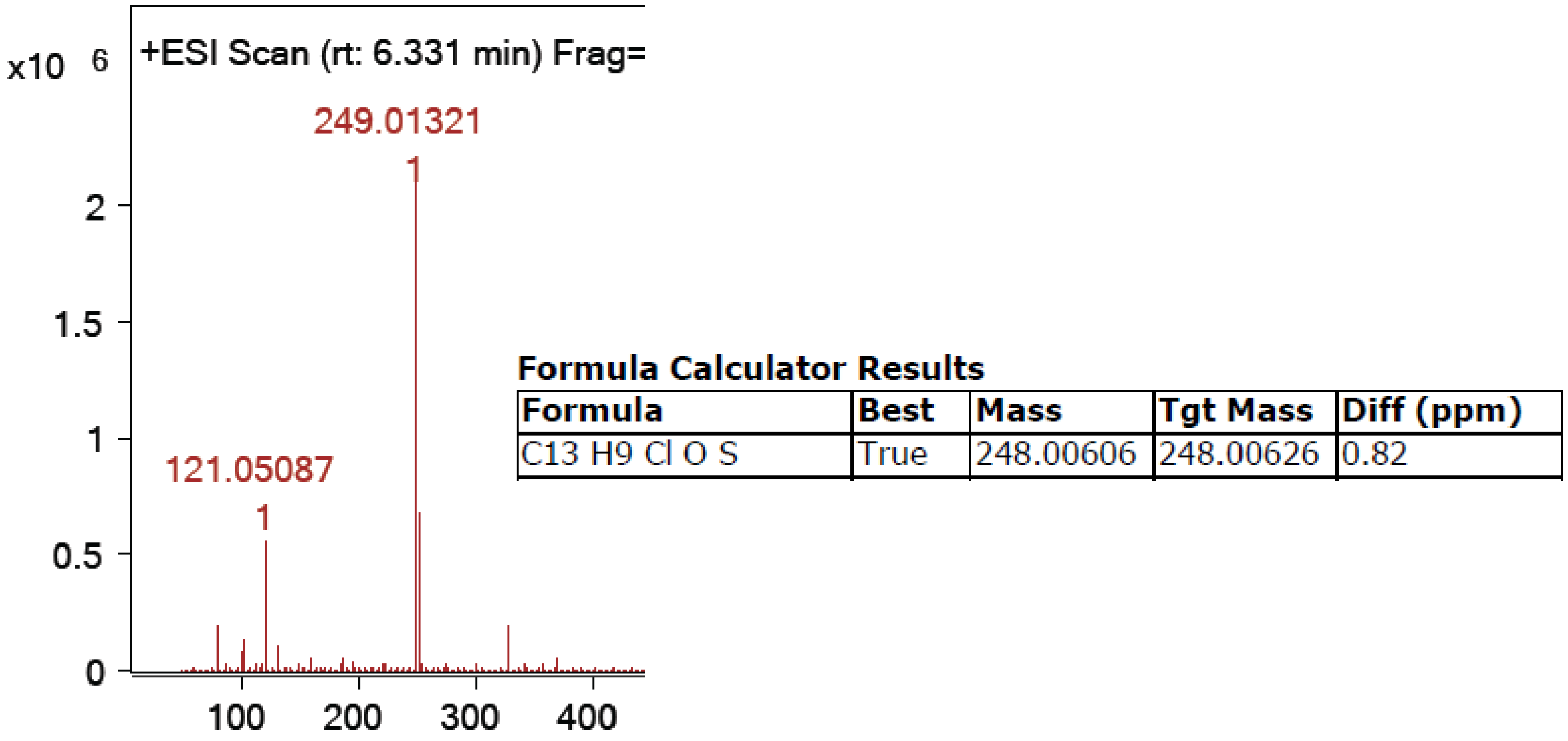

Formula Calculator Results

Formula	Best	Mass	Tgt Mass	Diff (ppm)
C13 H9 Cl O S	True	248.00606	248.00626	0.82

^{1}H NMR spectrum (600 MHz, $CDCl_3$) of the mixture of geometrical isomers of 4-(2-(5-formylthiophen-2-yl)vinyl)benzonitrile (*cis*-10 and *trans*-10)

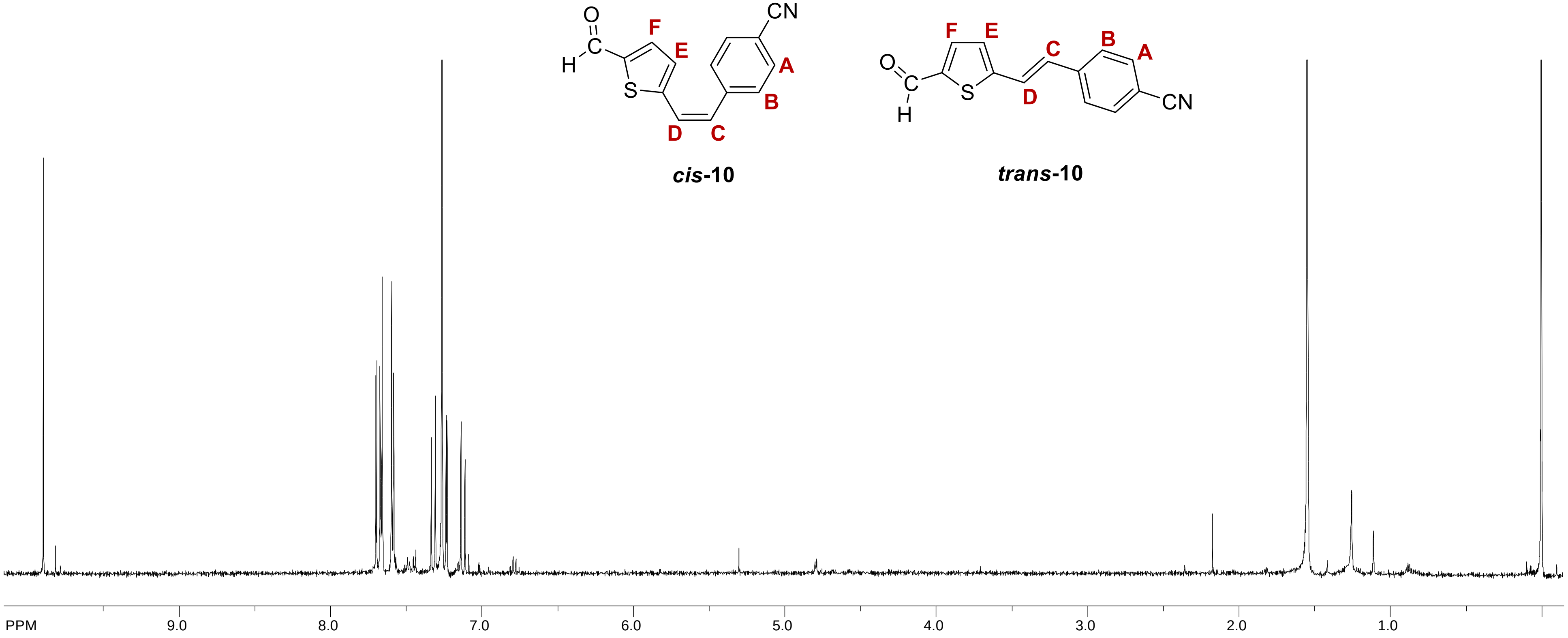

file: D:\PODACI\00 IVANA PUCEK\IvanaPucek diplomski rad\NMR\IP-59_F26-F30\fid expt: <zg30>
transmitter freq.: 600.135401 MHz
time domain size: 32768 points
width: 12019.23 Hz = 20.027532 ppm = 0.366798 Hz/pt
number of scans: 30

freq. of 0 ppm: 600.130007 MHz
processed size: 32768 complex points
LB: 0.000 GB: 0.0000

A part of the ^{1}H NMR spectrum (600 MHz, CDCl$_3$) of the mixture of geometrical isomers of 4-(2-(5-formylthiophen-2-yl)vinyl)benzonitrile (*cis*-10 and *trans*-10)

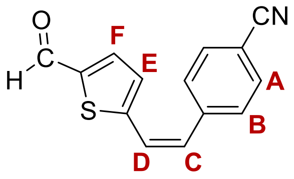

***cis*-10**

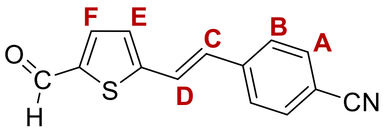

***trans*-10**

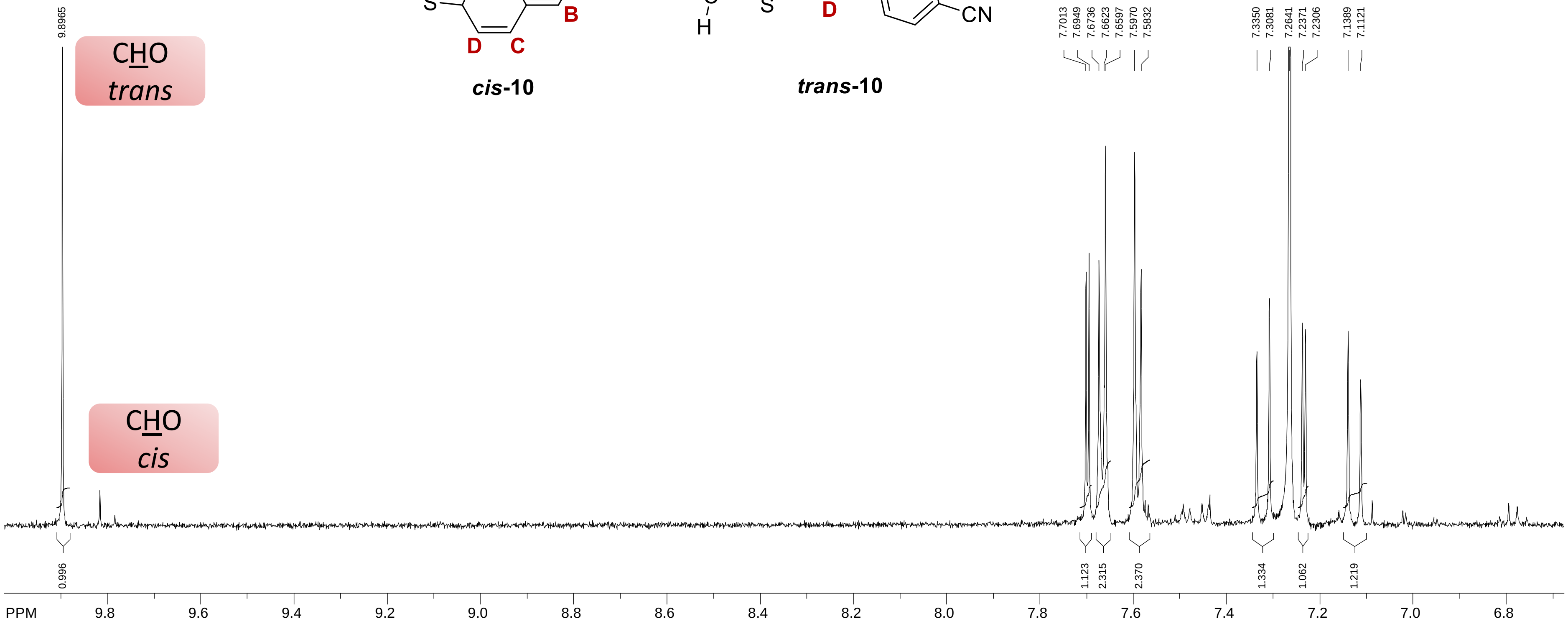

file: D:\PODACI\00 IVANA PUCEK\IvanaPucek diplomski rad\NMR\IP-59_F26-F30\fid expt: <zg30>
transmitter freq.: 600.135401 MHz
time domain size: 32768 points
width: 12019.23 Hz = 20.027532 ppm = 0.366798 Hz/pt
number of scans: 30

freq. of 0 ppm: 600.130007 MHz
processed size: 32768 complex points
LB: 0.000 GB: 0.0000

^{1}H NMR spectrum (600 MHz, $CDCl_3$) of the mixture of geometrical isomers of 5-(4-nitrostyryl)thiophene-2-carbaldehyde (*cis*-11 and *trans*-11)

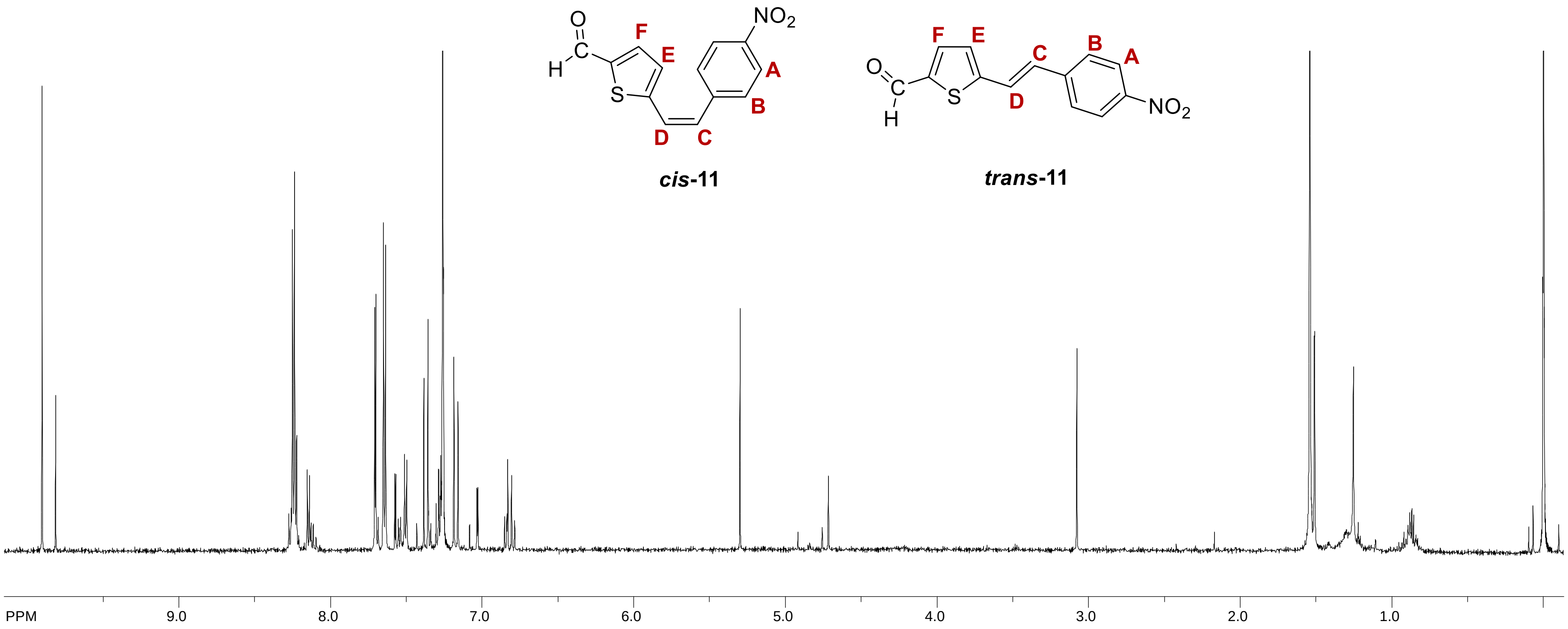

file: D:\PODACI\00 IVANA PUCEK\IvanaPucek diplomski rad\NMR\IP-64_F7-F10\fid expt: <zg30>
transmitter freq.: 600.135401 MHz
time domain size: 32768 points
width: 12019.23 Hz = 20.027532 ppm = 0.366798 Hz/pt
number of scans: 77

freq. of 0 ppm: 600.130010 MHz
processed size: 32768 complex points
LB: 0.000 GB: 0.0000

A part of the ^{1}H NMR spectrum (600 MHz, $CDCl_3$) of the mixture of geometrical isomers of 5-(4-nitrostyryl)thiophene-2-carbaldehyde (*cis*-11 and *trans*-11)

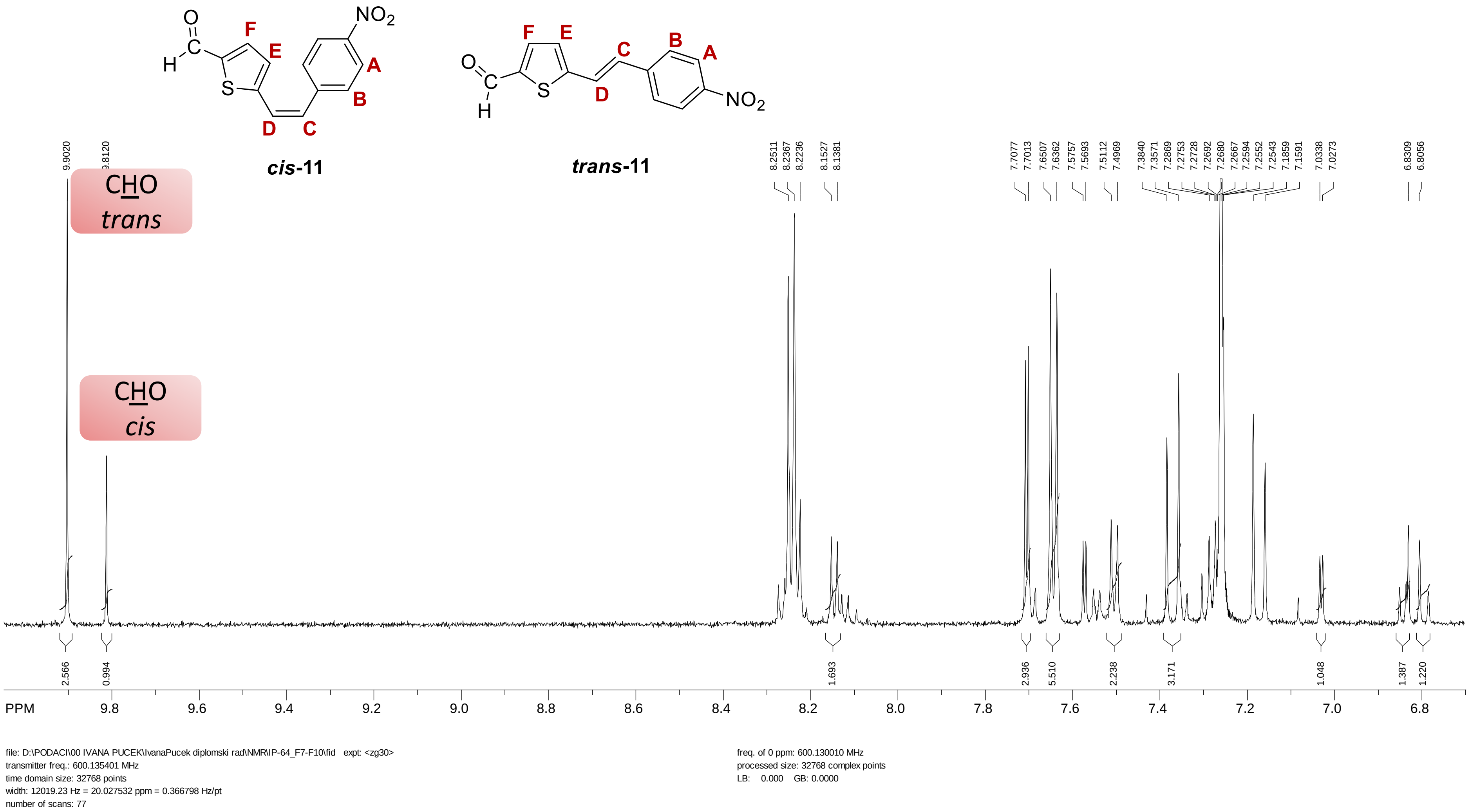

Mass spectra and HRMS analysis of the mixture of geometrical isomers of 5-(4-nitrostyryl)thiophene-2-carbaldehyde (*cis*-11 and *trans*-11)

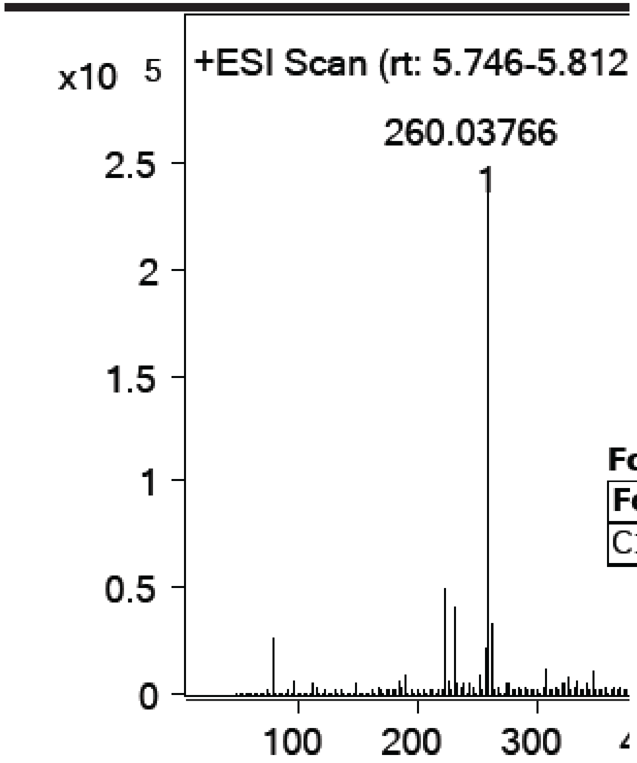

Formula Calculator Results

Formula	Best	Mass	Tgt Mass	Diff (ppm)
C13 H9 N O3 S	True	259.03056	259.03031	-0.96

^{1}H NMR spectrum (600 MHz, $CDCl_3$) of the mixture of geometrical isomers of 5-(4-(dimethylamino)styryl)thiophene-2-carbaldehyde (*cis*-12 and *trans*-12)

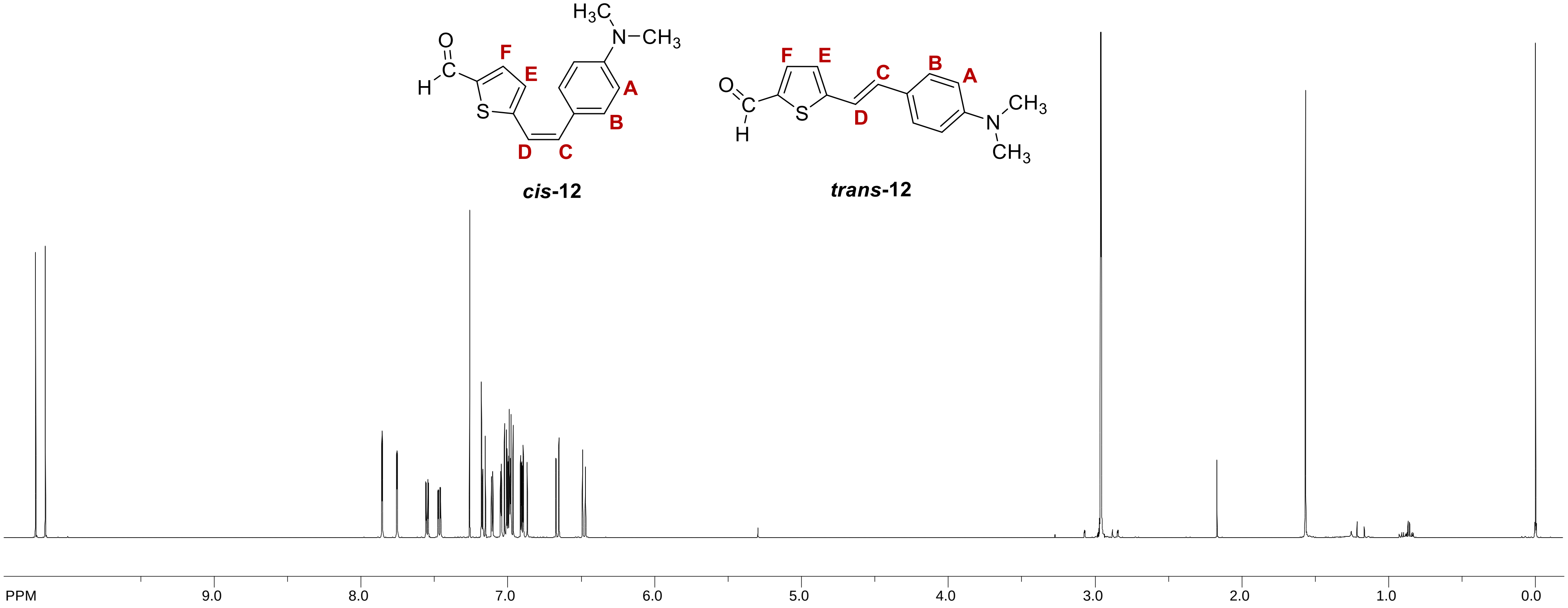

file: D:\PODACI\00 IVANA PUCEK\IvanaPucek diplomski rad\NMR\IP-63_F9-F12_F7-F17\fid expt: <zg30>
transmitter freq.: 600.135401 MHz
time domain size: 32768 points
width: 12019.23 Hz = 20.027532 ppm = 0.366798 Hz/pt
number of scans: 11

freq. of 0 ppm: 600.130010 MHz
processed size: 32768 complex points
LB: 0.000 GB: 0.0000

A part of the ^{1}H NMR spectrum (600 MHz, $CDCl_3$) of the mixture of geometrical isomers of 5-(4-(dimethylamino)styryl)thiophene-2-carbaldehyde (*cis*-12 and *trans*-12)

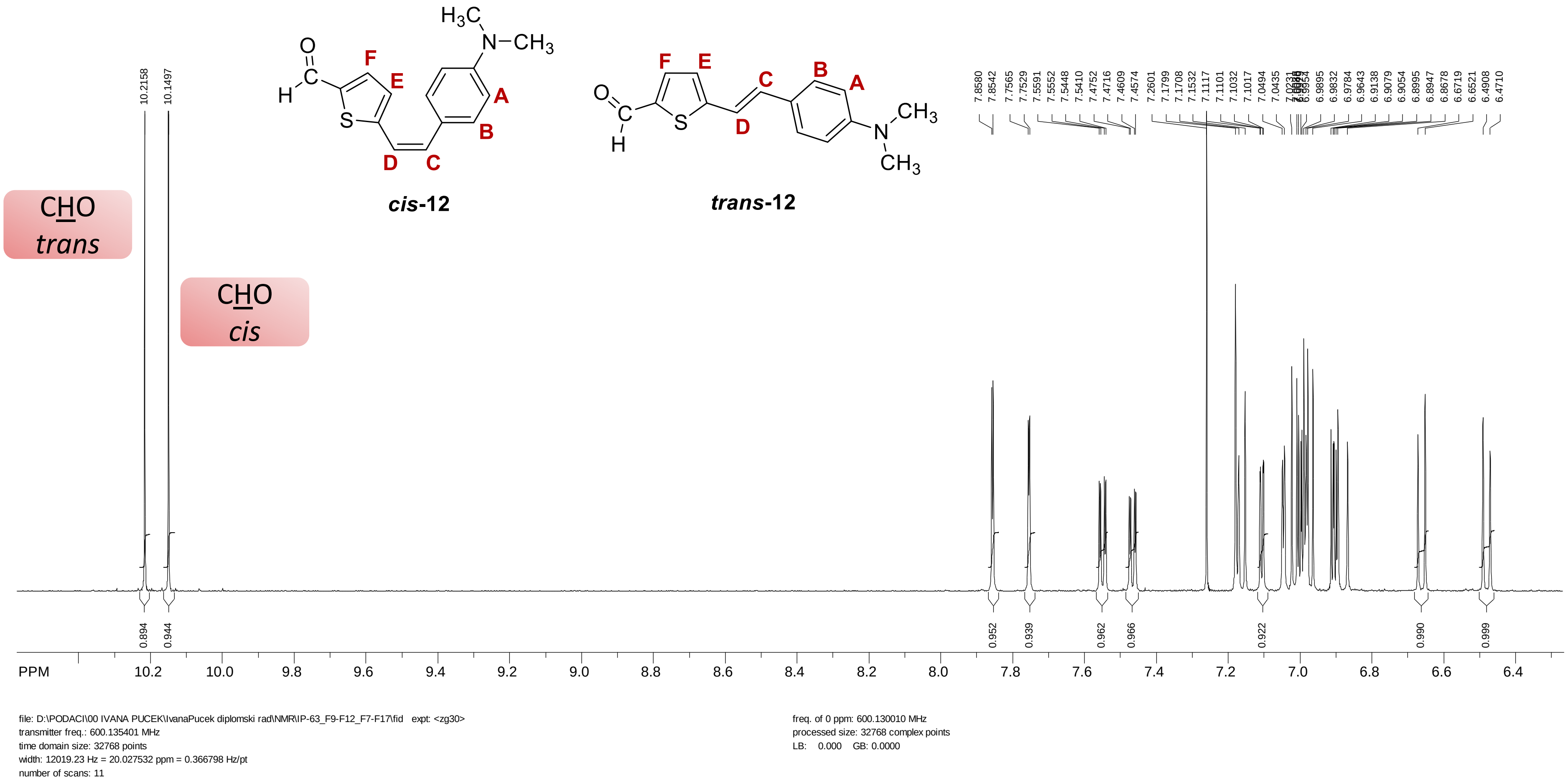

Mass spectra and HRMS analysis of the mixture of geometrical isomers of 5-(4-(dimethylamino)styryl)thiophene-2-carbaldehyde (*cis*-12 and *trans*-12)

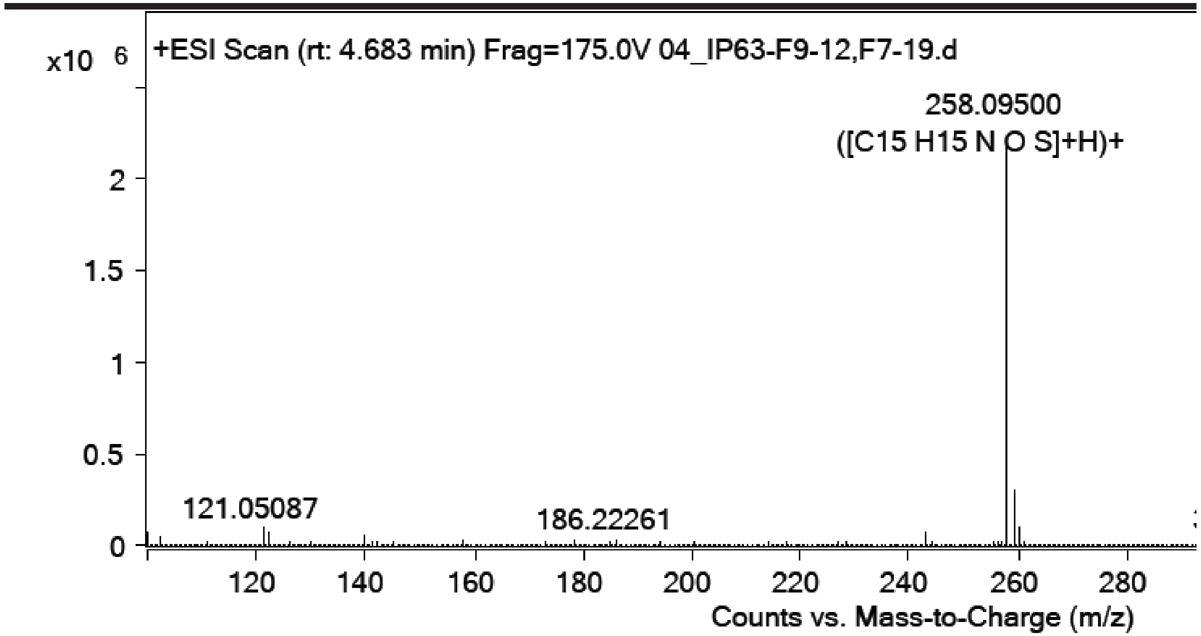

Formula Calculator Results

Formula	Best	Mass	Tgt Mass	Diff (ppm)
C15 H15 N O S	True	257.08787	257.08743	-1.67

^{1}H NMR spectrum (600 MHz, $CDCl_3$) of *trans,anti*-5-(4-methylstyryl)thiophene-2-carbaldehyde oxime (*trans,anti*-13)

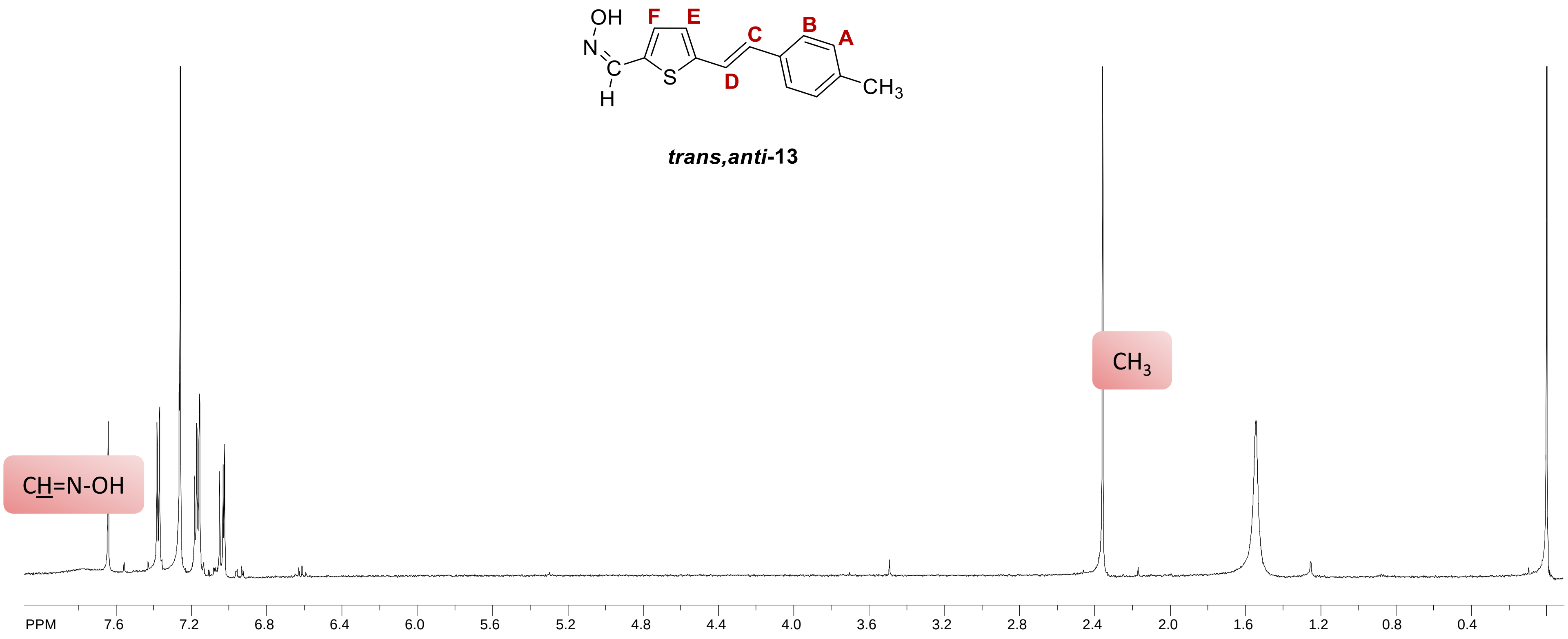

file: D:\PODACI\00 IVANA PUCEK\IvanaPucek diplomski rad\NMR\IP-51_F8-F18_F54-F55\fid expt: <zg30>
transmitter freq.: 600.135401 MHz
time domain size: 32768 points
width: 12019.23 Hz = 20.027532 ppm = 0.366798 Hz/pt
number of scans: 54

freq. of 0 ppm: 600.130012 MHz
processed size: 32768 complex points
LB: 0.000 GB: 0.0000

A part of the ^{1}H NMR spectrum (600 MHz, $CDCl_3$) of *trans,anti*-5-(4-methylstyryl)thiophene-2-carbaldehyde oxime (*trans,anti*-13)

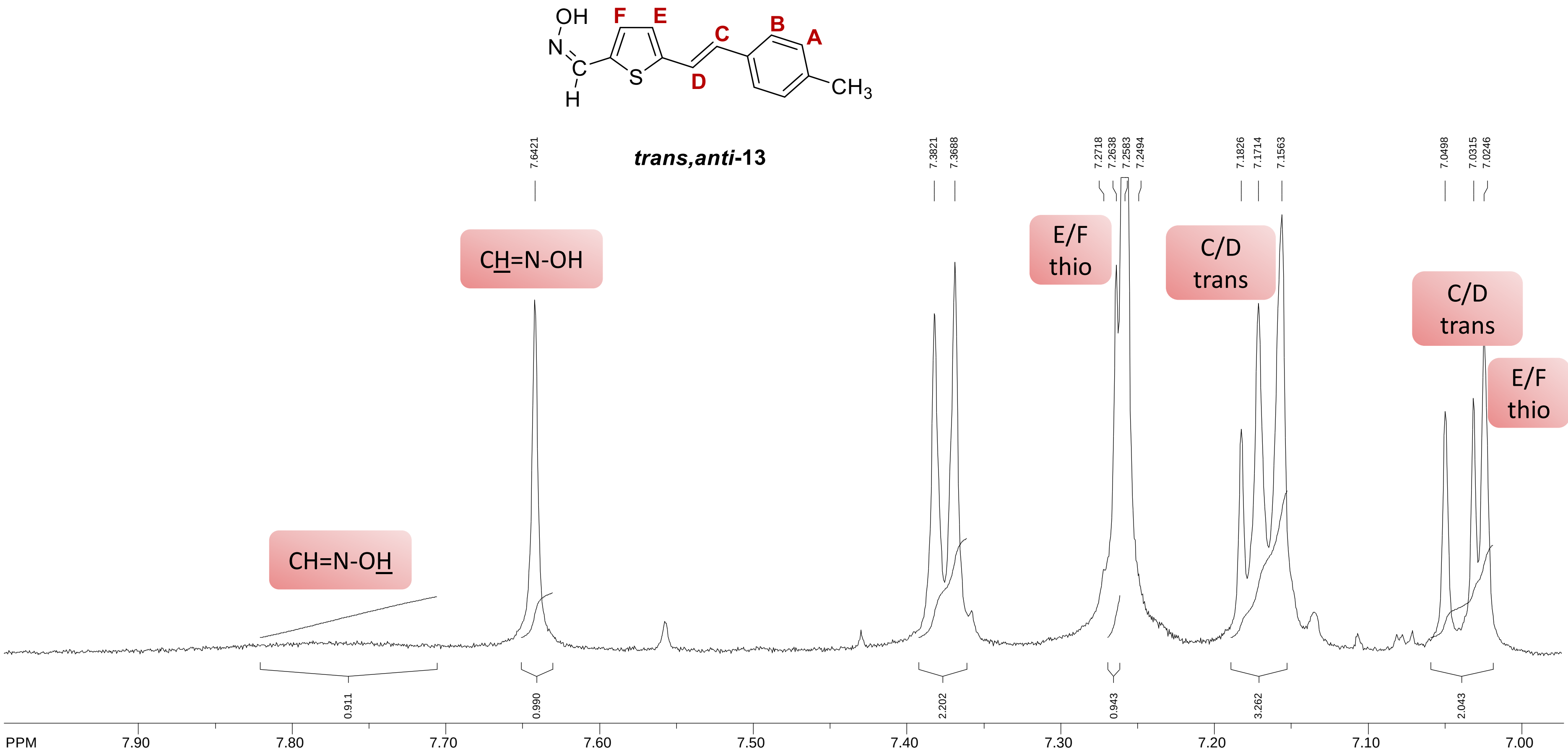

file: D:\PODACI\00 IVANA PUCEK\IvanaPucek diplomski rad\NMR\IP-51_F8-F18_F54-F55\fid expt: <zg30>
transmitter freq.: 600.135401 MHz
time domain size: 32768 points
width: 12019.23 Hz = 20.027532 ppm = 0.366798 Hz/pt
number of scans: 54

freq. of 0 ppm: 600.130012 MHz
processed size: 32768 complex points
LB: 0.000 GB: 0.0000

^{13}C NMR spectrum (150 MHz, $CDCl_3$) of *trans,anti*-5-(4-methylstyryl)thiophene-2-carbaldehyde oxime (*trans,anti*-13)

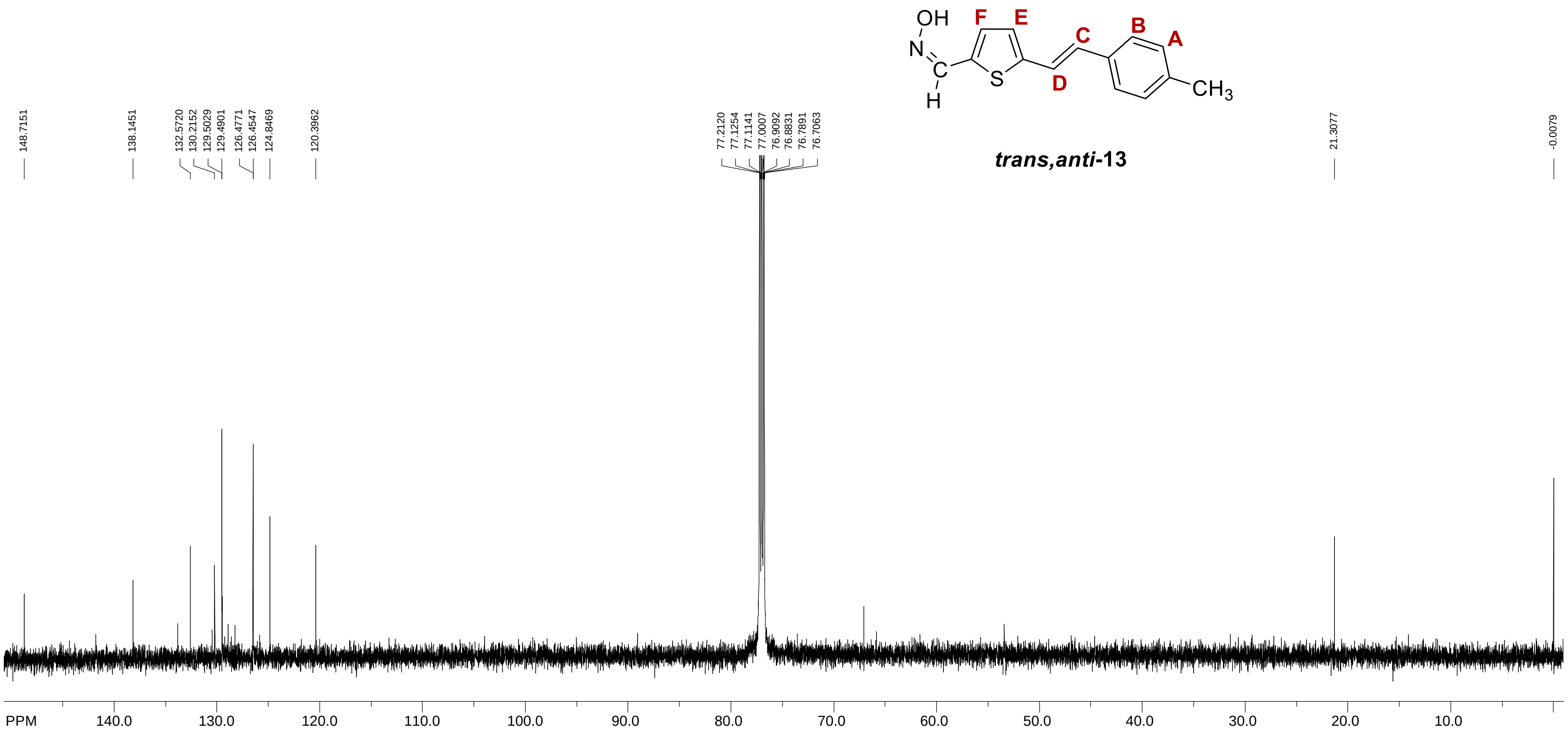

file: D:\PODACI\00 IVANA PUCEK\IvanaPucek diplomski rad\NMR\IP-51_F8-F18_F54-F55 sve\4\fid expt: <zgpg30>
transmitter freq.: 150.917899 MHz
time domain size: 65536 points
width: 35971.22 Hz = 238.349615 ppm = 0.548877 Hz/pt
number of scans: 65536

freq. of 0 ppm: 150.902809 MHz
processed size: 32768 complex points
LB: 0.000 GB: 0.0000

COSY spectrum of *trans,anti*-5-(4-methylstyryl)thiophene-2-carbaldehyde oxime (*trans,anti*-13)

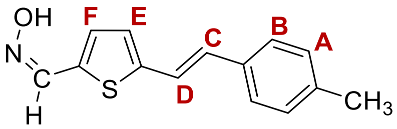

***trans,anti*-13**

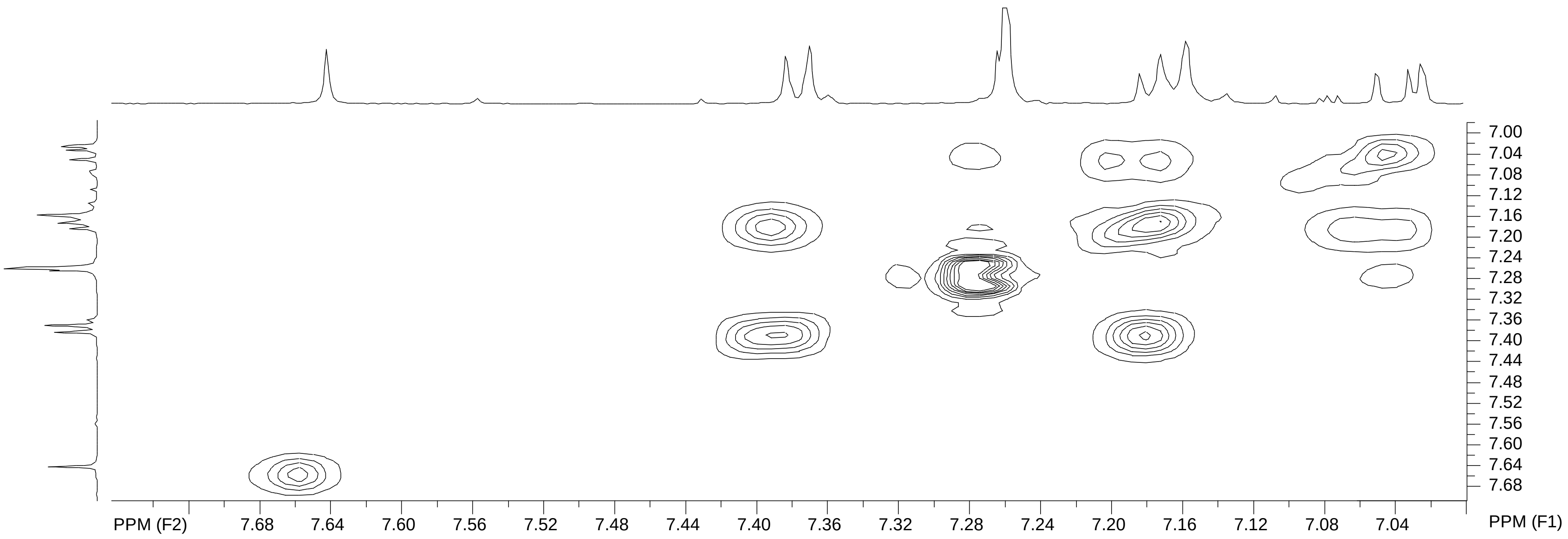

file: D:\PODACI\00 IVANA PUCEK\IvanaPucek diplomski rad\NMR\IP-51_F8-F18_F54-F55 sve\2\ser expt: <cosygpqf>
transmitter freq.: 600.133901 MHz
time domain size: 2048 by 512 points
width: 9615.38 Hz = 16.022065 ppm = 4.695012 Hz/pt
number of scans: 8

F2: freq. of 0 ppm: 600.130000 MHz
processed size: 1024 complex points
window function: Sine
shift: 0.0 degrees

F1: freq. of 0 ppm: 600.130000 MHz
processed size: 1024 complex points
window function: Sine
shift: 0.0 degrees

HSQC spectrum of *trans,anti*-5-(4-methylstyryl)thiophene-2-carbaldehyde oxime (*trans,anti*-13)

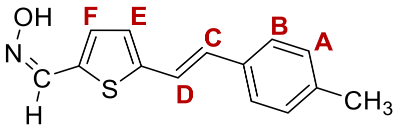

***trans,anti*-13**

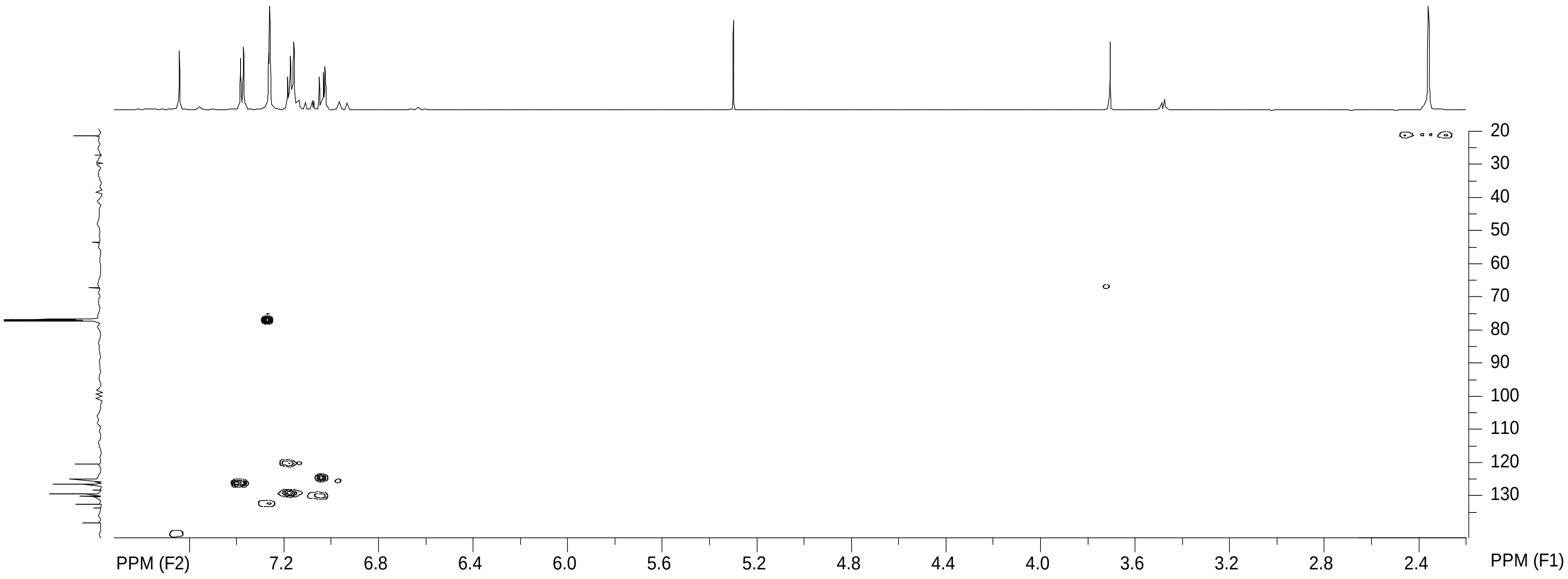

file: D:\PODACI\00 IVANA PUCEK\IvanaPucek diplomski rad\NMR\IP-51_F8-F18_F54-F55 sve\3\ser expt: <inv4gpqf>
transmitter freq.: 600.133901 MHz
time domain size: 2048 by 256 points
width: 9615.38 Hz = 16.022065 ppm = 4.695012 Hz/pt
number of scans: 128

F2: freq. of 0 ppm: 600.130000 MHz
processed size: 1024 complex points
window function: Sine
shift: 0.0 degrees

F1: freq. of 0 ppm: 150.902809 MHz
processed size: 1024 complex points
window function: Sine
shift: 0.0 degrees

^{1}H NMR spectrum (600 MHz, $CDCl_3$) of *trans,syn*-5-(4-methylstyryl)thiophene-2-carbaldehyde oxime (*trans,syn*-13)

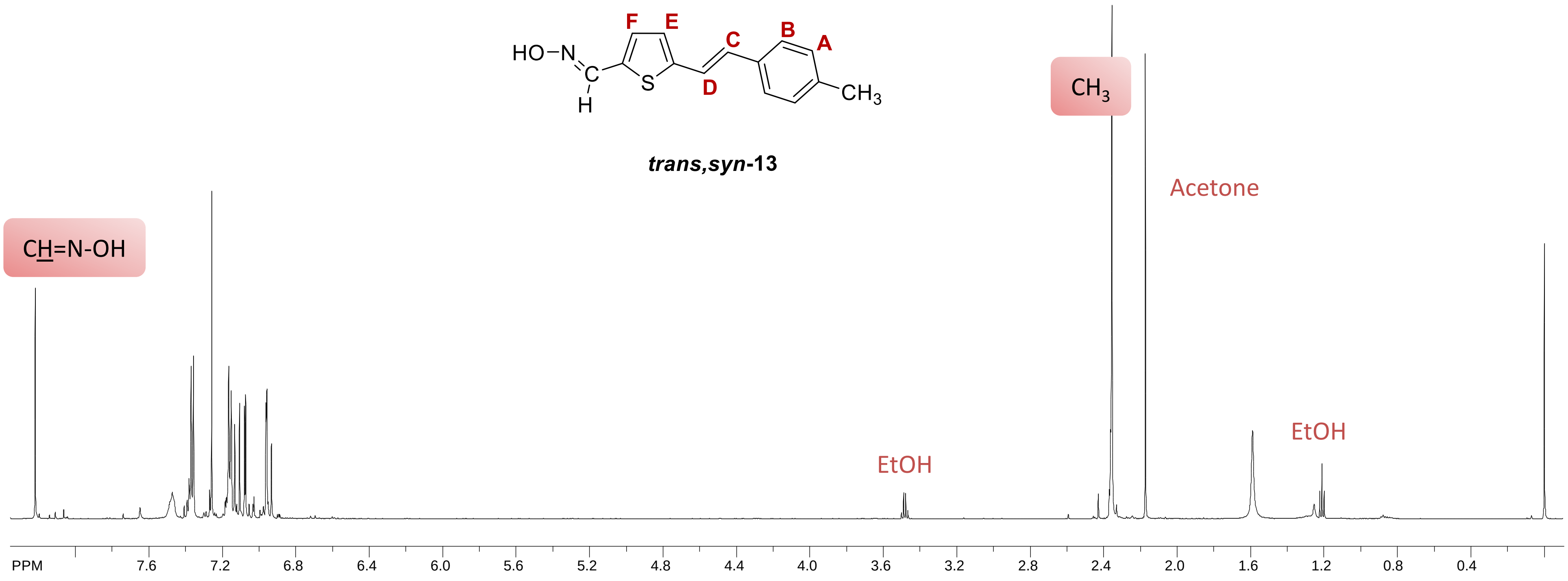

file: D:\PODACI\00 IVANA PUCEK\IvanaPucek diplomski rad\NMR\IP-51_F8-F18_F51-F54_B\fid expt: <zg30>
transmitter freq.: 600.135401 MHz
time domain size: 32768 points
width: 12019.23 Hz = 20.027532 ppm = 0.366798 Hz/pt
number of scans: 28

freq. of 0 ppm: 600.130011 MHz
processed size: 32768 complex points
LB: 0.000 GB: 0.0000

A part of the ^{1}H NMR spectrum (600 MHz, $CDCl_3$) of *trans,syn*-5-(4-methylstyryl)thiophene-2-carbaldehyde oxime (*trans,syn*-13)

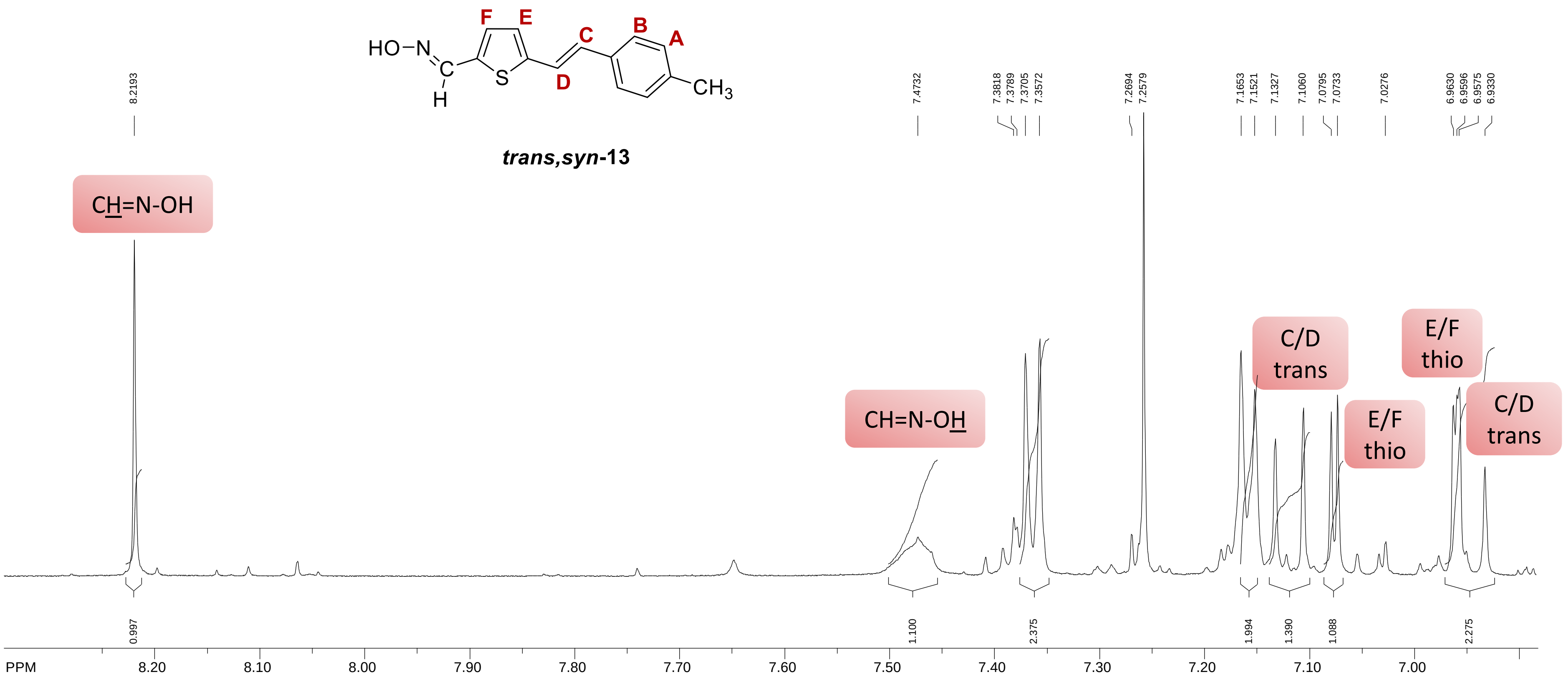

file: D:\PODACI\00 IVANA PUCEK\IvanaPucek diplomski rad\NMR\IP-51_F8-F18_F51-F54_B\fid expt: <zg30>
transmitter freq.: 600.135401 MHz
time domain size: 32768 points
width: 12019.23 Hz = 20.027532 ppm = 0.366798 Hz/pt
number of scans: 28

freq. of 0 ppm: 600.130011 MHz
processed size: 32768 complex points
LB: 0.000 GB: 0.0000

^{13}C NMR spectrum (150 MHz, $CDCl_3$) of *trans,syn*-5-(4-methylstyryl)thiophene-2-carbaldehyde oxime (*trans,syn*-13)

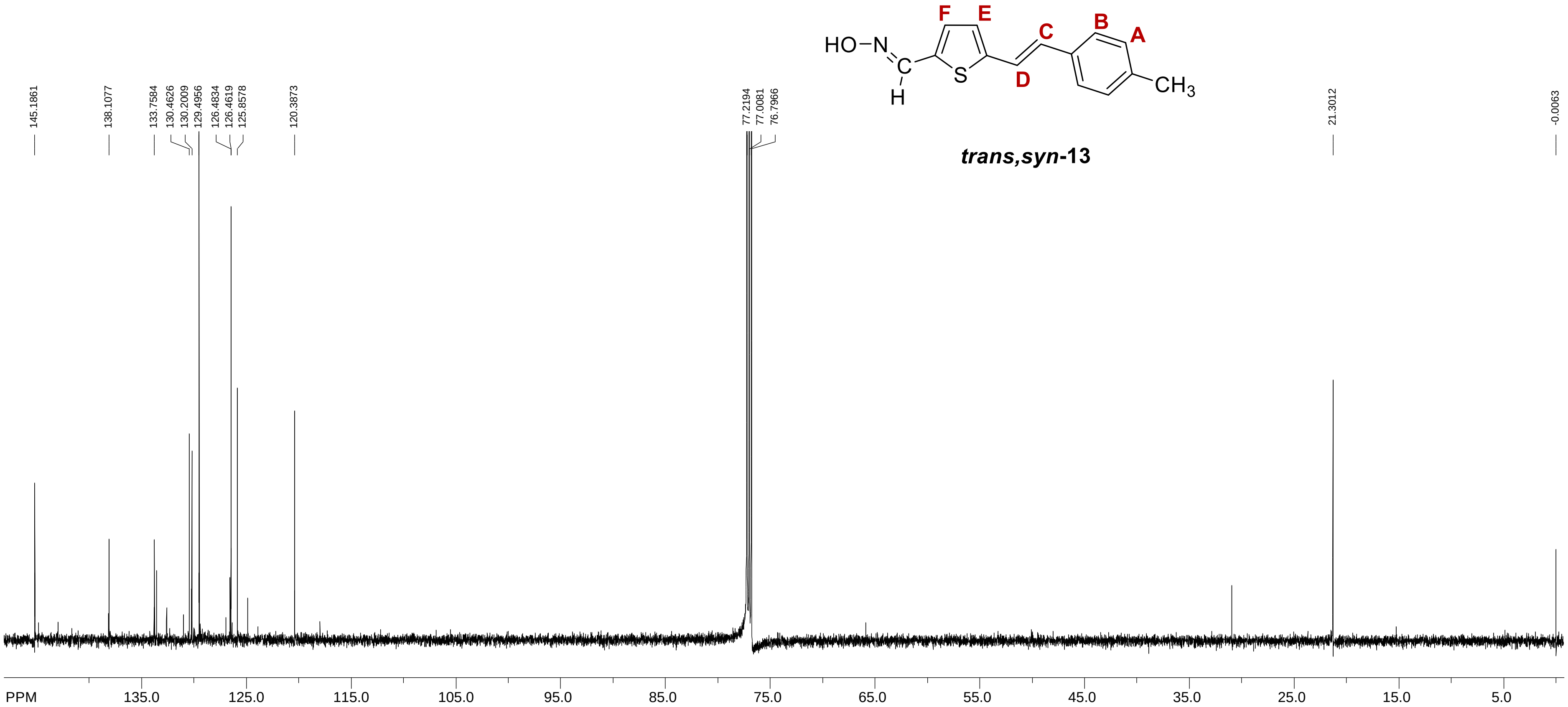

file: D:\PODACI\00 IVANA PUCEK\IvanaPucek diplomski rad\NMR\IP-51_F8-F18_F51-F53_B sve\4\fid expt: <zgpg30>
transmitter freq.: 150.917899 MHz
time domain size: 65536 points
width: 35971.22 Hz = 238.349615 ppm = 0.548877 Hz/pt
number of scans: 27043

freq. of 0 ppm: 150.902809 MHz
processed size: 32768 complex points
LB: 0.000 GB: 0.0000

COSY spectrum of *trans,syn*-5-(4-methylstyryl)thiophene-2-carbaldehyde oxime (*trans,syn*-13)

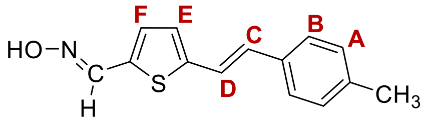

***trans,syn*-13**

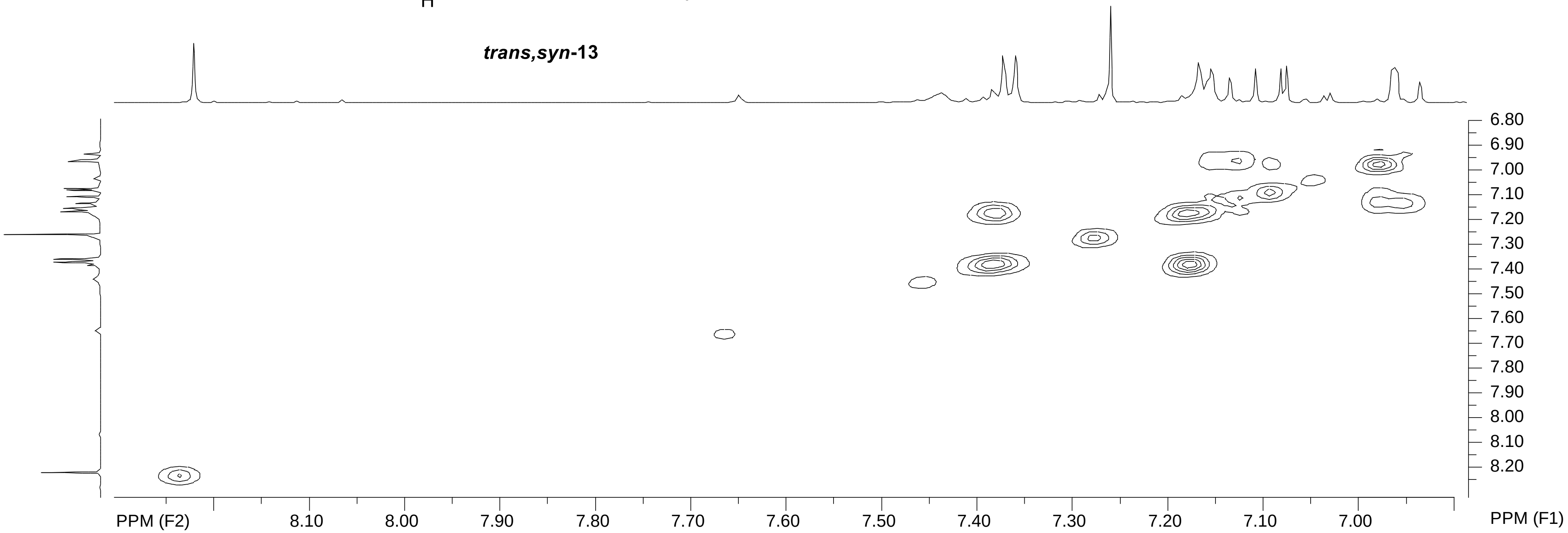

file: D:\PODACI\00 IVANA PUCEK\IvanaPucek diplomski rad\NMR\IP-51_F8-F18_F51-F53_B sve\2\ser expt: <cosygpqf>
transmitter freq.: 600.133901 MHz
time domain size: 2048 by 512 points
width: 9615.38 Hz = 16.022065 ppm = 4.695012 Hz/pt
number of scans: 4

F2: freq. of 0 ppm: 600.130000 MHz
processed size: 1024 complex points
window function: Sine
shift: 0.0 degrees

F1: freq. of 0 ppm: 600.130000 MHz
processed size: 1024 complex points
window function: Sine
shift: 0.0 degrees

HSQC spectrum of *trans,syn*-5-(4-methylstyryl)thiophene-2-carbaldehyde oxime (*trans,syn*-13)

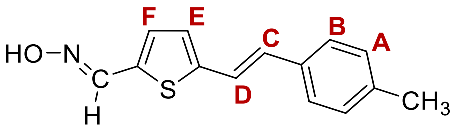

***trans,syn*-13**

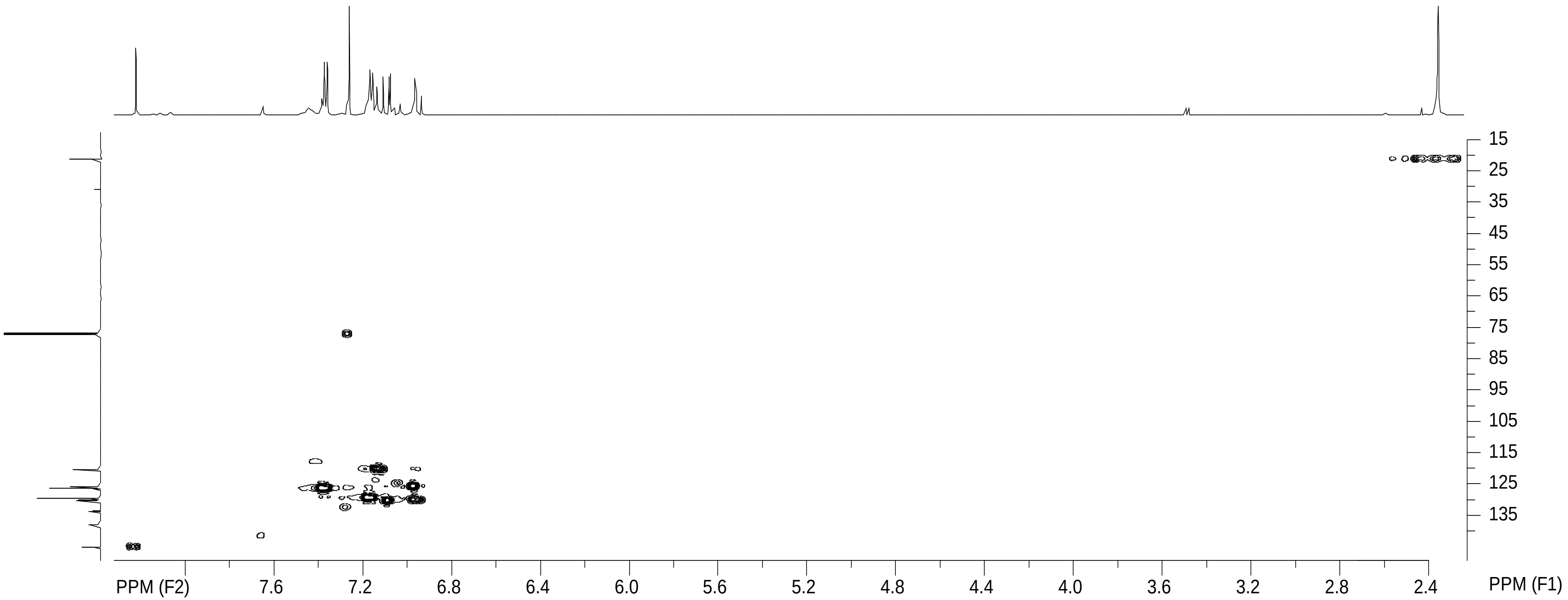

file: D:\PODACI\00 IVANA PUCEK\IvanaPucek diplomski rad\NMR\IP-51_F8-F18_F51-F53_B sve\3\ser expt: <inv4gpqf>
transmitter freq.: 600.133901 MHz
time domain size: 2048 by 256 points
width: 9615.38 Hz = 16.022065 ppm = 4.695012 Hz/pt
number of scans: 64

F2: freq. of 0 ppm: 600.130000 MHz
processed size: 1024 complex points
window function: Sine
shift: 0.0 degrees

F1: freq. of 0 ppm: 150.902809 MHz
processed size: 1024 complex points
window function: Sine
shift: 0.0 degrees

^{1}H NMR spectrum (600 MHz, $CDCl_3$) of *cis,syn*-5-(4-methylstyryl)thiophene-2-carbaldehyde oxime (*cis,syn*-13) with traces of *trans,syn*-**13**

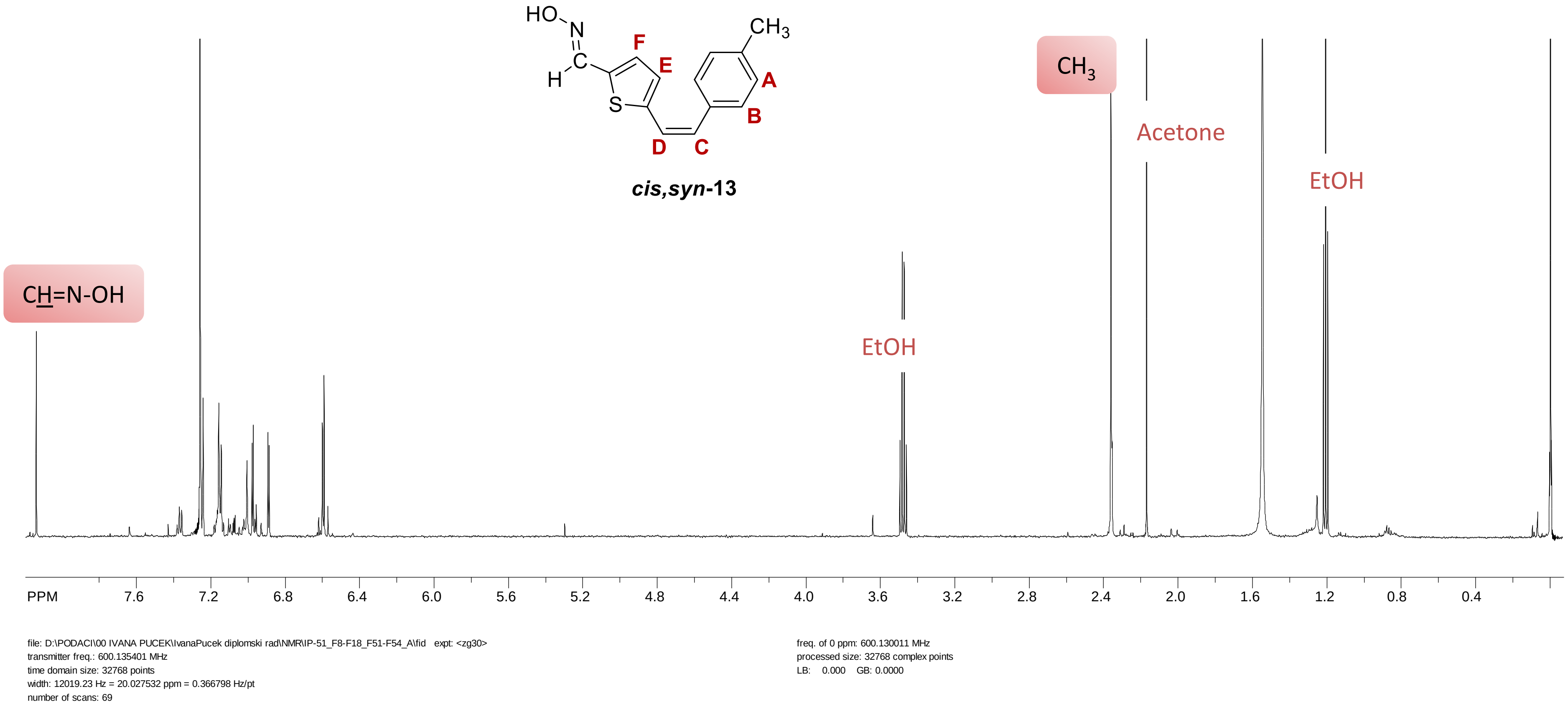

A part of the ¹H NMR spectrum (600 MHz, $CDCl_3$) of *cis,syn*-5-(4-methylstyryl)thiophene-2-carbaldehyde oxime (*cis,syn*-13) with traces of *trans,syn*-**13**

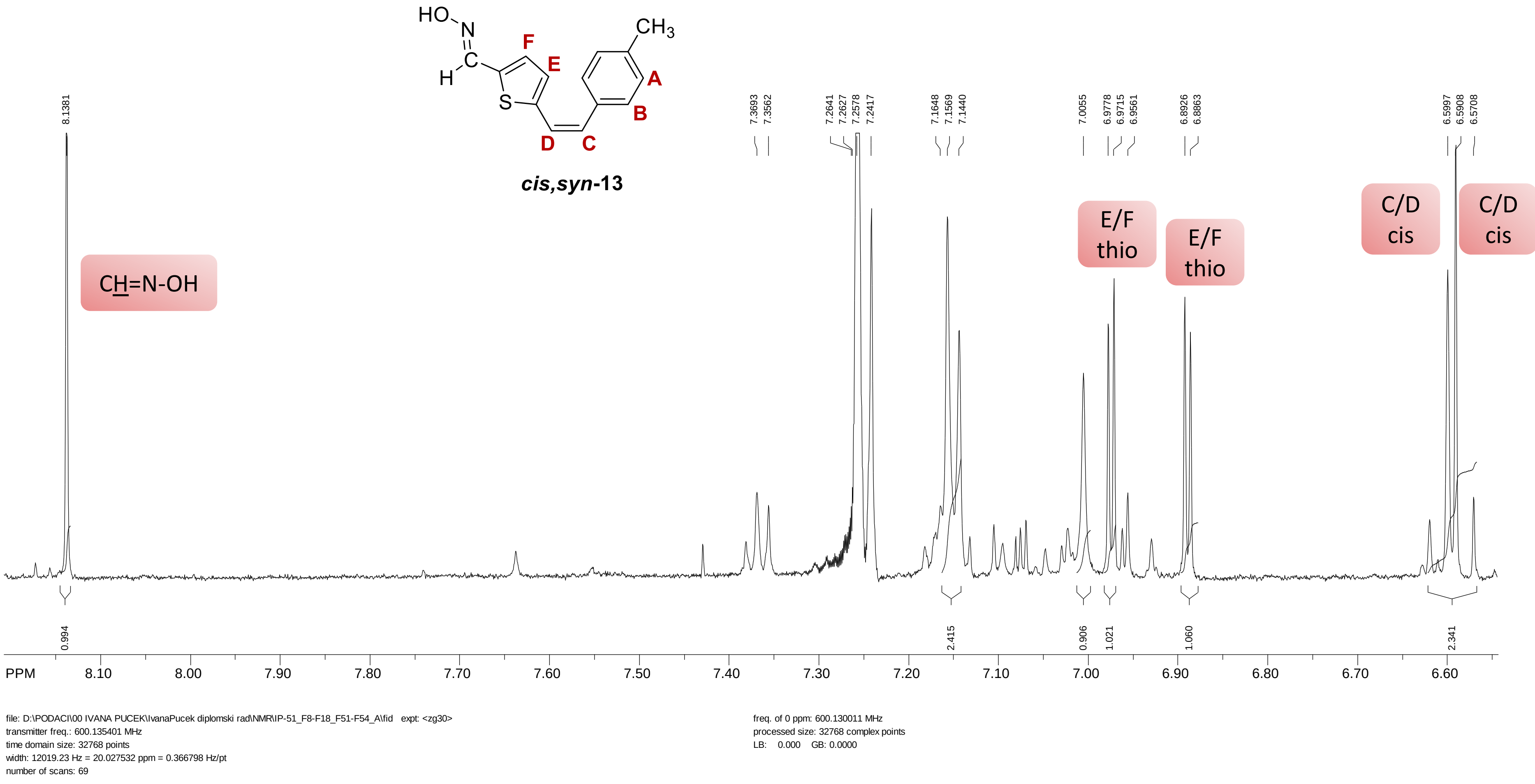

Mass spectra and HRMS analysis of the mixture of geometrical isomers of 5-(4-methylstyryl)thiophene-2-carbaldehyde oxime (13)

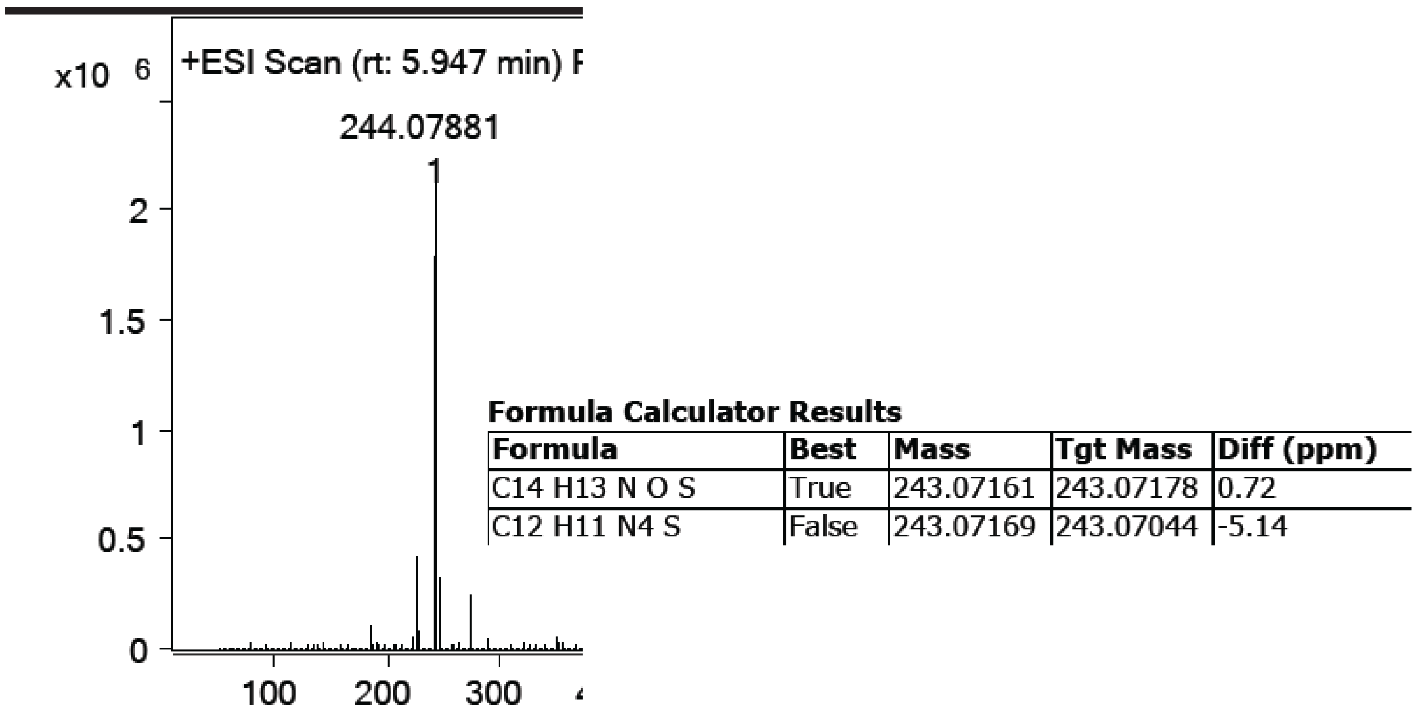

Formula Calculator Results

Formula	Best	Mass	Tgt Mass	Diff (ppm)
C14 H13 N O S	True	243.07161	243.07178	0.72
C12 H11 N4 S	False	243.07169	243.07044	-5.14

^{1}H NMR spectrum (600 MHz, $CDCl_3$ + CD_3OD) of *trans,anti*-5-(4-methoxystyryl)thiophene-2-carbaldehyde oxime (*trans,anti*-14)

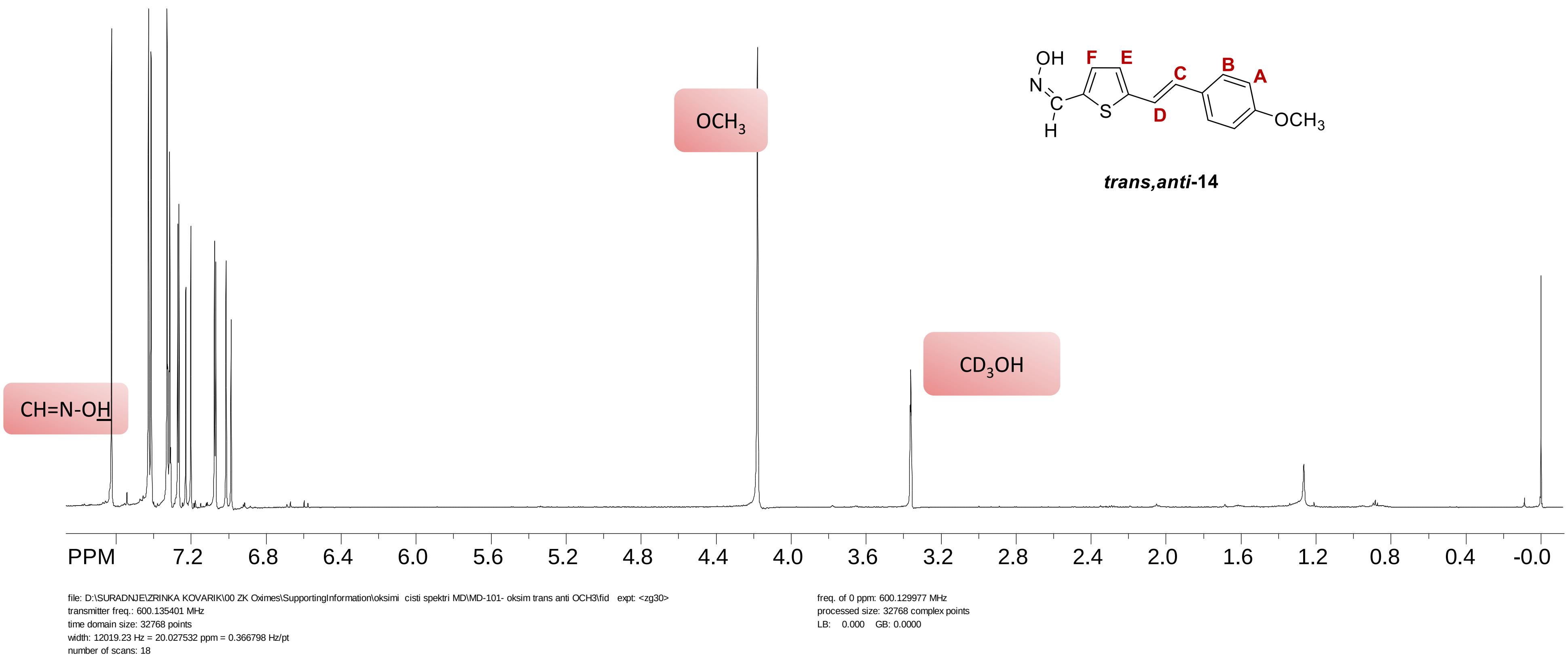

A part of the ^{1}H NMR spectrum (600 MHz, $CDCl_3$ + CD_3OD) of *trans,anti*-5-(4-methoxystyryl)thiophene-2-carbaldehyde oxime (*trans,anti*-14)

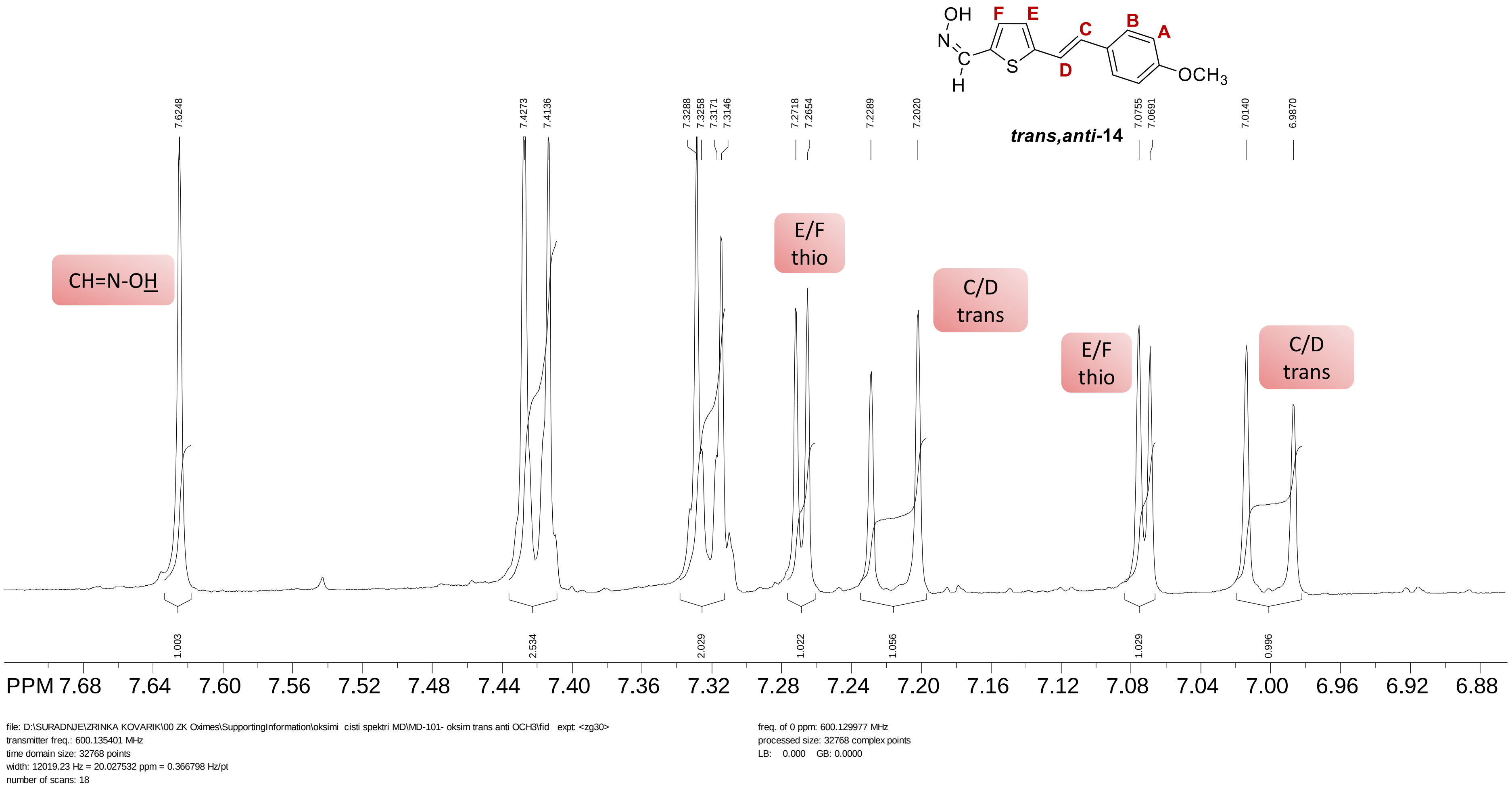

^{13}C NMR spectrum (150 MHz, $CDCl_3$ + CD_3OD) of *trans,anti*-5-(4-methoxystyryl)thiophene-2-carbaldehyde oxime (*trans,anti*-14)

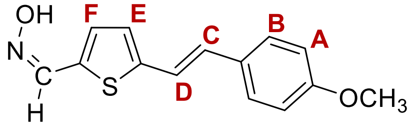

***trans,anti*-14**

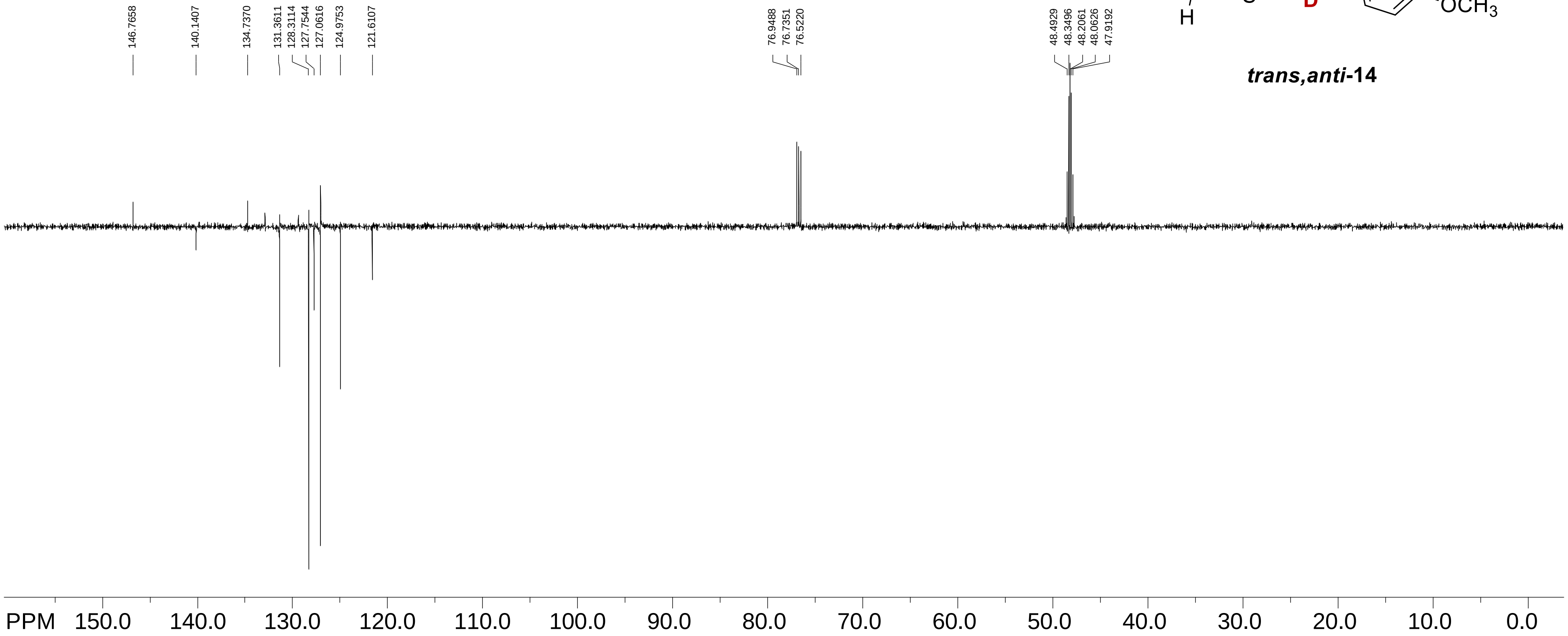

file: D:\SURADNJE\ZRINKA KOVARIK\00 ZK Oximes\SupportingInformation\oksimi cisti spektri MD\MD-101 APT- oksim trans anti OCH3\fid expt: <jmod>
transmitter freq.: 150.917899 MHz
time domain size: 65536 points
width: 39370.08 Hz = 260.870838 ppm = 0.600740 Hz/pt
number of scans: 1110

freq. of 0 ppm: 150.902884 MHz
processed size: 32768 complex points
LB: 0.000 GB: 0.0000

^{1}H NMR spectrum (600 MHz, $CDCl_3$) of *cis,syn*-5-(4-methoxystyryl)thiophene-2-carbaldehyde oxime (*cis,syn*-14)

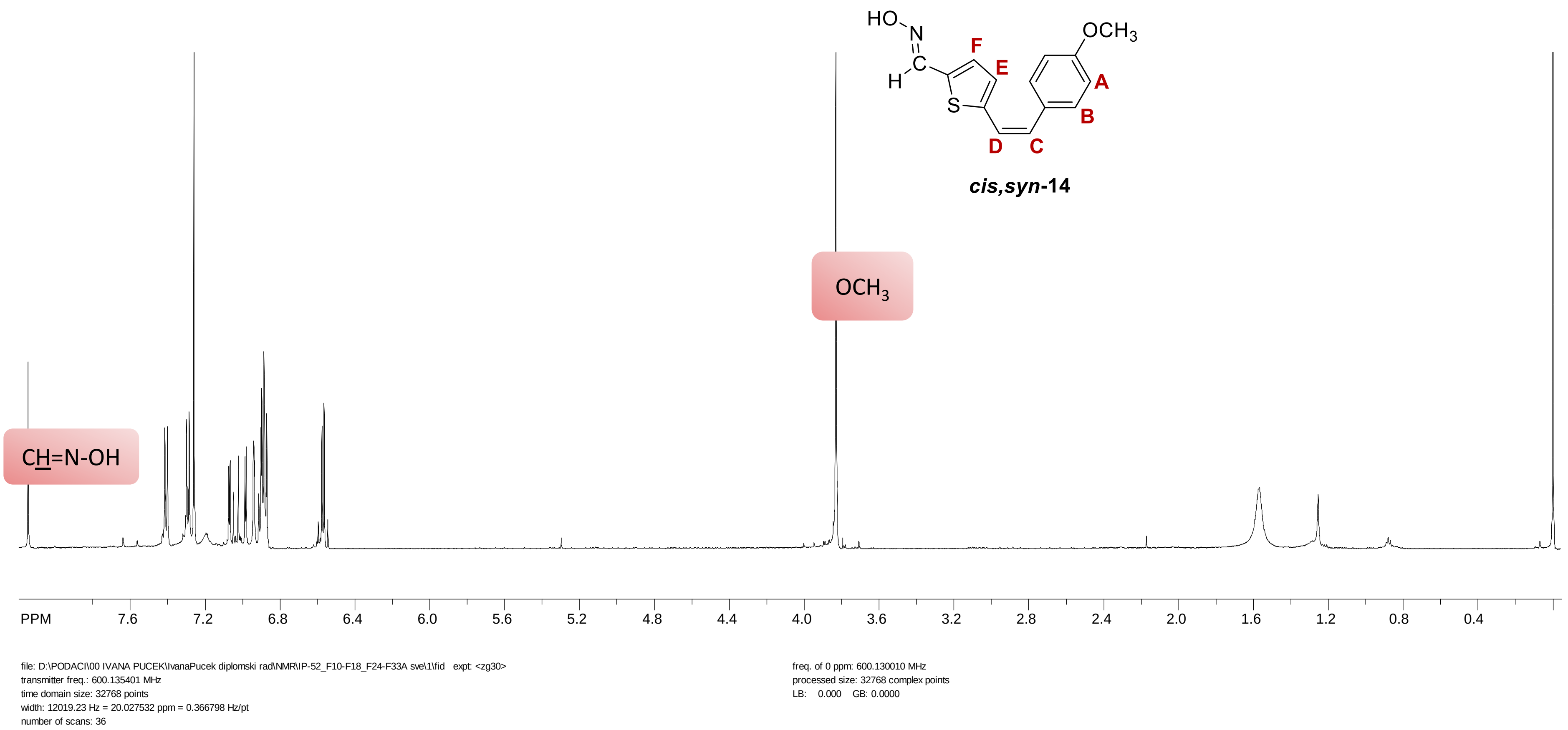

A part of the ^{1}H NMR spectrum (600 MHz, $CDCl_3$) of *cis,syn*-5-(4-methoxystyryl)thiophene-2-carbaldehyde oxime (*cis,syn*-14)

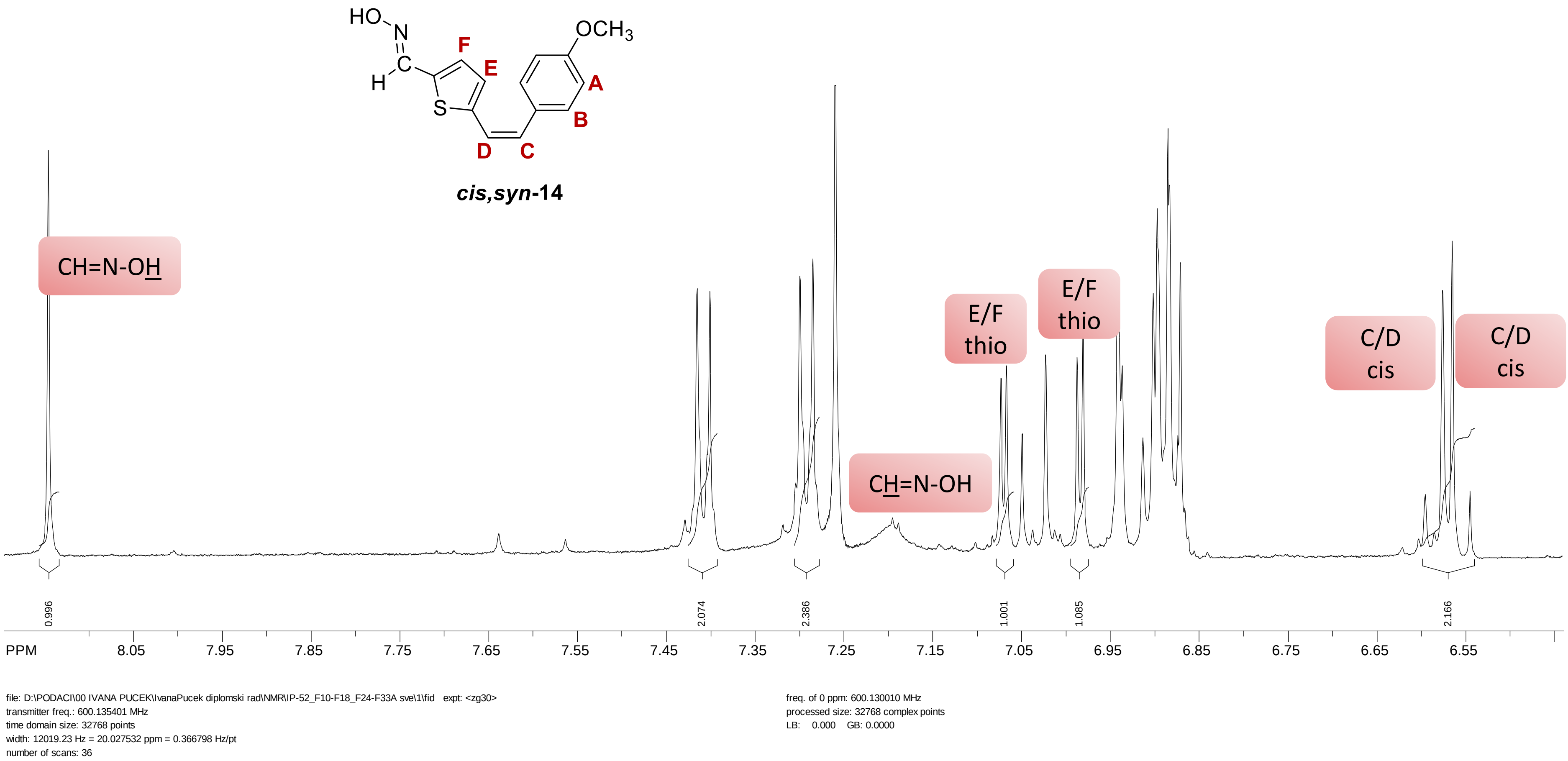

^{13}C NMR spectrum (150 MHz, $CDCl_3$) of *cis,syn*-5-(4-methoxystyryl)thiophene-2-carbaldehyde oxime (*cis,syn*-14)

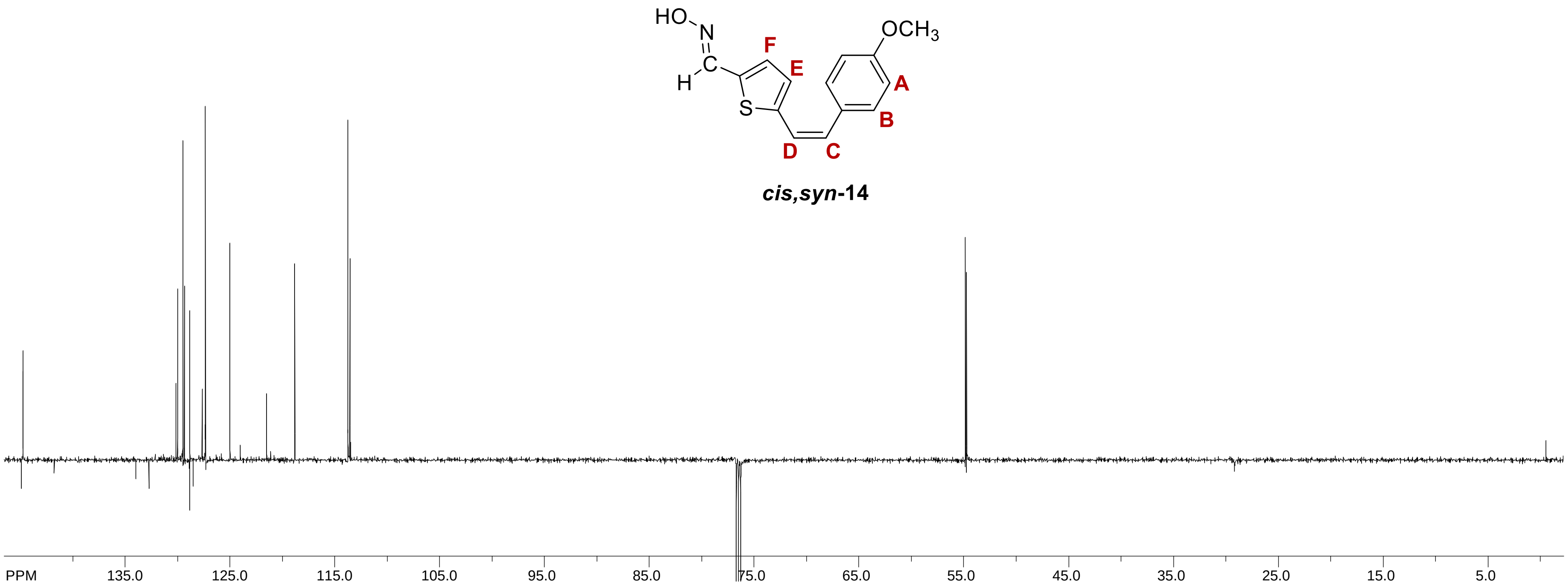

file: D:\PODACI\00 IVANA PUCEK\IvanaPucek diplomski rad\NMR\IP-52_F10-F18_F24-F33A sve\4\fid expt: <jmod>
transmitter freq.: 150.917899 MHz
time domain size: 65536 points
width: 39370.08 Hz = 260.870838 ppm = 0.600740 Hz/pt
number of scans: 64193

freq. of 0 ppm: 150.902884 MHz
processed size: 32768 complex points
LB: 0.000 GB: 0.0000

COSY spectrum of *cis,syn*-5-(4-methoxystyryl)thiophene-2-carbaldehyde oxime (*cis,syn*-14)

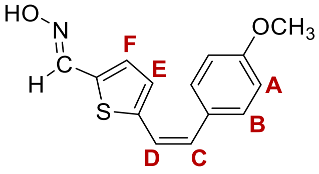

***cis,syn*-14**

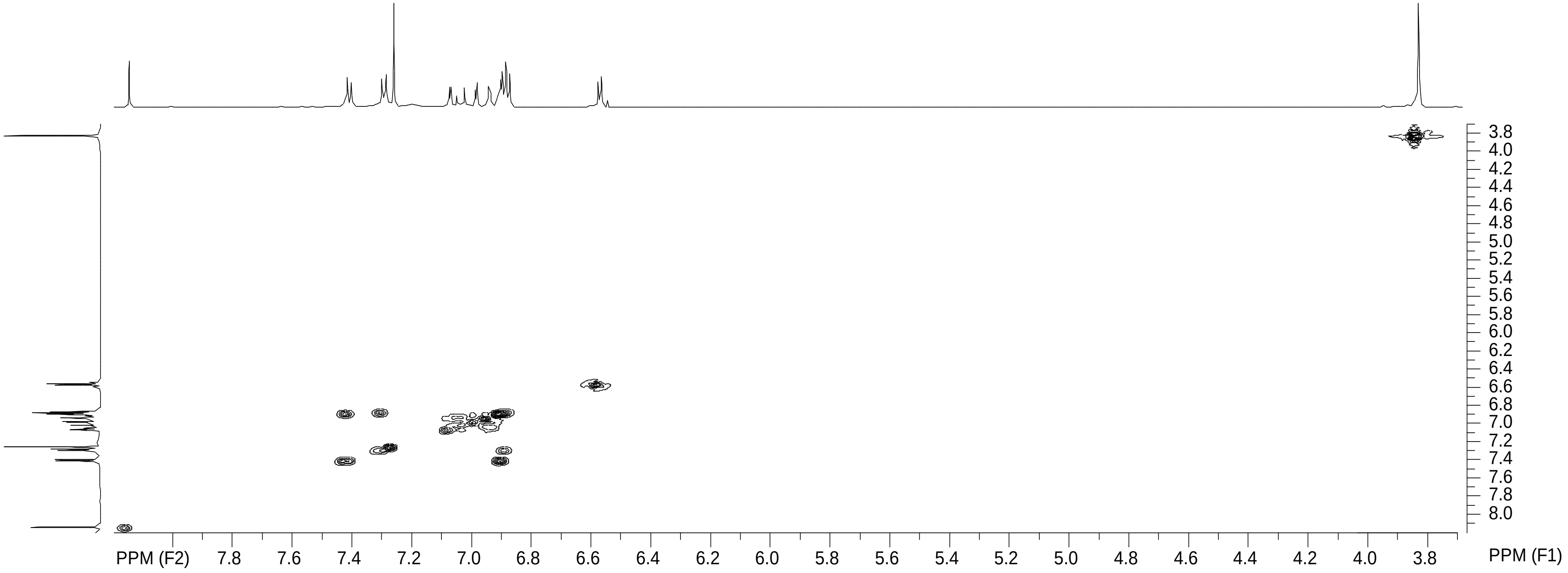

file: D:\PODACI\00 IVANA PUCEK\IvanaPucek diplomski rad\NMR\IP-52_F10-F18_F24-F33A sve\2\ser expt: <cosygpqf>
transmitter freq.: 600.133901 MHz
time domain size: 2048 by 512 points
width: 9615.38 Hz = 16.022065 ppm = 4.695012 Hz/pt
number of scans: 8

F2: freq. of 0 ppm: 600.130000 MHz
processed size: 1024 complex points
window function: Sine
shift: 0.0 degrees

F1: freq. of 0 ppm: 600.130000 MHz
processed size: 1024 complex points
window function: Sine
shift: 0.0 degrees

HSQC spectrum of *cis,syn*-5-(4-methoxystyryl)thiophene-2-carbaldehyde oxime (*cis,syn*-14)

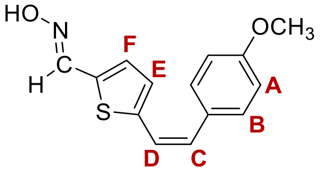

***cis,syn*-14**

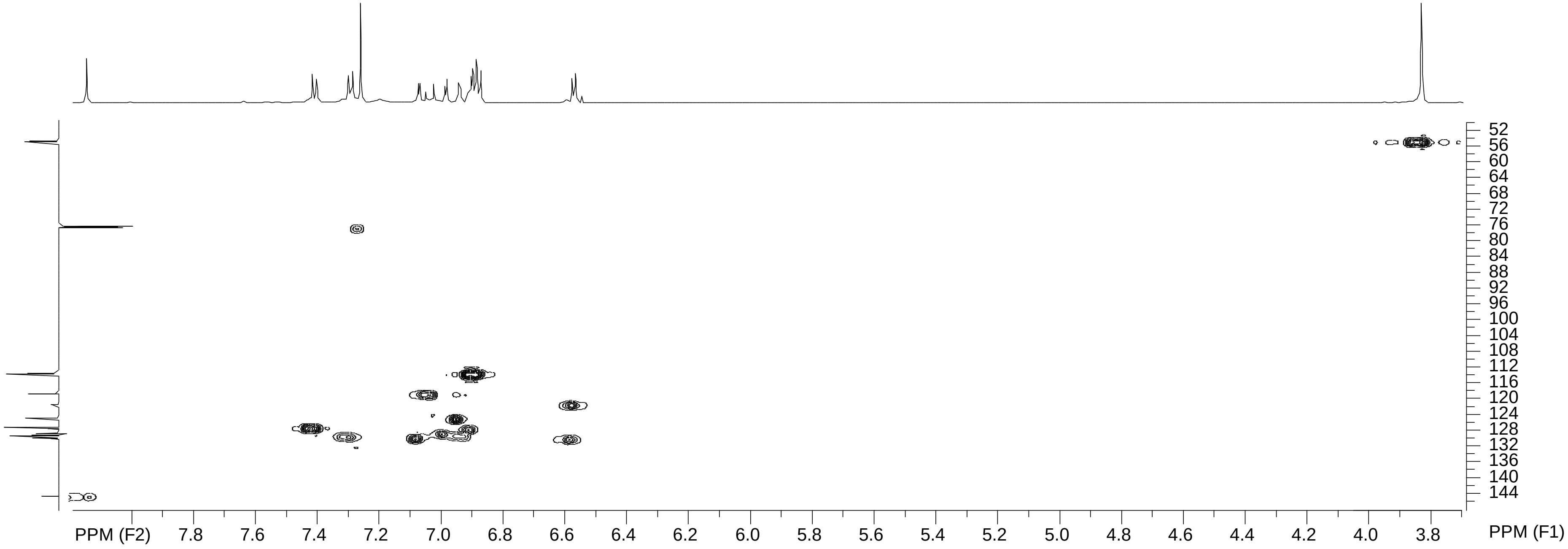

file: D:\PODACI\00 IVANA PUCEK\IvanaPucek diplomski rad\NMR\IP-52_F10-F18_F24-F33A sve\3\ser expt: <inv4gpqf>
transmitter freq.: 600.133901 MHz
time domain size: 2048 by 256 points
width: 9615.38 Hz = 16.022065 ppm = 4.695012 Hz/pt
number of scans: 128

F2: freq. of 0 ppm: 600.130000 MHz
processed size: 1024 complex points
window function: Sine
shift: 0.0 degrees

F1: freq. of 0 ppm: 150.902809 MHz
processed size: 1024 complex points
window function: Sine
shift: 0.0 degrees

^{1}H NMR spectrum (600 MHz, $CDCl_3$) of *trans,syn*-5-(4-methoxystyryl)thiophene-2-carbaldehyde oxime (*trans,syn*-14)

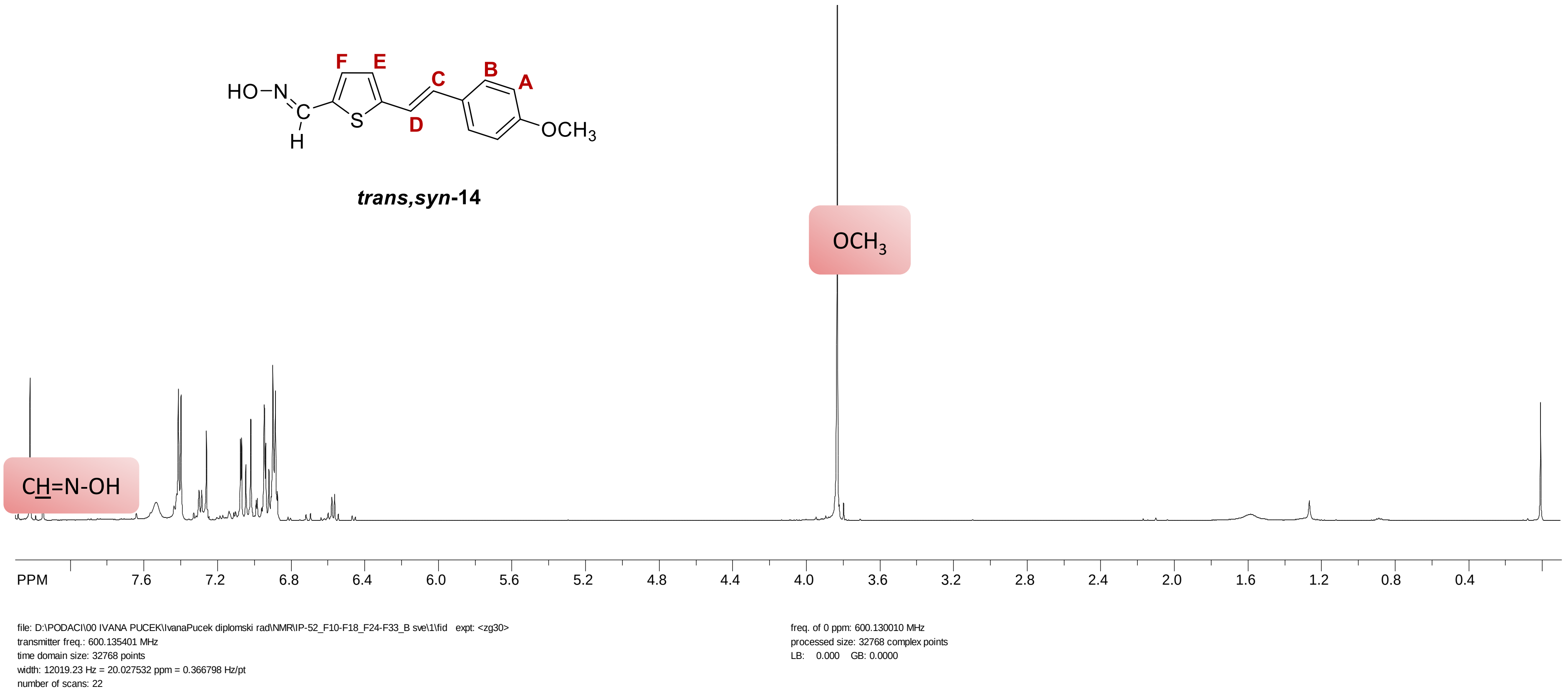

A part of the ^{1}H NMR spectrum (600 MHz, $CDCl_3$) of *trans,syn*-5-(4-methoxystyryl)thiophene-2-carbaldehyde oxime (*trans,syn*-14)

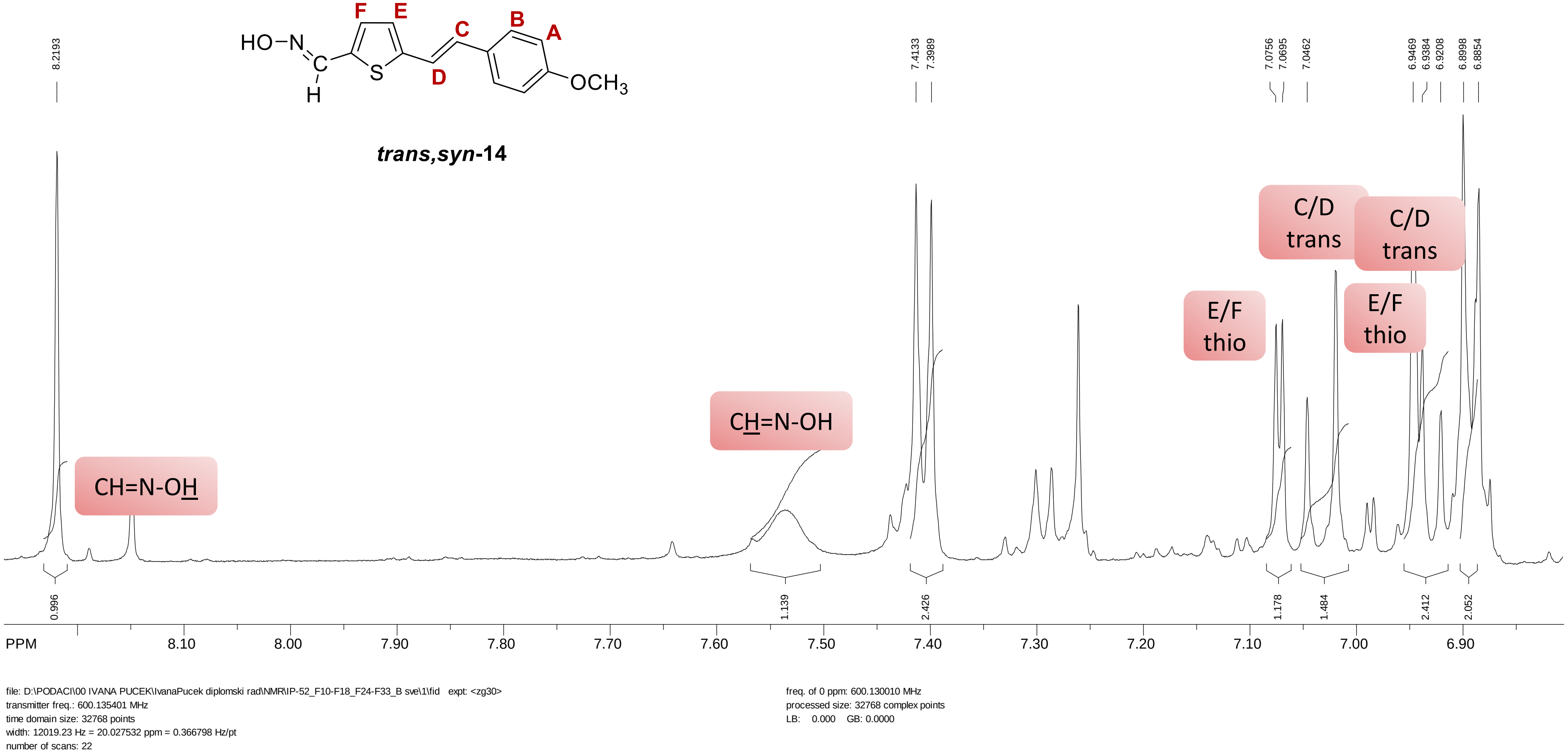

^{13}C NMR spectrum (150 MHz, $CDCl_3$) of *trans,syn*-5-(4-methoxystyryl)thiophene-2-carbaldehyde oxime (*trans,syn*-14)

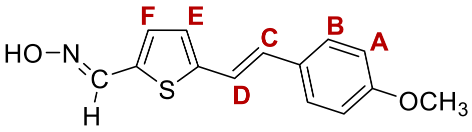

***trans,syn*-14**

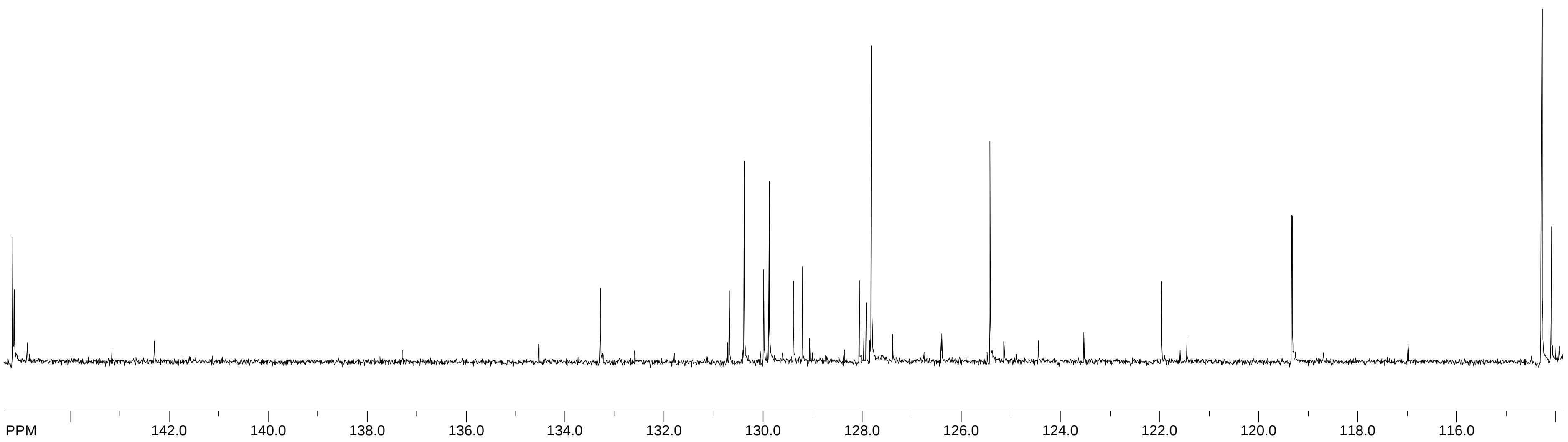

file: D:\PODACI\00 IVANA PUCEK\IvanaPucek diplomski rad\NMR\IP-52_F10-F18_F24-F33_B sve\4\fid expt: <zgpg30>
transmitter freq.: 150.917899 MHz
time domain size: 65536 points
width: 35971.22 Hz = 238.349615 ppm = 0.548877 Hz/pt
number of scans: 20559

freq. of 0 ppm: 150.902809 MHz
processed size: 32768 complex points
LB: 0.000 GB: 0.0000

COSY spectrum of *trans,syn*-5-(4-methoxystyryl)thiophene-2-carbaldehyde oxime (*trans,syn*-14)

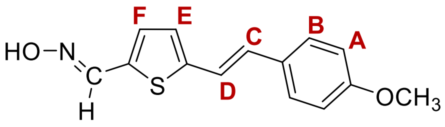

***trans,syn*-14**

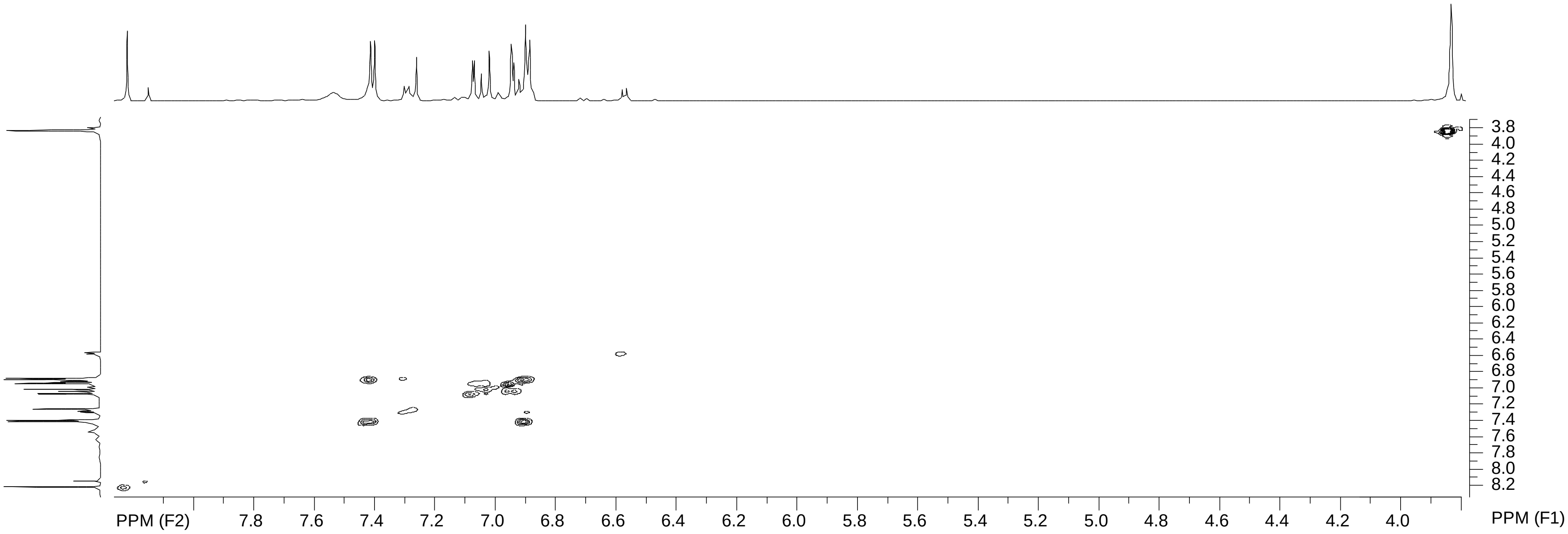

file: D:\PODACI\00 IVANA PUCEK\IvanaPucek diplomski rad\NMR\IP-52_F10-F18_F24-F33_B sve\2\ser expt: <cosygpqf>
transmitter freq.: 600.133901 MHz
time domain size: 2048 by 512 points
width: 9615.38 Hz = 16.022065 ppm = 4.695012 Hz/pt
number of scans: 4

F2: freq. of 0 ppm: 600.130000 MHz
processed size: 1024 complex points
window function: Sine
shift: 0.0 degrees

F1: freq. of 0 ppm: 600.130000 MHz
processed size: 1024 complex points
window function: Sine
shift: 0.0 degrees

HSQC spectrum of *trans,syn*-5-(4-methoxystyryl)thiophene-2-carbaldehyde oxime (*trans,syn*-14)

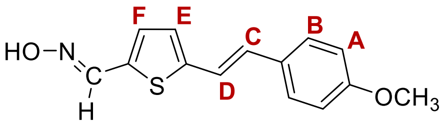

***trans,syn*-14**

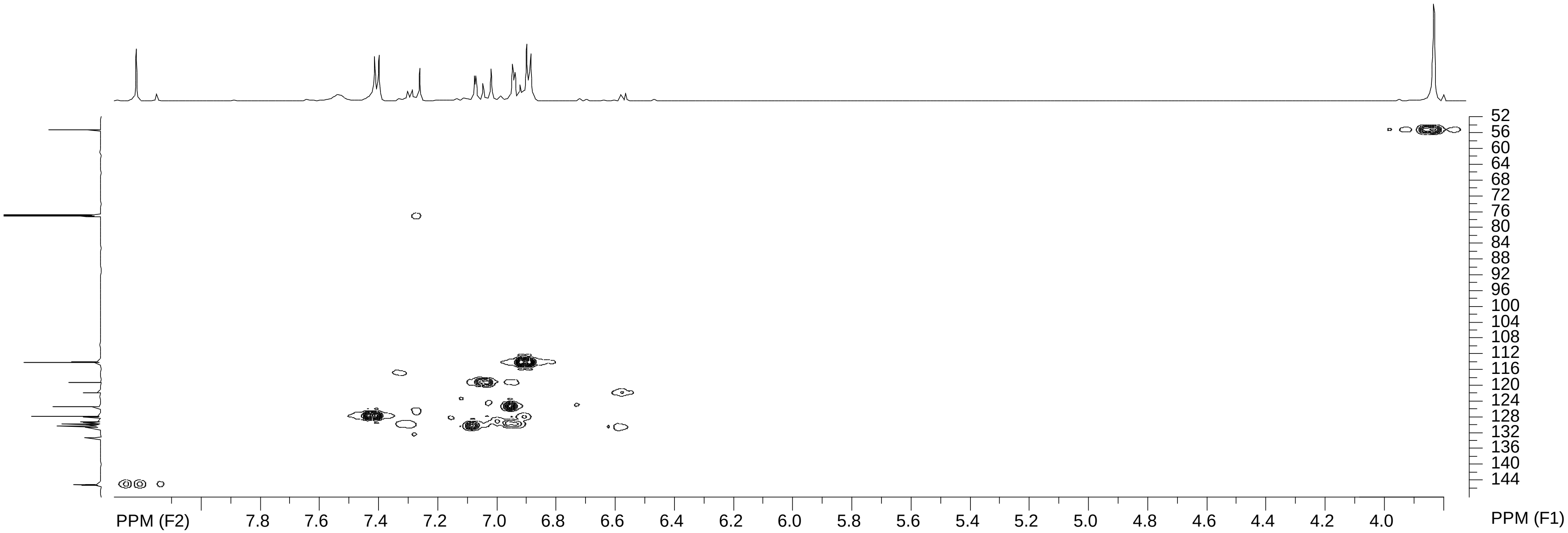

file: D:\PODACI\00 IVANA PUCEK\IvanaPucek diplomski rad\NMR\IP-52_F10-F18_F24-F33_B sve\3\ser expt: <inv4gpqf>
transmitter freq.: 600.133901 MHz
time domain size: 2048 by 256 points
width: 9615.38 Hz = 16.022065 ppm = 4.695012 Hz/pt
number of scans: 64

F2: freq. of 0 ppm: 600.130000 MHz
processed size: 1024 complex points
window function: Sine
shift: 0.0 degrees

F1: freq. of 0 ppm: 150.902809 MHz
processed size: 1024 complex points
window function: Sine
shift: 0.0 degrees

Mass spectra and HRMS analysis of the mixture of geometrical isomers of 5-(4-methoxystyryl)thiophene-2-carbaldehyde oxime (14)

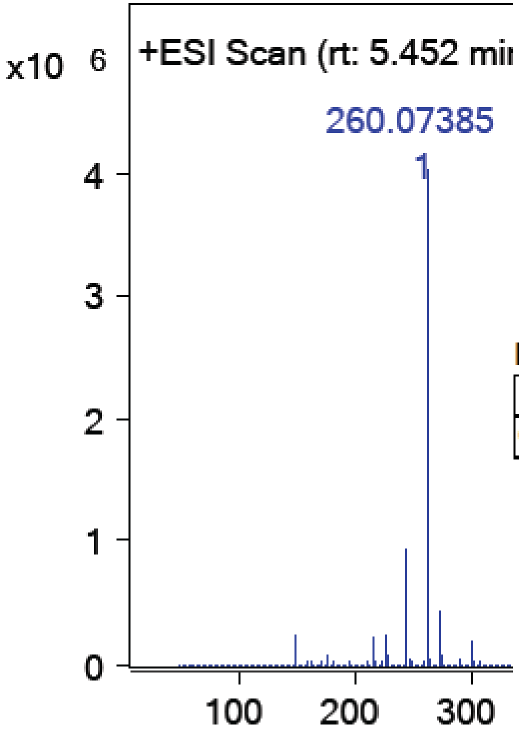

Formula Calculator Results

Formula	Best	Mass	Tgt Mass	Diff (ppm)
C14 H13 N O2 S	True	259.06659	259.0667	0.43

^{1}H NMR spectrum (600 MHz, $CDCl_3$) of *trans,syn*-5-(4-chlorostyryl)thiophene-2-carbaldehyde oxime (*trans,syn*-15)

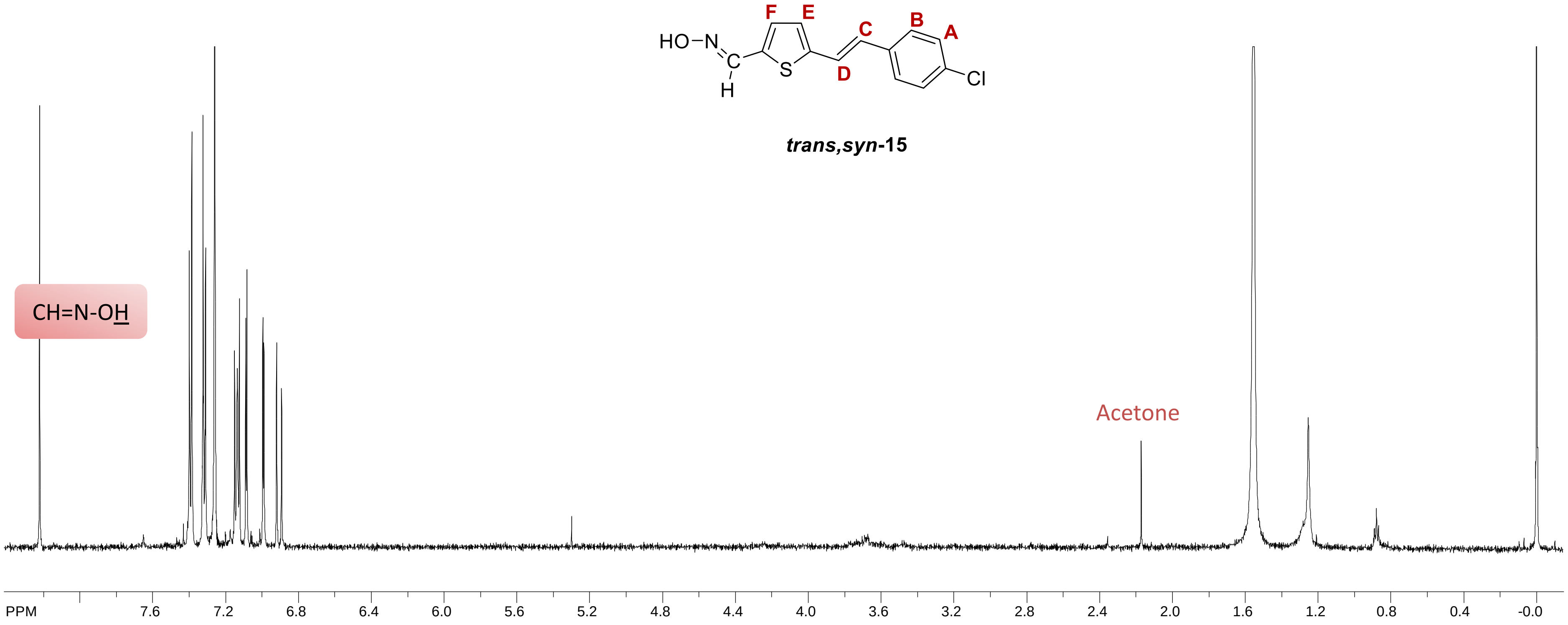

file: D:\PODACI\00 IVANA PUCEK\IvanaPucek diplomski rad\NMR\IP-57_F8-F10\fid expt: <zg30>
transmitter freq.: 600.135401 MHz
time domain size: 32768 points
width: 12019.23 Hz = 20.027532 ppm = 0.366798 Hz/pt
number of scans: 26

freq. of 0 ppm: 600.130010 MHz
processed size: 32768 complex points
LB: 0.000 GB: 0.0000

A part of the ^{1}H NMR spectrum (600 MHz, $CDCl_3$) of *trans,syn*-5-(4-chlorostyryl)thiophene-2-carbaldehyde oxime (*trans,syn*-15)

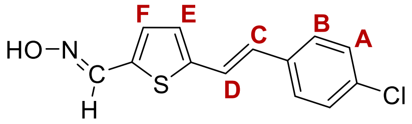

***trans,syn*-15**

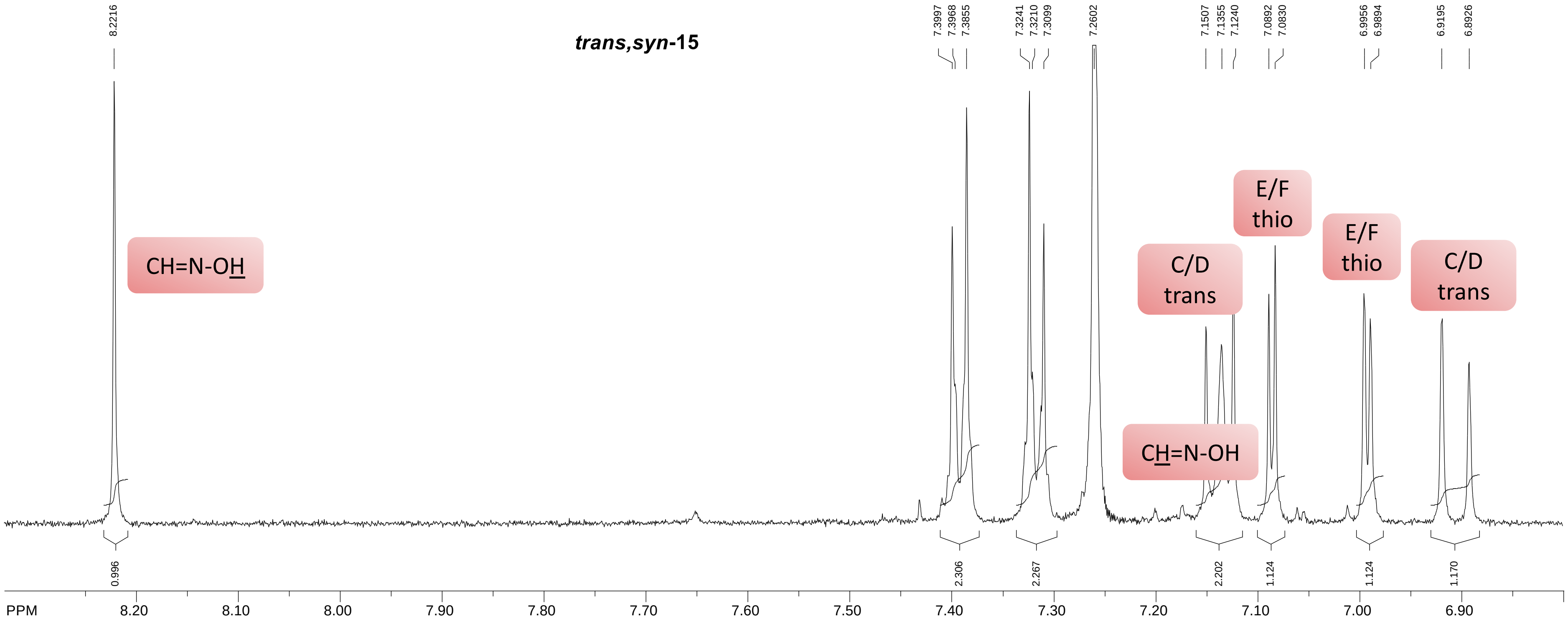

file: D:\PODACI\00 IVANA PUCEK\IvanaPucek diplomski rad\NMR\IP-57_F8-F10\fid expt: <zg30>
transmitter freq.: 600.135401 MHz
time domain size: 32768 points
width: 12019.23 Hz = 20.027532 ppm = 0.366798 Hz/pt
number of scans: 26

freq. of 0 ppm: 600.130010 MHz
processed size: 32768 complex points
LB: 0.000 GB: 0.0000

^{13}C NMR spectrum (150 MHz, CDCl$_3$) of *trans,syn*-5-(4-chlorostyryl)thiophene-2-carbaldehyde oxime (*trans,syn*-15)

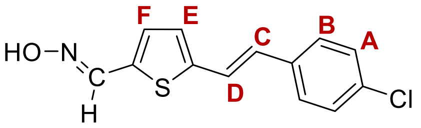

***trans,syn*-15**

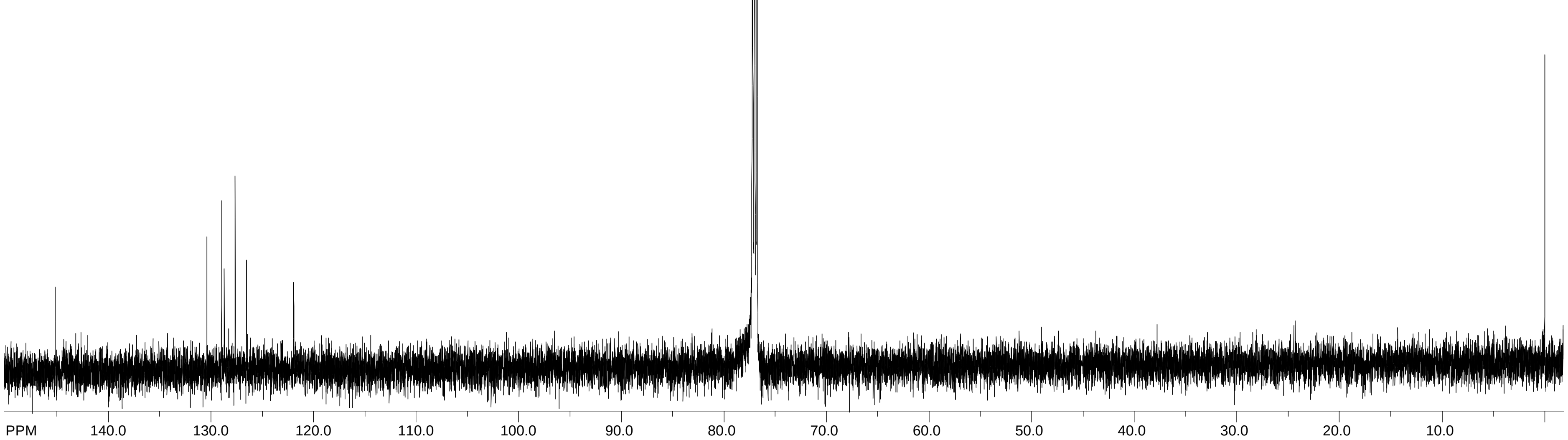

file: D:\PODACI\00 IVANA PUCEK\IvanaPucek diplomski rad\NMR\IP-57_F8-F10 sve\4\fid expt: <zgpg30>
transmitter freq.: 150.917899 MHz
time domain size: 65536 points
width: 35971.22 Hz = 238.349615 ppm = 0.548877 Hz/pt
number of scans: 21248

freq. of 0 ppm: 150.902809 MHz
processed size: 32768 complex points
LB: 0.000 GB: 0.0000

COSY spectrum of *trans,syn*-5-(4-chlorostyryl)thiophene-2-carbaldehyde oxime (*trans,syn*-15)

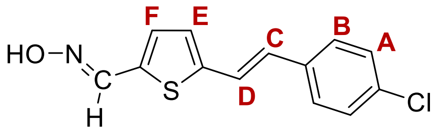

***trans,syn*-15**

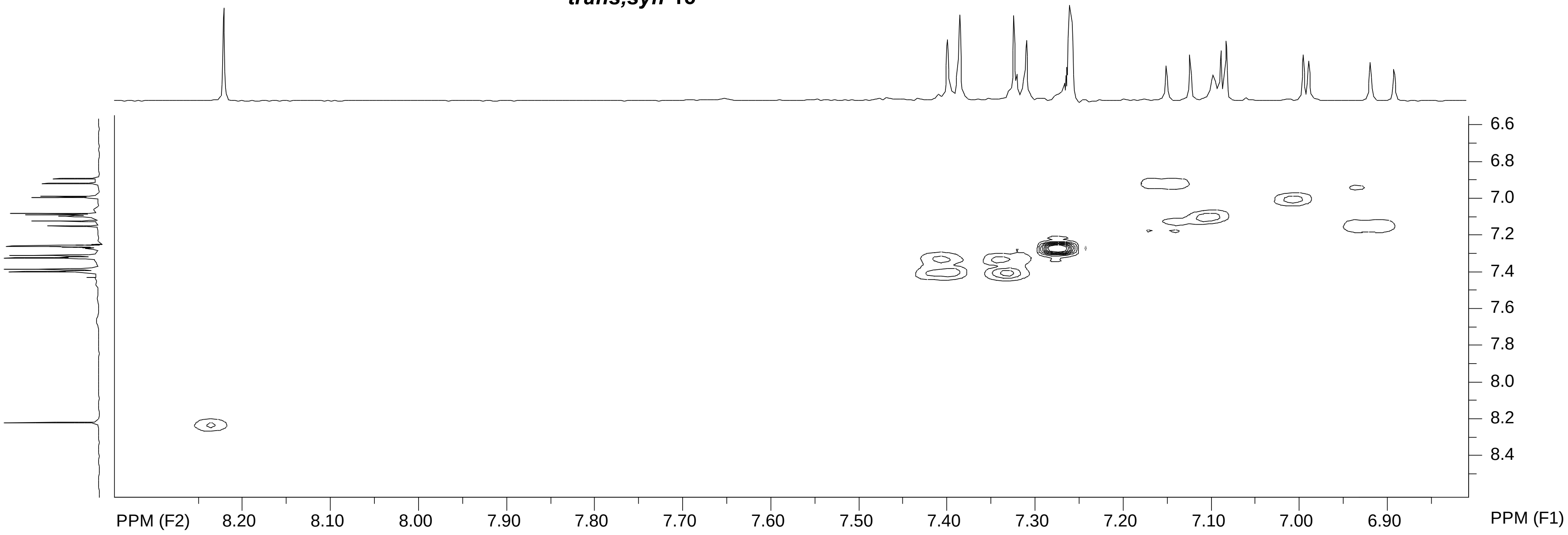

file: D:\PODACI\00 IVANA PUCEK\IvanaPucek diplomski rad\NMR\IP-57_F8-F10 sve\2\ser expt: <cosygpqf>
transmitter freq.: 600.133901 MHz
time domain size: 2048 by 512 points
width: 9615.38 Hz = 16.022065 ppm = 4.695012 Hz/pt
number of scans: 4

F2: freq. of 0 ppm: 600.130000 MHz
processed size: 1024 complex points
window function: Sine
shift: 0.0 degrees

F1: freq. of 0 ppm: 600.130000 MHz
processed size: 1024 complex points
window function: Sine
shift: 0.0 degrees

HSQC spectrum of *trans,syn*-5-(4-chlorostyryl)thiophene-2-carbaldehyde oxime (*trans,syn*-15)

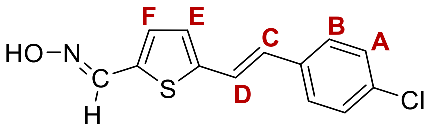

***trans,syn*-15**

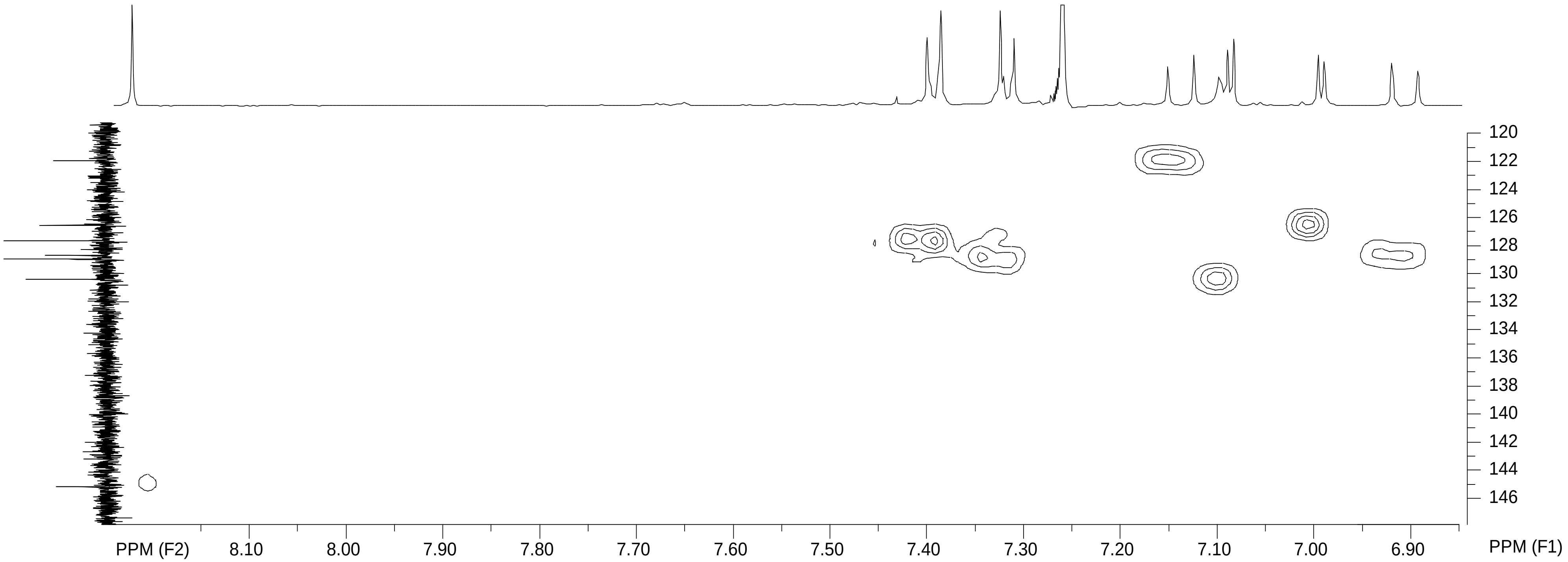

file: D:\PODACI\00 IVANA PUCEK\IvanaPucek diplomski rad\NMR\IP-57_F8-F10 sve\3\ser expt: <inv4gpqf>
transmitter freq.: 600.133901 MHz
time domain size: 2048 by 256 points
width: 9615.38 Hz = 16.022065 ppm = 4.695012 Hz/pt
number of scans: 64

F2: freq. of 0 ppm: 600.130000 MHz
processed size: 1024 complex points
window function: Sine
shift: 0.0 degrees

F1: freq. of 0 ppm: 150.902809 MHz
processed size: 1024 complex points
window function: Sine
shift: 0.0 degrees

^{1}H NMR spectrum (600 MHz, $CDCl_3$) of *trans,anti*-5-(4-chlorostyryl)thiophene-2-carbaldehyde oxime (*trans,anti*-15)

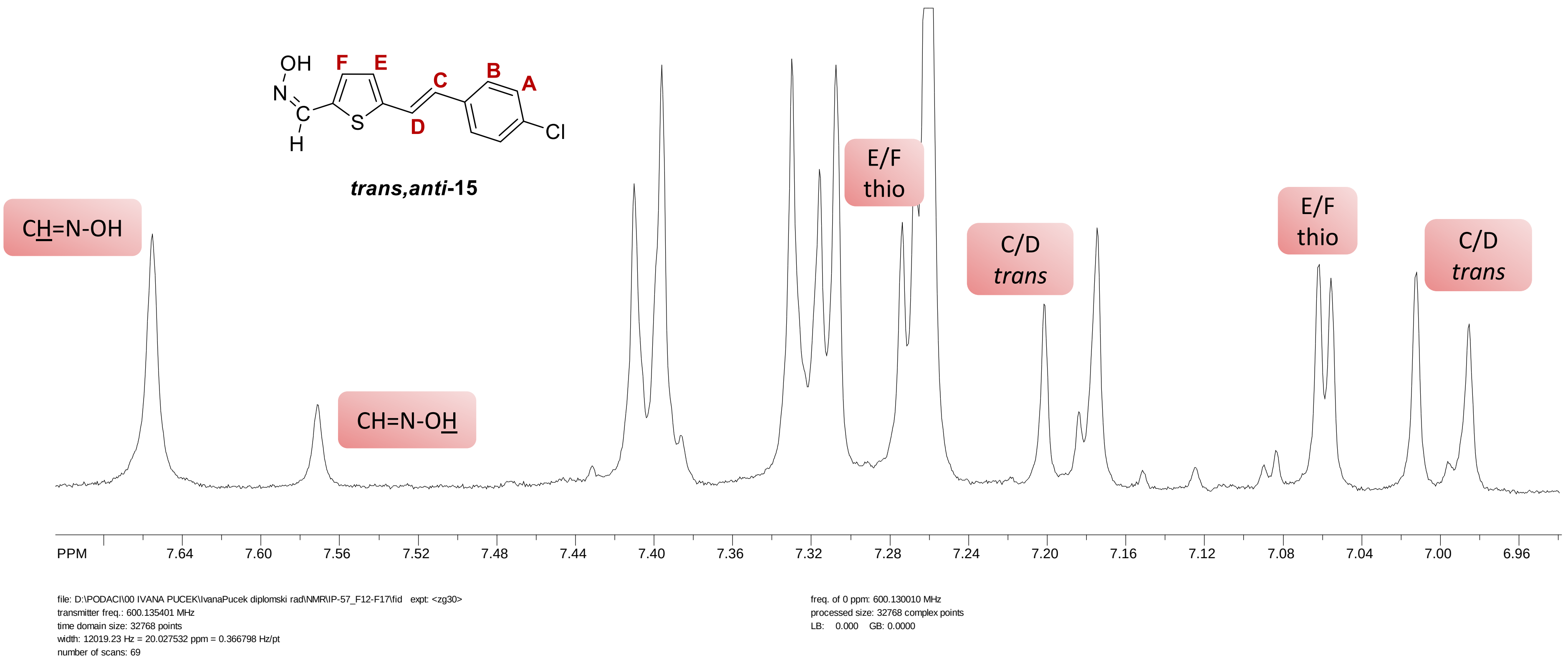

^{13}C NMR spectrum (150 MHz, $CDCl_3$) of *trans,anti*-5-(4-chlorostyryl)thiophene-2-carbaldehyde oxime (*trans,anti*-15)

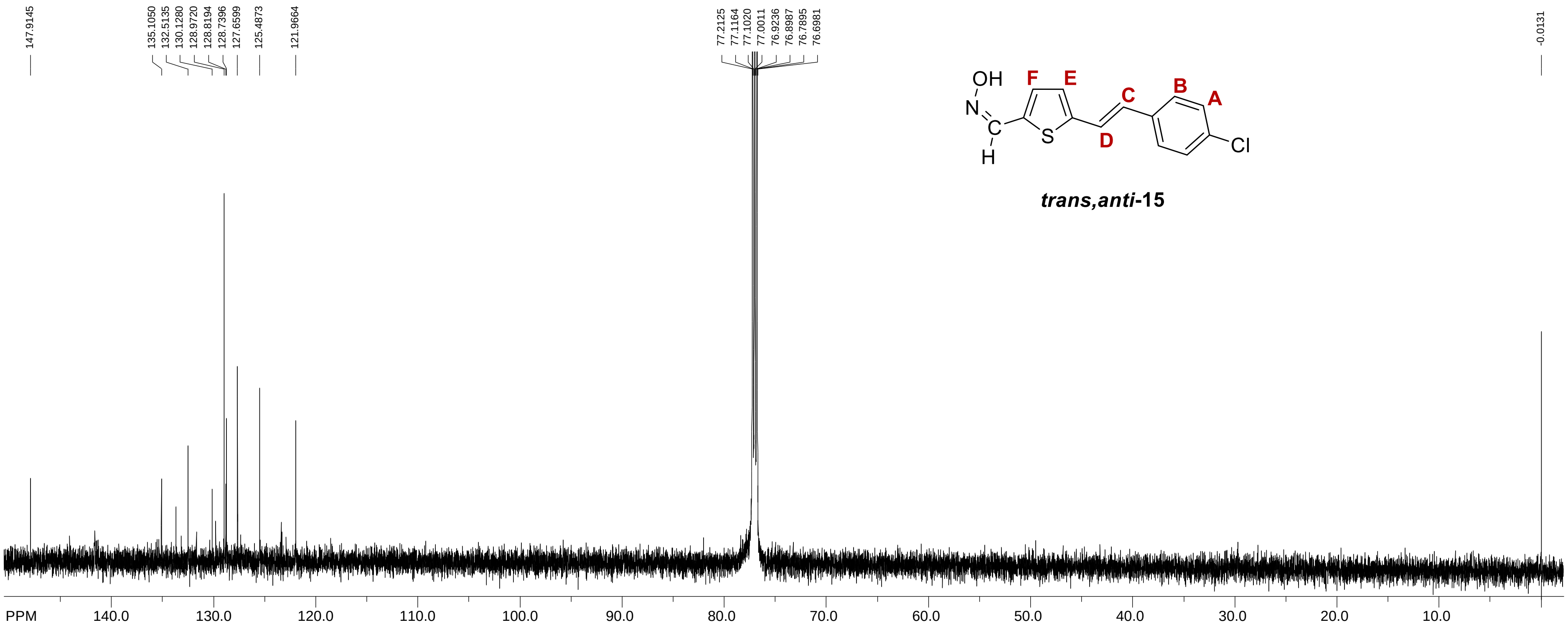

file: D:\PODACI\00 IVANA PUCEK\IvanaPucek diplomski rad\NMR\IP-57_F12-F17 sve\4\fid expt: <zgpg30>
transmitter freq.: 150.917899 MHz
time domain size: 65536 points
width: 35971.22 Hz = 238.349615 ppm = 0.548877 Hz/pt
number of scans: 65536

freq. of 0 ppm: 150.902809 MHz
processed size: 32768 complex points
LB: 0.000 GB: 0.0000

Mass spectra and HRMS analysis of the mixture of geometrical isomers of 5-(4-chlorostyryl)thiophene-2-carbaldehyde oxime (15)

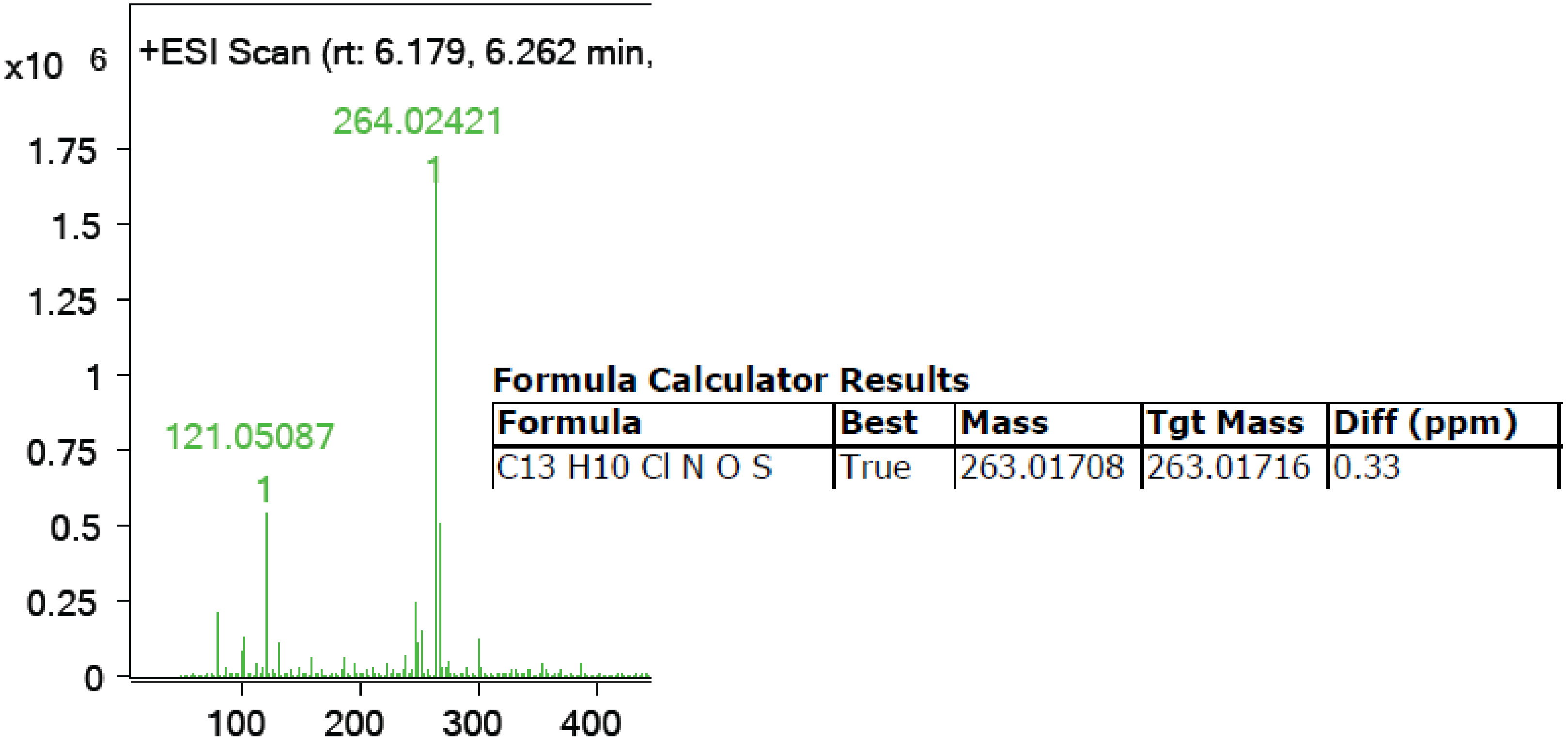

Formula Calculator Results

Formula	Best	Mass	Tgt Mass	Diff (ppm)
C13 H10 Cl N O S	True	263.01708	263.01716	0.33

^{1}H NMR spectrum (600 MHz, $CDCl_3$) of *cis,syn*-4-(2-(5-((hydroxyimino)methyl)thiophen-2-yl)vinyl)benzonitrile (*cis,syn*-16) in the mixture with *trans,syn*-16

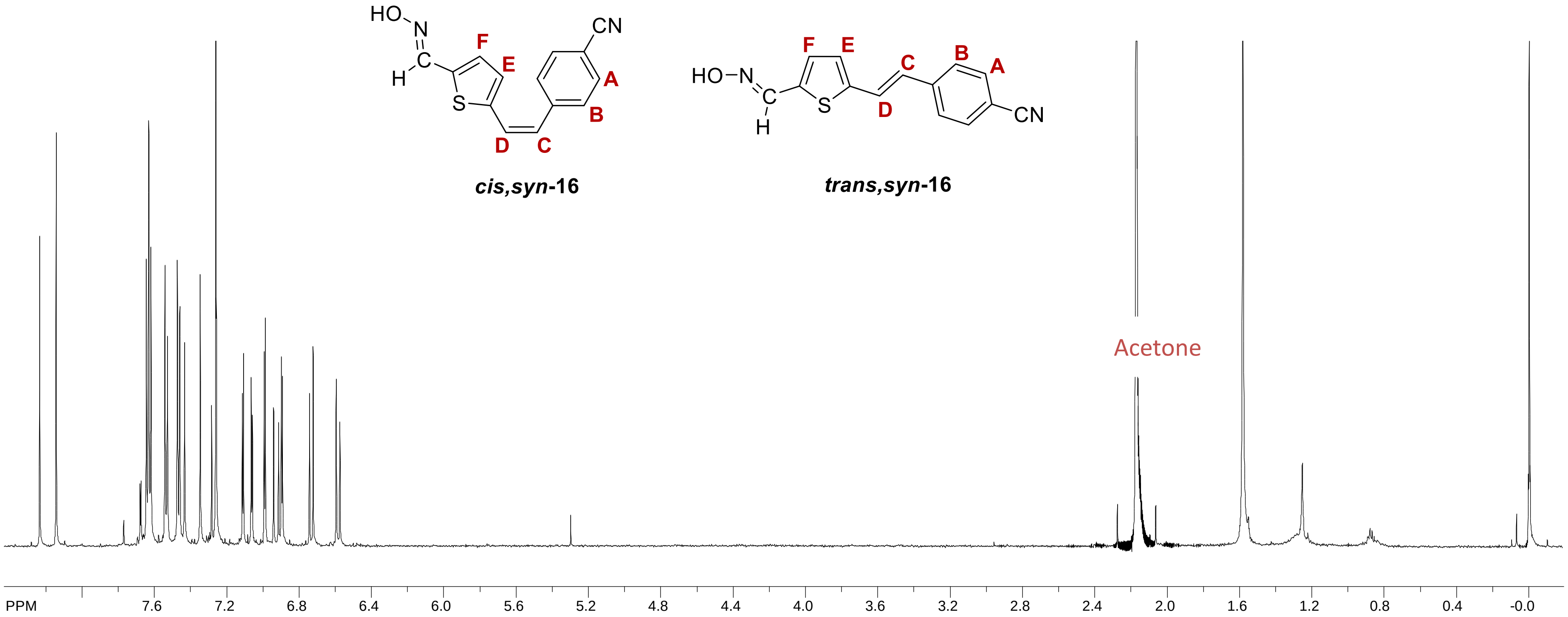

file: D:\PODACI\00 IVANA PUCEK\IvanaPucek diplomski rad\NMR\IP-62_F19-F24_A\fid expt: <zg30>
transmitter freq.: 600.135401 MHz
time domain size: 32768 points
width: 12019.23 Hz = 20.027532 ppm = 0.366798 Hz/pt
number of scans: 33

freq. of 0 ppm: 600.130010 MHz
processed size: 32768 complex points
LB: 0.000 GB: 0.0000

A part of the ^{1}H NMR spectrum (600 MHz, $CDCl_3$) of *cis,syn*-4-(2-(5-((hydroxyimino)methyl)thiophen-2-yl)vinyl)benzonitrile (*cis,syn*-16) in the mixture with *trans,syn*-16

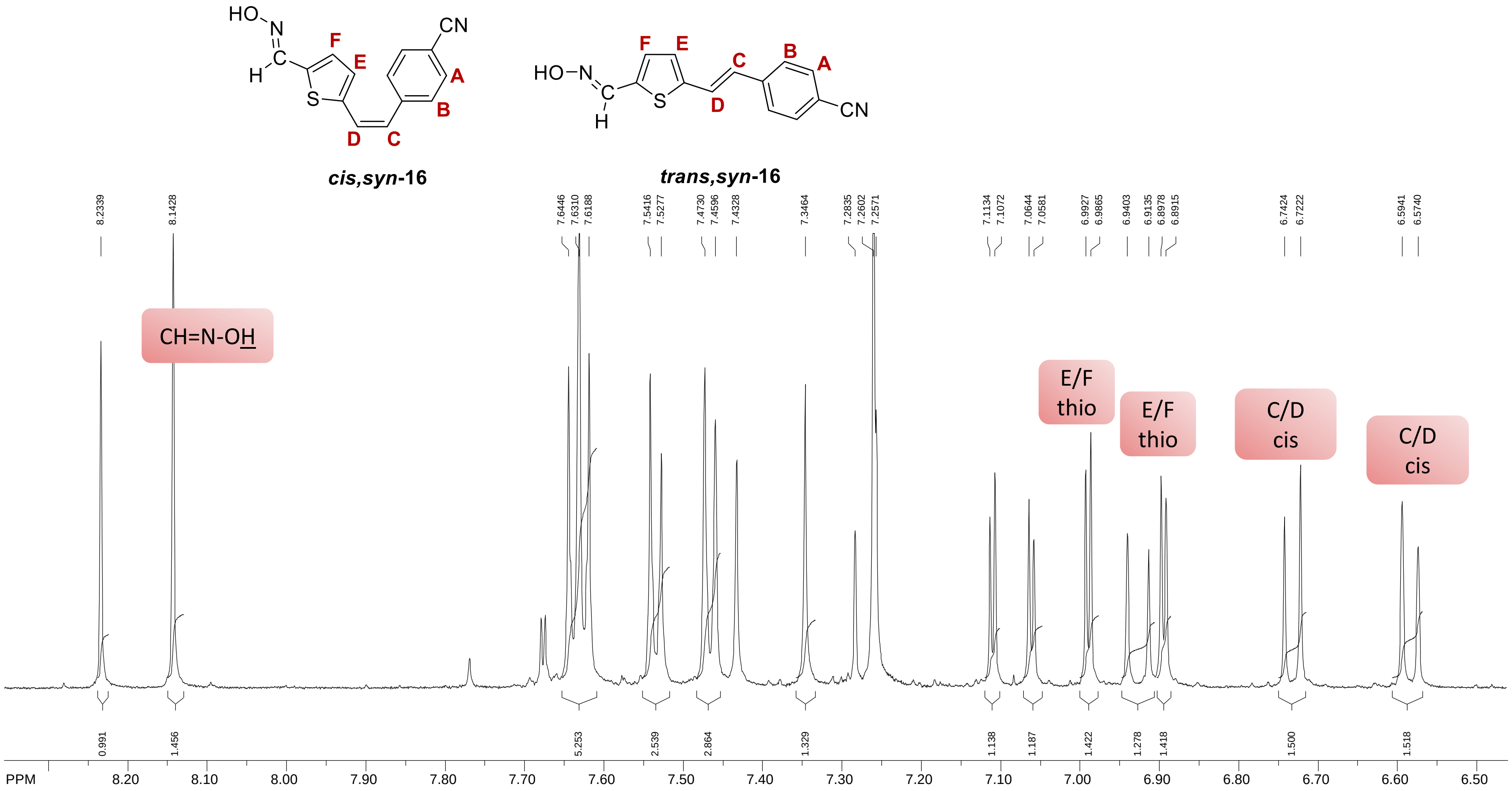

file: D:\PODACI\00 IVANA PUCEK\IvanaPucek diplomski rad\NMR\IP-62_F19-F24_A\fid expt: <zg30>
transmitter freq.: 600.135401 MHz
time domain size: 32768 points
width: 12019.23 Hz = 20.027532 ppm = 0.366798 Hz/pt
number of scans: 33

freq. of 0 ppm: 600.130010 MHz
processed size: 32768 complex points
LB: 0.000 GB: 0.0000

^{1}H NMR spectrum (600 MHz, $CDCl_3$) of *trans,syn*-4-(2-(5-((hydroxyimino)methyl)thiophen-2-yl)vinyl)benzonitrile (*trans,syn*-16) with traces of *cis,syn*-**16**

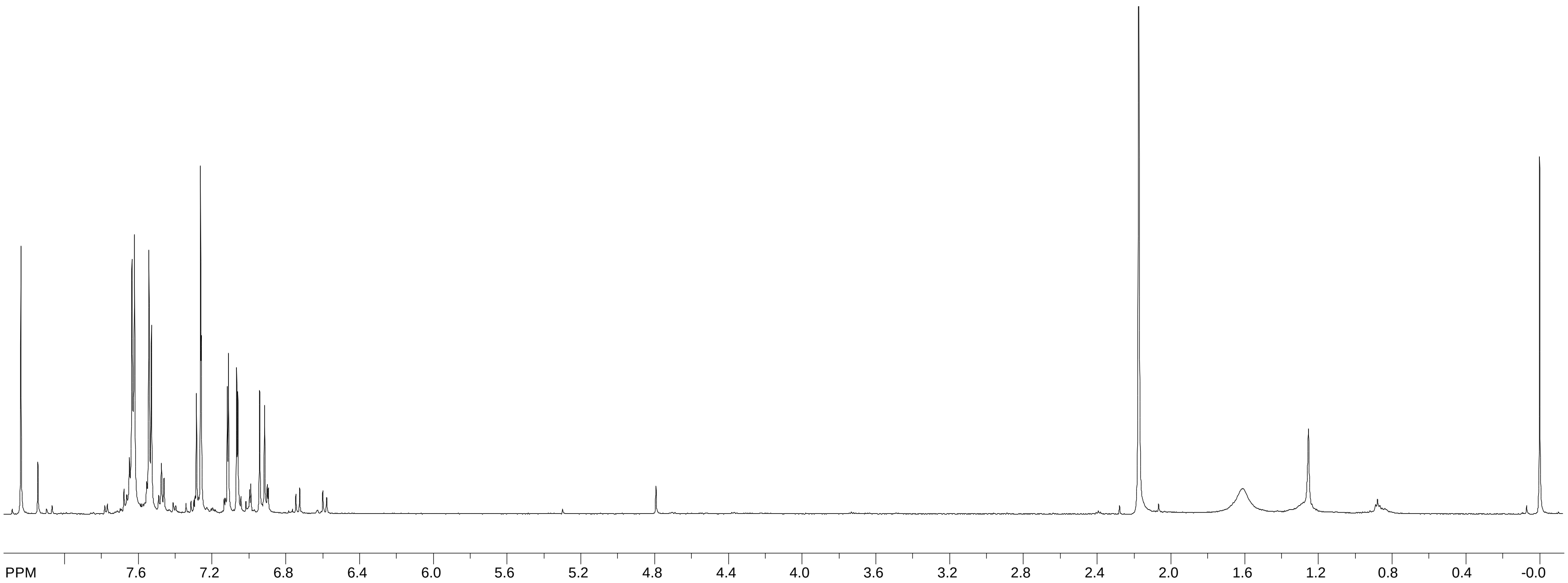

file: D:\PODACI\00 IVANA PUCEK\IvanaPucek diplomski rad\NMR\IP-62_F19-F24_B sve\1\fid expt: <zg30>
transmitter freq.: 600.135401 MHz
time domain size: 32768 points
width: 12019.23 Hz = 20.027532 ppm = 0.366798 Hz/pt
number of scans: 37

freq. of 0 ppm: 600.130009 MHz
processed size: 32768 complex points
LB: 0.000 GB: 0.0000

A part of the ^{1}H NMR spectrum (600 MHz, $CDCl_3$) of *trans,syn*-4-(2-(5-((hydroxyimino)methyl)thiophen-2-yl)vinyl)benzonitrile (*trans,syn*-16) with traces of *cis,syn*-**16**

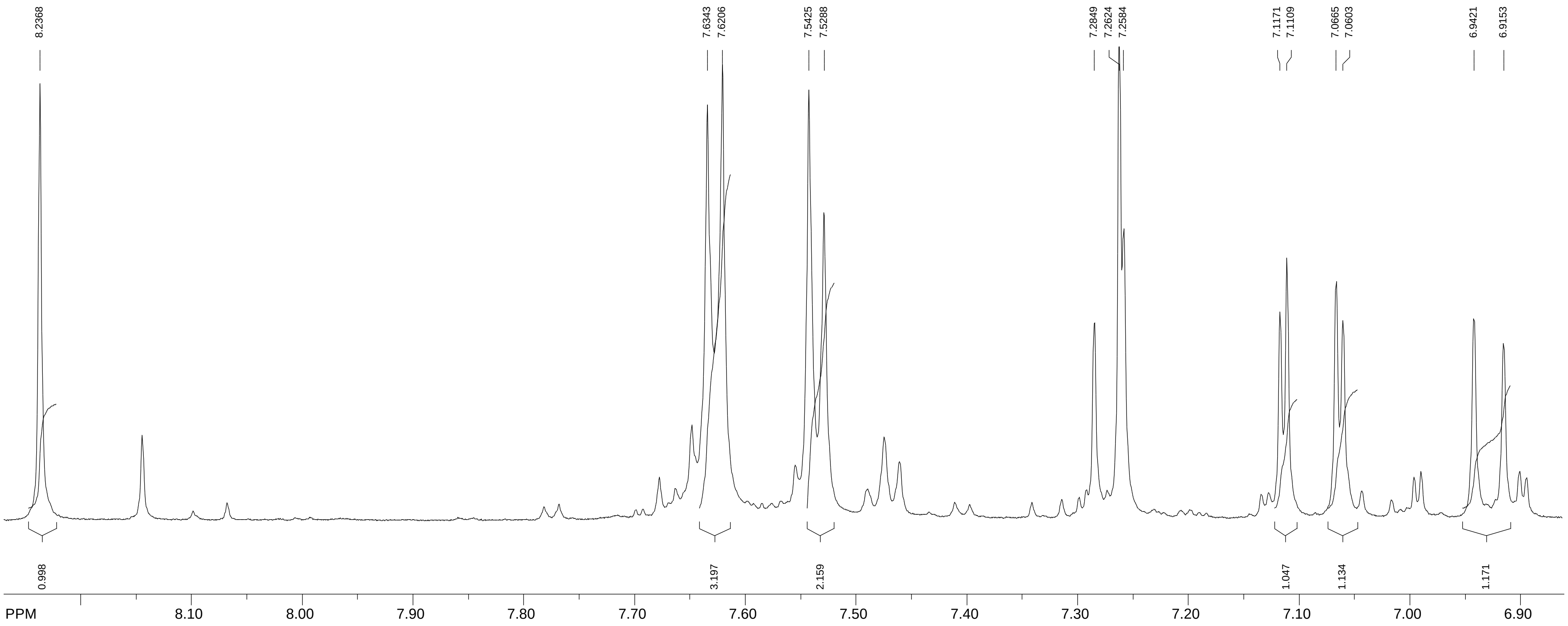

file: D:\PODACI\00 IVANA PUCEK\IvanaPucek diplomski rad\NMR\IP-62_F19-F24_B sve\1\fid expt: <zg30>
transmitter freq.: 600.135401 MHz
time domain size: 32768 points
width: 12019.23 Hz = 20.027532 ppm = 0.366798 Hz/pt
number of scans: 37

freq. of 0 ppm: 600.130009 MHz
processed size: 32768 complex points
LB: 0.000 GB: 0.0000

^{13}C NMR spectrum (150 MHz, $CDCl_3$) of *trans,syn*-4-(2-(5-((hydroxyimino)methyl)thiophen-2-yl)vinyl)benzonitrile (*trans,syn*-16)

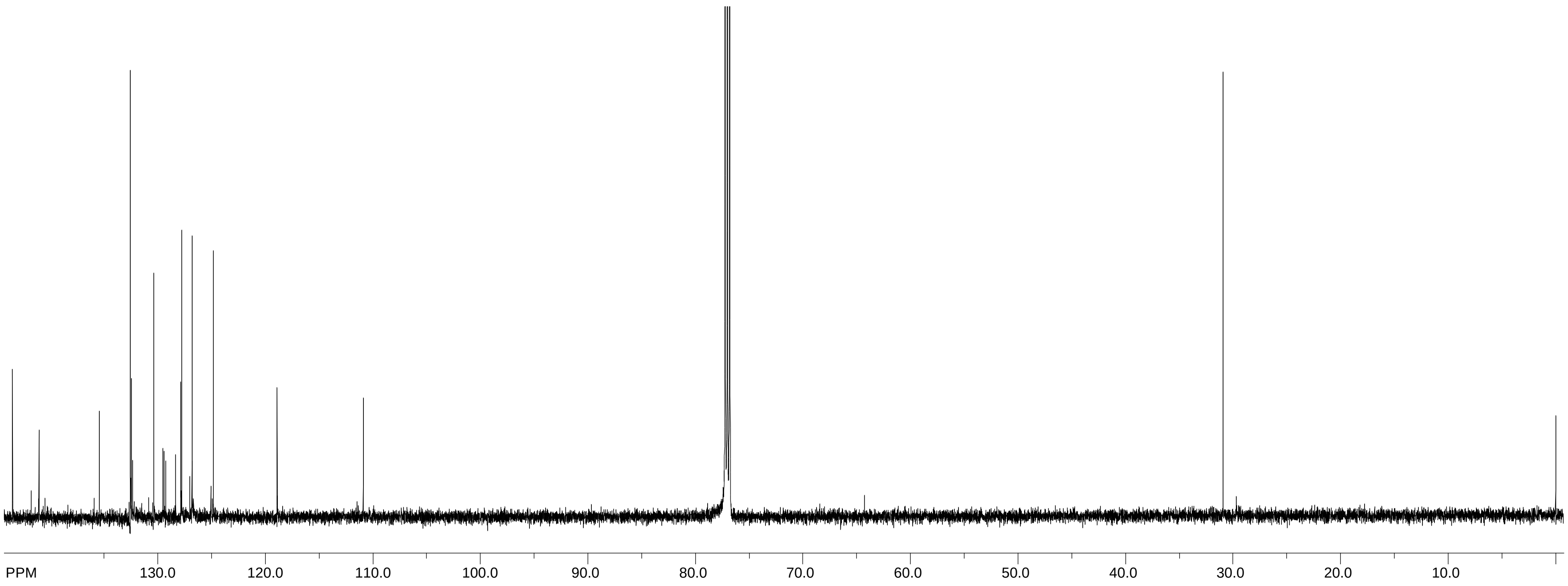

file: D:\PODACI\00 IVANA PUCEK\IvanaPucek diplomski rad\NMR\IP-62_F19-F24_B sve\4\fid expt: <zgpg30>
transmitter freq.: 150.917899 MHz
time domain size: 65536 points
width: 35971.22 Hz = 238.349615 ppm = 0.548877 Hz/pt
number of scans: 20821

freq. of 0 ppm: 150.902809 MHz
processed size: 32768 complex points
LB: 0.000 GB: 0.0000

^{1}H NMR spectrum (600 MHz, $CDCl_3$) of *cis,anti*-4-(2-(5-((hydroxyimino)methyl)thiophen-2-yl)vinyl)benzonitrile (*cis,anti*-16) with traces of other isomers

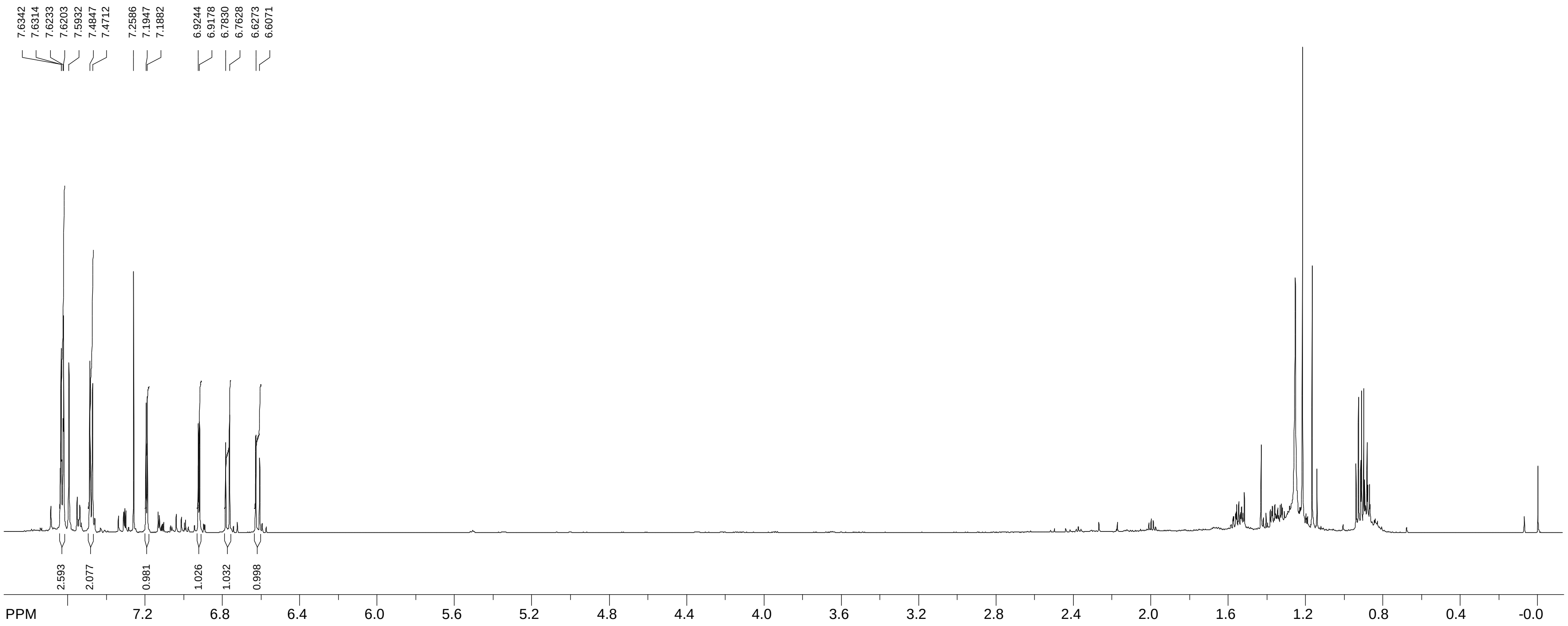

file: D:\SURADNJE\ZRINKA KOVARIK\00 ZK Oximes\SupportingInformation\oksimi cisti spektri MD\oksimi\MD-88(46-54)(2-7) najbolji koji imam od CN\fid expt: <zg30>
transmitter freq.: 600.135401 MHz
time domain size: 32768 points
width: 12019.23 Hz = 20.027532 ppm = 0.366798 Hz/pt
number of scans: 27

freq. of 0 ppm: 600.130010 MHz
processed size: 32768 complex points
LB: 0.000 GB: 0.0000

A part of the ¹H NMR spectrum (600 MHz, CDCl₃) of *cis,anti*-4-(2-(5-((hydroxyimino)methyl)thiophen-2-yl)vinyl)benzonitrile (*cis,anti*-16) with traces of other isomers

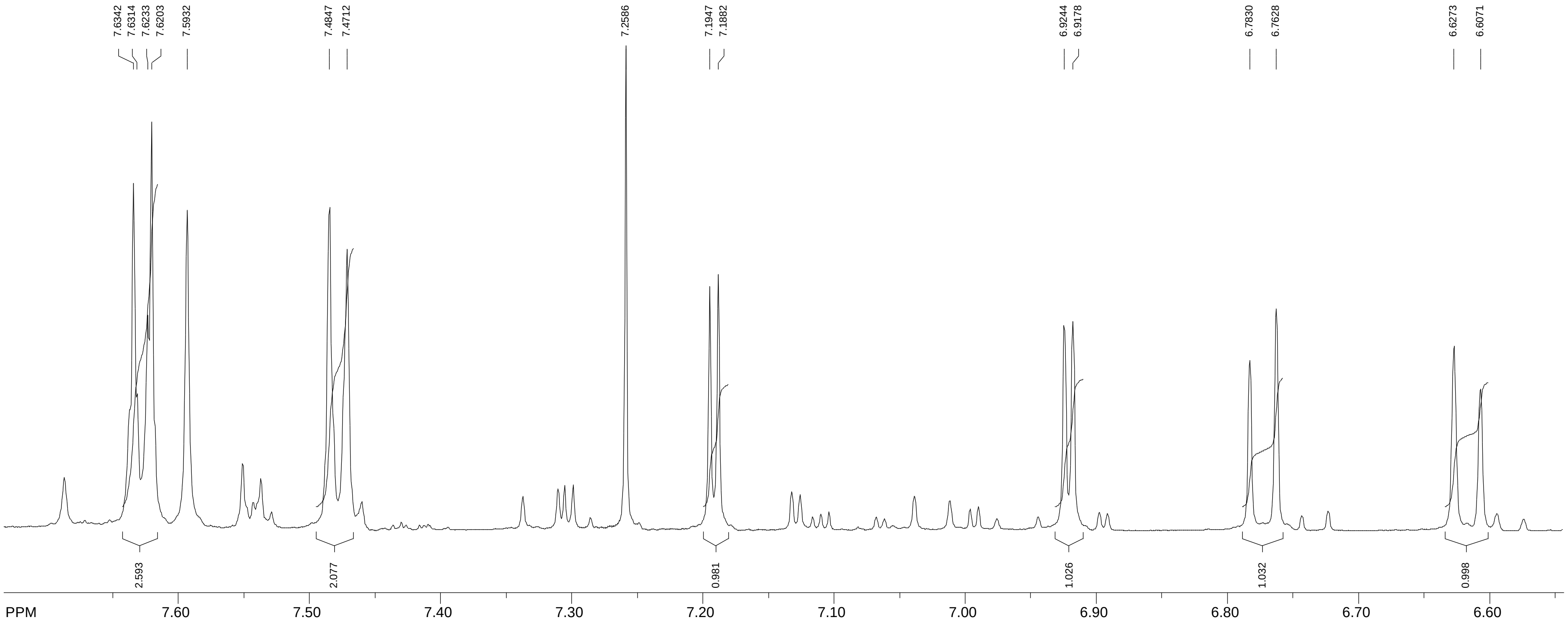

file: D:\SURADNJE\ZRINKA KOVARIK\00 ZK Oximes\SupportingInformation\oksimi cisti spektri MD\oksimi\MD-88(46-54)(2-7) najbolji koji imam od CN\fid expt: <zg30>
transmitter freq.: 600.135401 MHz
time domain size: 32768 points
width: 12019.23 Hz = 20.027532 ppm = 0.366798 Hz/pt
number of scans: 27

freq. of 0 ppm: 600.130010 MHz
processed size: 32768 complex points
LB: 0.000 GB: 0.0000

^{1}H NMR spectrum (600 MHz, $CDCl_3$ + CD_3OD) of *trans,anti*-4-(2-(5-((hydroxyimino)methyl)thiophen-2-yl)vinyl)benzonitrile (*trans,anti*-16)

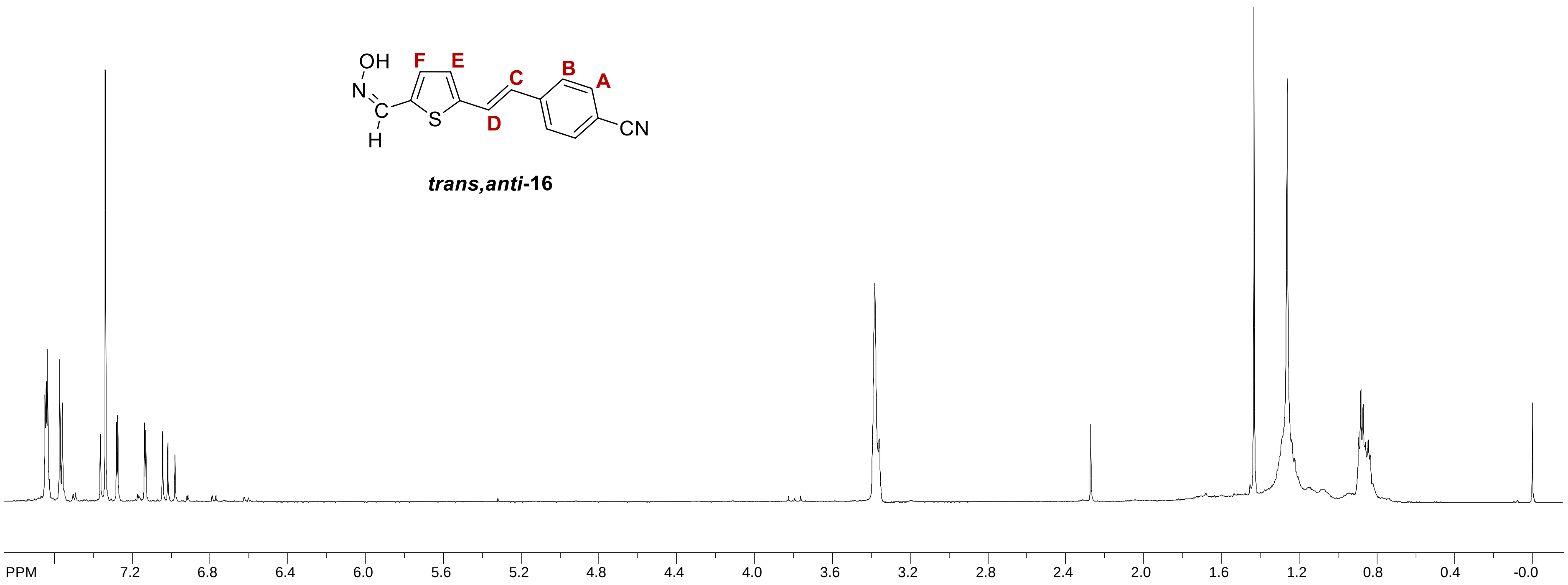

file: G:\00 ZK Oximes\SupportingInformation\oksimi cisti spektri MD\MD-88(46-54)(8-13)(11-14) CN trans najbolji koji imam\fid expt: <zg30>
transmitter freq.: 600.135401 MHz
time domain size: 32768 points
width: 12019.23 Hz = 20.027532 ppm = 0.366798 Hz/pt
number of scans: 48

freq. of 0 ppm: 600.129964 MHz
processed size: 32768 complex points
LB: 0.000 GB: 0.0000

A part of the ^{1}H NMR spectrum (600 MHz, $CDCl_3$ + CD_3OD) of *trans,anti*-4-(2-(5-((hydroxyimino)methyl)thiophen-2-yl)vinyl)benzonitrile (*trans,anti*-16)

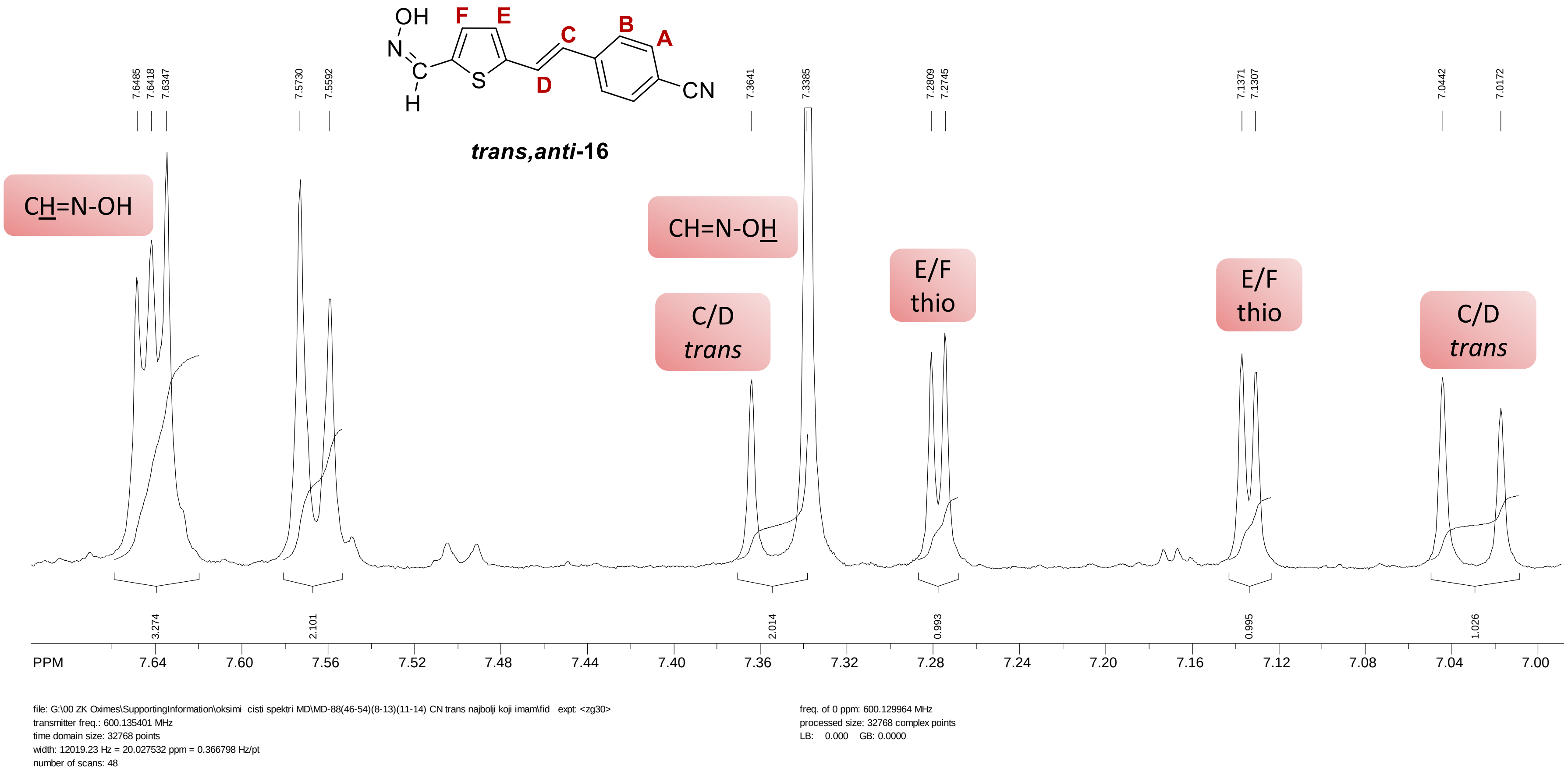

^{13}C NMR (APT) spectrum (150 MHz, $CDCl_3$ + CD_3OD) of *trans,anti*-4-(2-(5-((hydroxyimino)methyl)thiophen-2-yl)vinyl)benzonitrile (*trans,anti*-16)

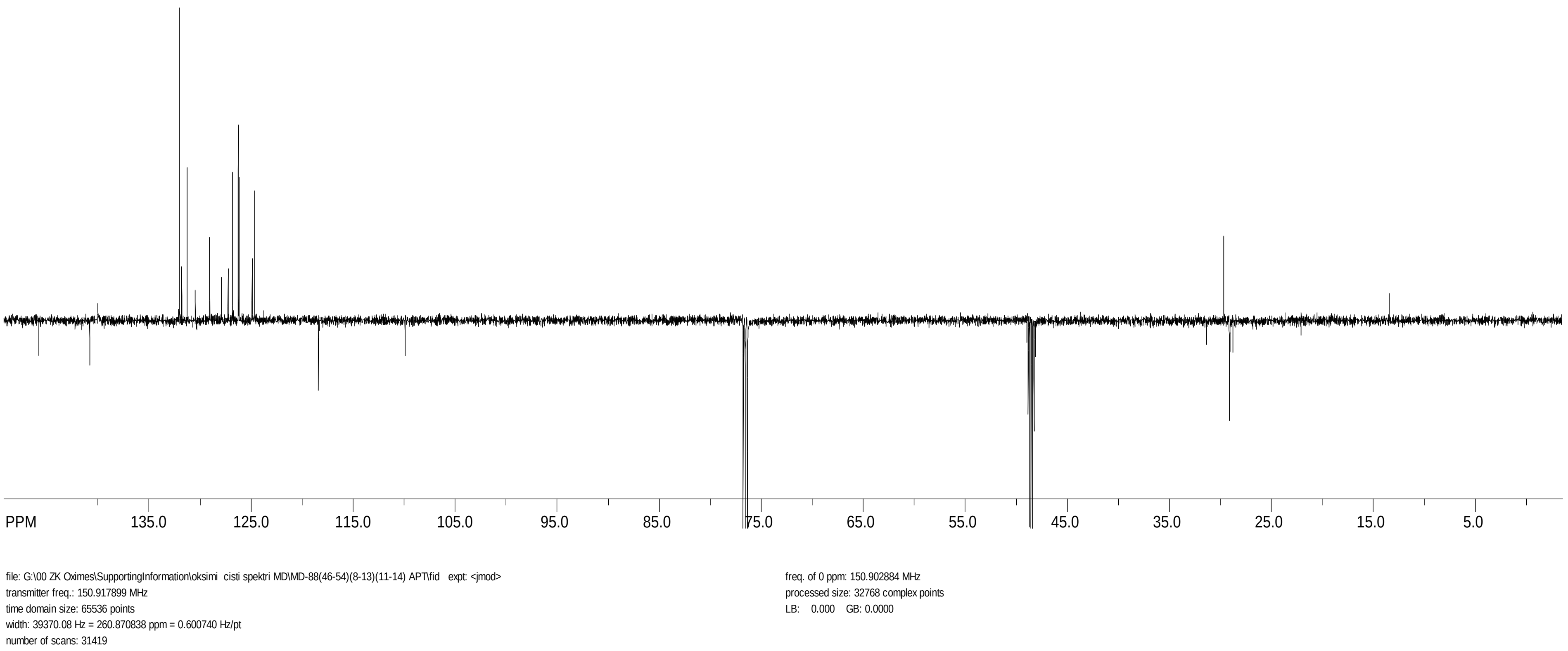

Mass spectra and HRMS analysis of the mixture of geometrical isomers of 4-(2-(5-hydroxyiminomethyl)thiophen-2-yl)vinyl)benzonitrile (16)

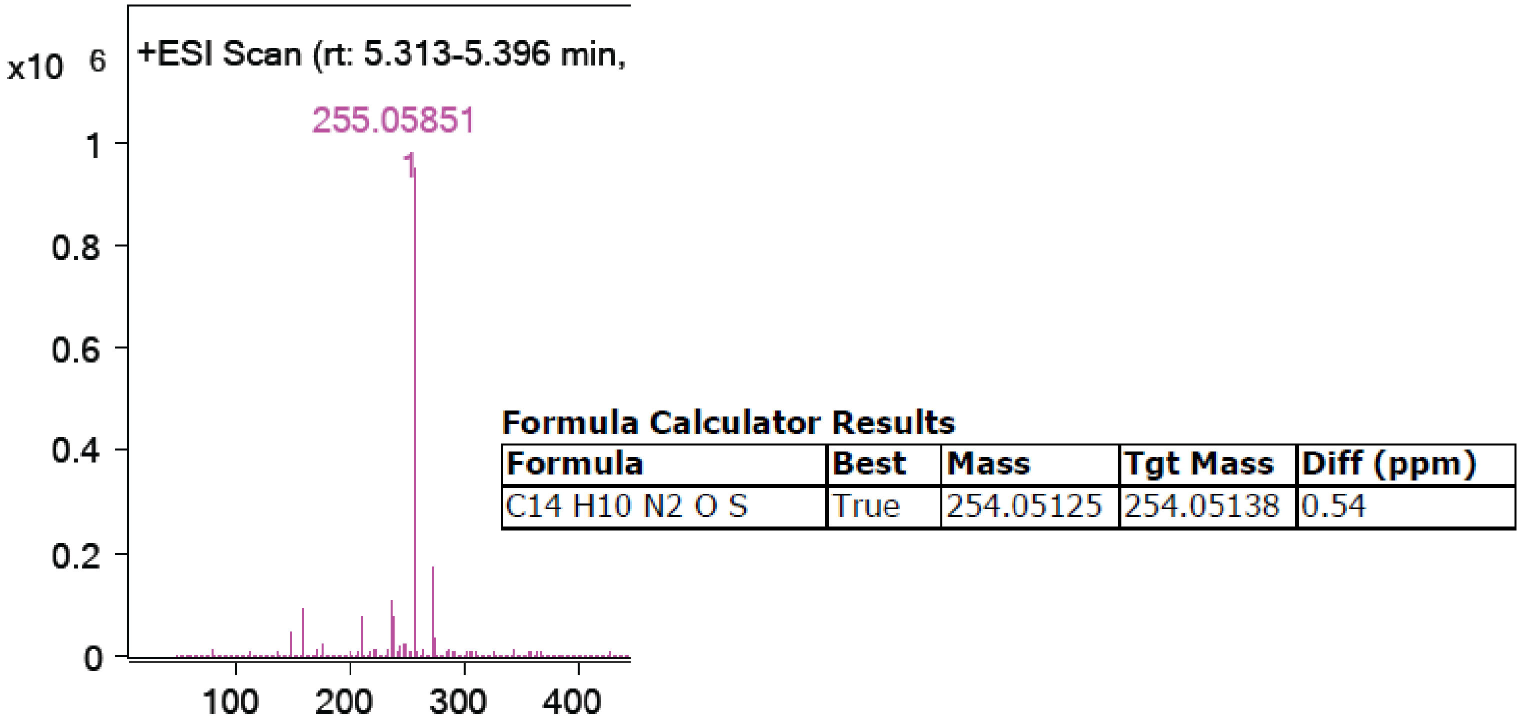

Formula Calculator Results

Formula	Best	Mass	Tgt Mass	Diff (ppm)
C14 H10 N2 O S	True	254.05125	254.05138	0.54

^{1}H NMR spectrum (600 MHz, $CDCl_3$) of *trans,syn*-5-(4-nitrostyryl)thiophene-2-carbaldehyde oxime (*trans,syn*-17)

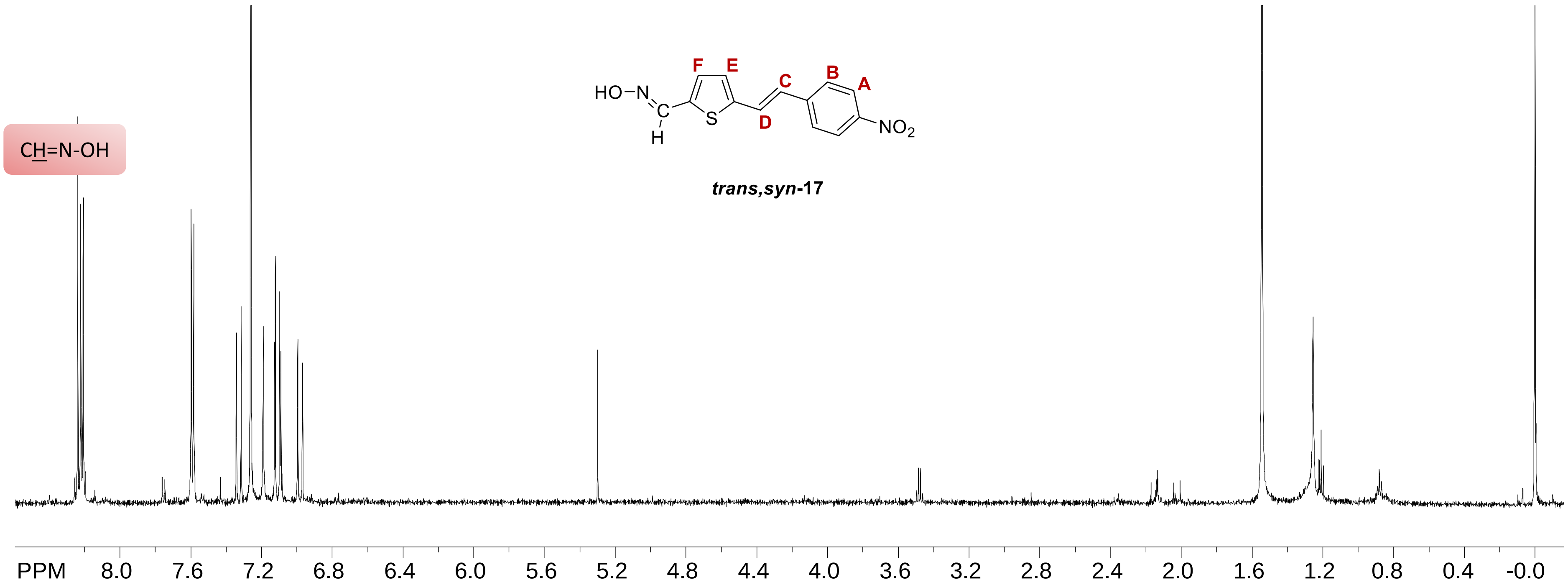

file: D:\SURADNJE\ZRINKA KOVARIK\00 ZK Oximes\SupportingInformation\oksimi cisti spektri MD\MD-93(C1)- oksim trans syn nitro\fid expt: <zg30>
transmitter freq.: 600.135401 MHz
time domain size: 32768 points
width: 12019.23 Hz = 20.027532 ppm = 0.366798 Hz/pt
number of scans: 31

freq. of 0 ppm: 600.130010 MHz
processed size: 32768 complex points
LB: 0.000 GB: 0.0000

A part of the ^{1}H NMR spectrum (600 MHz, $CDCl_3$) of *trans,syn*-5-(4-nitrostyryl)thiophene-2-carbaldehyde oxime (*trans,syn*-17)

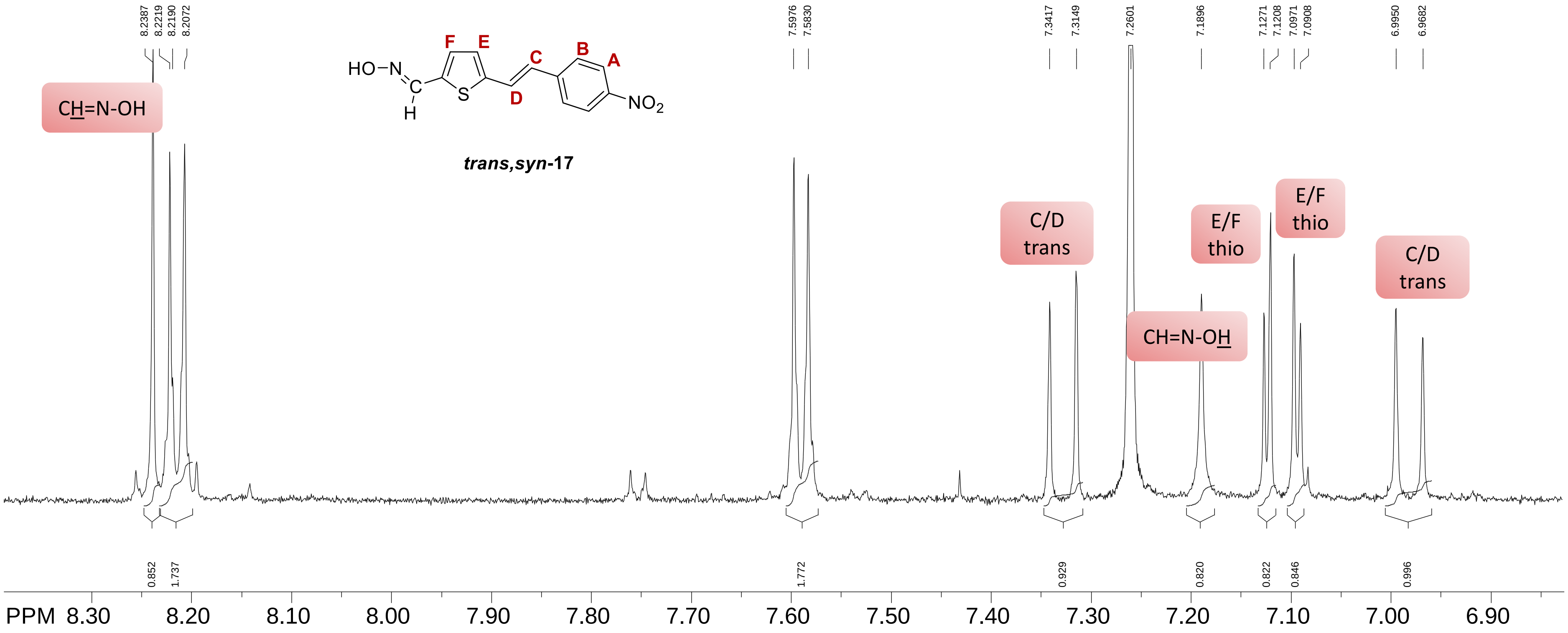

file: D:\SURADNJE\ZRINKA KOVARIK\00 ZK Oximes\SupportingInformation\oksimi cisti spektri MD\MD-93(C1)- oksim trans syn nitro\fid expt: <zg30>
transmitter freq.: 600.135401 MHz
time domain size: 32768 points
width: 12019.23 Hz = 20.027532 ppm = 0.366798 Hz/pt
number of scans: 31

freq. of 0 ppm: 600.130010 MHz
processed size: 32768 complex points
LB: 0.000 GB: 0.0000

^{13}C NMR spectrum (150 MHz, $CDCl_3$) of *trans,syn*-5-(4-nitrostyryl)thiophene-2-carbaldehyde oxime (*trans,syn*-17)

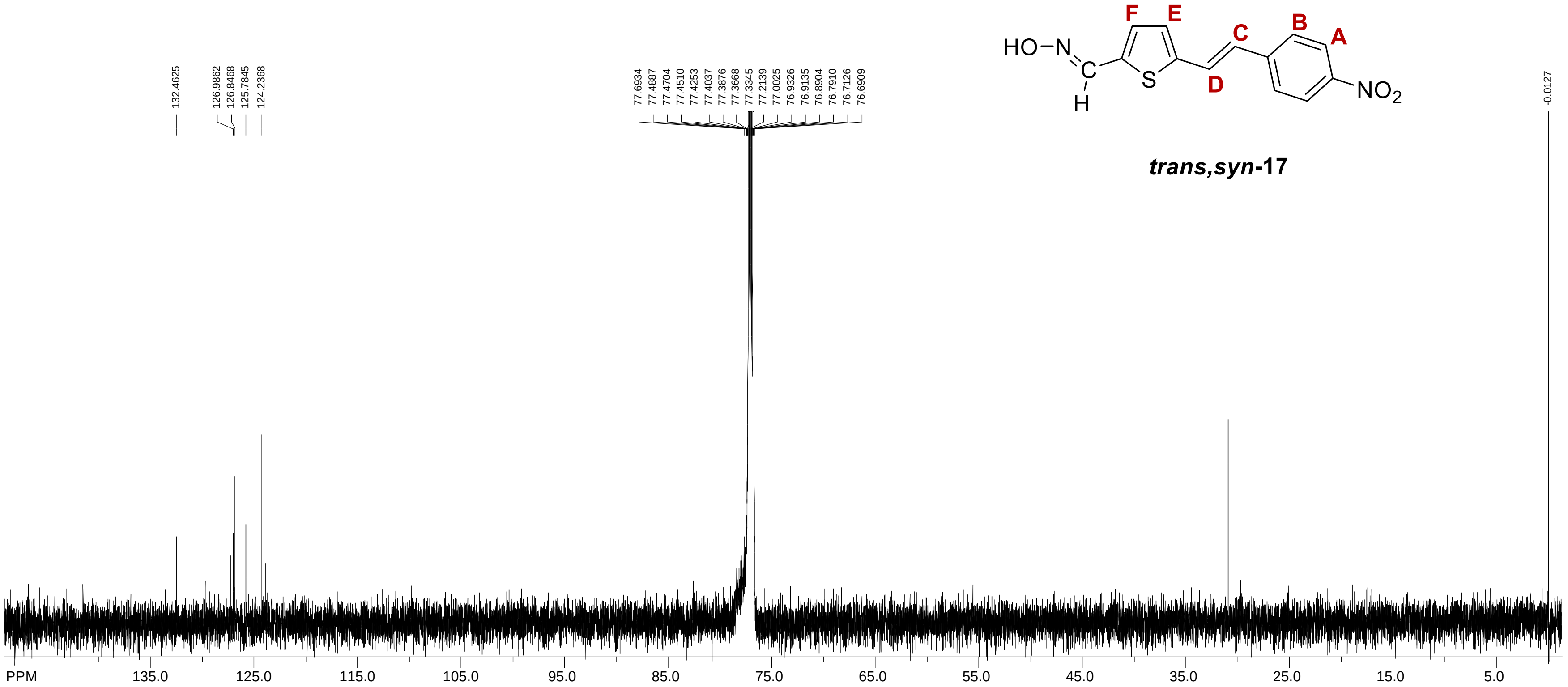

file: D:\PODACI\00 IVANA PUCEK\IvanaPucek diplomski rad\NMR\IP-67_F21-F24 sve\4\fid expt: <zgpg30>
transmitter freq.: 150.917899 MHz
time domain size: 65536 points
width: 35971.22 Hz = 238.349615 ppm = 0.548877 Hz/pt
number of scans: 65536

freq. of 0 ppm: 150.902809 MHz
processed size: 32768 complex points
LB: 0.000 GB: 0.0000

COSY spectrum of *trans,syn*-5-(4-nitrostyryl)thiophene-2-carbaldehyde oxime (*trans,syn*-17)

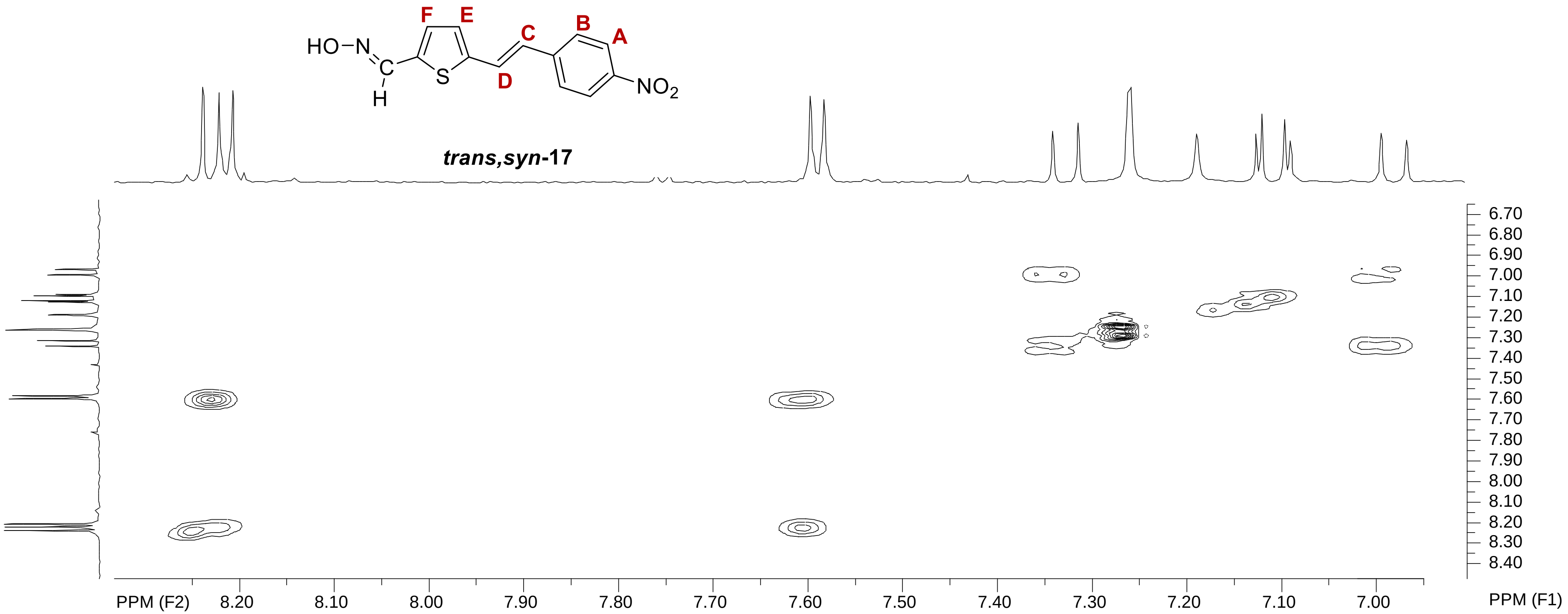

file: D:\SURADNJE\ZRINKA KOVARIK\00 ZK Oximes\SupportingInformation\oksimi cisti spektri MD\oksimi\MD-93(C1) ser 1\ser [illegible]
transmitter freq.: 600.133901 MHz
time domain size: 2048 by 512 points
width: 9615.38 Hz = 16.022065 ppm = 4.695012 Hz/pt
number of scans: 8

F2: freq. of 0 ppm: 600.130000 MHz
processed size: 1024 complex points
window function: Sine
shift: 0.0 degrees

F1: freq. of 0 ppm: 600.130000 MHz
processed size: 1024 complex points
window function: Sine
shift: 0.0 degrees

^{1}H NMR spectrum (600 MHz, $CDCl_3$) of *trans,anti*-5-(4-nitrostyryl)thiophene-2-carbaldehyde oxime (*trans,anti*-17) with traces of *cis,anti*-**17**

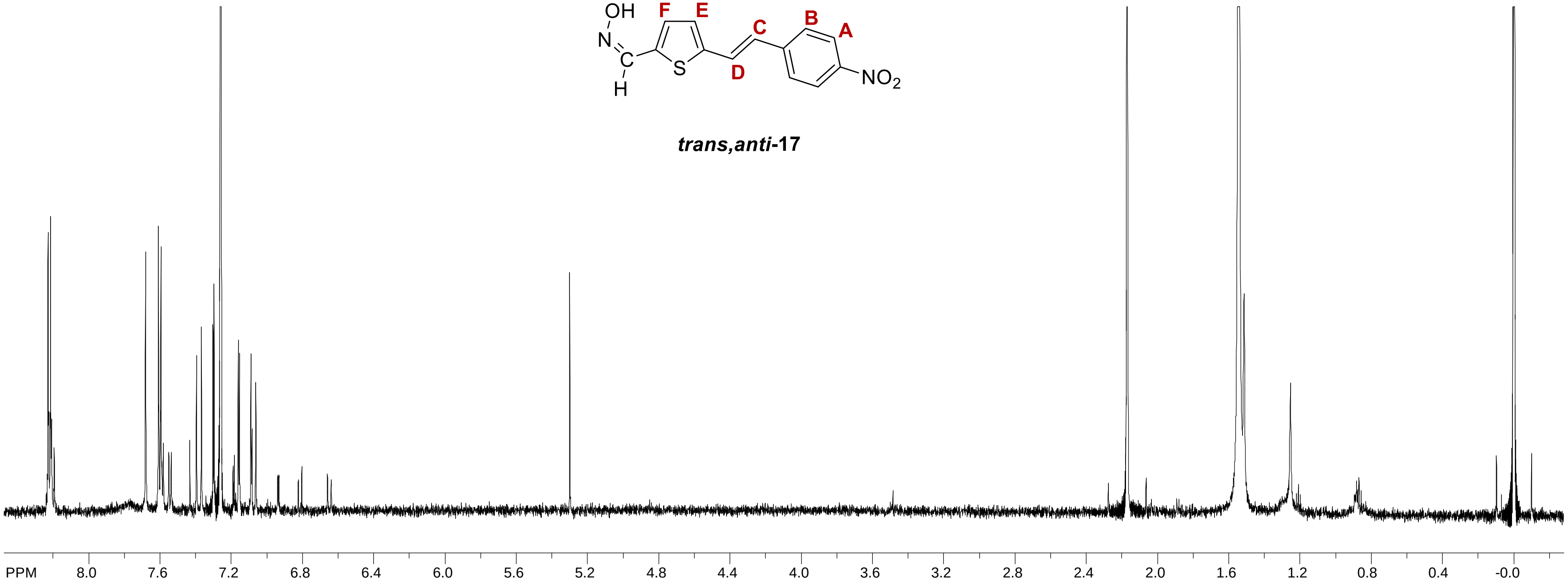

file: D:\PODACI\00 IVANA PUCEK\IvanaPucek diplomski rad\NMR\IP-67_F21-F24\fid expt: <zg30>
transmitter freq.: 600.135401 MHz
time domain size: 32768 points
width: 12019.23 Hz = 20.027532 ppm = 0.366798 Hz/pt
number of scans: 45

freq. of 0 ppm: 600.130010 MHz
processed size: 32768 complex points
LB: 0.000 GB: 0.0000

A part of the ^{1}H NMR spectrum (600 MHz, $CDCl_3$) of *trans,anti*-5-(4-nitrostyryl)thiophene-2-carbaldehyde oxime (*trans,anti*-17) with traces of *cis,anti*-**17**

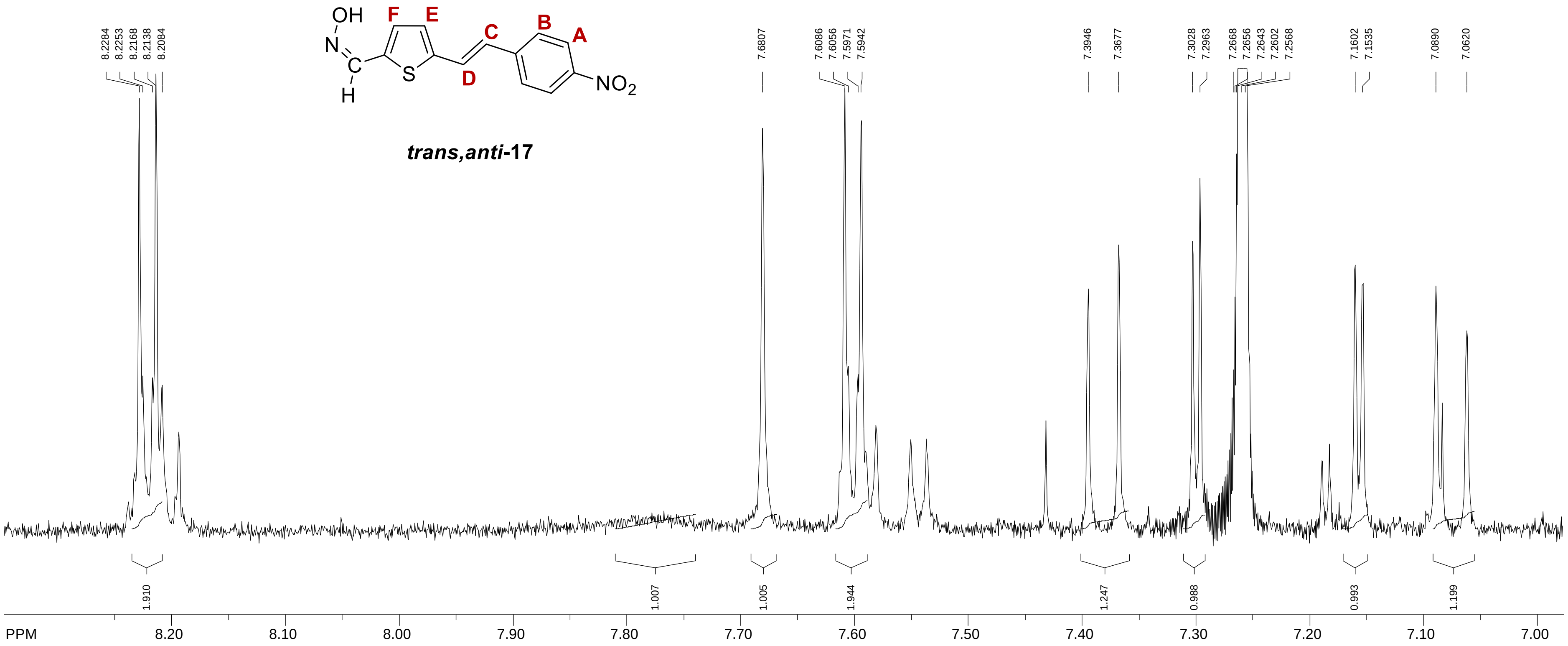

file: D:\PODACI\00 IVANA PUCEK\IvanaPucek diplomski rad\NMR\IP-67_F21-F24\fid expt: <zg30>
transmitter freq.: 600.135401 MHz
time domain size: 32768 points
width: 12019.23 Hz = 20.027532 ppm = 0.366798 Hz/pt
number of scans: 45

freq. of 0 ppm: 600.130010 MHz
processed size: 32768 complex points
LB: 0.000 GB: 0.0000

Mass spectra and HRMS analysis of the mixture of geometrical isomers of 5-(4-nitrostyryl)thiophene-2-carbaldehyde oxime (17)

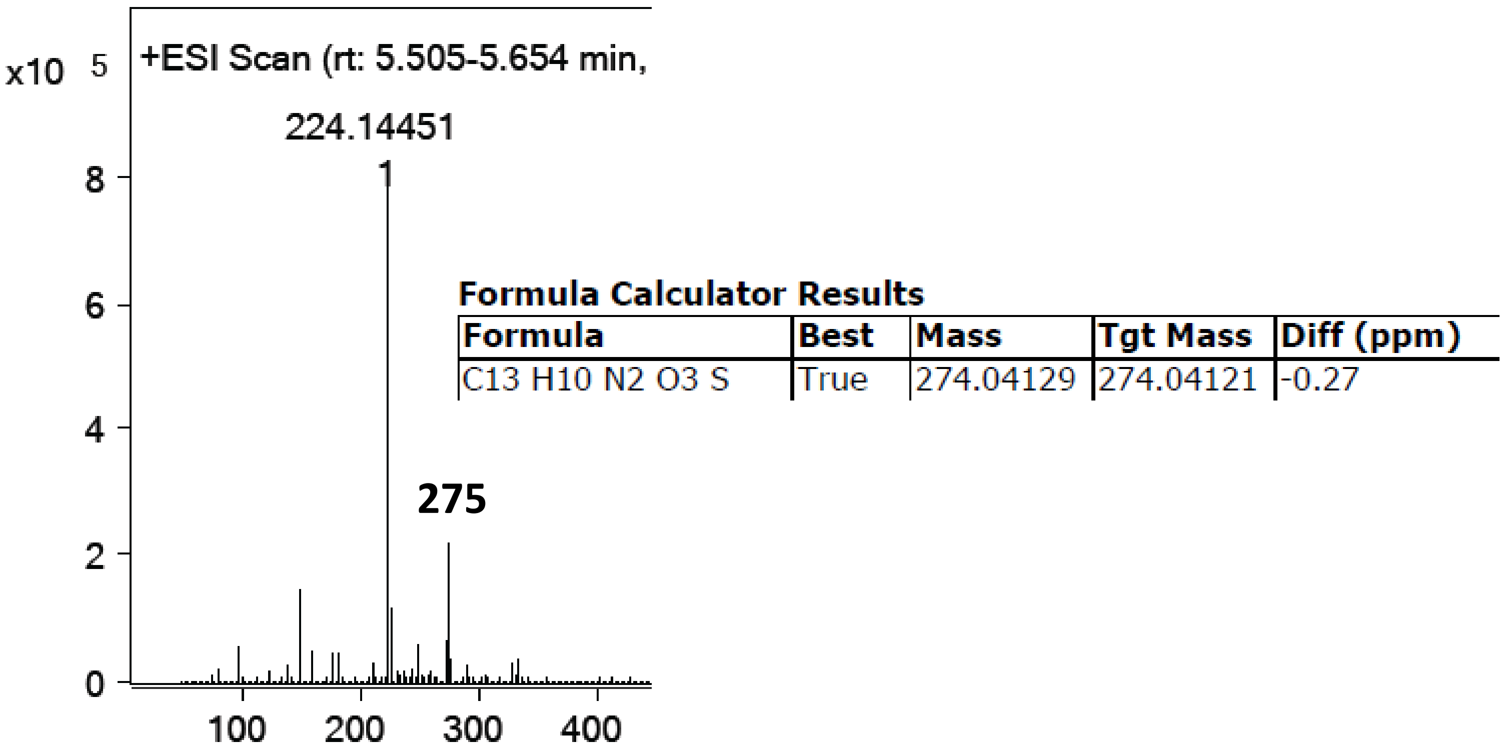

Formula Calculator Results

Formula	Best	Mass	Tgt Mass	Diff (ppm)
C13 H10 N2 O3 S	True	274.04129	274.04121	-0.27

^{1}H NMR spectrum (600 MHz, $CDCl_3$) of *cis,syn*-5-(4-dimethylaminostyryl)thiophene-2-carbaldehyde oxime (*cis,syn*-18)

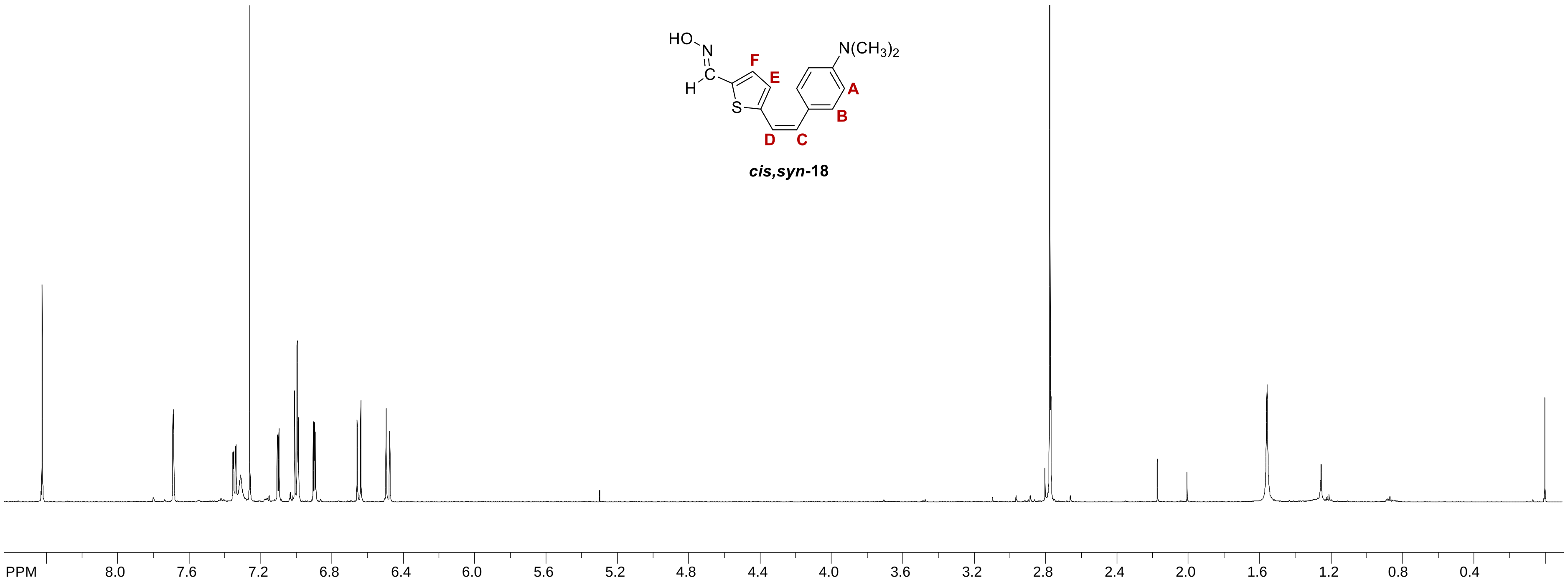

file: D:\SURADNJE\ZRINKA KOVARIK\00 ZK Oximes\SupportingInformation\oksimi cisti spektri MD\oksimi\MD-62(5-10)(41-43)(8) noviji dimetilamino syn cis\fid expt: <zg30>
transmitter freq.: 600.135401 MHz
time domain size: 32768 points
width: 12019.23 Hz = 20.027532 ppm = 0.366798 Hz/pt
number of scans: 22

freq. of 0 ppm: 600.130010 MHz
processed size: 32768 complex points
LB: 0.000 GB: 0.0000

A part of the ^{1}H NMR spectrum (600 MHz, $CDCl_3$) of *cis,syn*-5-(4-dimethylaminostyryl)thiophene-2-carbaldehyde oxime (*cis,syn*-18)

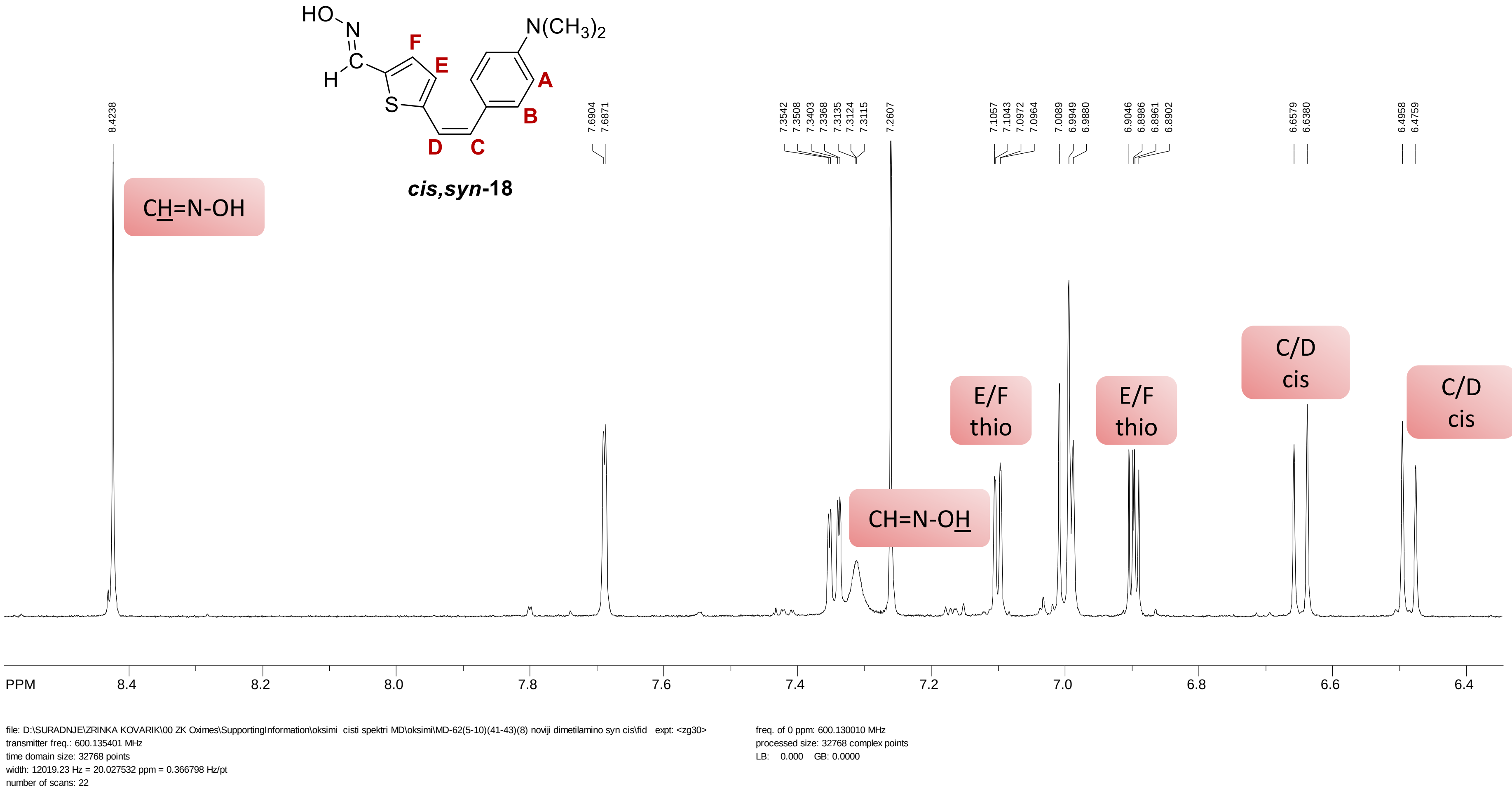

^{13}CNMR spectrum (600 MHz, $CDCl_3$) of *cis,syn*-5-(4-dimethylaminostyryl)thiophene-2-carbaldehyde oxime (*cis,syn*-18)

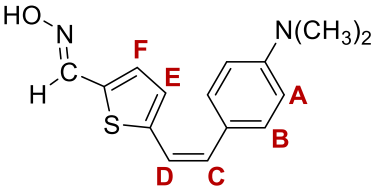

***cis,syn*-18**

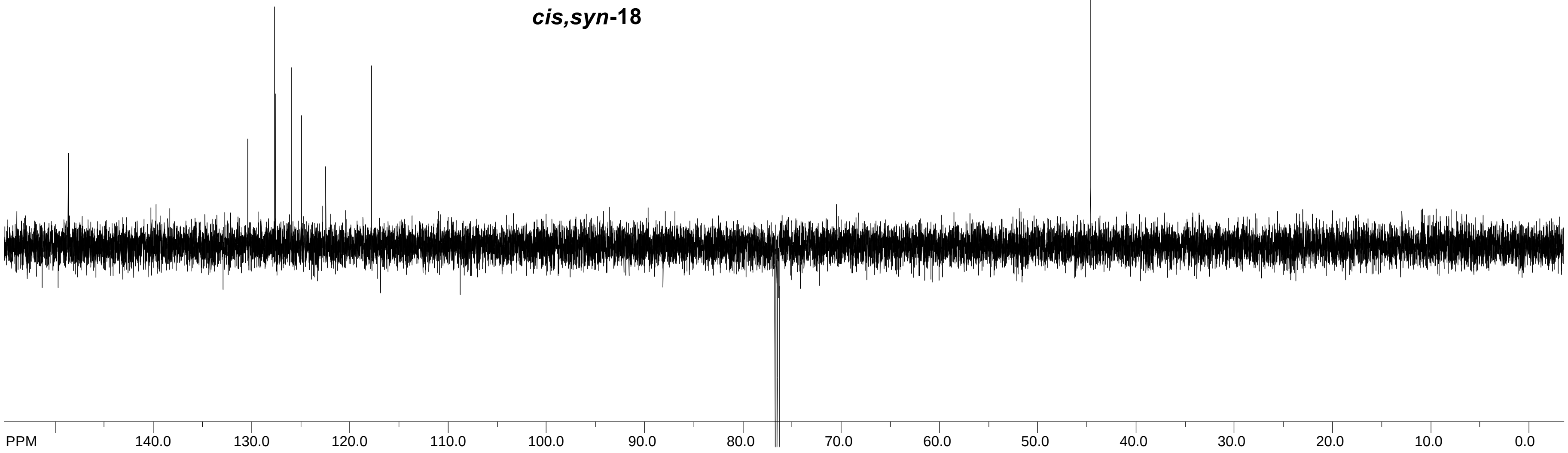

file: D:\SURADNJE\ZRINKA KOVARIK\00 ZK Oximes\SupportingInformation\oksimi cisti spektri MD\oksimi\MD-62(5-10)(41-43)(8) APT\fid expt: <jmod>
transmitter freq.: 150.917899 MHz
time domain size: 65536 points
width: 39370.08 Hz = 260.870838 ppm = 0.600740 Hz/pt
number of scans: 364

freq. of 0 ppm: 150.902884 MHz
processed size: 32768 complex points
LB: 0.000 GB: 0.0000

COSY spectrum of *cis,syn*-5-(4-dimethylaminostyryl)thiophene-2-carbaldehyde oxime (*cis,syn*-18)

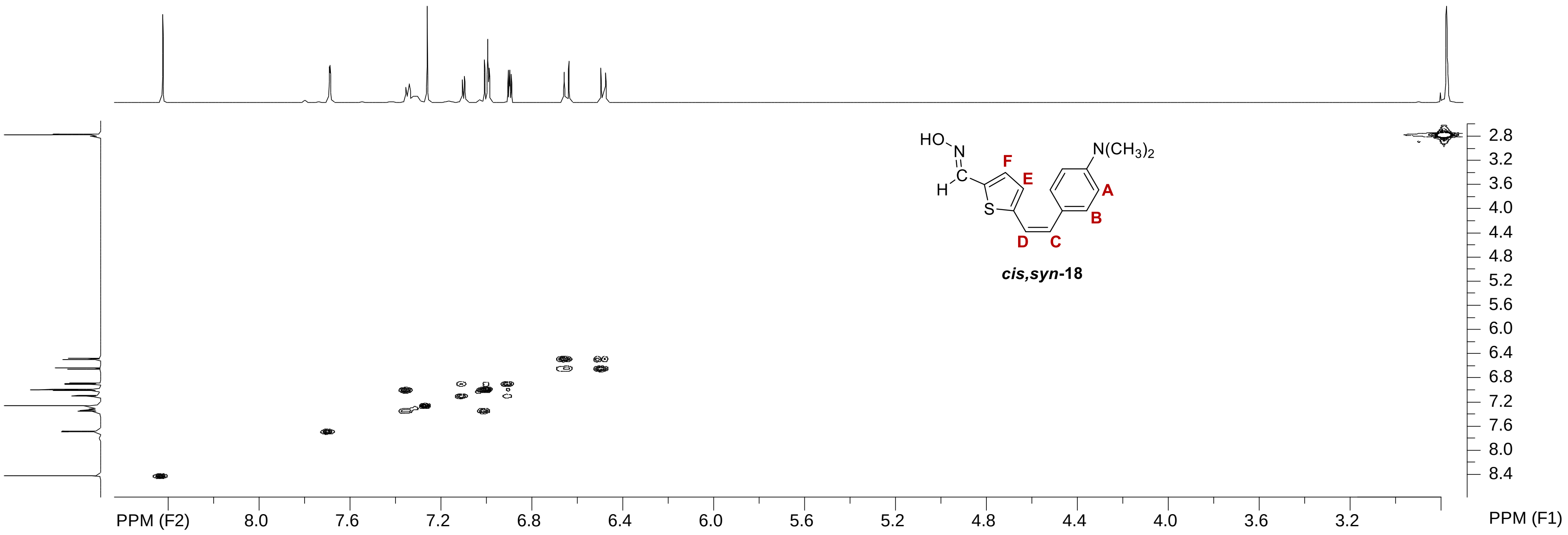

file: D:\SURADNJE\ZRINKA KOVARIK\00 ZK Oximes\SupportingInformation\oksimi cisti spektri MD\oksimi\MD-62(5-10)(41-43)(8) [illegible] 600.130000 MHz
transmitter freq.: 600.133901 MHz
time domain size: 2048 by 512 points
width: 9615.38 Hz = 16.022065 ppm = 4.695012 Hz/pt
number of scans: 2

processed size: 1024 complex points
window function: Sine
shift: 0.0 degrees

F1: freq. of 0 ppm: 600.130000 MHz
processed size: 1024 complex points
window function: Sine
shift: 0.0 degrees

HSQC spectrum of *cis,syn*-5-(4-dimethylaminostyryl)thiophene-2-carbaldehyde oxime (*cis,syn*-18)

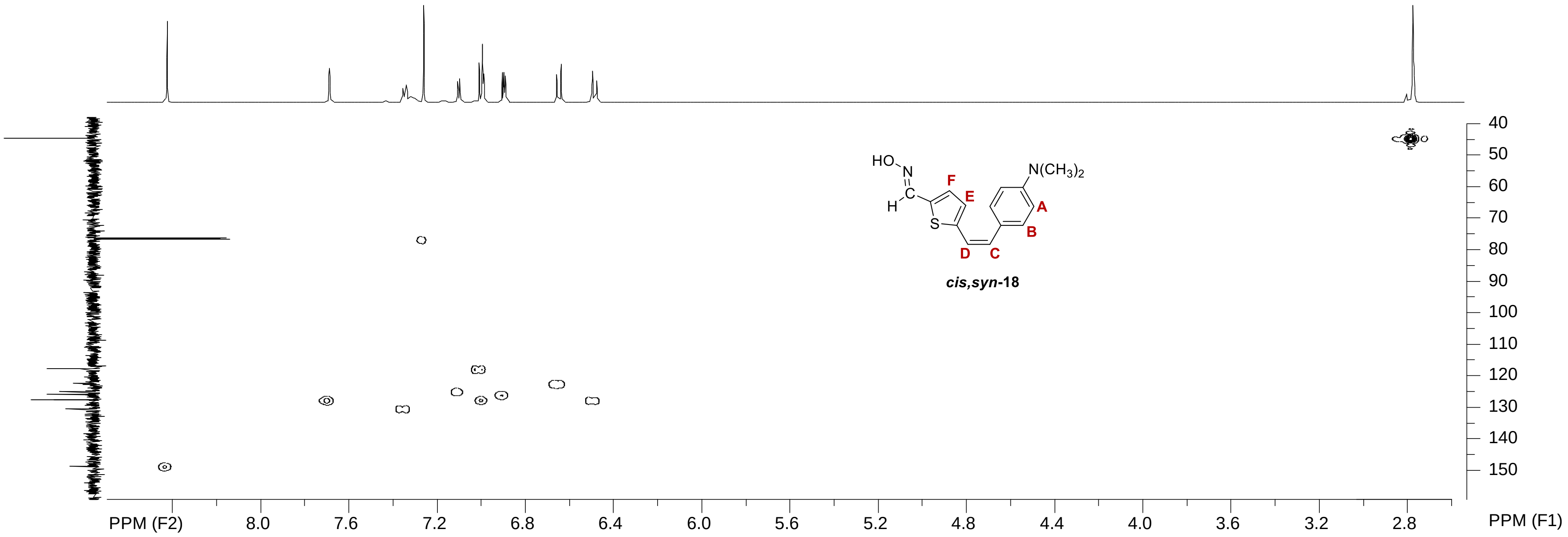

file: D:\SURADNJE\ZRINKA KOVARIK\00 ZK Oximes\SupportingInformation\oksimi cisti spektri MD\oksimi\MD-62(5-10)(41-43)(8) [illegible] MHz
transmitter freq.: 600.133901 MHz
time domain size: 2048 by 226 points
width: 9615.38 Hz = 16.022065 ppm = 4.695012 Hz/pt
number of scans: 16

processed size: 1024 complex points
window function: Sine
shift: 0.0 degrees

F1: freq. of 0 ppm: 150.902809 MHz
processed size: 1024 complex points
window function: Sine
shift: 0.0 degrees

^{1}H NMR spectrum (600 MHz, $CDCl_3$) of *trans,syn*-5-(4-dimethylaminostyryl)thiophene-2-carbaldehyde oxime (*trans,syn*-18) with traces of *cis,syn*-**18**

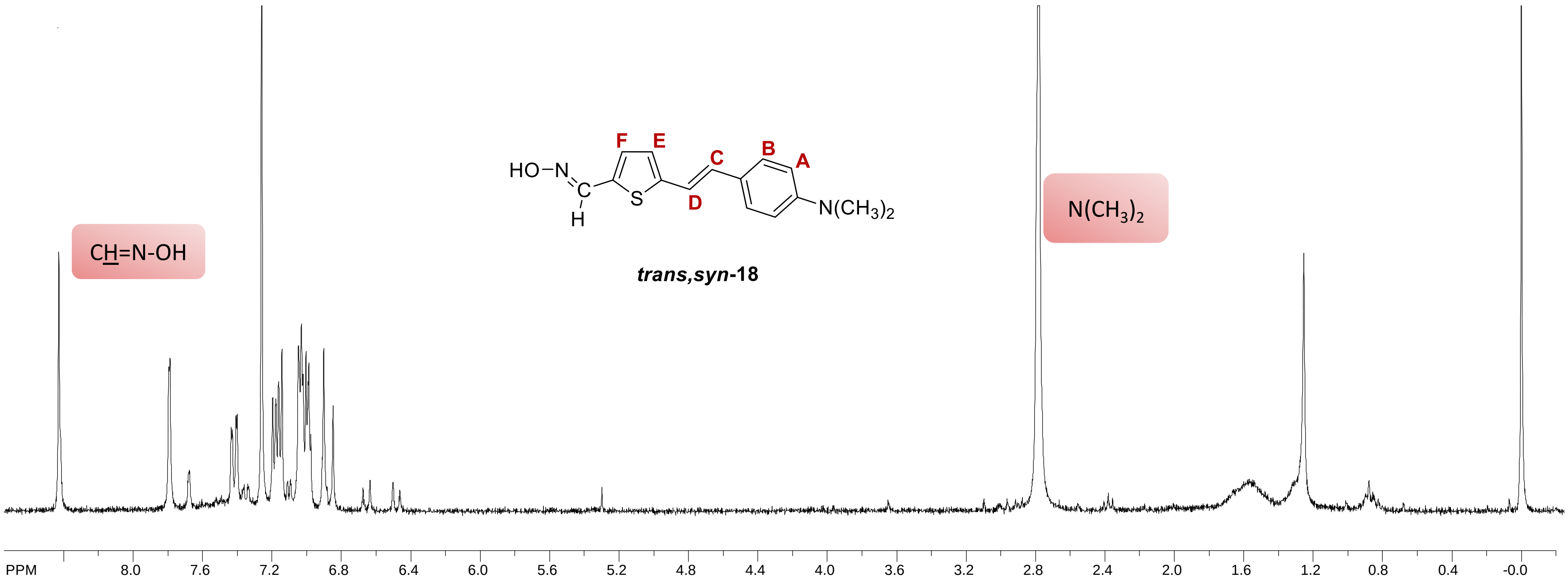

A part of the ^{1}H NMR spectrum (600 MHz, $CDCl_3$) of *trans,syn*-5-(4-dimethylaminostyryl)thiophene-2-carbaldehyde oxime (*trans,syn*-18) with traces of *cis,syn*-**18**

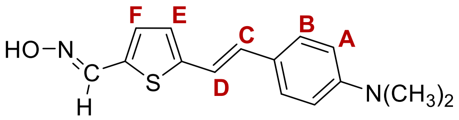

***trans,syn*-18**

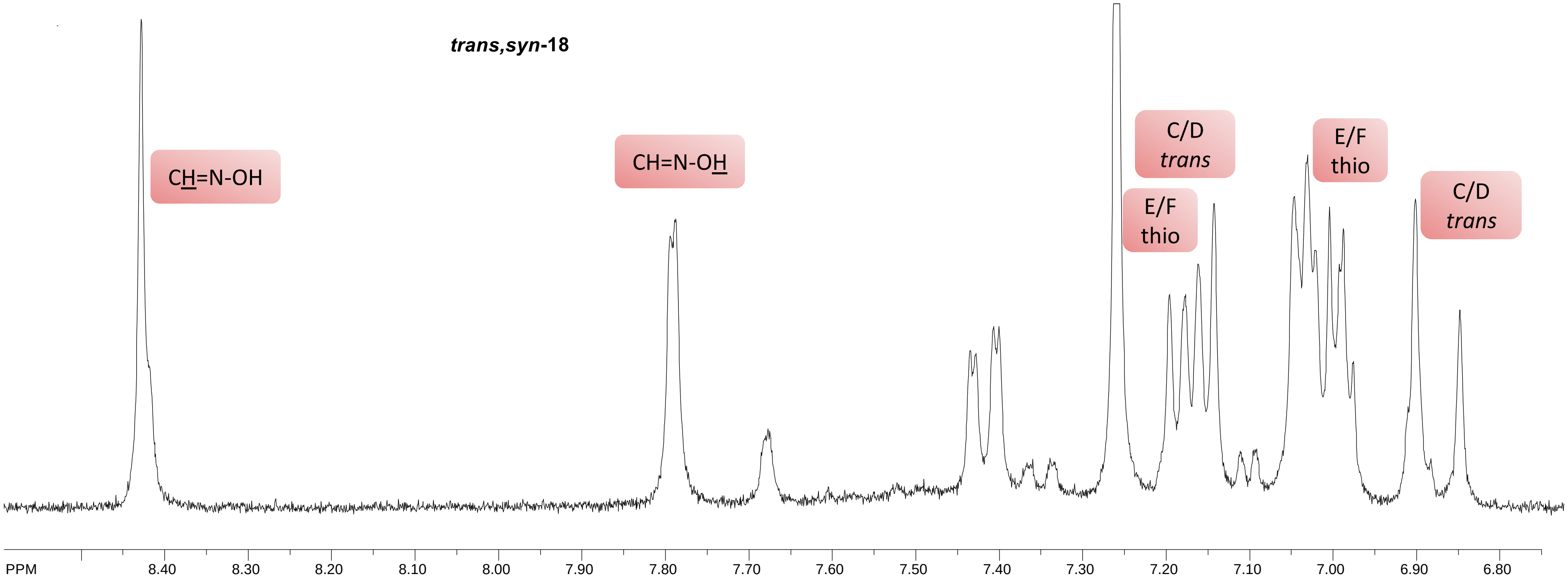

Mass spectra and HRMS analysis of the mixture of geometrical isomers of 5-(4-dimethylaminostyryl)thiophene-2-carbaldehyde oxime (18)

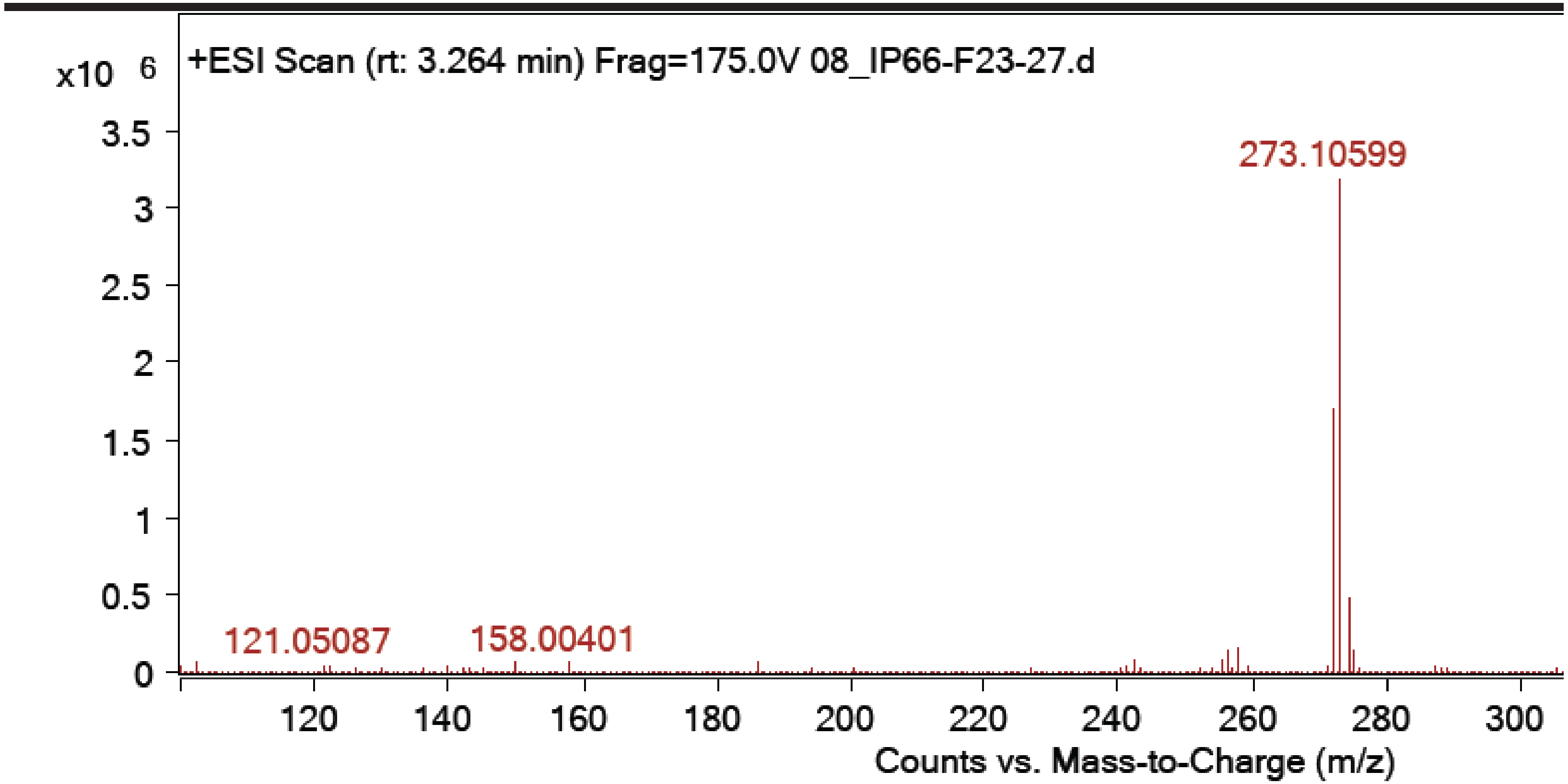

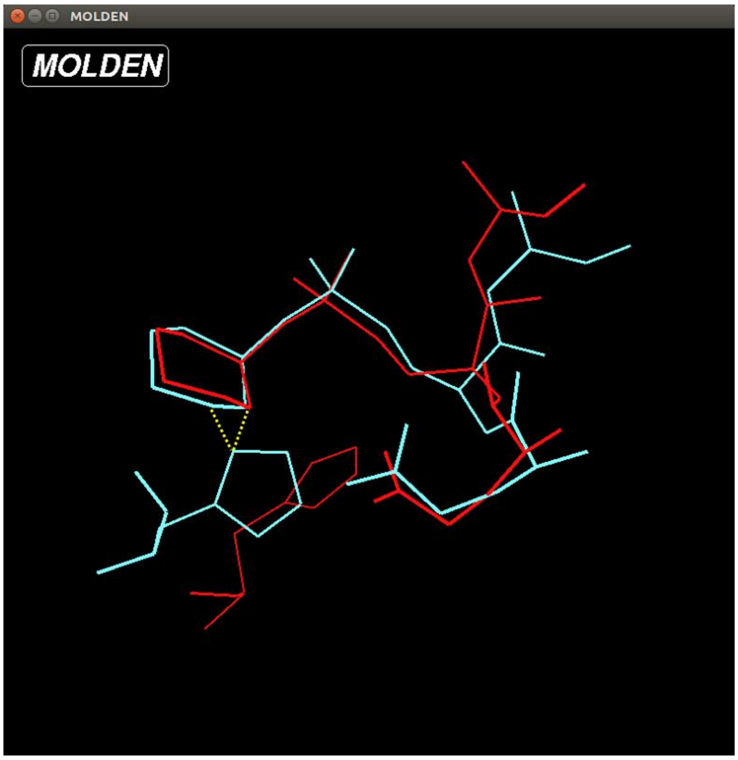

Figure S1. Superposition of the cyclosarin-bound AChE (3ZLU) and BChE (3DJY) with cyclosarin in the same conformation at the active serine.

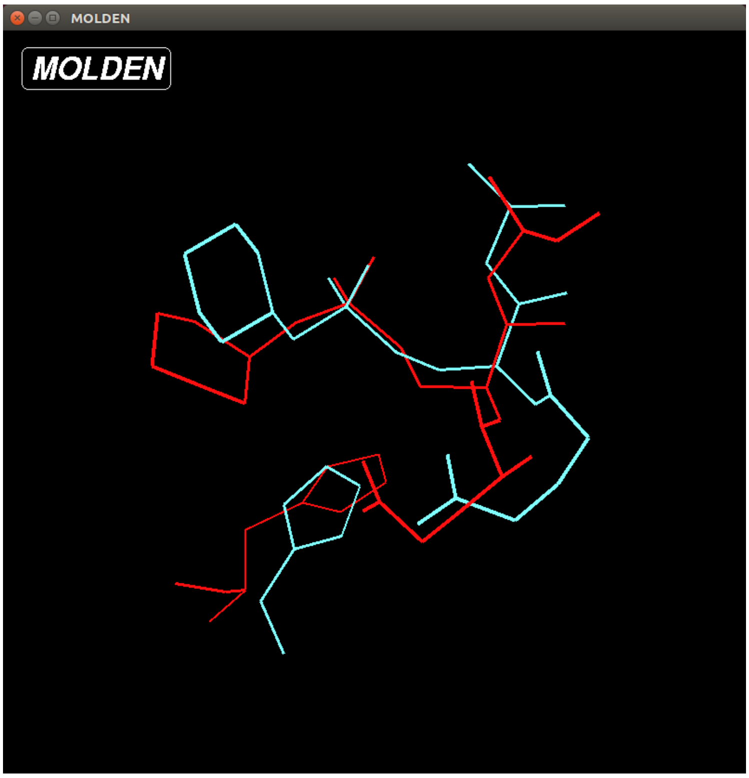

Figure S2. Superposition of cyclosarin-bound AChE (3ZLU) and BChE (3DJY) with cyclosarin bound at the active serine obtained by replacing the dimethylamino and ethoxy groups of tabun with methyl and cyclohexyloxy groups of cyclosarin, respectively.

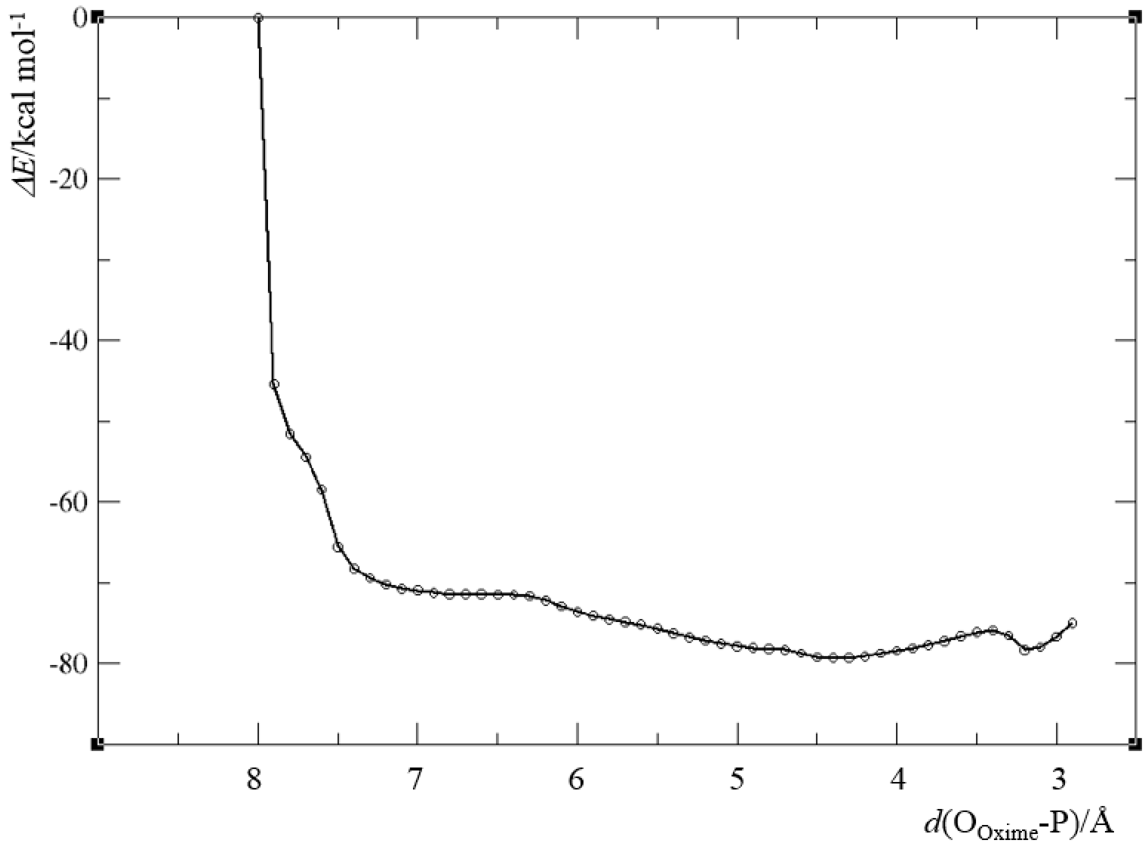

Figure S3. Energy profile for the incremental decrease of the distance between the oxygen of oxime and phosphorus.

Table S1: Data obtained by scanning of PES, presented in Figure S3.

$d(O_{Oxime}$-P)/Å	E/a.u.	ΔE/kcal mol^{-1}
8.0	-3023.14875211	0.0
7.9	-3023.22104022	-45.4
7.8	-3023.23083027	-51.5
7.7	-3023.23559504	-54.5
7.6	-3023.24197973	-58.5
7.5	-3023.25321603	-65.6
7.4	-3023.25750187	-68.2
7.3	-3023.25934329	-69.4
7.2	-3023.26064081	-70.2
7.1	-3023.26148648	-70.7
7.0	-3023.261806	-70.9
6.9	-3023.26221457	-71.2
6.8	-3023.26255554	-71.4
6.7	-3023.26257896	-71.4
6.6	-3023.26257942	-71.4
6.5	-3023.26259845	-71.4

6.4	-3023.26267886	-71.5
6.3	-3023.26292451	-71.6
6.2	-3023.26373582	-72.2
6.1	-3023.26489563	-72.9
6.0	-3023.26602838	-73.6
5.9	-3023.26684957	-74.1
5.8	-3023.26748589	-74.5
5.7	-3023.26807808	-74.9
5.6	-3023.26861376	-75.2
5.5	-3023.26932586	-75.7
5.4	-3023.27027757	-76.3
5.3	-3023.27099308	-76.7
5.2	-3023.27165175	-77.1
5.1	-3023.2722913	-77.5
5.0	-3023.27277758	-77.8
4.9	-3023.27313198	-78.0
4.8	-3023.27338063	-78.2
4.7	-3023.27355393	-78.3
4.6	-3023.27425762	-78.8
4.5	-3023.27491851	-79.2
4.4	-3023.27507675	-79.3
4.3	-3023.27511891	-79.3
4.2	-3023.27477219	-79.1
4.1	-3023.27423352	-78.7
4.0	-3023.27370378	-78.4
3.9	-3023.27320123	-78.1
3.8	-3023.27255992	-77.7
3.7	-3023.27176426	-77.2
3.6	-3023.27086624	-76.6
3.5	-3023.2701283	-76.2
3.4	-3023.26971677	-75.9
3.3	-3023.27073463	-76.5
3.2	-3023.27355892	-78.3
3.1	-3023.27296703	-77.9
3.0	-3023.27091124	-76.7
2.9	-3023.2682795	-75.0
2.8	-3023.26520104	-73.1
2.7	-3023.26183603	-71.0
2.6	-3023.25784468	-68.5
